# Structural Basis for Evasion of Nutritional Immunity by the Pathogenic *Neisseriae*

**DOI:** 10.3389/fmicb.2019.02981

**Published:** 2020-01-10

**Authors:** Ravi Yadav, Nicholas Noinaj, Nicholas Ostan, Trevor Moraes, Julie Stoudenmire, Stavros Maurakis, Cynthia Nau Cornelissen

**Affiliations:** ^1^Markey Center for Structural Biology, Department of Biological Sciences, Purdue Institute of Inflammation, Immunology and Infectious Disease, Purdue University, West Lafayette, IN, United States; ^2^Department of Biochemistry, University of Toronto, Toronto, ON, Canada; ^3^Institute for Biomedical Sciences, Georgia State University, Atlanta, GA, United States

**Keywords:** nutritional immunity, *Neisseria gonorrhoeae*, *Neisseria meningitidis*, transition metals, iron and zinc piracy

## Abstract

The pathogenic *Neisseria* species are human-adapted pathogens that cause quite distinct diseases. *Neisseria gonorrhoeae* causes the common sexually transmitted infection gonorrhea, while *Neisseria meningitidis* causes a potentially lethal form of bacterial meningitis. During infection, both pathogens deploy a number of virulence factors in order to thrive in the host. The focus of this review is on the outer membrane transport systems that enable the *Neisseriae* to utilize host-specific nutrients, including metal-binding proteins such as transferrin and calprotectin. Because acquisition of these critical metals is essential for growth and survival, understanding the structures of receptor-ligand complexes may be an important step in developing preventative or therapeutic strategies focused on thwarting these pathogens. Much can also be learned by comparing structures with antigenic diversity among the transporter sequences, as conserved functional domains in these essential transporters could represent the pathogens’ “Achilles heel.” Toward this goal, we present known or modeled structures for the transport systems produced by the pathogenic *Neisseria* species, overlapped with sequence diversity derived by comparing hundreds of neisserial protein sequences. Given the concerning increase in *N. gonorrhoeae* incidence and antibiotic resistance, these outer membrane transport systems appear to be excellent targets for new therapies and preventative vaccines.

## Introduction

The pathogenic *Neisseriae* include two obligate human pathogens: *Neisseria gonorrhoeae* and *Neisseria meningitidis*. Although these bacteria cause very different diseases, they are so similar at the genome-sequence level that they should be referred to as the same species according to threshold values currently recommended to differentiate species (Kim et al., 2014). *N. gonorrhoeae* causes the common sexually transmitted infection (STI) gonorrhea, which is associated with low mortality but high morbidity, particularly among women. Uncomplicated infections include urethritis in men and cervicitis in women; however, asymptomatic infections are common among women and, thus, the pathogen has the opportunity to ascend into the upper reproductive tract to cause salpingitis, pelvic inflammatory disease, ectopic pregnancy or infertility (Hook and Handsfield, 2008). *N. gonorrhoeae* can escape the genital tract entirely, resulting in disseminated gonococcal infection, for which the predominant symptoms are septic arthritis and dermatological eruptions. Due to the asymptomatic nature of many infections in women, these later manifestations are more likely to occur in females (Hook and Handsfield, 2008). Antibiotic resistance among gonococcal isolates is a serious and emergent problem. Of all the isolates collected in the United States in 2017, nearly 50% of isolates are resistant to one or more antibiotics and over 5% of isolates are resistant to at least three antimicrobial agents tested (CDC, 2017). Current therapy recommendations are to treat simultaneously with two drugs: ceftriaxone and azithromycin (CDC, 2015). However, resistance to both drugs has already emerged and therapy failures with this dual-drug approach have recently occurred (Eyre et al., 2018). Gonococcal infections are not protective and thus persons who suffer from this infection are not protected from subsequent episodes (Hedges et al., 1998, 1999). Finally, even after decades of research, there is no vaccine to prevent gonorrhea.

By contrast, *N. meningitidis* can cause rapidly fatal meningitis or meningococcemia. While colonization of the nasopharynx by *N. meningitidis* is common (referred to as carriage), dissemination to the blood or cerebrospinal fluid can be lethal if not treated quickly and appropriately with antimicrobial drugs (Thompson et al., 2006). Fortunately, antibiotic drug resistance has not emerged in *N. meningitidis*, as it has among gonococcal isolates, and penicillin-G is still the drug of choice for treatment of meningococcal infections (Nadel, 2016). In well-resourced countries, cases of meningococcal disease have been decreasing in frequency due to vaccination with efficacious vaccines. Recently, newly licensed vaccines to protect against serogroup B *N. meningitidis* strains have been recommended to high-incidence groups, leading to a further decline in meningococcal disease cases (Atkinson et al., 2016).

Pathogenic *Neisseria* species utilize similar virulence factors to cause disease in their human hosts. A major difference between *N. gonorrhoeae* and *N. meningitidis* is that the former lacks a polysaccharide capsule, which enables the latter to more effectively evade phagocytosis and desiccation (reviewed in Lewis and Ram, 2014). Both *N. gonorrhoeae* and *N. meningitidis* produce pili that are required for adherence to mucosal epithelia, and a series of phase-variable adhesins and invasins, called Opacity or Opa proteins (reviewed in Merz and So, 2000). Other key virulence factors include various other adhesins, an IgA protease, toxic lipooligosaccharide, and a series of outer membrane proteins involved in serum resistance (for review see Quillin and Seifert, 2018). Another key virulence trait is the ability to acquire the necessary nutrients for bacterial replication in the face of the host’s attempts to starve the pathogen of these building blocks. This innate immunity defense is often referred to as nutritional immunity (Kochan, 1973; Weinberg, 1977, 1978), which is the subject of this review.

## Nutritional Immunity and Metals

*N. gonorrhoeae* and *N. meningitidis* are both obligate human pathogens, which necessitates the requirement for these invaders to obtain all necessary nutrients for growth and pathogenesis from within their human hosts. Humans devote a considerable armament to sequestering many of these necessary nutrients for bacterial growth, particularly metals, into niches that pathogens cannot access. This process, which effectively inhibits the growth of human pathogens due to nutrient deprivation, has been referred to as “nutritional immunity” (Kochan, 1973; Weinberg, 1977, 1978). The best characterized forms of nutritional immunity relate to iron sequestration by the host, including the production of proteins such as lactoferrin (Weinberg, 2001) and lipocalin (Miethke and Skerra, 2010), which actively absorb iron and ferric siderophores, respectively. Other human proteins that maintain free iron at scarce levels in human hosts include transferrin and hemoglobin. In the face of these metal chelating proteins, many pathogens are unable to compete for the necessary nutrient, iron, and thus bacterial growth is restricted. In a similar fashion, a recently recognized phenomenon occurs in humans attempting to sequester another metal, zinc (Liuzzi et al., 2005). Nutritional immunity proteins including calprotectin and other so-called S100 proteins actively, and with high affinity, bind to and “hide” zinc from microbial invaders. The S100 family of proteins are EF-hand proteins involved in calcium sensing and chelation and also bind other divalent cations, including zinc and manganese, with high affinity (Zackular et al., 2015). In a number of microbial systems, these proteins have been shown to be powerful suppressants of growth and therefore pathogenesis.

## Iron Piracy and Bacteria

In order to be successful pathogens, bacteria must overcome nutritional immunity imposed by human hosts. One of the most common approaches to acquiring sufficient iron is to deploy bacterial siderophores, which are molecular cages capable of chelating ferric iron with high affinity. The affinity of these compounds for iron is so high that it out-competes that of the human proteins produced for iron sequestration. For example, the affinity of enterobactin for ferric iron has been estimated to be 10^–52^ M (Neilands, 1981), whereas the affinity of transferrin for iron is considerably lower at 10^–30^ M (Davis et al., 1962). However, enterobactin is not an effective virulence factor in many niches due to the production of another host innate immunity protein called lipocalin. Lipocalin sequesters enterobactin so that bacterial producers do not reap the benefits of deploying this siderophore for iron uptake (Borregaard and Cowland, 2006). Some crafty pathogens, including *Salmonella* species, have evolved a parallel pathway to enterobactin biosynthesis in which they further decorate enterobactin with glucose molecules, yielding salmochelin, which effectively evades lipocalin sequestration (Feldmann et al., 2007; Muller et al., 2009). Thus, both pathogens and the human host are evolving in ways that either enable iron uptake (pathogen) or further sequester iron (host) in the ongoing struggle for this necessary nutrient.

## The Structural Basis for Iron Piracy by the *Neisseriae*

The pathogenic *Neisseriae* are somewhat unusual in that they do not produce any siderophores for metal acquisition. Instead, these stealthy pathogens rely primarily on metal extraction directly from human innate immunity proteins, including transferrin (Tf) and lactoferrin (Lf). The focus of the remainder of this review is on the structural basis by which these transporters enable the pathogenic *Neisseria* species to overcome nutritional immunity imposed by the human host.

## Tf-Iron Acquisition System

**FIGURE 1:**
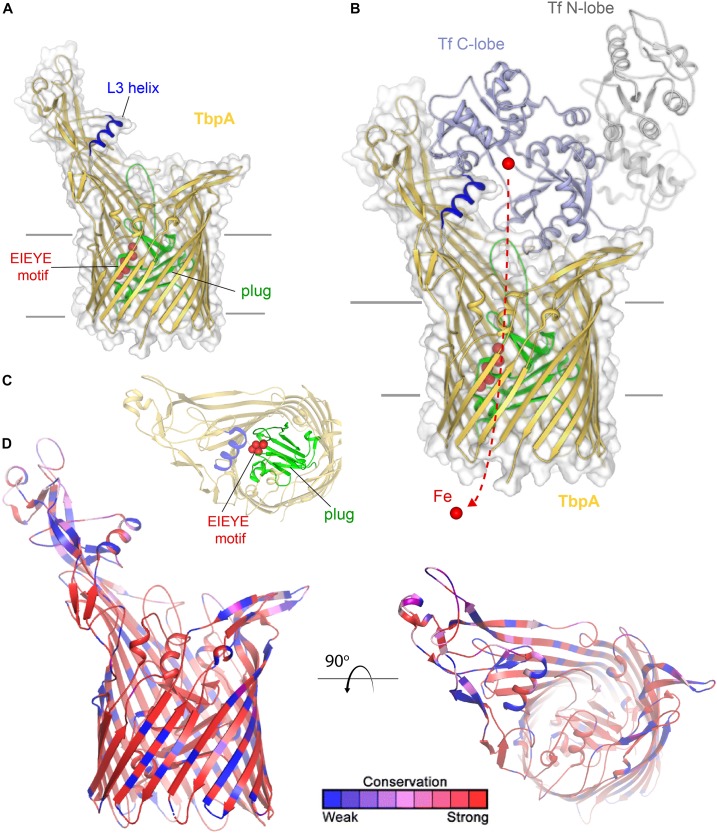
Overall structure of TbpA and interactions with transferrin. **(A)** The structure of TbpA from *N. meningitidis* (K454) (from PDB ID 3V8X) is shown in gold cartoon. The L3 helix is shown in blue, the EIEYE motif in red, and the plug domain in green. The surface is shown in gray transparency. **(B)** The complex of TbpA with apo transferrin (PDB ID 3V8X) is shown with the C-lobe of transferrin in light blue cartoon and the N-lobe in gray cartoon. Iron (red sphere) has been modeled with the red dashed line indicating the putative route through the barrel domain of TbpA during import into the periplasm. **(C)** View looking down the barrel of TbpA showing the plug domain (green) and residues of the EIEYE motif (red spheres). **(D)** The structure of TbpA in cartoon mapping the Consurf conservation scores for *N. meningitidis* and *N. gonorrhoeae* where red is most conserved and blue is the least conserved (also see Supplementary Figure 1).

Transferrin-binding protein A, or TbpA (Noinaj et al., 2012), is a typical TonB-dependent transporter (TdT), which, like all others, possesses 22 transmembrane beta-strands, separated by 11 surface-exposed loops and 11 short periplasmic turns (Figure 1). Also, like other TdTs, TbpA has an amino-terminal plug domain of ∼150 amino acids, which effectively occludes the beta-barrel domain (Noinaj et al., 2012). At least three motifs have been identified in TbpA that are critical to the iron-uptake function of this transporter. As is true for all TdTs, an amino-terminal sequence motif known as the TonB-box is essential for interaction with the charging protein, TonB (Kenney and Cornelissen, 2002). We demonstrated that mutagenesis of the TonB-box abrogated transport function while preserving transferrin-binding capabilities (Kenney and Cornelissen, 2002). On the surface of TbpA is an extruding helix finger protruding from extracellular loop 3 (Figure 1A). The co-crystal structure between TbpA and human transferrin strikingly demonstrated that this helix finger projects into the cleft within the C-lobe of transferrin, which is the only lobe with which TbpA interacts to extract iron (Noinaj et al., 2012) (Figure 1B). This led to the hypothesis that the loop 3 helix is a critical motif with which TbpA manages to remove iron from its ligand. Cash et al. (2015) tested this hypothesis by site-directed mutagenesis and found that changing the charges of the helix or changing charged residues to alanine residues did not fully abrogate either transferrin binding or iron uptake. However, complete deletion of this motif on loop 3 did disable the transporter and prevent *N. gonorrhoeae* from growing on human transferrin as a sole source of iron (Cash et al., 2015). The third motif important for transferrin-iron internalization is within the plug domain. Noto and Cornelissen (2008) and subsequently Banerjee et al. (2012) found that the EIEYE motif in the plug (Figures 1A–C) was necessary for iron utilization from transferrin and moreover for direct interaction between the positively charged ion and the plug. These observations support the hypothesis that after iron is extracted from transferrin, the cation is transiently chelated by the plug. Thus, this protein is effectively a transferrin receptor and iron transporter; it is currently unclear whether TbpA is capable of transporting iron independent of its protein carrier. As shown in Figure 1B, this sequence motif is located in the proposed iron pathway through the TbpA barrel. After the plug is energized by TonB, and presumably moved or removed from the barrel, this change in conformation is sufficient to enable iron removal from the plug and the subsequent hand off of the cation to FbpA (not shown), which is located in the periplasm (Noinaj et al., 2012).

TbpA is very well conserved (Cornelissen et al., 2000) and not subject to high-frequency phase or antigenic variation. Gonococcal TbpA sequences share greater than 95% sequence identity and thus the choice of any single TbpA type as a vaccine antigen is expected to elicit broadly cross-reactive responses. These observations, along with the fact that TbpA is a necessary virulence factor for human infection (Cornelissen et al., 1998), lead to the conclusion that this antigen is a promising vaccine component. As shown in Figure 1D, sequence diversity (shown in blue) is primarily restricted to the long extracellular loops. The plug and barrel domains are very well conserved. Interestingly, even those regions that directly interact with ligand, such as the loop 3 helix, demonstrate some sequence variation. These data suggest that while the charge, conformation, and perhaps the size of the helix finger may be critical for iron extraction, the precise sequence is not. These observations are consistent with those of Cash et al. (2015) who concluded that the helix finger sequence is quite tolerant to mutation and therefore sequence variation.

**FIGURE 2:**
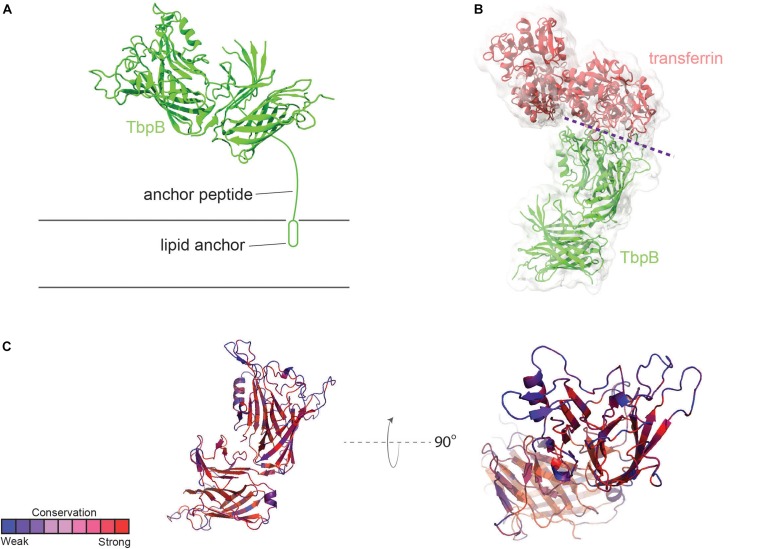
Overall structure of TbpB and interactions with transferrin. **(A)** *N. meningitidis* M982 TbpB (from PDB ID 3VE2) is shown in green cartoon; anchored to the membrane by a lipid moiety. **(B)** The complex of TbpB with transferrin (PDB ID 3VE2) is shown (binding interface represented with dashed purple line). **(C)** Conservation scores of specific residues from AL2CO are typically high within the core barrel and handle (red). Loop regions involved in binding to human transferrin are typically subject to a more intense selection pressure (blue) (also see Supplementary Figure 2).

Transferrin-binding protein B, or TbpB, is a lipid-modified protein (Anderson et al., 1994) that extends outward from the outer membrane via an extended anchor peptide (Figure 2A). Like TbpA, TbpB interacts with human transferrin via the C-lobe of the ligand (Noinaj et al., 2012). TbpB exhibits a bi-lobed structure as well (Moraes et al., 2009; Noinaj et al., 2012), with the N-lobe of TbpB forming a binding interface with the C-lobe of transferrin (Figure 2B). It is unclear what the function of the C-lobe of TbpB is, but this domain may, in part, be important to accomplish export of the lipidated protein to the outer leaflet of the outer membrane via the SLAM system (Hooda et al., 2017). TbpB recognizes only the ferrated form of human transferrin (Cornelissen and Sparling, 1996), enabling this protein to distinguish between the ligand that is useful for iron transport (ferrated) and that which is not (apo form). DeRocco et al. (2008) demonstrated that the presence of TbpB on the gonococcal cell surface enhanced both association with and disassociation from the TbpA transporter. The presence of TbpB enhances iron uptake from transferrin by 50% (Anderson et al., 1994), which is consistent with its observed metal selectivity and ability to form a chamber to trap extracted iron when it complexes with both TbpA and transferrin (Noinaj et al., 2012).

TbpB is considerably more antigenically variable than is TbpA (Cornelissen et al., 1997), although neither protein is subject to high-frequency variation mechanisms as are the pilin or opacity proteins. As shown in Figure 2C, loops of TbpB again show the greatest variation from strain to strain. However, given that the entire protein, including the beta-barrels, is surface-exposed, it is not surprising that immune pressure drives increased antigenic diversity.

Both TbpA and TbpB have been evaluated as possible vaccine targets in a series of studies by Price et al. (2004). Natural infections generate little to no anti-Tbp immune responses, and considering that infections are not protective, these observations engender optimism that generating an anti-Tbp immune response by vaccination could lead to protection. Immunization of mice with full-length Tbp proteins conjugated to the B-subunit of cholera toxin generated immune responses with potentially protective activities (Price et al., 2007). Both mucosal and serum IgA and IgG antibodies were generated in this study. Serum antibodies were capable of fixing human complement and killing Tbp-producing gonococci. Interestingly, vaccination of mice with a cocktail of both TbpA and TbpB conjugates resulted in complement-mediated killing of both the homologous strain and heterologous strains of *N. gonorrhoeae* (Price et al., 2007). Finally, in a third study, Price et al. demonstrated that genetic chimeras consisting of the amino-terminal half of TbpB plus loop 2 of TbpA fused to the A2 subunit of cholera toxin generated both mucosal and serum immune responses. Similarly, the serum antibodies were cross-bactericidal and additionally the pooled mucosal secretions from vaccinated mice were capable of blocking the use of human transferrin by *N. gonorrhoeae in vitro* (Price et al., 2005). These studies cumulatively indicate that the more variable TbpB elicits a vigorous but sequence-specific immune response while addition of TbpA to the vaccine formulation results in cross-reactive, potentially protective immune responses.

## Lf-Iron Acquisition System

**FIGURE 3:**
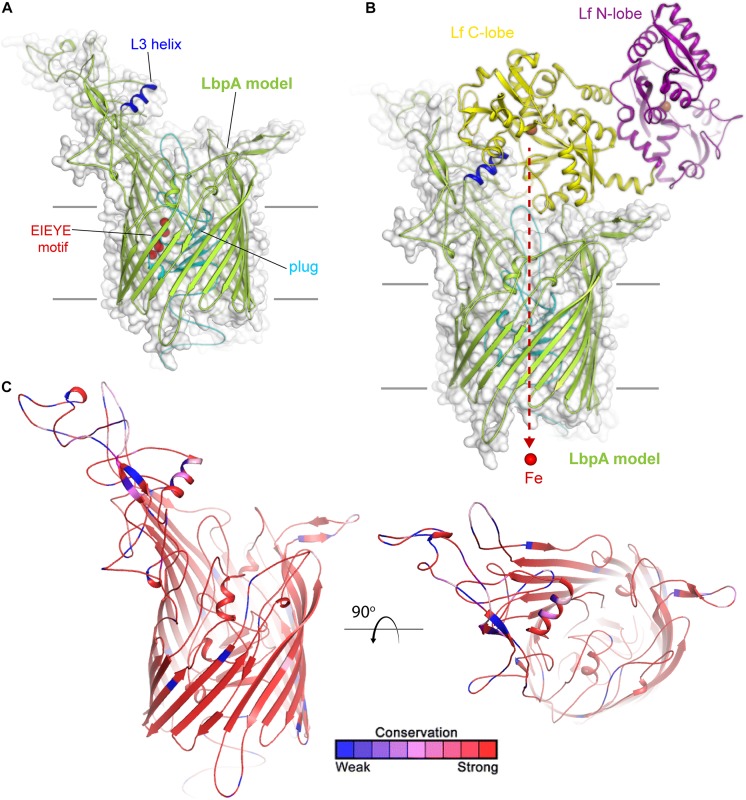
Insights into the structure of LbpA and interactions with lactoferrin. **(A)** Model (SWISS-MODEL) for LbpA from *N. meningitidis* (MC58) is shown in green cartoon. The L3 helix is shown in blue, the EIEYE motif in red, and the plug domain in cyan. The surface is shown in gray transparency. **(B)** Model for the complex of LbpA with holo lactoferrin (PDB ID 1LFG) is shown with the C-lobe of lactoferrin in yellow cartoon and the N-lobe in purple cartoon. This model is based on the reported TbpA-apo transferrin complex structure. Iron (red sphere) has been modeled with the red dashed line indicating the putative route through the barrel domain of LbpA during import into the periplasm. **(C)** The structure of LbpA in cartoon mapping the Consurf conservation scores for *N. meningitidis* and *N. gonorrhoeae* where red is most conserved and blue is the least conserved (also see Supplementary Figure 3).

Much less is known about lactoferrin binding protein A, or LbpA, including its precise crystal structure. However, given the sequence conservation between TbpA and LbpA, high confidence can be afforded to models that demonstrate important structural features of LbpA (Figure 3). The motifs that have been demonstrated to be important for TbpA function can also be identified in LbpA. These motifs include the TonB-box (not shown), the L3 helix and the EIEYE motif. In fact, the EIEYE motif was initially identified by scanning the plug domain for the following residues: conserved in all TbpAs, conserved in all LbpAs, and capable of coordinating iron based on negative charges (Noto and Cornelissen, 2008). Although not directly tested, these motifs are anticipated to function analogously in LbpA to their demonstrated functions in TbpA. Like TbpA, LbpA is quite well conserved (Adamiak et al., 2015). Despite its conservation among strains, LbpA and its lipoprotein partner, LbpB (see next section), are not thought to be particularly good vaccine candidates. In some studies, only approximately half of the gonococcal strains express LbpA (Anderson et al., 2003) and, in many additional isolates, LbpB is phase off due to slipped-strand mispairing (Anderson et al., 2003). Despite these observations, there is concern that if the Tbps are targeted for vaccine development, acquired immunity could drive selection for more strains able to produce the Lbps and therefore utilize lactoferrin as a sole iron source. The potential redundancy in these iron transport systems has been experimentally verified in human infection studies. In studies conducted by Anderson et al. (2003), a genetically engineered strain that could produce the Lbp proteins, but could not produce the Tbps, was capable of causing experimental infection. Moreover, a strain producing both iron acquisition systems was more competitive than a strain producing only one of these systems (Anderson et al., 2003). These studies suggest that perhaps the best approach will be to target multiple iron transport systems in a cocktail-type vaccine.

**FIGURE 4:**
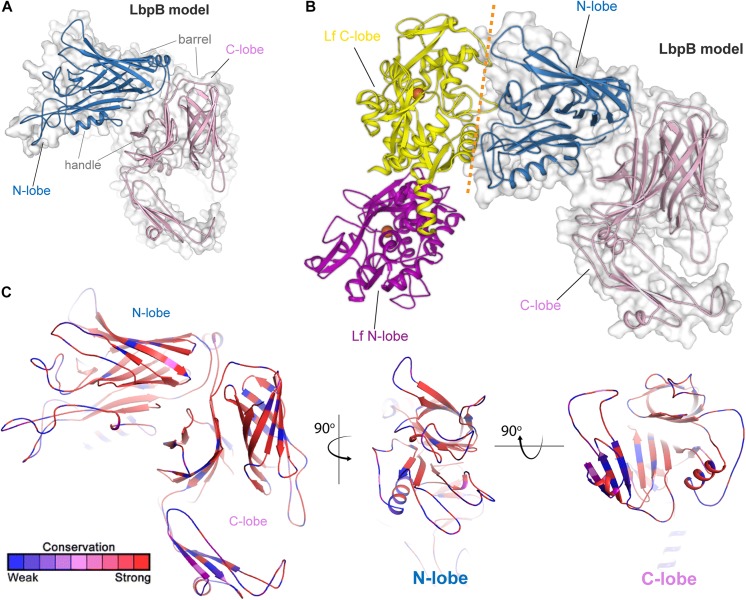
Insights into the structure of LbpB and interactions with lactoferrin. **(A)** Model (SWISS-MODEL and PDB ID 4U9C) for LbpB (MC58) from *N. meningitidis* is shown in blue (N-lobe) and pink (C-lobe) cartoon. The barrel and handle domains are conserved motifs and indicated for each lobe. The surface is shown in gray transparency. **(B)** Model for the complex of LbpB with lactoferrin (PDB ID 1LFG) is shown with the C-lobe of lactoferrin in yellow cartoon and the N-lobe in purple cartoon. Lactoferrin-bound irons are indicated as orange spheres with the red dashed line indicating putative binding interface between lactoferrin and LbpB. This model is based on the reported TbpB-holo transferrin complex structure (PDB ID 3VE1). **(C)** Model of LbpB in cartoon mapping the Consurf conservation scores for *N. meningitidis* and *N. gonorrhoeae* where red is most conserved and blue is the least conserved (also see Supplementary Figure 4). Orthogonal views of the N-lobe and C-lobe are shown for comparison.

Lactoferrin-binding protein B, or LbpB (Figure 4) is, like its transferrin-binding homolog, a lipoprotein that is entirely exposed on the cell surface and tethered to the cell by a lipid anchor (Noinaj et al., 2013). Similar to LbpA, no full-length structure has yet been reported for LbpB; however, structures of the N-lobe only have been determined (Brooks et al., 2014). As expected based upon homology (Noinaj et al., 2013), the conformation of this protein resembles that of TbpB, including a bilobed structure with both barrel and handle domains (Figures 4A,B). Similar to TbpB, it is anticipated that the N-lobe of LbpB interacts exclusively with the C-lobe of human lactoferrin and thus increases the efficiency of iron transport from lactoferrin. Sequence diversity for LbpB resembles that of TbpB in that the solvent-exposed loops are highly diverse (Figure 4C). However, the beta-barrels are better conserved than those in TbpB, perhaps reflecting the fact that this protein is often not produced *in vivo*, thus an immune response does not drive antigenic diversity. Lastly, *N. meningitidis* selectively expresses NalP, which is a phase-variable autotransporter/lipoprotein hybrid that houses a subtilisin-like protease domain responsible for releasing LbpB from the bacterial membrane (Roussel-Jazede et al., 2010; Del Tordello et al., 2014). NalP is thus believed to help divert host immune factors away from the bacterial cell surface during invasive infection, again suggesting that LbpB is likely a poor vaccine candidate for protection against *N. meningitidis*. NalP is, however, exclusive to *N. meningitidis* and thus whether similar evasion mechanisms exist in *N. gonorrhoeae* is currently unknown.

## Hb-Iron Acquisition Systems

The genomes of the pathogenic *Neisseria* contain the genetic capacity to express two distinct hemoglobin acquisition systems. HmbR or HmbR plus HpuAB are most commonly produced by *N. meningitidis* strains (Harrison et al., 2013); however, all *N. gonorrhoeae* strains characterized to date only produce HpuAB since the *hmbR* gene is a pseudogene (Harrison et al., 2013). Moreover, the *hpuAB* genes in both pathogenic species are subject to phase variation (Chen et al., 1998; Lewis et al., 1999). Interestingly, several species of commensal *Neisseriae* do not have the repeat region upstream of *hpuAB*, resulting in expression that is not subject to high-frequency phase variation (Harrison et al., 2013).

**FIGURE 5:**
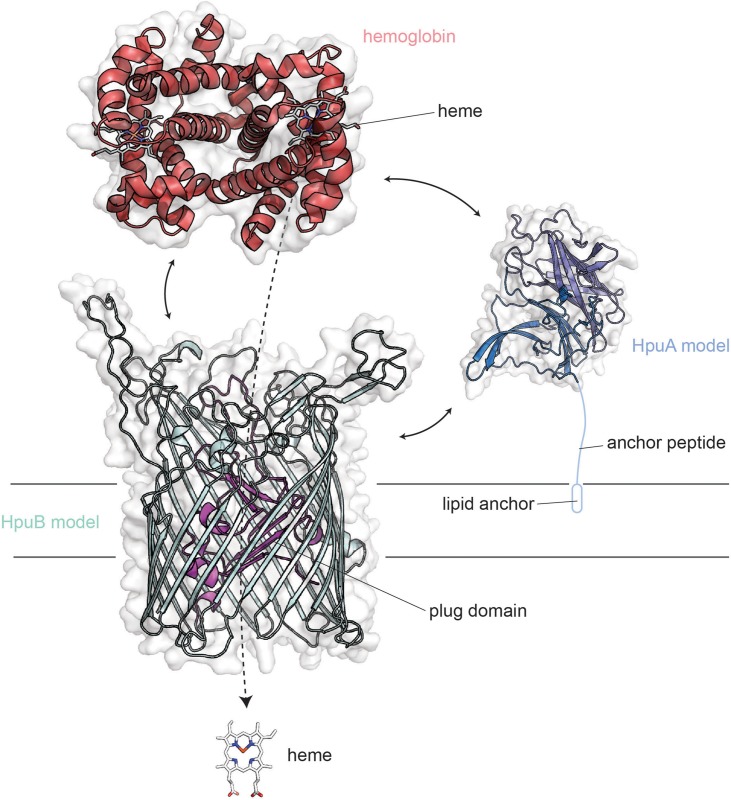
Overall structures of HpuA/B visualized with human hemoglobin. HpuA model (blue; SWISS-MODEL) is shown anchored to the membrane via a lipidated anchor peptide. HpuB (teal; SWISS-MODEL) is shown embedded within the outer membrane. These two proteins act in concert to form a ternary complex and pirate heme from host hemoglobin (red).

The *hpuAB* locus encodes two proteins, HpuA and HpuB, which are capable of binding to hemoglobin (Figure 5) and also to the hemoglobin:haptoglobin complex (Lewis et al., 1997) (not shown). HpuA is the lipoprotein component of the system (Lewis et al., 1997) and is anticipated to take on a conformation that is fully extended outward from the cell surface. HpuB is the TdT component that enables the entry of heme after its extraction from hemoglobin. Unlike the transferrin and lactoferrin transport systems, ligand binding and utilization requires the participation of both HpuA and HpuB (Chen et al., 2002). The HpuA protein is roughly half the size of the other lipoprotein transport components but structurally resembles the C-lobe of both TbpB and LbpB (Wong et al., 2015). Much less is known about the structure-function relationships of the HpuAB proteins; however, the crystal structure has been reported for a related HpuA from *Kingella* (Wong et al., 2015), which was used to form the homology model for neisserial HpuA, shown in Figure 5.

HpuA and B are produced by both pathogenic *Neisseria* species but are also subject to high-frequency phase variation (Lewis et al., 1997, 1999). Some virulent clonal populations of *N. meningitidis* possess both HpuAB and HmbR gene loci suggesting there may be a benefit to producing both systems, particularly in systemic infections (Harrison et al., 2013). However, no *N. gonorrhoeae* strains produce HmbR (Harrison et al., 2013), and in all of the infected men tested, the variants isolated *in vivo* were phase-off for the *hpuAB* system (Anderson et al., 2001). In the same study, Anderson et al. found that most women with a localized infection by *N. gonorrhoeae* similarly were culture positive for isolates that were phase-off for the *hpuAB* system. The only group from which phase-on *hpuAB* variants were isolated were women infected during the first 2-weeks of their menstrual cycle (Anderson et al., 2001). These observations cumulatively suggest that during localized *N. gonorrhoeae* infections, the HpuAB hemoglobin acquisition system is not critical for survival or pathogenesis. However, when hemoglobin is plentiful, such as during menses, this system is selected for as the ligand is available and could potentially perpetuate the infection (Anderson et al., 2001). In fact, ascending infections in women are associated with the onset of menses (Jossens et al., 1996; Korn et al., 1998; Holder, 2008).

**FIGURE 6:**
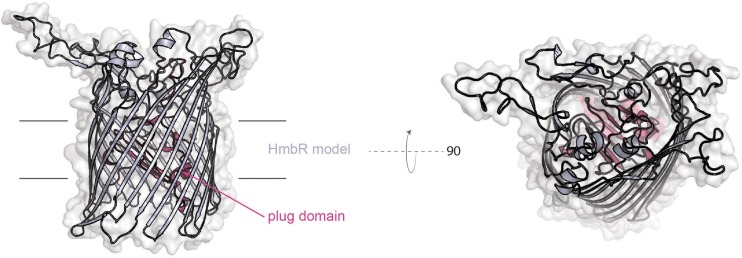
Model of *N. meningitidis* (z4242) HmbR created with SWISS-MODEL.

HmbR (Figure 6) is only produced by some *N. meningitidis* isolates and a few commensal *Neisseria* species (Harrison et al., 2013). Like the HpuAB system, HmbR is subject to high-frequency phase variation by slipped-strand mispairing (Lewis et al., 1999). This protein is a TdT, which unlike those discussed above, does not have a lipoprotein partner. Possible contributions for the lipoprotein components of these systems are: an immunogenic shield for the conserved transporter (Price et al., 2005), and to impose specificity for the ligand that is useful for metal uptake (Cornelissen and Sparling, 1996; Noinaj et al., 2012). The fact that HmbR can function alone without a lipoprotein partner suggests that *in vivo* selection of hemoglobin for heme uptake does not require discrimination between the loaded form and the apo form. Alternatively, the absence of a lipoprotein component could suggest that immunological shielding is not as necessary to hide this protein from immune surveillance. A meningococcal mutant unable to produce HmbR was defective in an infant rat model of infection (Stojiljkovic et al., 1995); however, a similar mutant was not attenuated for growth in human blood, although a *tbpAB* mutant was not capable of growth in human blood (Bidmos et al., 2015). These data suggest that the use of transferrin in human blood is preferred over hemoglobin by the meningococcus, perhaps because hemoglobin is less available under normal conditions without significant red blood cell lysis.

## Siderophore-Iron Acquisition System

**FIGURE 7:**
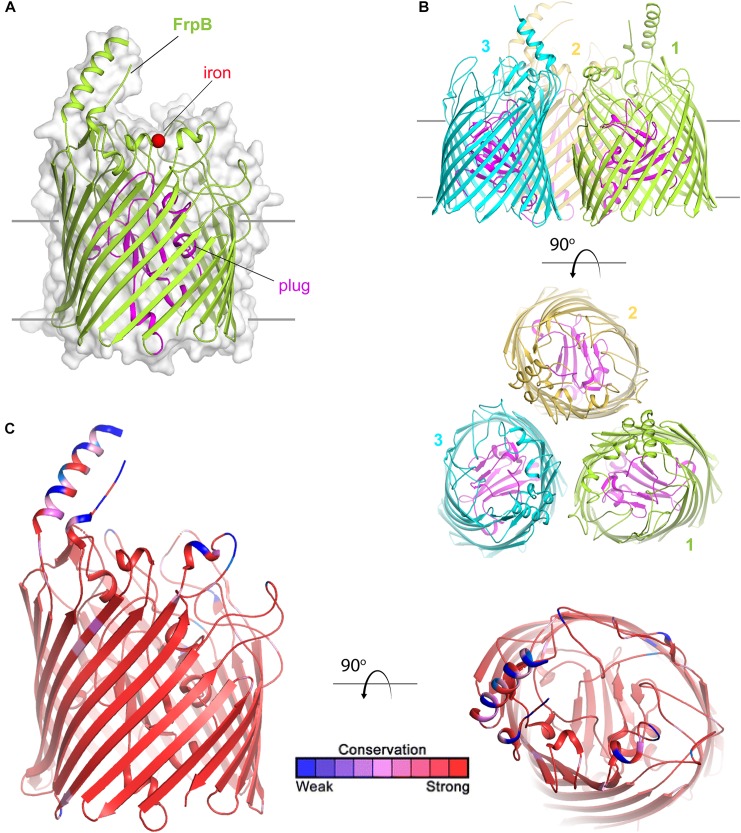
Overall structure of FetA (FrpB). **(A)** The structure of FetA from *N. meningitidis* (Variant F5-1) (from PDB ID 4AIQ) is shown in green cartoon. The bound iron is shown as a red sphere and the plug domain in magenta. The surface is shown in gray transparency. **(B)** The trimeric form of FetA (Variant F3-3) (PDB ID 4AIP) is shown in cartoon with each monomer in a different color. The plug domains are in magenta. **(C)** The structure of FetA in cartoon mapping the Consurf conservation scores for *N. meningitidis* and *N. gonorrhoeae* where red is most conserved and blue is the least conserved (also see Supplementary Figure 5 for neisserial FetA).

FetA (formerly called FrpB) is a TdT (Figure 7) than enables internalization of siderophores that the *Neisseria* species themselves do not have the capacity to produce. Originally named for its ability to transport ferric enterobactin (Carson et al., 1999), Hollander et al. demonstrated that this transporter has a broader ligand specificity than first realized (Hollander et al., 2011). FetA can internalize not only ferric-enterobactin, but also ferric-salmochelin, and dihydroxybenzoate monomers of these siderophores (Hollander et al., 2011). Recently, the crystal structure of FetA (Saleem et al., 2013) demonstrated two interesting features. First, consistent with the broad ligand specificity, free iron was directly coordinated to FetA in the crystal structure (Saleem et al., 2013) (Figure 7A). Second, as with the *Escherichia coli* enterobactin transporter, FetA is suggested to form a trimer (Saleem et al., 2013) (Figure 7B). The authors concluded that FetA is an iron transporter and has the potential to take up unchelated iron in the absence of siderophore (Saleem et al., 2013). TbpA similarly has the capacity to interact with the iron cation and could also contribute to free iron internalization. However, *in vivo* free iron concentrations are exceedingly low, thus it seems unlikely that iron internalization without extraction from some chelating or binding entity is physiologically relevant.

Saleem et al. (2013) demonstrated that conserved residues responsible for coordination of free iron to FetA are also found in similar positions in heme transporters and in other FetA homologs in all of the pathogenic *Neisseria*. However, a hypervariable loop and helix (Figure 7C) is located near this iron binding pocket. The authors of this study (Saleem et al., 2013) suggest that the helix essentially shields the vulnerable iron binding residues from immune detection, and further, that the structure of the helix is maintained despite antigenic diversity which is evoked by immunological pressure.

Similar to the hemoglobin and lactoferrin transporters, expression of the *fetA* gene is subject to high-frequency phase variation (Carson et al., 2000). But unlike in the previous cases, the slipped-strand in the *fetA* gene lies upstream within the promoter region. An increase or decrease in the number of C residues in the promoter, changes the distance between the −10 and −35 elements and therefore changes the strength of the promoter (Carson et al., 2000). Thus, the variation deployed in this system is more like a rheostat than a light switch.

## Zn-Acquisition Systems

**FIGURE 8:**
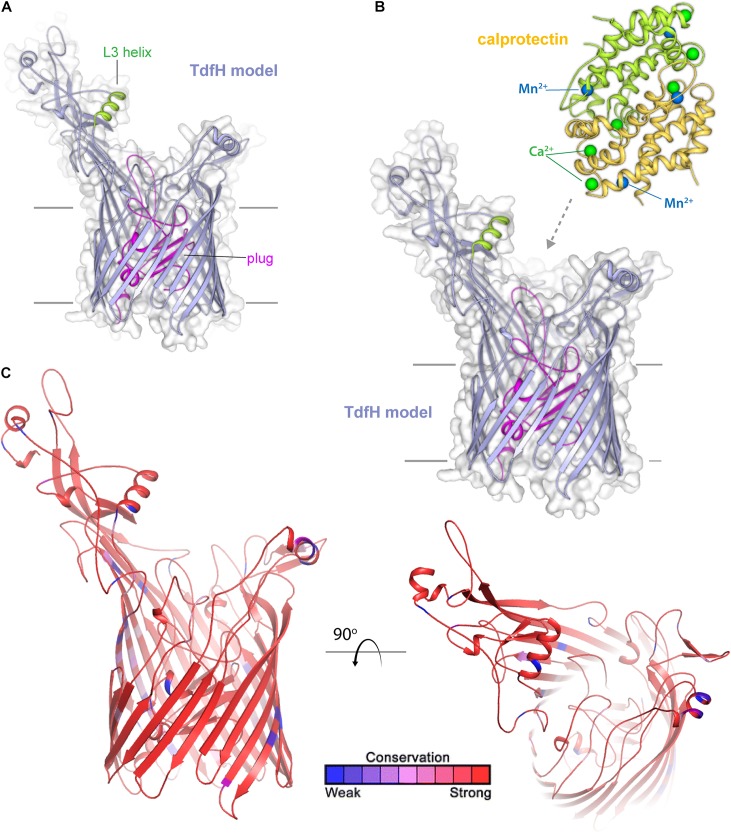
Insights into the structure of TdfH and interactions with calprotectin. **(A)** Model (Phyre; based on TbpA) for TdfH from *N. gonorrhoeae* (FA1090) is shown in violet cartoon. The L3 helix is shown in green and the plug domain in magenta. The surface is shown in gray transparency. **(B)** Model of TdfH in proximity with calprotectin (PDB ID 4GGF) shown in green/yellow cartoon. Calcium ions are shown as green spheres and manganese metals are shown in blue spheres; zinc has been shown to replace the manganese metals with no overall structural changes to calprotectin (personal communication). While the molecular interactions between TdfH and calprotectin have not been reported, the interaction shown here is loosely modeled based on work with other transporters (PDB IDs 3CSN and 3V8X). **(C)** The model of TdfH in cartoon mapping the Consurf conservation scores for *N. meningitidis* and *N. gonorrhoeae* where red is most conserved and blue is the least conserved (also see Supplementary Figure 6).

TdfH (CbpA) (Figure 8) is expressed by both of the pathogenic *Neisseria* species but by very few commensal *Neisseria* species. TdfH is a TdT that shows both sequence similarity to TbpA and HasR from *Serratia marcescens* (Figure 8A). The homolog of this protein was named CbpA by Stork et al. (2013) when they demonstrated that this transporter bound to the human protein, calprotectin (Figure 8B). Calprotectin is found in extremely high concentrations in neutrophils (Zackular et al., 2015) and also in neutrophil NETs (Jean et al., 2016). This innate immunity protein sequesters metals, including zinc and manganese, and in doing so inhibits microbial growth (Zackular et al., 2015). The ability to overcome this sequestration by the pathogenic *Neisseria* species enables them to usurp nutritional immunity and in fact take advantage of this prevalent protein as a metal source. In fact, Jean et al. (2016) demonstrated that gonococcal TdfH enabled calprotectin binding and subsequent zinc incorporation into the cell in a TdfH-dependent manner. In addition, the same study demonstrated that a mutant unable to produce TdfH was less able to survive within neutrophil NETs, suggesting that TdfH is an important virulence factor. Unlike many of the other transport systems deployed by the pathogenic *Neisseria*, TdfH does not have a lipoprotein component partner. Furthermore, TdfH is not subject to variation via slipped-strand mispairing or any other high frequency mechanism. There is some variation from strain to strain among the *Neisseriae*, but the amino acid changes are modest and primarily limited to hypothetically surface-exposed loops (Figure 8C).

**FIGURE 9:**
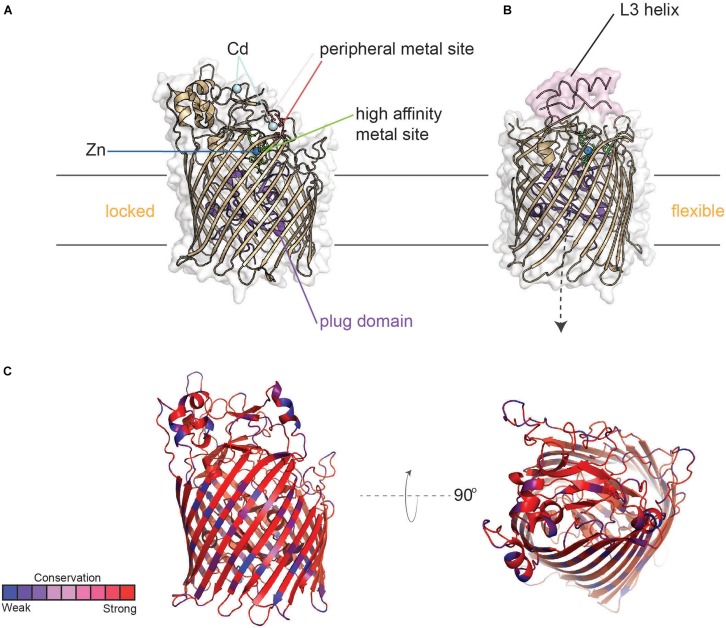
Structure of TdfJ (ZnuD) in locked and flexible conformations. **(A)** Crystal structure of TdfJ (ZnuD) (PDB ID 4RDR) in the locked conformation. **(B)** Crystal structure of TdfJ (ZnuD) in the flexible conformation (PDB ID 4RDT). **(C)** TdfJ (ZnuD) with primary sequence conservation scores from AL2CO mapped onto the tertiary structure for 78 distinct (90% sequence identity cutoff threshold) *N. meningitidis* isolates from BigsDB (also see Supplementary Figure 7).

TdfJ (ZnuD) (Figure 9) is a highly conserved TdT that is also expressed by both of the pathogenic *Neisseria* species, but unlike TdfH, TdfJ is also found ubiquitously amongst the commensal *Neisseriae*. Stork et al. (2010) first characterized the meningococcal homolog of this protein as contributing to zinc uptake, hence its appellation ZnuD, and this function was recapitulated for TdfJ by Jean et al. (2016), by showing that TdfJ contributes to gonococcal growth in Zn-restricted conditions. Further investigation of TdfJ revealed that this protein is able to bind to human S100A7, from which it facilitates zinc removal and subsequent internalization by the gonococcus (Maurakis et al., 2019). S100A7, like the TdfH ligand calprotectin, inhibits growth of many microbes by means of metal sequestration (reviewed by Zackular et al., 2015). S100A7 is highly enriched in human epithelial tissue, including those of the oral and genital mucosa, and indeed a meningococcal mutant unable to produce the TdfJ homolog, ZnuD, was defective for survival within epithelial cells (Kumar et al., 2012). While it is not currently known whether ZnuD shares the S100A7 binding ability of TdfJ, these results suggest that interaction with S100A7 may play a critical role in establishing infection.

Similar to TdfH, TdfJ lacks a lipid-modified accessory protein like TbpB; however, the crystal structure of ZnuD from *N. meningitidis* shows that extracellular loop 3 contains an exposed helix similar to that of TbpA which coordinates transferrin utilization. Strikingly, ZnuD loop 3 is flanked on each side by a His/Asp-rich region that senses zinc (Calmettes et al., 2015), so it is possible that this helix finger in TdfJ may play a role in the interaction with S100A7, but this has not yet been tested. Interestingly, ZnuD undergoes a significant conformational change upon zinc binding. By remodeling the external loop structures, ZnuD goes from a “locked” position (Figure 9A) to a more “flexible” state upon zinc binding (Figure 9B), allowing for metal sequestration in a buried high-affinity site (Calmettes et al., 2015). Like the other TdTs discussed above, TdfJ (ZnuD) is well conserved, but has some sequence variation strain-to-strain within the *Neisseria* species (Figure 9C). TdfJ is also not subject to high-frequency phase or antigenic variation. As seen with the Tbps and Lbps, it is possible that the two aforementioned zinc acquisition systems create redundancy, but experimentation is needed to test for any compensatory effects.

## Conclusion and Perspectives

*Neisseria gonorrhoeae* is an urgent threat pathogen that is evolving resistance factors faster than therapeutic interventions are being developed. There is currently no preventative vaccine for this STI and infections are not protective against subsequent exposures. Thus, new therapies and prevention strategies are urgently needed against this very common pathogen. While *N. meningitidis* treatment and prevention are considerably more straightforward and well-developed, recent evidence suggests that vaccines to prevent meningococcal infections could also confer some level of protection against gonorrhea. Given that the newer-generation vaccines against *N. meningitidis* contain outer membrane vesicles and highly conserved outer membrane proteins, this cross-protection is perhaps not surprising. This proof-of-principle however, further suggests that deploying conserved outer membrane proteins and transporters as part of an anti-gonococcal vaccine could further protect against meningococcal infections as well. While the importance of expression of the transferrin binding proteins has been demonstrated in a human infection model, none of the other outer membrane transporters have been tested for their impacts on virulence in humans. Animal modeling is limited due to the species restriction of binding only to the human proteins. However, given the clear-cut advantage that the transferrin-iron acquisition system confers on wild-type *N. gonorrhoeae*, it seems likely that these other metal transport systems, which similarly enable the pathogen to overcome nutritional immunity, are equally critical to virulence. Interference with multiple metal uptake systems is likely to reduce the possible redundancy among the systems and hopefully result in greater killing by overpowering the pathogen’s ability to subvert nutritional immunity. While current efforts are largely focused on vaccine development against *N. gonorrhoeae*, the same approach can be directed at identifying targets for newer-generation antibacterial drugs. Targeting one or more outer membrane transport systems that are responsible for importing necessary nutrients into the cell could be an Achilles heel for this stealth pathogen. Such an approach will require detailed structures of both transporter and bound human proteins, the origins of which are described in this review.

## Author Contributions

CC, NN, and TM initiated the collaborative project and acquired funding to support the project. RY and NO contributed the images and participated in writing the manuscript. TM, JS, SM, and CC contributed to writing the manuscript.

## Conflict of Interest

The authors declare that the research was conducted in the absence of any commercial or financial relationships that could be construed as a potential conflict of interest.
