# Supplementary material for: Structural Basis for Evasion of Nutritional Immunity by the Pathogenic Neisseriae
Source: Front Microbiol. 2020 Jan 10;10:2981. doi: 10.3389/fmicb.2019.02981 (PMC6965322; doi:10.3389/fmicb.2019.02981)
Supplement: Supplementary file 1 [file Data_Sheet_1.PDF]

# Supplementary Figure 1. Sequence alignment of TbpAs.

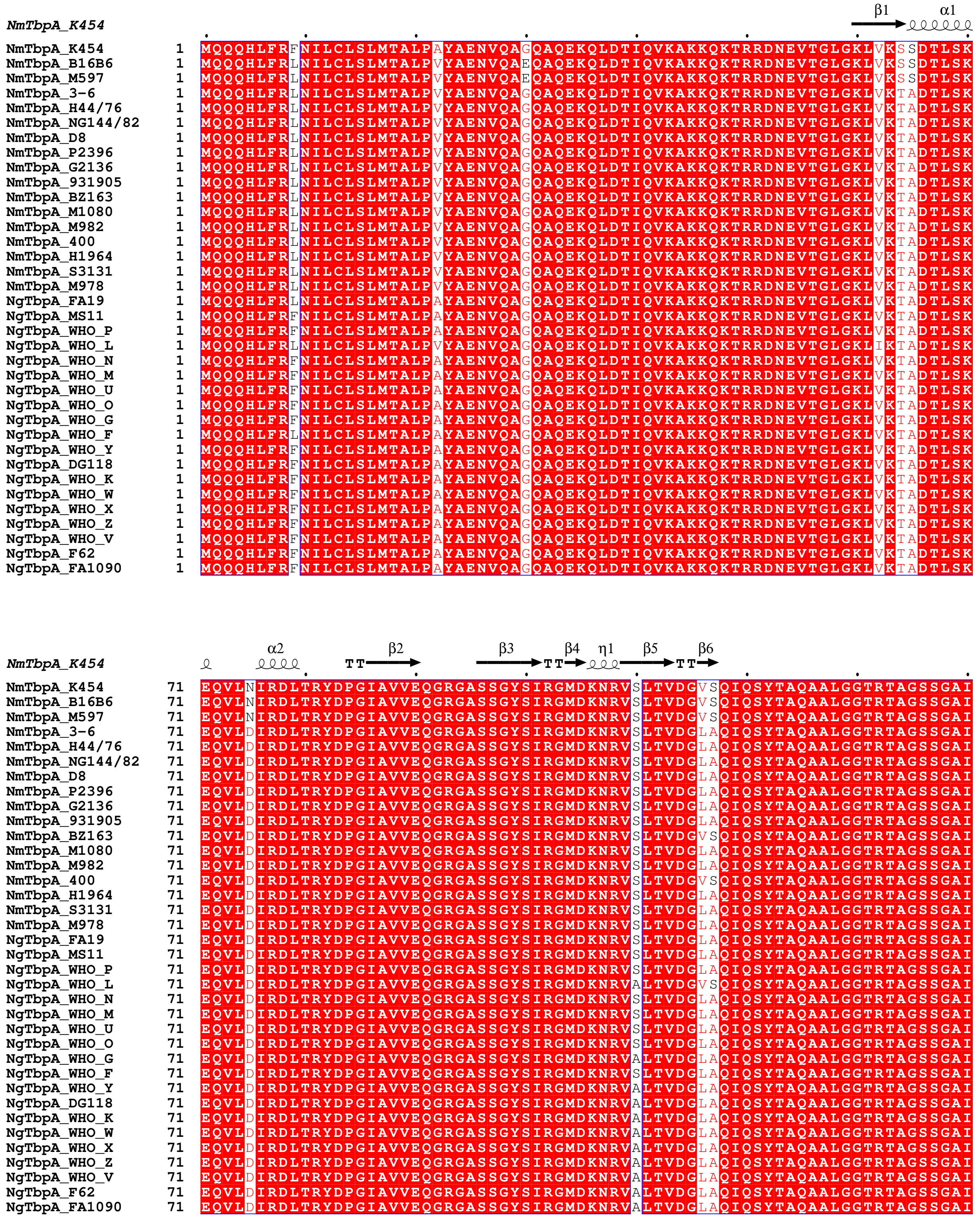

**EIEYE motif**

```
                         η2  β7          α3        β8         η3   TT     β9          η4    β10
NmTbpA_K454

NmTbpA_K454      141  NEIEYENVKAVEISKGSNSVEQGSGALAGSVAFQTKTADDVIGEGRQWGIQSKTAYSGKNRGLTQSIALA
NmTbpA_B16B6     141  NEIEYENVKAVEISKGSNSSEYGNGALAGSVAFQTKTAADIIGEGKQWGIQSKTAYSGKDHALTQSLALA
NmTbpA_M597      141  NEIEYENVKAVEISKGSNSSEYGNGALAGSVAFQTKTAADIIGEGKQWGIQSKTAYSGKDHALTQSLALA
NmTbpA_3-6       141  NEIEYENVKAVEISKGSNSVEQGSGALAGSVAFQTKTADDVIGEGRQWGIQSKTAYSGKNRGLTQSIALA
NmTbpA_H44/76    141  NEIEYENVKAVEISKGSNSVEQGSGALAGSVAFQTKTADDVIGEGRQWGIQSKTAYSGKNRGLTQSIALA
NmTbpA_NG144/82  141  NEIEYENVKAVEISKGSNSVEQGSGALAGSVAFQTKTADDVIGEGRQWGIQSKTAYSGKNRGLTQSIALA
NmTbpA_D8        141  NEIEYENVKAVEISKGSNSVEQGSGALAGSVAFQTKTADDVIGEGRQWGIQSKTAYSGKNRGLTQSIALA
NmTbpA_P2396     141  NEIEYENVKSVEISKGSNSVEQGSGALAGSVAFQTKTADDVIGEGRQWGIQSKTAYSGKNRGLTQSIALA
NmTbpA_G2136     141  NEIEYENVKAVEISKGSNSVEQGSGALAGSVAFQTKTADDVIGEGRQWGIQSKTAYSGKNRGLTLSIALA
NmTbpA_931905    141  NEIEYENVKAVEISKGSNSVEQGSGALAGSVAFQTKTADDVIGEGRQWGIQSKTAYSGKNRGLTQSIALA
NmTbpA_BZ163     141  NEIEYENVKAVEISKGSNSVEQGSGALAGSVAFQTKTADDVIGEGRQWGIQSKTAYSGKNRGLTQSIALA
NmTbpA_M1080     141  NEIEYENVKAVEISKGSNSVEQGSGALAGSVAFQTKTADDVIGEGRQWGIQSKTAYSGKNRGLTQSIALA
NmTbpA_M982      141  NEIEYENVKAVEISKGSNSVEQGSGALAGSVAFQTKTADDVIGEGRQWGIQSKTAYSGKNRGLTQSIALA
NmTbpA_400       141  NEIEYENVKAVEISKGSNSVEQGSGALAGSVAFQTKTADDVIGEGRQWGIQSKTAYSGKNRGLTQSIALA
NmTbpA_H1964     141  NEIEYENVKAVEISKGSNSVEQGSGALAGSVAFQTKTADDVIGEGRQWGIQSKTAYSGKNRGLTQSIALA
NmTbpA_S3131     141  NEIEYENVKAVEISKGSNSVEQGSGALAGSVAFQTKTADDVIGEGRQWGIQSKTAYSGKNRGLTQSIALA
NmTbpA_M978      141  NEIEYENVKAVEISKGSNSVEQGSGALAGSVAFQTKTADDVIGEGRQWGIQSKTAYSGKNRGLTQSIALA
NgTbpA_FA19      141  NEIEYENVKAVEISKGSNSVEQGSGALAGSVAFQTKTADDVIGEGRQWGIQSKTAYSGKNRGLTQSIALA
NgTbpA_MS11      141  NEIEYENVKAVEISKGSNSVEQGSGALAGSVAFQTKTADDVIGEGRQWGIQSKTAYSGKNRGLTQSIALA
NgTbpA_WHO_P     141  NEIEYENVKAVEISKGSNSVEQGSGALAGSVAFQTKTADDVIGEGRQWGIQSKTAYSGKNRGLTQSIALA
NgTbpA_WHO_L     141  NEIEYENVKAVEISKGSNSVEQGSGALAGSVAFQTKTADDVIGEGRQWGIQSKTAYSGKNRGLTQSIALA
NgTbpA_WHO_N     141  NEIEYENVKAVEISKGSNSVEQGSGALAGSVAFQTKTADDVIGEGRQWGIQSKTAYSGKNRGLTQSIALA
NgTbpA_WHO_M     141  NEIEYENVKAVEISKGSNSVEQGSGALAGSVAFQTKTADDVIGEGRQWGIQSKTAYSGKNRGLTQSIALA
NgTbpA_WHO_U     141  NEIEYENVKAVEISKGSNSVEQGSGALAGSVAFQTKTADDVIGEGRQWGIQSKTAYSGKNRGLTQSIALA
NgTbpA_WHO_O     141  NEIEYENVKAVEISKGSNSVEQGSGALAGSVAFQTKTADDVIGEGRQWGIQSKTAYSGKNRGLTQSIALA
NgTbpA_WHO_G     141  NEIEYENVKAVEISKGSNSVEQGSGALAGSVAFQTKTADDVIGEGRQWGIQSKTAYSGKNRGLTQSIALA
NgTbpA_WHO_F     141  NEIEYENVKAVEISKGSNSVEQGSGALAGSVAFQTKTADDVIGEGRQWGIQSKTAYSGKNRGLTQSIALA
NgTbpA_WHO_Y     141  NEIEYENVKAVEISKGSNSVEQGSGALAGSVAFQTKTADDVIGEGRQWGIQSKTAYSGKNRGLTQSIALA
NgTbpA_DG118     141  NEIEYENVKAVEISKGSNSVEQGSGALAGSVAFQTKTADDVIGEGRQWGIQSKTAYSGKNRGLTQSIALA
NgTbpA_WHO_K     141  NEIEYENVKAVEISKGSNSVEQGSGALAGSVAFQTKTADDVIGEGRQWGIQSKTAYSGKNRGLTQSIALA
NgTbpA_WHO_W     141  NEIEYENVKAVEISKGSNSVEQGSGALAGSVAFQTKTADDVIGEGRQWGIQSKTAYSGKNRGLTQSIALA
NgTbpA_WHO_X     141  NEIEYENVKAVEISKGSNSVEQGSGALAGSVAFQTKTADDVIGEGRQWGIQSKTAYSGKNRGLTQSIALA
NgTbpA_WHO_Z     141  NEIEYENVKAVEISKGSNSVEQGSGALAGSVAFQTKTADDVIGEGRQWGIQSKTAYSGKNRGLTQSIALA
NgTbpA_WHO_V     141  NEIEYENVKAVEISKGSNSVEQGSGALAGSVAFQTKTADDVIGEGRQWGIQSKTAYSGKNRGLTQSIALA
NgTbpA_F62       141  NEIEYENVKAVEISKGSNSVEQGSGALAGSVAFQTKTADDVIGEGRQWGIQSKTAYSGKNRGLTQSIALA
NgTbpA_FA1090    141  NEIEYENVKAVEISKGSNSVEQGSGALAGSVAFQTKTADDVIGEGRQWGIQSKTAYSGKNRGLTQSIALA
```

```
                         TT  β11                 TT   β12    β13            TT   β14  η5        α4
NmTbpA_K454

NmTbpA_K454      211  GRIGGAEALLIHTGRRAGEIRAHEDAGRGVQSFNRLVPVE....DSSNYAYFIVKEECKNGSYETCKANP
NmTbpA_B16B6     211  GRSGGAEALLIYTKRRGREIHAHKDAGKGVQSFNRLVLDEDKKEGGSQYRYFIVEEECHNG.YAACKNKL
NmTbpA_M597      211  GRSGGAEALLIYTKRRGREIHAHKDAGKGVQSFNRLVLDEDKKEGGSQYRYFIVEEECHNG.YAACKNKL
NmTbpA_3-6       211  GRIGGAEALLIHTGRRAGEIRAHEDAGRGVQSFNRLVPVE....DTNQYAHFVVEEECPNG.YAACKDKP
NmTbpA_H44/76    211  GRIGGAEALLIHTGRRAGEIRAHEAAGRGVQSFNRLVPVE....DTNQYANFIVEEECESGNYETCKANP
NmTbpA_NG144/82  211  GRIGGAEALLIHTGRRAGEIRAHEDAGRGVQSFNRLVPVE....DSSNYAYFIVKEECKNGSYETCKANP
NmTbpA_D8        211  GRIGGAEALLIHTGRHAGEIRAHEDAGRGVQSFNRLVPVE....DDNQYAHFVVEEECKSG.YETCKANP
NmTbpA_P2396     211  GRIGGAEALLIHTGRRAGEIRAHEAAGRGVQSFNRLVPVE....DDNQYAHFVVEEECKSG.YETCKANP
NmTbpA_G2136     211  GLIVCAEALLIQMGRCAGEIRAHEDAGRGVQSFNRLVPVE....DTNQYANFIVEEECESGSYETCKANP
NmTbpA_931905    211  GRIGGAEALLIHTGRRAGEIRAHEDAGRGVQSFNRLVPVE....DDNQYAHFVVEEECKNGSYETCKSKP
NmTbpA_BZ163     211  GRIGGAEALLIHTGRRAGEIRAHEDAGRGVQSFNRLVPVE....DGSTYAYFVVEEECPNG.YAACKDKP
NmTbpA_M1080     211  GRIGGAEALLIHTGRRAGEIRAHEDAGRGVQSFNRLVPVE....DTHPYANFIVEEECESGNYETCKSKP
NmTbpA_M982      211  GRIGGAEALLIHTGRHAGEIRAHEDAGRGVQSFNRLVPVE....DTHPYANFIVEEECESGNYETCKSKP
NmTbpA_400       211  GRIGGAEALLIHTGRRAGEIRAHEDAGRGVQSFNRLVPVE....DDSKYAYFIVEEECESKNYETCKANP
NmTbpA_H1964     211  GRIGGAEALLIHTGRRAGEIRAHEDAGRGVQSFNRLVPVE....DSSNYAYFIVKEECKNGSYETCKANP
NmTbpA_S3131     211  GRIGGAEALLIHTGRRAGEIRAHEDAGRGVQSFNRLVPVE....DTNQYAHFVVEEECKNRNYEACKANP
NmTbpA_M978      211  GRIGGAEALLIHTSRRAGEIRAHEAAGRGVQSFNRLVPVE....DDHQYAHFVVEEECESRNYETCKANP
NgTbpA_FA19      211  GRIGGAEALLIRTGRHAGEIRAHEAAGRGVQSFNRLAPVD....DGSKYAYFIVEEECKNGGHEKCKANP
NgTbpA_MS11      211  GRIGGAEALLIRTGRHAGEIRAHEAAGRGVQSFNRLAPVD....DASTYAHFIVEAECENGSYETCKTRP
NgTbpA_WHO_P     211  GRIGGAEALLIRTGRHAGEIRAHEAAGRGVQSFNRLAPVD....DASTYAHFIVEAECENGSYETCKTRP
NgTbpA_WHO_L     211  GRIGGAEALLIRTGRHAGEIRAHEAAGRGVQSFNRLAPVD....DASTYAHFIVEAECENGSYETCKTRP
NgTbpA_WHO_N     211  GRIGGAEALLIRTGRHAGEIRAHEAAGRGVQSFNRLAPVD....DGSDYAYFVVEGECPK.GYAACKDKP
NgTbpA_WHO_M     211  GRIGGAEALLIRTGRHAGEIRAHEAAGRGVQSFNRLAPVD....DGSDYAYFVVEGECPK.GYAACKDKP
NgTbpA_WHO_U     211  GRIGGAEALLIRTGRHAGEIRAHEAAGRGVQSFNRLAPVD....DGSDYAYFVVEGECPK.GYAACKDKP
NgTbpA_WHO_O     211  GRIGGAEALLIRTGRHAGEIRAHEAAGRGVQSFNRLAPVD....DGSDYAYFVVEGECPK.GYAACKDKP
NgTbpA_WHO_G     211  GRIGGAEALLIRTGRHAGEIRAHEAAGRGVQSFNRLAPVY....DGSKYAYFIVEEECKNGGYEKCKTRP
NgTbpA_WHO_F     211  GRIGGAEALLIRTGRHAGEIRAHEAAGRGVQSFNRLAPVE....DGSKYAYFIVEEECKNEGYEKCKANP
NgTbpA_WHO_Y     211  GRIGGAEALLIRTGRHAGEIRAHEAAGRGVQSFNRLVPVD....DASTYAYFIVEEECKNGGYEKCKA..
NgTbpA_DG118     211  GRIGGAEALLIRTGRHAGEIRAHEAAGRGVQSFNRLAPVE....DTNQYANFIVEEECKNEGYEKCKANP
NgTbpA_WHO_K     211  GRIGGAEALLIRTGRHAGEIRAHEAAGRGVQSFNRLAPVD....DASTYAYFIVEEECKNGGYEKCKA..
NgTbpA_WHO_W     211  GRIGGAEALLIRTGRHAGEIRAHEAAGRGVQSFNRLAPVD....DASTYAYFIVEEECKNGGYEKCKA..
NgTbpA_WHO_X     211  GRIGGAEALLIRTGRHAGEIRAHEAAGRGVQSFNRLAPVD....DASTYAYFIVEEECKNGGYEKCKA..
NgTbpA_WHO_Z     211  GRIGGAEALLIRTGRHAGEIRAHEAAGRGVQSFNRLAPVD....DASTYAYFIVEEECKNGGYEKCKA..
NgTbpA_WHO_V     211  GRIGGAEALLIRTGRHAGEIRAHEAAGRGVQSFNRLAPVD....DSSDYAHFIVEGECKNGGYETCKTRP
NgTbpA_F62       211  GRIGGAEALLIRTGRHAGEIRAHEAAGRGVQSFNRLAPVD....DASTWVYFIVEEECKNEGYEKCKA..
NgTbpA_FA1090    211  GRIGGAEALLIRTGRHAGEIRAHEAAGRGVQSFNRLVPVD....DASTYAYFIVEEECKNEGYEKCKA..
                                                                          1        1
```

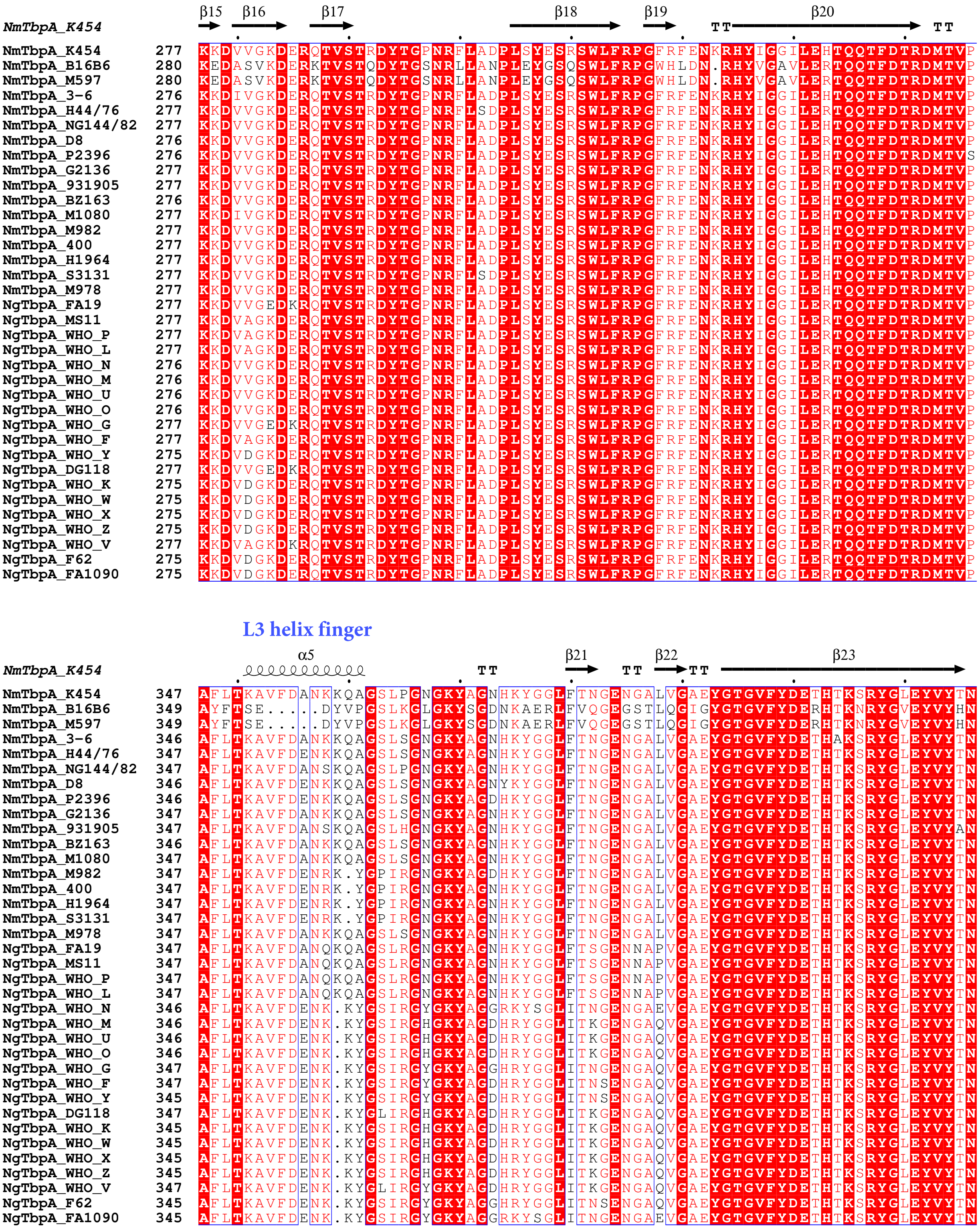
β15 β16 β17 β18 β19 β20
NmTbpA_K454
NmTbpA_K454 277
NmTbpA_B16B6 280
NmTbpA_M597 280
NmTbpA_3-6 276
NmTbpA_H44/76 277
NmTbpA_NG144/82 277
NmTbpA_D8 276
NmTbpA_P2396 276
NmTbpA_G2136 277
NmTbpA_931905 277
NmTbpA_BZ163 276
NmTbpA_M1080 277
NmTbpA_M982 277
NmTbpA_400 277
NmTbpA_H1964 277
NmTbpA_S3131 277
NmTbpA_M978 277
NgTbpA_FA19 277
NgTbpA_MS11 277
NgTbpA_WHO_P 277
NgTbpA_WHO_L 277
NgTbpA_WHO_N 276
NgTbpA_WHO_M 276
NgTbpA_WHO_U 276
NgTbpA_WHO_O 276
NgTbpA_WHO_G 277
NgTbpA_WHO_F 277
NgTbpA_WHO_Y 275
NgTbpA_DG118 277
NgTbpA_WHO_K 275
NgTbpA_WHO_W 275
NgTbpA_WHO_X 275
NgTbpA_WHO_Z 275
NgTbpA_WHO_V 277
NgTbpA_F62 275
NgTbpA_FA1090 275
L3 helix finger
α5 β21 β22 β23
NmTbpA_K454
NmTbpA_K454 347
NmTbpA_B16B6 349
NmTbpA_M597 349
NmTbpA_3-6 346
NmTbpA_H44/76 347
NmTbpA_NG144/82 347
NmTbpA_D8 346
NmTbpA_P2396 346
NmTbpA_G2136 347
NmTbpA_931905 347
NmTbpA_BZ163 346
NmTbpA_M1080 347
NmTbpA_M982 347
NmTbpA_400 347
NmTbpA_H1964 347
NmTbpA_S3131 347
NmTbpA_M978 347
NgTbpA_FA19 347
NgTbpA_MS11 347
NgTbpA_WHO_P 347
NgTbpA_WHO_L 347
NgTbpA_WHO_N 346
NgTbpA_WHO_M 346
NgTbpA_WHO_U 346
NgTbpA_WHO_O 346
NgTbpA_WHO_G 347
NgTbpA_WHO_F 347
NgTbpA_WHO_Y 345
NgTbpA_DG118 347
NgTbpA_WHO_K 345
NgTbpA_WHO_W 345
NgTbpA_WHO_X 345
NgTbpA_WHO_Z 345
NgTbpA_WHO_V 347
NgTbpA_F62 345
NgTbpA_FA1090 345

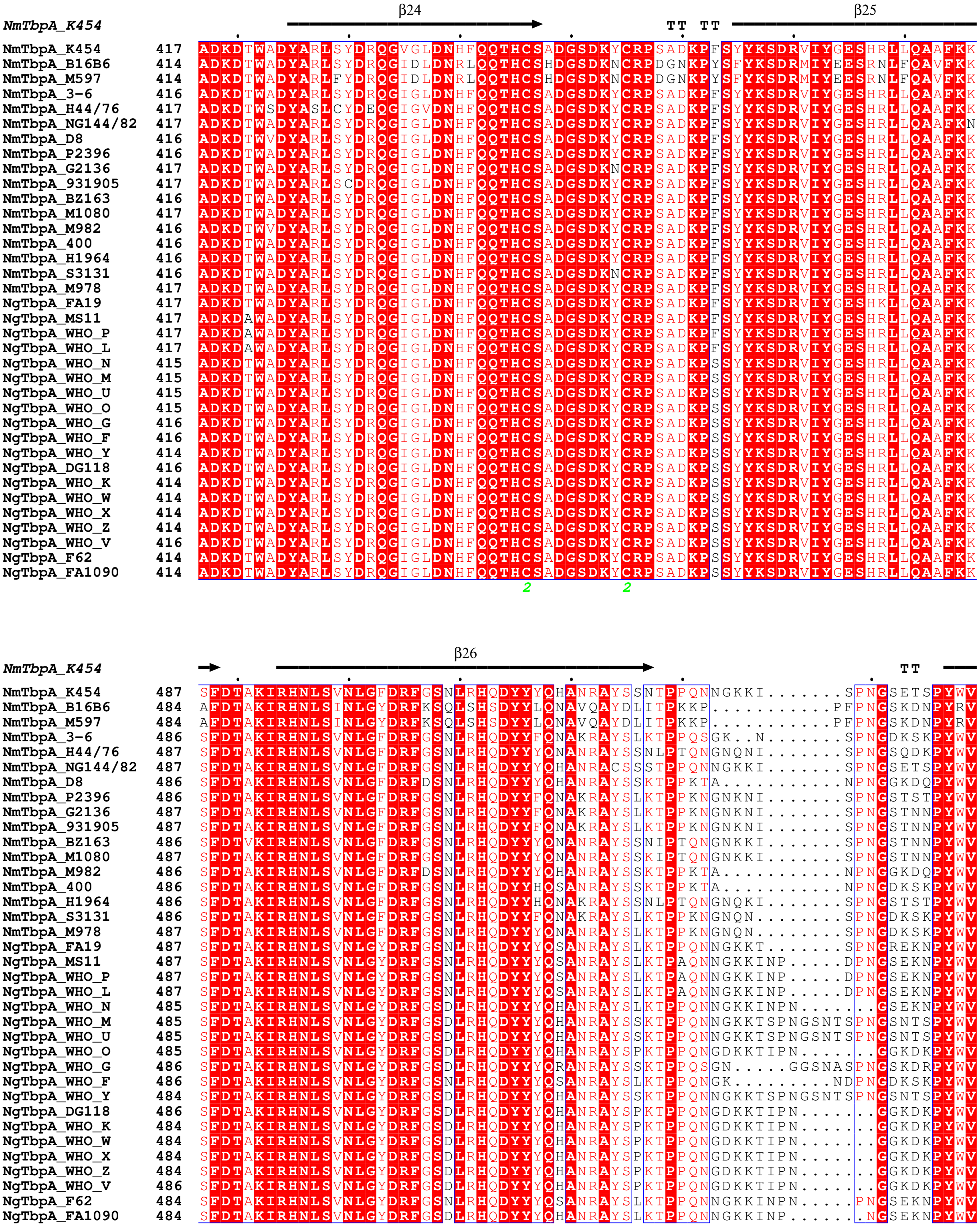

NmTbpA_K454
β24
β25
TT TT
NmTbpA_K454 417
NmTbpA_B16B6 414
NmTbpA_M597 414
NmTbpA_3-6 416
NmTbpA_H44/76 417
NmTbpA_NG144/82 417
NmTbpA_D8 416
NmTbpA_P2396 416
NmTbpA_G2136 417
NmTbpA_931905 417
NmTbpA_BZ163 416
NmTbpA_M1080 417
NmTbpA_M982 416
NmTbpA_400 416
NmTbpA_H1964 416
NmTbpA_S3131 416
NmTbpA_M978 417
NgTbpA_FA19 417
NgTbpA_MS11 417
NgTbpA_WHO_P 417
NgTbpA_WHO_L 417
NgTbpA_WHO_N 415
NgTbpA_WHO_M 415
NgTbpA_WHO_U 415
NgTbpA_WHO_O 415
NgTbpA_WHO_G 416
NgTbpA_WHO_F 416
NgTbpA_WHO_Y 414
NgTbpA_DG118 416
NgTbpA_WHO_K 414
NgTbpA_WHO_W 414
NgTbpA_WHO_X 414
NgTbpA_WHO_Z 414
NgTbpA_WHO_V 416
NgTbpA_F62 414
NgTbpA_FA1090 414
2
2
NmTbpA_K454
β26
TT
NmTbpA_K454 487
NmTbpA_B16B6 484
NmTbpA_M597 484
NmTbpA_3-6 486
NmTbpA_H44/76 487
NmTbpA_NG144/82 487
NmTbpA_D8 486
NmTbpA_P2396 486
NmTbpA_G2136 487
NmTbpA_931905 487
NmTbpA_BZ163 486
NmTbpA_M1080 487
NmTbpA_M982 486
NmTbpA_400 486
NmTbpA_H1964 486
NmTbpA_S3131 486
NmTbpA_M978 487
NgTbpA_FA19 487
NgTbpA_MS11 487
NgTbpA_WHO_P 487
NgTbpA_WHO_L 487
NgTbpA_WHO_N 485
NgTbpA_WHO_M 485
NgTbpA_WHO_U 485
NgTbpA_WHO_O 485
NgTbpA_WHO_G 486
NgTbpA_WHO_F 486
NgTbpA_WHO_Y 484
NgTbpA_DG118 486
NgTbpA_WHO_K 484
NgTbpA_WHO_W 484
NgTbpA_WHO_X 484
NgTbpA_WHO_Z 484
NgTbpA_WHO_V 486
NgTbpA_F62 484
NgTbpA_FA1090 484

```
                           β27                  TT          β28                     β29          TT
NmTbpA_K454
NmTbpA_K454         550  TIGRGNVVTGQICRLGNNTYTDCTPRSINGKSYYAAVRDNVRLGRWADVGAGLRYDYRSTHSDDGSVSTG
NmTbpA_B16B6        543  SIGKTTVNTSPICRFGNNTYTDCTPRNIGGNGYYAAVQDNVRLGRWADVGAGIRYDYRSTHSEDKSVSTG
NmTbpA_M597         543  SIGKTTVNTSPICRFGNNTYTDCTPRNIGGNGYYAAVQDNVRLGRWADVGAGIRYDYRSTHSEDKSVSTG
NmTbpA_3-6          547  SIGGGNVVTGQICLFGNNTYTDCTPRSINGKSYYAAVRDNVRLGRWADVGAGLRYDYRSTHSDDSSVSTG
NmTbpA_H44/76       550  SIGGGNVVTGQICRLGNNTYTDCTPRSINGKSYYAAVRDNVRLGRWADVGAGLRYDYRSTHSDDGSVSTG
NmTbpA_NG144/82     550  TIGRGNVVTGQICRLGNNTYTDCTPRSINGKSYYAAVRDNVRLGRWADVGAGLRYDYRSTHSDDGSVSTG
NmTbpA_D8           545  IICRGNVVTGQICRLGNIIYRDWPPRNFNGKSYYAAIRDNVRLGRWADVGAGLRYDYRSTHSDDGSVSTG
NmTbpA_P2396        549  SIGGGNVVTGQICLFGNNTYTDCTPRSINGKSYYAAVRDNVRLGRWADVGAGLRYDYRSTHSDDGSVSTG
NmTbpA_G2136        550  SIGGGNVVTGQICRLGNNTYTDCTPRSINGKSYYAAVRDNVRLGRWADVGAGLRYDYRSTHSDDGSVSTG
NmTbpA_931905       550  SIGGGNVVTGQICRLGNNTYTDCTPRSINGKSYYAAVRDNVRLGRWADVGAGLRYDYRSTHSDDGSVSTG
NmTbpA_BZ163        549  SIGGGNVVTGQICRLGNNTYTDCTPRSINGKSYYAAVRDNVRLGRWADVGAGLRYDYRSTHSDDGSVSTG
NmTbpA_M1080        550  SIGGGNVVTGQICRLGNNTYTDCTPRSINGKSYYAAVRDNVRLGRWADVGAGLRYDYRSTHSDDGSVSTG
NmTbpA_M982         545  SIGRGNVVTGQICRLGNNTYTDCTPRSINGKSYYAAVRDNVRLGRWADVGAGLRYDYRSTHSDDGSVSTG
NmTbpA_400          545  SIGGGNVVTGQICRLGNNTYTDCTPRSINGKSYYAAVRDNVRLGRWADVGAGLRYDYRSTHSDDGSVSTG
NmTbpA_H1964        549  SIGRGNVVTEQICRLGNNTYTDCTPRSINGKSYYAAVRDNVRLGRWADVGAGLRYDYRSTHSDDSSVSTG
NmTbpA_S3131        548  SIGGGNVVTEQICRLGNNTYTDCTPRSINGKSYYAAVRDNVRLGRWADVGAGLRYDYRSTHSDDGSVSTG
NmTbpA_M978         549  SIGGGNVVTEQICLFGNNTYTDCTPRSINGKSYYAAVRDNVRLGRWADVGAGLRYDYRSTHSDDGSVSTG
NgTbpA_FA19         550  SIGRGNVVTRQICLFGNNTYTDCTPRSINGKSYYAAVRDNVRLGRWADVGAGLRYDYRSTHSDDGSVSTG
NgTbpA_MS11         552  SIGRGNVVTRQICLFGNNTYTDCTPRSINGKSYYAAVRDNVRLGRWADVGAGLRYDYRSTHSDDGSVSTG
NgTbpA_WHO_P        552  SIGRGNVVTRQICLFGNNTYTDCTPRSINGKSYYAAVRDNVRLGRWADVGAGLRYDYRSTHSDDGSVSTG
NgTbpA_WHO_L        552  SIGRGNVVTRQICLFGNNTYTDCTPRSINGKSYYAAVRDNVRLGRWADVGAGLRYDYRSTHSDDGSVSTG
NgTbpA_WHO_N        548  SIGGGNVVTGQICLFGNNTYTDCTPRSINGKSYYAAVRDNVRLGRWADVGAGLRYDYRSTHSDDGSVSTG
NgTbpA_WHO_M        555  SIGGGNVVTGQICLFGNNTYTDCTPRSINGKSYYAAVRDNVRLGRWADVGAGLRYDYRSTHSDDGSVSTG
NgTbpA_WHO_U        555  SIGGGNVVTGQICLFGNNTYTDCTPRSINGKSYYAAVRDNVRLGRWADVGAGLRYDYRSTHSDDGSVSTG
NgTbpA_WHO_O        548  SIGGGNVVTGQICLFGNNTYTDCTPRSINGKSYYAAVRDNVRLGRWADVGAGLRYDYRSTHSDDGSVSTG
NgTbpA_WHO_G        551  SIGGGNVVTGQICLFGNNTYTDCTPRSINGKSYYAAVRDNVRLGRWADVGAGLRYDYRSTHSDDGSVSTG
NgTbpA_WHO_F        547  SIGGGNVVTRQICLFGNNTYTDCTPRSINGKSYYAAVRDNVRLGRWADVGAGLRYDYRSTHSDDGSVSTG
NgTbpA_WHO_Y        554  SIGGGNVVTGQICLFGNNTYTDCTPRSINGKSYYAAVRDNVRLGRWADVGAGLRYDYRSTHSDDGSVSTG
NgTbpA_DG118        549  SIGGGNVVTGQICLFGNNTYTDCTPRSINGKSYYAAVRDNVRLGRWADVGAGLRYDYRSTHSDDGSVSTG
NgTbpA_WHO_K        547  SIGGGNVVTGQICLFGNNTYTDCTPRSINGKSYYAAVRDNVRLGRWADVGAGLRYDYRSTHSDDGSVSTG
NgTbpA_WHO_W        547  SIGGGNVVTGQICLFGNNTYTDCTPRSINGKSYYAAVRDNVRLGRWADVGAGLRYDYRSTHSDDGSVSTG
NgTbpA_WHO_X        547  SIGGGNVVTGQICLFGNNTYTDCTPRSINGKSYYAAVRDNVRLGRWADVGAGLRYDYRSTHSDDGSVSTG
NgTbpA_WHO_Z        547  SIGGGNVVTGQICLFGNNTYTDCTPRSINGKSYYAAVRDNVRLGRWADVGAGLRYDYRSTHSDDGSVSTG
NgTbpA_WHO_V        549  SIGGGNVVTGQICLFGNNTYTDCTPRSINGKSYYAAVRDNVRLGRWADVGAGLRYDYRSTHSDDGSVSTG
NgTbpA_F62          549  SIGGGNVVTGQICLFGNNTYTDCTPRSINGKSYYAAVRDNVRLGRWADVGAGLRYDYRSTHSDDGSVSTG
NgTbpA_FA1090       547  SIGGGNVVTGQICLFGNNTYTDCTPRSINGKSYYAAVRDNVRLGRWADVGAGLRYDYRSTHSDDGSVSTG
                                     3         3

                             β30              β31        α6                   β32        TT
NmTbpA_K454
NmTbpA_K454         620  THRTLSWNAGIVLKPTDWLDLTYRTSTGFRLPSFAEMYGWRAGVQSKAVKIDPEKSFNKEAGIVFKGDFG
NmTbpA_B16B6        613  THRNLSWNAGVVLKPFTWMDLTYRASTGFRLPSFAEMYGWRAGESLKTLDLKPEKSFNREAGIVFKGDFG
NmTbpA_M597         613  THRNLSWNAGVVLKPFTWMDLTYRASTGFRLPSFAEMYGWRAGESLKTLDLKPEKSFNREAGIVFKGDFG
NmTbpA_3-6          617  THRTLSWNAGIVLKPADWLDLTYRTSTGFRLPSFAEMYGWRAGVQSKAVKIDPEKSFNKEAGIVLKAISA
NmTbpA_H44/76       620  THRTLSWNAGVVLKPFTWMDLTYRTSTGFRLPSFSEMYGWRSGDKIKAVKIDPEKSFNKEAGIVFKGDFG
NmTbpA_NG144/82     620  THRTLSWNAGIVLKPTDWLDLTYRTSTGFRLPSFAEMYGWRAGVQSKAVKIDPEKSFNKEAGIVFKGDFG
NmTbpA_D8           615  THRTLSWNAGIVLKPTDWLDLTYRTSTGFRLPSFAEMYGWRAGVQSKAVKIDPEKSFNKEAGIVFKGDFG
NmTbpA_P2396        619  THRTLSWNAGIVLKPADWLDLTYRTSTGFRLPSFAEMYGWRAGDKIKAVKIDPEKSFNKEAGIVFKGDFG
NmTbpA_G2136        620  THRTLSWNAGIVLKPADWLDLTYRTSTGFRLPSFAEMYGWRAGVQSKAVKIDPEKSFNKEAGIVFKGDFG
NmTbpA_931905       620  THRTLSWNAGIVLKPADWLDLTYRTSTGFRLPSFAEMYGWRAGVQSKAVKIDPEKSFNKEAGIVFKGDFG
NmTbpA_BZ163        619  THRTLSWNAGIVLKPADWLDLTYRTSTGFRLPSFAEMYGWRAGVQSKAVKIDPEKSFNKEAGIVFKGDFG
NmTbpA_M1080        620  THRTLSWNAGIVLKPADWLDLTYRTSTGFRLPSFAEMYGWRAGVQSKAVKIDPEKSFNKEAGIVFKGDFG
NmTbpA_M982         615  THRTLSWNAGIVLKPTDWLDLTYRTSTGFRLPSFAEMYGWRAGVQSKAVKIDPEKSFNKEAGIVFKGDFG
NmTbpA_400          615  THRTLSWNAGIVLKPADWLDLTYRTSTGFRLPSFAEMYGWRSGDKIKAVKIDPEKSFNKEAGIVFKGDFG
NmTbpA_H1964        619  THRTLSWNAGIVLKPADWLDLTYRTSTGFRLPSFAEMYGWRAGVQSKAVKIDPEKSFNKEAGIVFKGDFG
NmTbpA_S3131        618  THRTLSWNAGIVLKPADWLDLTYRTSTGFRLPSFAEMYGWRAGVQSKAVKIDPEKSFNKEAGIVFKGDFG
NmTbpA_M978         619  THRTLSWNAGIVLKPADWLDLTYRTSTGFRLPSFAEMYGWRAGVQSKAVKIDPEKSFNKEAGIVFKGDFG
NgTbpA_FA19         620  THRTLSWNAGIVLKPADWLDLTYRTSTGFRLPSFAEMYGWRSGDKIKAVKIDPEKSFNKEAGIVFKGDFG
NgTbpA_MS11         622  THRTLSWNAGIVLKPADWLDLTYRTSTGFRLPSFAEMYGWRSGDKIKAVKIDPEKSFNKEAGIVFKGDFG
NgTbpA_WHO_P        622  THRTLSWNAGIVLKPADWLDLTYRTSTGFRLPSFAEMYGWRSGDKIKAVKIDPEKSFNKEAGIVFKGDFG
NgTbpA_WHO_L        622  THRTLSWNAGIVLKPADWLDLTYRTSTGFRLPSFAEMYGWRSGDKIKAVKIDPEKSFNKEAGIVFKGDFG
NgTbpA_WHO_N        618  THRTLSWNAGIVLKPADWLDLTYRTSTGFRLPSFAEMYGWRSGDKIKAVKIDPEKSFNKEAGIVFKGDFG
NgTbpA_WHO_M        625  THRTLSWNAGIVLKPADWLDLTYRTSTGFRLPSFAEMYGWRSGDKIKAVKIDPEKSFNKEAGIVFKGDFG
NgTbpA_WHO_U        625  THRTLSWNAGIVLKPADWLDLTYRTSTGFRLPSFAEMYGWRSGDKIKAVKIDPEKSFNKEAGIVFKGDFG
NgTbpA_WHO_O        618  THRTLSWNAGIVLKPADWLDLTYRTSTGFRLPSFAEMYGWRSGDKIKAVKIDPEKSFNKEAGIVFKGDFG
NgTbpA_WHO_G        621  THRTLSWNAGIVLKPADWLDLTYRTSTGFRLPSFAEMYGWRSGDKIKAVKIDPEKSFNKEAGIVFKGDFG
NgTbpA_WHO_F        617  THRTLSWNTGIVLKPADWLDLTYRTSTGFRLPSFAEMYGWRSGDKIKAVKIDPEKSFNKEAGIVFKGDFG
NgTbpA_WHO_Y        624  THRTLSWNAGIVLKPADWLDLTYRTSTGFRLPSFAEMYGWRSGDKIKAVKIDPEKSFNKEAGIVFKGDFG
NgTbpA_DG118        619  THRTLSWNTGIVLKPADWLDLTYRTSTGFRLPSFAEMYGWRSGDKIKAVKIDPEKSFNKEAGIVFKGDFG
NgTbpA_WHO_K        617  THRTLSWNTGIVLKPADWLDLTYRTSTGFRLPSFAEMYGWRSGDKIKAVKIDPEKSFNKEAGIVFKGDFG
NgTbpA_WHO_W        617  THRTLSWNTGIVLKPADWLDLTYRTSTGFRLPSFAEMYGWRSGDKIKAVKIDPEKSFNKEAGIVFKGDFG
NgTbpA_WHO_X        617  THRTLSWNTGIVLKPADWLDLTYRTSTGFRLPSFAEMYGWRSGDKIKAVKIDPEKSFNKEAGIVFKGDFG
NgTbpA_WHO_Z        617  THRTLSWNTGIVLKPADWLDLTYRTSTGFRLPSFAEMYGWRSGDKIKAVKIDPEKSFNKEAGIVFKGDFG
NgTbpA_WHO_V        619  THRTLSWNAGIVLKPADWLDLTYRTSTGFRLPSFAEMYGWRSGDKIKAVKIDPEKSFNKEAGIVFKGDFG
NgTbpA_F62          619  THRTLSWNAGIVLKPADWLDLTYRTSTGFRLPSFSEMYGWRSGGKIKAVKIDPEKSFNKEAGIVFKGDFG
NgTbpA_FA1090       617  THRTLSWNTGIVLKPADWLDLTYRTSTGFRLPSFAEMYGWRSGGKIKAVKIDPEKSFNKEAGIVFKGDFG
```

```
                 β33                        β34
NmTbpA_K454
NmTbpA_K454      690 NLEASWFNNAYRDLIVRGYEAQIKDGKEEAKGDPAYLNAQSARITGINILGKIDWNGVWDKLPEGWYSTF
NmTbpA_B16B6     683 NLEASYFNNAYRDLIAFGYETRTQNGQTSASGDPGYRNAQNARIAGINILGKIDWHGVWGGLPDGLYSTL
NmTbpA_M597      683 NLEASYFNNAYRDLIAFGYETRTQNGQTSASGDPGYRNAQNARIAGINILGKIDWHGVWGGLPDGLYSTL
NmTbpA_3-6       687 TRRQGGSTMPSRDLIVRGYEAQIQNGKETAKGDPAYMNAQSARITGINVLGKIDWNGVWDKLPEGWYSTF
NmTbpA_H44/76    690 NLEASWFNNAYRDLIVRGYEAQIKNGKEEAKGDPAYLNAQSARITGINILGKIDWNGVWDKLPEGWYSTF
NmTbpA_NG144/82  690 NLEASWFNNAYRDLIVRGYEAQIKDGKEEAKGDPAYLNAQSARITGINILGKIDWNGVWDKLPEGWYSTF
NmTbpA_D8        685 NLEASWFNNAYRDLIVRGYEAQIQNGKETAKGDPAYLNAQSARITGINVLGKIDWNGVWDKLPEGWYSTF
NmTbpA_P2396     689 NLEASWFNNAYRDLIVRGYEAQIQNGKETAKGDPAYLNAQSARITGINILGKIDWNGVWDKLPEGWYSTF
NmTbpA_G2136     690 NLEASWFNNAYRDLIVRGYEAQIKNGKEEAKGDPAYLNAQSARITGINILGKIDWNGVWDKLPEGWYSTF
NmTbpA_931905    690 NLEASWFNNAYRDLIVRGYEAQIENGKETAKGDPAYLNAQSARITGINILGKIDWNGVWDKLPEGWYSTF
NmTbpA_BZ163     689 NLEASWFNNAYRDLIVRGYEAQIQNGKETAKGDPAYLNAQSARITGINVLGKIDWNGVWDKLPEGWYSTF
NmTbpA_M1080     690 NLEASWFNNAYRDLIVRGYEAQIQNGKETAKGDPAYLNAQSARITGINVLGKIDWNGVWDKLPEGWYSTF
NmTbpA_M982      685 NLEASWFNNAYRDLIVRGYEAQIQNGKETAKGDPAYLNAQSARITGINVLGKIDWNGVWDKLPEGWYSTF
NmTbpA_400       685 NLEASWFNNAYRDLIVRGYEAQIQNGKETAKGDPAYLNAQSARITGINILGKIDWNGVWDKLPEGWYSTF
NmTbpA_H1964     689 NLEASWFNNAYRDLIVRGYEAQIENGKETAKGDPAYLNAQSARITGINILGKIDWNGVWDKLPEGWYSTF
NmTbpA_S3131     688 NLEASWFNNAYRDLIVRGYEAQIQNGKEEAKGDPAYLNAQSARITGINVLGKIDWNGVWDKLPEGWYSTF
NmTbpA_M978      689 NLEASWFNNAYRDLIVRGYEAQIKNGKEEAKGDPAYLNAQSARITGINILGKIDWNGVWDKLPEGWYSTF
NgTbpA_FA19      690 NLEASWFNNAYRDLIVRGYEAQIKDGKEQVKGNPAYLNAQSARITGINILGKIDWNGVWDKLPEGWYSTF
NgTbpA_MS11      692 NLEASWFDNAYRDLIVRGYEAEIKNGKEQTKGDPAYLNAQSARITGINILGKIDWNGVWDKLPEGWYSTF
NgTbpA_WHO_P     692 NLEASWFDNAYRDLIVRGYEVQIKDGKEGVKGDPAYLNAQSARITGINILGKIDWNGVWDKLPEGWYSTF
NgTbpA_WHO_L     692 NLEASWFDNAYRDLIVRGYEAQIKDGKEQVKGDPAYLNAQSARITGINILGKIDWNGVWDKLPEGWYSTF
NgTbpA_WHO_N     688 NLEASWFDNAYRDLIVRGYEAEIKNGKEQAKGAPAYLNAQSARITGINILGKIDWNGVWDKLPEGWYSTF
NgTbpA_WHO_M     695 NLEASWFNNAYRDLIVRGYEVQIKDGKEQVKGDPAYLNAQSARITGINILGKIDWNGVWDKLPEGWYSTF
NgTbpA_WHO_U     695 NLEASWFNNAYRDLIVRGYEVQIKDGKEQVKGDPAYLNAQSARITGINILGKIDWNGVWDKLPEGWYSTF
NgTbpA_WHO_O     688 NLEASWFNNAYRDLIVRGYEVQIKDGKEGVKGDPAYLNAQSARITGINILGKIDWNGVWDKLPEGWYSTF
NgTbpA_WHO_G     691 NLEASWFDNAYRDLIVRGYEVQIQDGKEQAKGDPAYLNAQSARITGINILGKIDWNGVWDKLPEGWYSTF
NgTbpA_WHO_F     687 NLEASWFDNAYRDLIVRGYEAQIKDGKEGVKGDPAYLNAQSARITGINILGKIDWNGVWDKLPEGWYSTF
NgTbpA_WHO_Y     694 NLEASWFDNAYRDLIVRGYEVQIKDGKEQVKGDPAYLNAQSARITGINILGKIDWNGVWDKLPEGWYSTF
NgTbpA_DG118     689 NLEASWFDNAYRDLIVRGYEAQIKDGKEGVKGDPAYLNAQSARITGINVLGKIDWNGVWDKLPEGWYSTF
NgTbpA_WHO_K     687 NLEASWFDNAYRDLIVRGYEVQIKDGKEGVKGDPAYLNAQSARITGINILGKIDWNGVWDKLPEGWYSTF
NgTbpA_WHO_W     687 NLEASWFDNAYRDLIVRGYEVQIKDGKEGVKGDPAYLNAQSARITGINILGKIDWNGVWDKLPEGWYSTF
NgTbpA_WHO_X     687 NLEASWFDNAYRDLIVRGYEVQIKDGKEGVKGDPAYLNAQSARITGINILGKIDWNGVWDKLPEGWYSTF
NgTbpA_WHO_Z     687 NLEASWFDNAYRDLIVRGYEVQIKDGKEGVKGDPAYLNAQSARITGINILGKIDWNGVWDKLPEGWYSTF
NgTbpA_WHO_V     689 NLEASWFNNAYRDLIVRGYEVQIKDGKEGVKGDPAYLNAQSARITGINILGKIDWNGVWDKLPEGWYSTF
NgTbpA_F62       689 NLEASWFDNAYRDLIVRGYEAEIKNGKEQTKGDPAYLNAQSARITGINILGKIDWNGVWDKLPEGWYSTF
NgTbpA_FA1090    687 NLEASWFNNAYRDLIVRGYEAQIKDGKEQVKGDPAYLNAQSARITGINILGKIDWNGVWDKLPEGWYSTF

                 β35         β36 β37           β38          β39          η6 β40
NmTbpA_K454                TT           TT               TT
NmTbpA_K454      760 AYNRVRVRDIKKRADRTDIQSHLFDAIQPSRYVVGLGYDQPEGKWGVNGMLTYSKAKEITELLGSRALLN
NmTbpA_B16B6     753 AYNRIKVKDADIRADRTFVTSYLFDAVQPSRYVLGLGYDHPDGIWGINTMFTYSKAKSVDELLGSQALLN
NmTbpA_M597      753 AYNRIKVKDADIRADRTFVTSYLFDAVQPSRYVLGLGYDHPDGIWGINTMFTYSKAKSVDELLGSQALLN
NmTbpA_3-6       757 AYNRVHVRDIKKRADRADIQSHLFDAIQPSRYVVGLGYDQPEGKWGVNGMLTYSKAKEITELLGSRALLN
NmTbpA_H44/76    760 AYNRVRVRDIKKRADRTDIQSHLFDAIQPSRYVVGLGYDQPEGKWGVNGMLTYSKAKEITELLGSRALLN
NmTbpA_NG144/82  760 AYNRVRVRDIKKRADRTDIQSHLFDAIQPSRYVVGLGYDQPEGKWGVNGMLTYSKAKEITELLGSRALLN
NmTbpA_D8        755 AYNRVHVRDIKKRADRTDIQSHLFDAIQPSRYVVGLGYDQPEGKWGVNGMLTYSKAKEITELLGSRALLN
NmTbpA_P2396     759 AYNRVRVRDIKKRADRTDIQSHLFDAIQPSRYVVGLGYDQPEGKWGVNGMLTYSKAKEITELLGSRALLN
NmTbpA_G2136     760 AYNRVRVRDIKKRADRTDIQSHLFDAIQPSRYVVGLGYDQPEGKWGVNGMLTYSKAKEITELLGSRALLN
NmTbpA_931905    760 AYNRVHVRDIKKRADRTDIQSHLFDAIQPSRYVVGLGYDQPEGKWGVNGMLTYSKAKEITELLGSRALLN
NmTbpA_BZ163     759 AYNRVHVRDIKKRADRTDIQSHLFDAIQPSRYVVGLGYDQPEGKWGVNGMLTYSKAKEITELLGSRALLN
NmTbpA_M1080     760 AYNRVRVRDIKKRADRTDIQSHLFDAIQPSRYVVGLGYDQPEGKWGVNGMLTYSKAKEITELLGSRALLN
NmTbpA_M982      755 AYNRVHVRDIKKRADRTDIQSHLFDAIQPSRYVVGLGYDQPEGKWGVNGMLTYSKAKEITELLGSRALLN
NmTbpA_400       755 AYNRVHVRDIKKRADRTDIQSHLFDAIQPSRYVVGLGYDQPEGKWGVNGMLTYSKAKEITELLGSRALLN
NmTbpA_H1964     759 AYNRVRVRDIKKRADRTDIQSHLFDAIQPSRYVVGLGYDQPEGKWGVNGMLTYSKAKEITELLGSRALLN
NmTbpA_S3131     758 AYNRVHVRDIKKRADRTDIQSHLFDAIQPSRYVVGLGYDQPEGKWGVNGMLTYSKAKEITELLGSRALLN
NmTbpA_M978      759 AYNRVRVRDIKKRADRTDIQSHLFDAIQPSRYVIGLGYDQPEGKWGVNGMLTYSKAKEITELLGSRALLN
NgTbpA_FA19      760 AYNRVRVRDIKKRADRTDIQSHLFDAIQPSRYVVGSGYDQPEGKWGVNGMLTYSKAKEITELLGSRALLN
NgTbpA_MS11      762 AYNRVRVRDIKKRADRTGIQSHLFDAIQPSRYVVGSGYDQPEGKWGVNGMLTYSKAKEITELLGSRALLN
NgTbpA_WHO_P     762 AYNRVRVRDIKKRADRTDIQSHLFDAIQPSRYVVGSGYDQPEGKWGVNGMLTYSKAKEITELLGSRALLN
NgTbpA_WHO_L     762 AYNRVRVRDIKKRADRTDIQSHLFDAIQPSRYVVGSGYDQPEGKWGVNGMLTYSKAKEITELLGSRALLN
NgTbpA_WHO_N     758 AYNRVRVRDIKKRADRTDIQSHLFDAIQPSRYVVGSGYDQPEGKWGVNGMLTYSKAKEITELLGSRALLN
NgTbpA_WHO_M     765 AYNRVRVRDIKKRADRTDIQSHLFDAIQPSRYVVGSGYDQPEGKWGVNGMLTYSKAKEITELLGSRALLN
NgTbpA_WHO_U     765 AYNRVRVRDIKKRADRTDIQSHLFDAIQPSRYVVGSGYDQPEGKWGVNGMLTYSKAKEITELLGSRALLN
NgTbpA_WHO_O     758 AYNRVRVRDIKKRADRTDIQSHLFDAIQPSRYVVGSGYDQPEGKWGVNGMLTYSKAKEITELLGSRALLN
NgTbpA_WHO_G     761 AYNRVRVRDIKKRADRTDIQSHLFDAIQPSRYVVGSGYDQPEGKWGVNGMLTYSKAKEITELLGSRALLN
NgTbpA_WHO_F     757 AYNRVRVRDIKKRADRTDIQSHLFDAIQPSRYVVGSGYDQPEGKWGVNGMLTYSKAKEITELLGSRALLN
NgTbpA_WHO_Y     764 AYNRVRVRDIKKRADRTDIQSHLFDAIQPSRYVVGSGYDQPEGKWGVNGMLTYSKAKEITELLGSRALLN
NgTbpA_DG118     759 AYNRVRVRDIKKRADRTDIQSHLFDAIQPSRYVVGSGYDQPEGKWGVNGMLTYSKAKEITELLGSRALLN
NgTbpA_WHO_K     757 AYNRVRVRDIKKRADRTDIQSHLFDAIQPSRYVVGSGYDQPEGKWGVNGMLTYSKAKEITELLGSRALLN
NgTbpA_WHO_W     757 AYNRVRVRDIKKRADRTDIQSHLFDAIQPSRYVVGSGYDQPEGKWGVNGMLTYSKAKEITELLGSRALLN
NgTbpA_WHO_X     757 AYNRVRVRDIKKRADRTDIQSHLFDAIQPSRYVVGSGYDQPEGKWGVNGMLTYSKAKEITELLGSRALLN
NgTbpA_WHO_Z     757 AYNRVRVRDIKKRADRTDIQSHLFDAIQPSRYVVGSGYDQPEGKWGVNGMLTYSKAKEITELLGSRALLN
NgTbpA_WHO_V     759 AYNRVRVRDIKKRADRTDIQSHLFDAIQPSRYVVGSGYDQPEGKWGVNGMLTYSKAKEITELLGSRALLN
NgTbpA_F62       759 AYNRVRVRDIKKRADRTDIQSHLFDAIQPSRYVVGSGYDQPEGKWGVNGMLTYSKAKEITELLGSRALLN
NgTbpA_FA1090    757 AYNRVRVRDIKKRADRTDIQSHLFDAIQPSRYVVGSGYDQPEGKWGVNGMLTYSKAKEITELLGSRALLN
```

```
                      β41        β42           TT   β43           α7  η7     TT TT      η8
NmTbpA_K454

NmTbpA_K454     830 GNSRNTKATARRTRPWYIVDVSGYYTVKKHFTLRAGVYNLLNYRYVTWENVRQTAGGAVNQHKNVGVYNR
NmTbpA_B16B6    823 GNANAKKAASRRTRPWYVTDVSGYYNIKKHLTLRAGVYNLLNYRYVTWENVRQTAGGAVNQHKNVGVYNR
NmTbpA_M597     823 GNANAKKAASRRTRPWYVTDVSGYYNIKKHLTLRAGVYNLLNYRYVTWENVRQTAGGAVNQHKNVGVYNR
NmTbpA_3-6      827 GNSRNTKATARRTRPWYIVDVSGYYTIKKHFTLRAGVYNLLNHRYVTWENVRQTAGGAVNQHKNVGVYNR
NmTbpA_H44/76   830 GNSRNTKATARRTRPWYIVDVSGYYTIKKHFTLRAGVYNLLNHRYVTWENVRQTAGGAVNQHKNVGVYNR
NmTbpA_NG144/82 830 GNSRNTKATARRTRPWYIVDVSGYYTVKKHFTLRAGVYNLLNYRYVTWENVRQTAGGAVNQHKNVGVYNR
NmTbpA_D8       825 GNSRNTKATARRTRPWYIVDVSGYYTIKKHFTLRAGVYNLLNYRYVTWENVRQTAGGAVNQHKNVGVYNR
NmTbpA_P2396    829 GNSRNTKATARRTRPWYIVDVSGYYTIKKHFTLRAGVYNLLNYRYVTWENVRQTAGGAVNQHKNVGVYNR
NmTbpA_G2136    830 GNSRNTKATARRTRPWYIVDVSGYYTIKKHFTLRAGVYNLLNHRYVTWENVRQTAGGAVNQHKNVGVYNR
NmTbpA_931905   830 GNSRNTKATARRTRPWYIVDVSGYYTIKKHFTLRAGVYNLLNYRYVTWENVRQTAGGAVNQHKNVGVYNR
NmTbpA_BZ163    829 GNSRNTKATARRTRPWYIVDVSGYYTIKKHFTLRAGVYNLLNYRYVTWENVRQTAGGAVNQHKNVGVYNR
NmTbpA_M1080    830 GNSRNTKATARRTRPWYIVDVSGYYTIKKHFTLRAGVYNLLNYRYVTWENVRQTAGGAVNQHKNVGVYNR
NmTbpA_M982     825 GNSRNTKATARRTRPWYIVDVSGYYTIKKHFTLRAGVYNLLNYRYVTWENVRQTAGGAVNQHKNVGVYNR
NmTbpA_400      825 GNSRNTKATARRTRPWYIVDVSGYYTIKKHFTLRAGVYNLLNYRYVTWENVRQTAGGAVNQHKNVGVYNR
NmTbpA_H1964    829 GNSRNTKATARRTRPWYIVDVSGYYTIKKHFTLRAGVYNLLNYRYVTWENVRQTAGGAVNQHKNVGVYNR
NmTbpA_S3131    828 GNSRNTKATARRTRPWYIVDVSGYYTIKKHFTLRAGVYNLLNYRYVTWENVRQTAGGAVNQHKNVGVYNR
NmTbpA_M978     829 GNSRNTKATARRTRPWYIVDVSGYYTIKKHFTLRAGVYNLLNHRYVTWENVRQTAGGAVNQHKNVGVYNR
NgTbpA_FA19     830 GNSRNTKATARRTRPWYIVDVSGYYTVKKHFTLRAGVYNLLNHRYVTWENVRQTAAGAVNQHKNVGVYNR
NgTbpA_MS11     832 GNSRDTKATARRTRPWYIVDVSGYYTVKKHFTLRAGVYNLLNHRYVTWENVRQTAAGAVNQHKNVGVYNR
NgTbpA_WHO_P    832 GNSRNTKATARRTRPWYIVDVSGYYTVKKHFTLRAGVYNLLNHRYVTWENVRQTAGGAVNQHKNVGVYNR
NgTbpA_WHO_L    832 GNSRDTKATARRTRPWYIVDVSGYYTVKKHFTLRAGVYNLLNHRYVTWENVRQTAAGAVNQHKNVGVYNR
NgTbpA_WHO_N    828 GNSRNTKATARRTRPWYIVDVSGYYTVKKHFTLRAGVYNLLNHRYVTWENVRQTAAGAVNQHKNVGVYNR
NgTbpA_WHO_M    835 GNSRDTKATARRTRPWYIVDVSGYYTVKKHFTLRAGVYNLLNHRYVTWENVRQTAAGAVNQHKNVGVYNR
NgTbpA_WHO_U    835 GNSRDTKATARRTRPWYIVDVSGYYTVKKHFTLRAGVYNLLNHRYVTWENVRQTAAGAVNQHKNVGVYNR
NgTbpA_WHO_O    828 GNSRDTKATARRTRPWYIVDVSGYYTVKKHFTLRAGVYNLLNHRYVTWENVRQTAAGAVNQHKNVGVYNR
NgTbpA_WHO_G    831 GNSRNTKATARRTRPWYIVDVSGYYTVKKHFTLRAGVYNLLNHRYVTWENVRQTAGGAVNQHKNVGVYNR
NgTbpA_WHO_F    827 GNSRDTKATARRTRPWYIVDVSGYYTVKKHFTLRAGVYNLLNHRYVTWENVRQTAAGAVNQHKNVGVYNR
NgTbpA_WHO_Y    834 GNSRNTKATARRTRPWYIVDVSGYYTVKKHFTLRAGVYNLLNHRYVTWENVRQTAAGAVNQHKNVGVYNR
NgTbpA_DG118    829 GNSRNTKATARRTRPWYIVDVSGYYTVKKHFTLRAGVYNLLNHRYVTWENVRQTAAGAVNQHKNVGVYNR
NgTbpA_WHO_K    827 GNSRDTKATARRTRPWYIVDVSGYYTVKKHFTLRAGVYNLLNHRYVTWENVRQTAAGAVNQHKNVGVYNR
NgTbpA_WHO_W    827 GNSRDTKATARRTRPWYIVDVSGYYTVKKHFTLRAGVYNLLNHRYVTWENVRQTAAGAVNQHKNVGVYNR
NgTbpA_WHO_X    827 GNSRDTKATARRTRPWYIVDVSGYYTVKKHFTLRAGVYNLLNHRYVTWENVRQTAAGAVNQHKNVGVYNR
NgTbpA_WHO_Z    827 GNSRDTKATARRTRPWYIVDVSGYYTVKKHFTLRAGVYNLLNHRYVTWENVRQTAAGAVNQHKNVGVYNR
NgTbpA_WHO_V    829 GNSRDTKATARRTRPWYIVDVSGYYTVKKHFTLRAGVYNLLNHRYVTWENVRQTAAGAVNQHKNVGVYNR
NgTbpA_F62      829 GNSRNTKATARRTRPWYIVDVSGYYTVKKHFTLRAGVYNLLNHRYVTWENVRQTAAGAVNQHKNVGVYNR
NgTbpA_FA1090   827 GNSRDTKATARRTRPWYIVDVSGYYTVKKHFTLRAGVYNLLNHRYVTWENVRQTAAGAVNQHKNVGVYNR
```

```
                       β44
NmTbpA_K454

NmTbpA_K454     900 YAAPGRNYTFSLEMKF...
NmTbpA_B16B6    893 YAAPGRNYTFSLEMKF...
NmTbpA_M597     893 YAAPGRNYTFSLEMKF...
NmTbpA_3-6      897 YAAPGRNYTFSLEMKF...
NmTbpA_H44/76   900 YAAPGRNYTFSLEMKFNGO
NmTbpA_NG144/82 900 YAAPGRNYTFSLEMKF...
NmTbpA_D8       895 YAAPGRNYTFSLEMKF...
NmTbpA_P2396    899 YAAPGRNYTFSLEMKF...
NmTbpA_G2136    900 YAAPGRNYTFSLEMKF...
NmTbpA_931905   900 YAAPGRNYTFSLEMKF...
NmTbpA_BZ163    899 YAAPGRNYTFSLEMKF...
NmTbpA_M1080    900 YAAPGRNYTFSLEMKF...
NmTbpA_M982     895 YAAPGRNYTFSLEMKF...
NmTbpA_400      895 YAAPGRNYTFSLEMKF...
NmTbpA_H1964    899 YAAPGRNYTFSLEMKF...
NmTbpA_S3131    898 YAAPGRNYTFSLEMKF...
NmTbpA_M978     899 YAAPGRNYTFSLEMKF...
NgTbpA_FA19     900 YAAPGRNYTFSLEMKF...
NgTbpA_MS11     902 YAAPGRNYTFSLEMKF...
NgTbpA_WHO_P    902 YAAPGRNYTFSLEMKF...
NgTbpA_WHO_L    902 YAAPGRNYTFSLEMKF...
NgTbpA_WHO_N    898 YAAPGRNYTFSLEMKF...
NgTbpA_WHO_M    905 YAAPGRNYTFSLEMKF...
NgTbpA_WHO_U    905 YAAPGRNYTFSLEMKF...
NgTbpA_WHO_O    898 YAAPGRNYTFSLEMKF...
NgTbpA_WHO_G    901 YAAPGRNYTFSLEMKF...
NgTbpA_WHO_F    897 YAAPGRNYTFSLEMKF...
NgTbpA_WHO_Y    904 YAAPGRNYTFSLEMKF...
NgTbpA_DG118    899 YAAPGRNYTFSLEMKF...
NgTbpA_WHO_K    897 YAAPGRNYTFSLEMKF...
NgTbpA_WHO_W    897 YAAPGRNYTFSLEMKF...
NgTbpA_WHO_X    897 YAAPGRNYTFSLEMKF...
NgTbpA_WHO_Z    897 YAAPGRNYTFSLEMKF...
NgTbpA_WHO_V    899 YAAPGRNYTFSLEMKF...
NgTbpA_F62      899 YAAPGRNYTFSLEMKF...
NgTbpA_FA1090   897 YAAPGRNYTFSLEMKF...
```

**Supplementary Figure 2. Sequence alignment of TbpBs.**

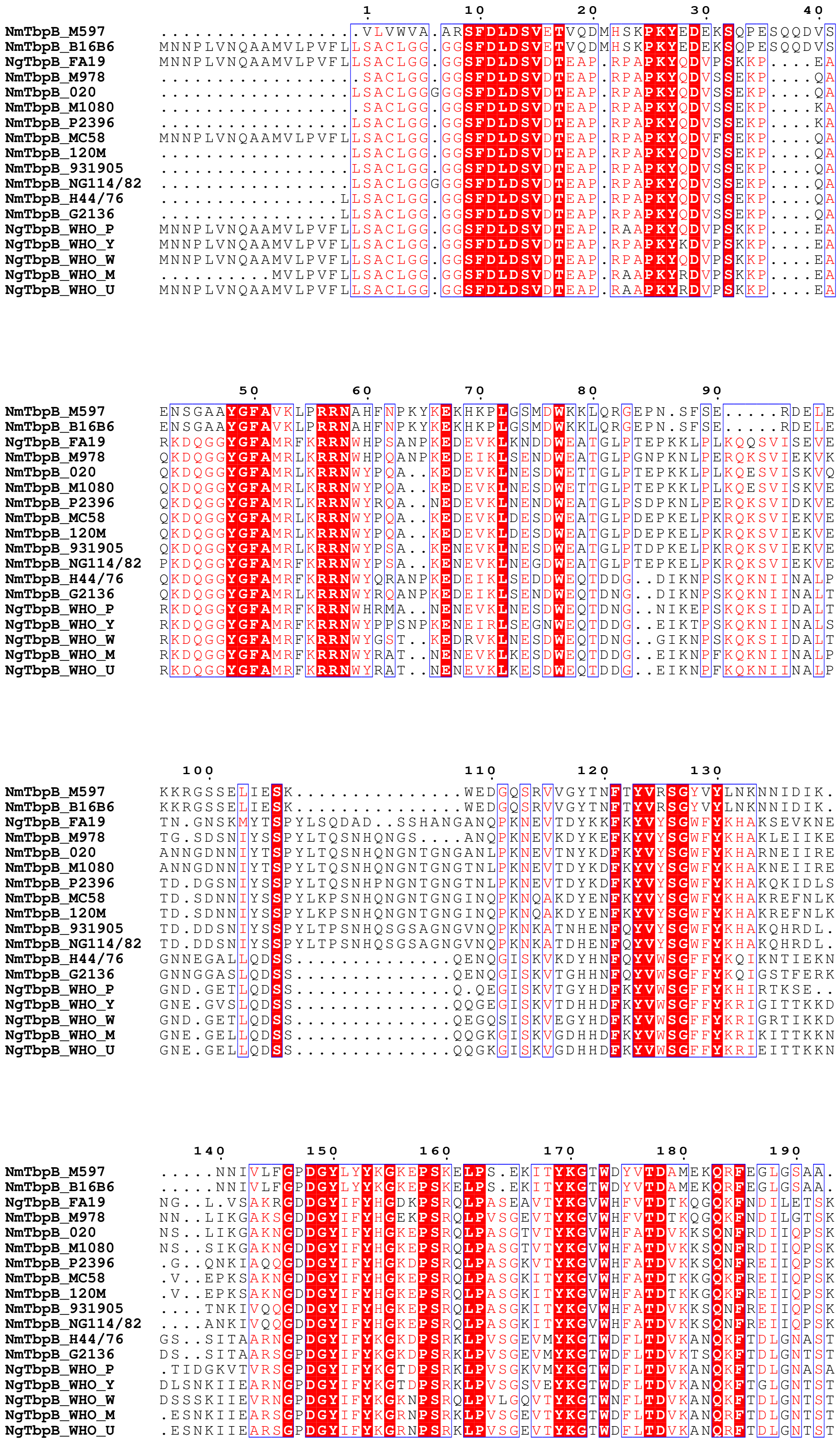

```
                        200       210       220       230       240
NmTbpB_M597         .GGDKSGALSALE..EGVLRNQAEASSGHTDFGMTSEFEVDFSDKTIKGTLYRNNRITQN
NmTbpB_B16B6        .GGDKSGALSALE..EGVLRNQAEASSGHTDFGMTSEFEVDFSDKTIKGTLYRNNRITQN
NgTbpB_FA19         GQGDKYSGFSGDEGETTSNRTDSNLNDKHEGYGFTSNFKVDFNNKKLTGKLIRNNKVINT
NmTbpB_M978         KQGDRYSGFPGDDGEEYSNKNEATLQGSQEGYGFTSNLKVDFNKKKLTGELIRNNRVTNA
NmTbpB_020          KQGDRYSGFSGDDDEQYSNKNESMLKDGQEGYGFTSNLEVDFNNKKLTGKLIRNDKVTNA
NmTbpB_M1080        KQGDRYSGFSGDDDEQYSNKNESMLKDGQEGYGFTSNLEVDFDNKKLTGKLIRNDKVTNA
NmTbpB_P2396        RQGDKYSGFSGDEGEEYSNKNEKTLQSGHEGYGFTSNLEVDFGNKKLTGKLIRNNANQNN
NmTbpB_MC58         SQGDRYSGFSGDDGEEYSNKNKSTLTDGQEGYGFTSNLEVDFHNKKLTGKLIRNNANTD.
NmTbpB_120M         SQGDRYSGFSGDDGEEYSNKNKSTLTDGQEGYGFTSNLEVDFHNKKLTGKLIRNNANTD.
NmTbpB_931905       SQGDRYSGFSGDDGEEYSNKNETTLQSGHEGYGFTSNLEVDFDNKKLTGKLIRNNANTS.
NmTbpB_NG114/82     SQGDRYSGFSGDDGEEYSNKNETTLQSGHEGYGFTSNLEVDFDNKKLTGKLIRNNANTS.
NmTbpB_H44/76       KPGDRYSAFSGEL..DYIVKQENDKKDGHVGLGLTTEITVDFEKKTLSGKLIKNNSVITT
NmTbpB_G2136        GPGDRYSAFSGEL..DYIVNKDSDKKDEHVGWGLTTEITVDFEKKTLSGKLIKNNRV.NN
NgTbpB_WHO_P        KPGDRYSAFSGEL..DYIVKKEDDKKDGHVGLGLTTEITVDFGKKTLSGKLIKNNMVINN
NgTbpB_WHO_Y        KSGDRYSAFSGEL..DYIVKKESDKKDGHVGLGLTTEITVDFGKKTLSGKLIKNNMVINN
NgTbpB_WHO_W        KPGDRYSAFPGEL..DYIVKKEDDKKEKHKGLGLTTEITVDFENKTLKGKLVKNNHVIND
NgTbpB_WHO_M        KSGDQYSAFSGEL..DYIVKKEEDKKEKHKGLGLTTEITVDFGKKILNGKLIKNNYVINN
NgTbpB_WHO_U        KSGDQYSAFSGEL..DYIVKKEEDKKEKHKGLGLTTEITVDFGKKILNGKLIKNNYVINN
```

```
                   250        260       270       280            290       300
NmTbpB_M597         NSENKQ.IKTTRYTIQATLHGNRFKGKALAADKGA......TNGSHPFISDSDSLEGGFY
NmTbpB_B16B6        NSENKQ.IKTTRYTIQATLHGNRFKGKALAADKGA......TNGSHPFISDSDSLEGGFY
NgTbpB_FA19         AAS..DGYTTEYYSLDATLRGNRFSGKAIATDKPN.T...GGTKLHPFVFDSSSLSGGFF
NmTbpB_M978         TAN..DKYTTQYYSLEAQVTGNRFNGKATATDKPG.T...GETKQHPFVSDSSSLSGGFF
NmTbpB_020          TTG..GKHATQYYSLEAQVTGNRFNGKATATDKPQ.N...SETKEHPFVSDSSSLSGGFF
NmTbpB_M1080        TTS..DKHTTQYYSLEAQVTGNRFNGKATATDKPG.N...GETKQHPFVSDSSSLSGGFF
NmTbpB_P2396        TNN..DKHTTQYYSLDATLKGNRFSGKAEATDKPKND...GETKEHPFVSDSSSLSGGFF
NmTbpB_MC58         .NN..QATTTQYYSLEAQVTGNRFNGKATATDKPQQN...SETKEHPFVSDSSSLSGGFF
NmTbpB_120M         .NN..QATTTQYYSLEAQVTGNRFNGKATATDKPQ.N...SETKEHPFVSDSSSLSGGFF
NmTbpB_931905       .NN..QA.TTQYYSLEAQVTGNRFNGKATATDKPQ.N...SETKEHPFVSDSSSLSGGFF
NmTbpB_NG114/82     .NN..QA.TTQYYSLDAQITGNRFNGKAMATDKPG.T...GETKLHPFVSDSSSLSGGFF
NmTbpB_H44/76       NND...KHTTQYYSLEATLKGNRFNGKATATDKPKE....NETKQHPFVSDSSSLSGGFF
NmTbpB_G2136        DND...KHTTQYYSLDATLKGNRFSGKAEATDKPKND...GETKEHPFVSDSSSLSGGFF
NgTbpB_WHO_P        GDE....PTTQYYSLEAQVTGNRFNGKAIATDKPKAN....ETKEHPFVSDSSSLSGGFF
NgTbpB_WHO_Y        GDE....PTTQYYSLEAQVTGNRFNGKAIATDKPKVN....ETKEHPFVSDSSSLSGGFF
NgTbpB_WHO_W        NSDDQNKYTTEYYTLDATLRGNRFSGKAMATDKSKNGETGDQTKKHPFVSDSFSLSGGFF
NgTbpB_WHO_M        NGDGQNKYTTEYYTLDATLRGNRFSGKATATDKKEN.....ETGQHPFVSDSSSLSGGFF
NgTbpB_WHO_U        NGDGQNKYTTEYYTLDATLRGNRFSGKATATDKKEN.....ETGQHPFVSDSSSLSGGFF
```

```
                        310       320       330                           340
NmTbpB_M597         GPKGEELAGKFLSNDNKVAAVFGAKQKDKKDGENAA.....................GPAT
NmTbpB_B16B6        GPKGEELAGKFLSNDNKVAAVFGAKQKDKKDGENAA.....................GPAT
NgTbpB_FA19         GPQGEELGFRFLSDDGKVAVVGSAKTKDSTANGNAPAASSGPGAA.......TM.PSETRL
NmTbpB_M978         GPKGEELGFRFLSNDQKVAVVGSAKTQDKAANGNTAAASGGTDAAASNGAAGTSSENSKL
NmTbpB_020          GPKGEELGFRFLSNDKKVAVVGSAKTQDKPGN..GAAASDGAGAAASNGAAAMPSENGKL
NmTbpB_M1080        GPKGEELGFRFLSDDKKVAVVGSAKTKDNTANGNTAAASGGAGAAASGGAAAMPSENSKL
NmTbpB_P2396        GPQGEELGFRFLSDDKKVAVVGSAKTQDKPENGA..AASGGTDAAASNGAAGTSSENSKL
NmTbpB_MC58         GPQGEELGFRFLSDDQKVAVVGSAKTKDKPANGNTAAASGGTDAAASNGAAGTSSENGKL
NmTbpB_120M         GPQGEELGFRFLSDDQKVAVVGSAKTKDKPANGNTAAASGGTDAAASNGAAGTSSENGKL
NmTbpB_931905       GRKGEELGFRFLSDDKKVAVVGSAKTQDKPENGA..AALGGAGAAASGGVAAMPSENGKL
NmTbpB_NG114/82     GPKGEELGFRFLSDDQKVAVVGSAKTQDKPGNGA..AASGGTGAAASGGAADMPSENSKL
NmTbpB_H44/76       GPQGEELGFRFLSDDKKVAVVGSAKTKDKPGNGA..AAPGGTDAAASNGAAGTSSENSKL
NmTbpB_G2136        GPKGEELGFRFLSDDQKVAVVGSAKTQDKPRNGA..VASGGAGAAASNGAAGTSSENSKL
NgTbpB_WHO_P        GPKGEELGFRFLSDDGKVAVVGSAKTKDETASSGGT..SGGASVS.ASGGTTGTPSENKL
NgTbpB_WHO_Y        GPQGEELGFRFLSHDNKVAVVGSAKTKDKNANGNTA..AAGT.....AGAAGMSSEDTKL
NgTbpB_WHO_W        GPQGKELGFRFLSDDGKVAVVGSAKTGDKAENGGNGASGGASVSVSNGAAGTSSENGKL
NgTbpB_WHO_M        GPQGEELGFRFLSDDGKVAVVGSAKTKDNTANGNPAVSS.......GAGAAAM.PSETGL
NgTbpB_WHO_U        GPQGEELGFRFLSDDGKVAVVGSAKTKDNTANGNPAVSS.......GAGAAAM.PSETGL
```

```
                        350       360       370       380
NmTbpB_M597         ETVIDAYRITGEEFKKEQIDSFGDVKKLLVDGVELSLLPSEGNKA...............
NmTbpB_B16B6        ETVIDAYRITGEEFKKEQIDSFGDVKKLLVDGVELSLLPSEGNKA...............
NgTbpB_FA19         TTVLDAVELTPDGKEIKNLDNFSNATRLVVDGIMIPLLPTES..GNGQADKG......KN
NmTbpB_M978         TTVLDAVELTLNDKKIKNLDNFSNAAQLVVDGIMIPLLPETSESGSNQADKGKKGKNGKN
NmTbpB_020          TTVLDAVELTHGGTAIKKLDNFSNAAQLVVDGIMIPLLPEASESGNNQANQG......TN
NmTbpB_M1080        TTVLDAVELTHGGTAIKNLDNFSNAAQLVVDGIMIPLLPEASESGN..TNQG......TN
NmTbpB_P2396        TTVLDAVELKSGGKEVKNLDNFSNAAQLVVDGIMIPLLPKDSESGNTQADKG......KN
NmTbpB_MC58         TTVLDAVELKLGDKEVQKLDNFSNAAQLVVDGIMIPLLPEASESGNNQANQG......TN
NmTbpB_120M         TTVLDAVELKLGDKEVQKLDNFSNAAQLVVDGIMIPLLPEASESGNNQANQG......TN
NmTbpB_931905       TTVLDAVELTHGGTAIKNLDNFSNAAQLVVDGIMIPLLPETSESGKNQADKG......KN
NmTbpB_NG114/82     TTVLDAVELTHGGTAIKNLDNFSNAAQLVVDGIMIPLLPEASESGHNQANQG......TN
NmTbpB_H44/76       TTVLDAVELKSGGKEVKNLDNFSNAAQLVVDGIMIPLLPKDSESGNTQADKG......KN
NmTbpB_G2136        TTVLDAVELTLNDKKIKNLDNFSNAAQLVVDGIMIPLLPEASESGKNQANQG......TN
NgTbpB_WHO_P        TTVLDAVELTPDGKKIKDLDNFSNAAQLVVDGIMIPLLPT..ESGNGQADKG......EN
NgTbpB_WHO_Y        TTVLDAVELTPDGKKVKNLDNFSDATQLVVDGIMIPLLPT..ESGNGQADKG......EN
NgTbpB_WHO_W        TTVLDAVELTPDGKEIKNLDNFSNATRLVVDGIMIPLLPT..ESGDGQADKG......EN
NgTbpB_WHO_M        TTVLDAVELTLDGKEIKNLDNFSDATRLVVDGIMIPLLST..ESGDGQADKG......KN
NgTbpB_WHO_U        TTVLDAVELTLDGKEIKNLDNFSDATRLVVDGIMIPLLST..ESGDGQADKG......KN
```

```
                      390                                     400
                        .                                       .
NmTbpB_M597       ...AFQHE.........................................IEQNGVKATVC
NmTbpB_B16B6      ...AFQHE.........................................IEQNGVKATVC
NgTbpB_FA19       GGTDFTYE....TTYTPESDKKDTKAQTGAGGMQTASGTAG...VNGGQVGTKTYKVQVC
NmTbpB_M978       GGTDFTYK....TTYTPKNDDKDTKAQTGAAGSSGAQTDLGKADVNGGKAETKTYEVEVC
NmTbpB_020        GGTAFTRK....FDYTPKSDEKDAQAGTAANGDQAASNTAG.....DANGKTKTYAVEVC
NmTbpB_M1080      GGTAFTRK....FAHTPKSDEKDTQAGTAANGDQAASNTAG.....DTNGKTKTYEVEVC
NmTbpB_P2396      GGTEFTRK....FEHTPESDKKDAQAGTQTNGAQTASNTAG.....DTNGKTKTYEVEVC
NmTbpB_MC58       GGTAFTRK....FDHTPESDKKDAQAGTQTNGAQTASNTAG.....DTNGKTKTYEVEVC
NmTbpB_120M       GGTAFTRK....FDHTPESDKKDAQAGTQTNGAQTASNTAG.....DTNGKTKTYEVEVC
NmTbpB_931905     GETEFTRK....FDYTPESDKKDTQAGTPTNGAQTASNTAG.....DTNGKTKTYEVEVC
NmTbpB_NG114/82   GGTAFTRK....FNHTPTSDEKDTQAGTAANGNQAASNTAG.....DANGKTKTYAVEVC
NmTbpB_H44/76     GGTEFTRK....FEHTPESDKKDAQAGTAENGNPAASNTAG...DTNGK..TKTYAVEVC
NmTbpB_G2136      GGTAFTRK....FNHTPKSDEKDTQAGTAENGNPAASNTAG...DANGK..TKTYEVEVC
NgTbpB_WHO_P      GKTAFIYE....TTYTPESDKKDTQTGMATNGVQTVSNTAG...GTSGKT.KTHYKVQAC
NgTbpB_WHO_Y      GKTAFIYE....TTYTPESDKKDTQTGMATNGVQTVSNTAG...GTSGKT.KTHYKVQAC
NgTbpB_WHO_W      GKTAFIYE....TTYTPESDEKNTQAQTGAGGMQTVSNAAG...GT..SGETKTYKVQAC
NgTbpB_WHO_M      GGTDFTYTTTYTTTYTPESDKKDTKAQTGAVGMQTAPGAAG...VNGGQAGTKTYEVEAC
NgTbpB_WHO_U      GGTDFTYTTTYTTTYTPESDKKDTKAQTGAVGMQTAPGAAG...VNGGQAGTKTYEVEAC

                     410                                  420       430       440
                       .                                    .         .         .
NmTbpB_M597       CSNLDYMSFGKLSKEN.....................KDDMFLQGVRTPVSDVAARTEAN
NmTbpB_B16B6      CSNLDYMSFGKLSKEN.....................KDDMFLQGVRTPVSDVAARTEAN
NgTbpB_FA19       CSNLNYLKYGLLTRENNNSVMQAVKNSSQADAKTKQIEQSMFLQGERTDENKIPQE..QG
NmTbpB_M978       CSNLNYLKYGMLTRKNSKSAMQAGGNSSQADAKTEQVEQSMFLQGERTDEKEIPND..QN
NmTbpB_020        CSNLNYLKYGLLTRKTAGNTGEGGNGSPTA..AAQTDAQSMFLQGERTDEKEIPND..QN
NmTbpB_M1080      CSNLNYLKYGLLTRKTAGNTGEGGNGSPTA...AQTGAQSMFLQGERTDEKEIPKE.QQN
NmTbpB_P2396      CSNLNYLKYGMLTRKNSKSAMQAGENGSQADAKTEQVEQSMFLQGERTDEKEIPTD..QN
NmTbpB_MC58       CSNLNYLKYGMLTRKNSKSAMQAGESSSQADAKTEQVEQSMFLQGERTDEKEIPSE..QN
NmTbpB_120M       CSNLNYLKYGMLTRKNSKSAMQAGESSSQADAKTEQVEQSMFLQGERTDEKEIPSE..QN
NmTbpB_931905     CSNLNYLKYGLLTRKTAGNTVGSGNGSQ...AAAQTDAQSMFLQGERTDEKEIPSE..QN
NmTbpB_NG114/82   CSNLNYLKYGLLTRKTAGNTGEGGNGSQ...TAAQTDAQSMFLQGERTDENKIPTD..QN
NmTbpB_H44/76     CSNLNYLKYGLLTRKTAGNTGEGGNGAA......QTDAQSMFLQGERTDEKEIPN..DQN
NmTbpB_G2136      CSNLNYLKYGMLTRKNSKSAMQAGESSSQADAKTEQVGQSMFLQGERTDEKEIPN..DQN
NgTbpB_WHO_P      CSNLNYLKYGLLTRKNSESAMQAGESSSRTAVQTAQGAQSMFLQGERTDEKEIPK..DGN
NgTbpB_WHO_Y      CSNLNYLKYGLLTRENSNSVMQTVRNSSQAAARTEQGAQSMFLQGERTDEKEIPK..EQK
NgTbpB_WHO_W      CSNLNYLKYGLLTRKTAGNT.GGGGKGSQAAAQTAQGARSMFLQGERTDENKIPQ..GQD
NgTbpB_WHO_M      CSNLNYLKYGMLTRKNSESAMQAGGSSNQADAKTEQAEQSMFLQGERTPVSDMAARTEAN
NgTbpB_WHO_U      CSNLNYLKYGMLTRKNSESAMQAGGSSNQADAKTEQAEQSMFLQGERTPVSDMAARTEAN

                      450       460       470       480       490
                        .         .         .         .         .
NmTbpB_M597       AKYRGTWYGYIA.NG..TSWSGEASNQEGGNRAEFDVDFSTKKISGTLTAKDRTSPAFTI
NmTbpB_B16B6      AKYRGTWYGYIA.NG..TSWSGEASNQEGGNRAEFDVDFSTKKISGTLTAKDRTSPAFTI
NgTbpB_FA19       IVYLGFWYGRIA.NG..TSWSGKASNATDGNRAKFTVNFDRKEITGTLTAENRSEATFTI
NmTbpB_M978       VVYRGSWYGHIA.SS..TSWSGNASNATSGNRAEFTVNFDTKKINGTLTAENRQEATFTI
NmTbpB_020        IVYRGSWYGHIA.NG..TSWSGNASNATSGNKADFTVNFGEKKINGTLTAENRQAATFTI
NmTbpB_M1080      IVYRGSWYGHIA.NG..TSWSGNASNATSGNKADFTVNFGEKKINGTLTAENRQAATFTI
NmTbpB_P2396      VVYRGSWYGYIA.NDKSTSWSGNASDREGGNRADFTVNFGEKKITGTLTAENRQAATFTI
NmTbpB_MC58       IVYRGSWYGYIA.NDKSTSWSGNASNATSGNRAEFTVNFADKKITGTLTADNRQEATFTI
NmTbpB_120M       VVYRGSWYGHIA.N..GTSWSGNASDKEGGNRAEFTVDFGAKKINGKLTAENRQAATFTI
NmTbpB_931905     VVYRGSWYGHIA.NG..TSWSGNASDKEGGNRAEFTVDFGAKKINGKLTAENRQAATFTI
NmTbpB_NG114/82   VVYRGSWYGHIA.NG..TSWSGNASDKEGGNRAEFTVNFGEKKINGTLTAENRQAATFTI
NmTbpB_H44/76     IVYRGSWYGHIA.NG..TSWSGNASDKEGGNRAEFTVNFADKKLNGTLTAGERTSPTFTI
NmTbpB_G2136      VVYRGSWYGHIA.NG..TSWSGNASDKEGGNRADFTVNFGTKKINGTLTADNRQAATFTI
NgTbpB_WHO_P      VVYLGTWYGHIAING..TSWTREASNQENGNRAKFDVNFKDKKITGTLTAANRQEATFTI
NgTbpB_WHO_Y      VVYLGTWYGHIAANG..TSWTGKASDQQSGNRAKFDVNFKDKKITGTLTAANRQAETFTI
NgTbpB_WHO_W      IVYRGSWYGYIV.SG..TSWSGNASNATSGNRAEFTVNFDAKKINGKLTAENRSEATFTI
NgTbpB_WHO_M      AKYRGTWYGYIA.NG..TSWSGNASNTTSGNKAEFTVNFDKKKITGMLTAANRQEATFTI
NgTbpB_WHO_U      AKYRGTWYGYIA.NG..TSWSGNASNTTSGNKAEFTVNFDKKKITGMLTAANRQEATFTI

                 500       510       520       530       540       550
                   .         .         .         .         .         .
NmTbpB_M597       TAMIKDNGFSGVAKTGENGFALDPQNTGNSHYTHI.EATVSGGFYGKNAIEMGGSFSFPG
NmTbpB_B16B6      TAMIKDNGFSGVAKTGENGFALDPQNTGNSHYTHI.EATVSGGFYGKNAIEMGGSFSFPG
NgTbpB_FA19       DAMIEGNGFKGTAKTGNDGFAPDQNNSTVTHKVHIANAEVQGGFYGPNAEELGGWFAYPG
NmTbpB_M978       DGKIEGNGFSGTAKTADLGFDLDQSNTTGTPKAYITDAKVQGGFYGPKAEELGGWFAYPG
NmTbpB_020        EGTIQGNGFSGTAKTADSGFDLDQSNTTGTPKAYITDAKVQGGFYGPKAEELGGWFAYPG
NmTbpB_M1080      EGMIQGNGFSGTAKTADLGFDLDQSNTTGTPKAYITNAKVQGGFYGPKAEELGGWFAYPG
NmTbpB_P2396      EGDIEGNGFSGTAKTDNLGFDLDQSDNTRTPKAYITDAKVQGGFYGPRAEELGGWFAYPG
NmTbpB_MC58       DGNIKDNGFEGTAKTAESGFDLDQSNTTRTPKAYITDAKVQGGFYGPKAEELGGWFAYPG
NmTbpB_120M       EGDIEGNGFSGTAKTAESGFDLDQSNTTRTPKAYITDAKVQGGFYGPKAEELGGWFAYPG
NmTbpB_931905     EGDIEGNGFSGTAKTAESGFDLDQSNTTRTPKAYITDAKVQGGFYGPKAEELGGWFAYPG
NmTbpB_NG114/82   EGDIKDNGFEGTAKTADLGFDLDQSNTTGTPKAYITNAKVQGGFYGPKADELGGWFAYPG
NmTbpB_H44/76     TATIQGNGFEGTAKTGDDGFALDTKNTVDTHKAHITDANVQGGFYGPKAEELGGWFAYPG
NmTbpB_G2136      VGDIEGNGFSGTAKTADSGFDLDQSNNTRTPKAYITNAKVQGGFYGPKAEELGGWFAYSD
NgTbpB_WHO_P      DAMIEGNGFKGTAKTGDGGFAPDQNNSTGTHKVHIAEAKVQGGFYGPNAEELGGWFAYPG
NgTbpB_WHO_Y      SGMIDGNGFEGTAKTGNGGFALDANNTAATHKAHIAEAKVRGGFYGPNAEELGGWFAYPG
NgTbpB_WHO_W      DAMIDGNGFKGTAKTGNGGFAPDQNSSTGTHKVHITSAAVQGGFYGPKAEELGGWFAYPG
NgTbpB_WHO_M      DAMIESNGFKGMAKTGNGGFAPDQNSSTGTHKVHITSAAVQGGFYGPKAEELGGWFAYPG
NgTbpB_WHO_U      DAMIESNGFKGMAKTGNGGFAPDQNSSTGTHKVHITSAAVQGGFYGPKAEELGGWFAYPG
```

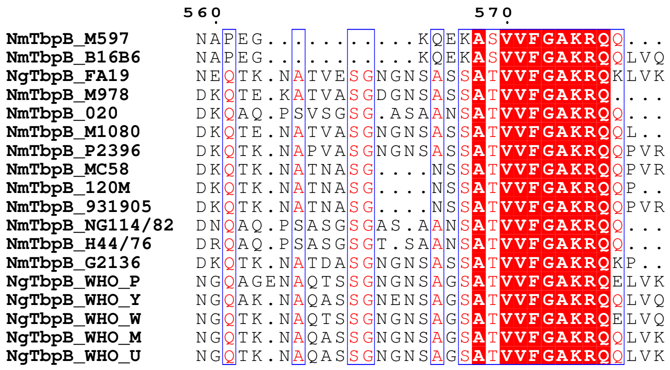
560
570
NmTbpB_M597 NAPEG..........KQEKASVVFGAKRQQ...
NmTbpB_B16B6 NAPEG..........KQEKASVVFGAKRQQLVQ
NgTbpB_FA19 NEQTK.NATVESSGNGNSASSATVVFGAKRQKLVK
NmTbpB_M978 DKQTE.KATVASGDGNSASSATVVFGAKRQ....
NmTbpB_020 DKQAQ.PSVSGSG.ASAANSATVVFGAKRQQ...
NmTbpB_M1080 DKQTE.NATVASGNGNSASSATVVFGAKRQQL..
NmTbpB_P2396 DKQTK.NAPVASGNGNSASSATVVFGAKRQQPVR
NmTbpB_MC58 DKQTK.NATNASG....NSSATVVFGAKRQQPVR
NmTbpB_120M DKQTK.NATNASG....NSSATVVFGAKRQQP..
NmTbpB_931905 DKQTK.NATNASG....NSSATVVFGAKRQQPVR
NmTbpB_NG114/82 DNQAQ.PSASGSGAS.AANSATVVFGAKRQQ...
NmTbpB_H44/76 DRQAQ.PSASGSGT.SAANSATVVFGAKRQQ...
NmTbpB_G2136 DKQTK.NATDASGNGNSASSATVVFGAKRQKP..
NgTbpB_WHO_P NGQAGENAQTSSGNGNSAGSATVVFGAKRQELVK
NgTbpB_WHO_Y NGQAK.NAQASSGNENSAGSATVVFGAKRQQLVQ
NgTbpB_WHO_W NGQTK.NAQTSSGNGNSAGSATVVFGAKRQELVQ
NgTbpB_WHO_M NGQTK.NAQASSGNGNSAGSATVVFGAKRQQLVK
NgTbpB_WHO_U NGQTK.NAQASSGNGNSAGSATVVFGAKRQQLVK

**Supplementary Figure 3. Sequence alignment of LbpAs.**

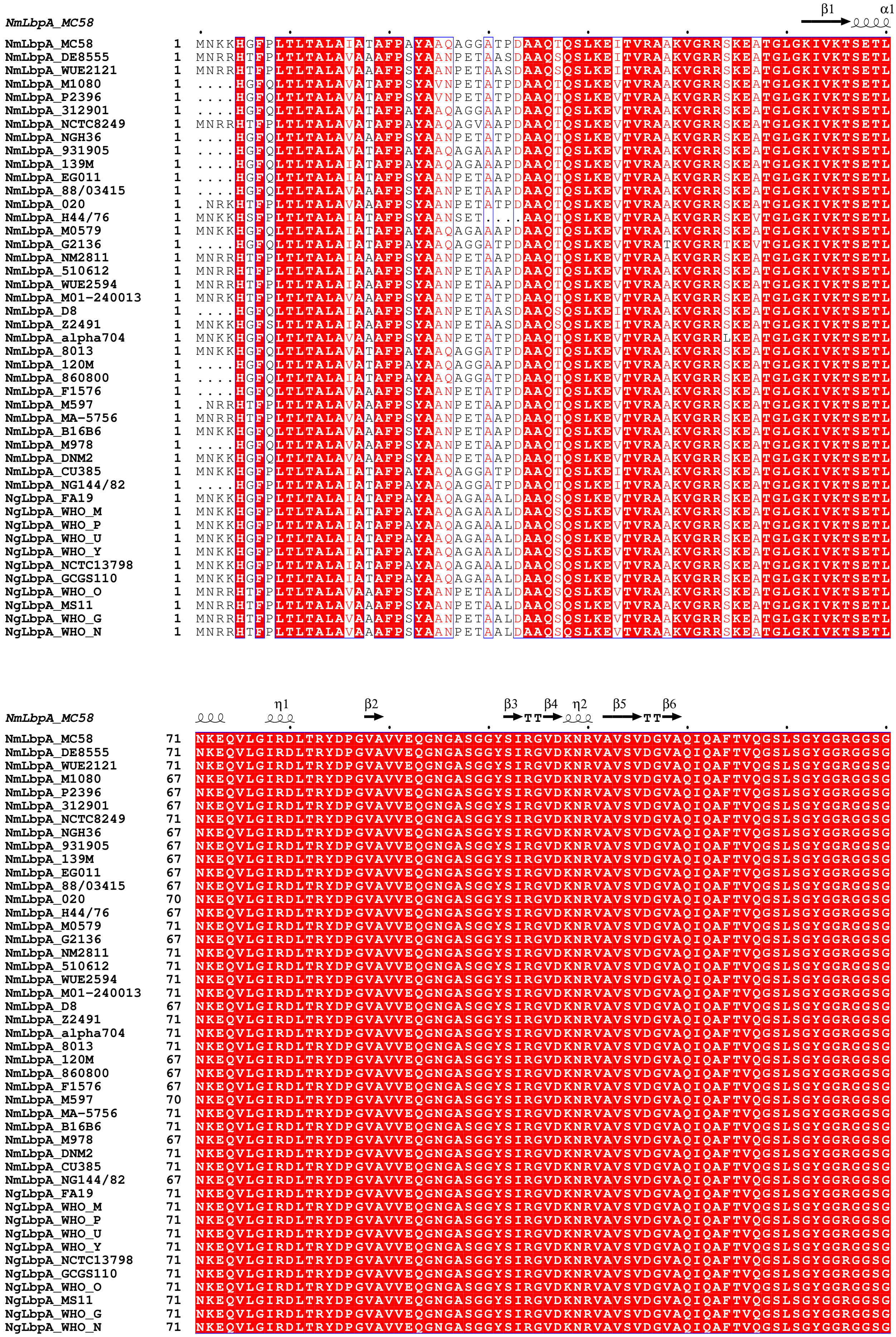

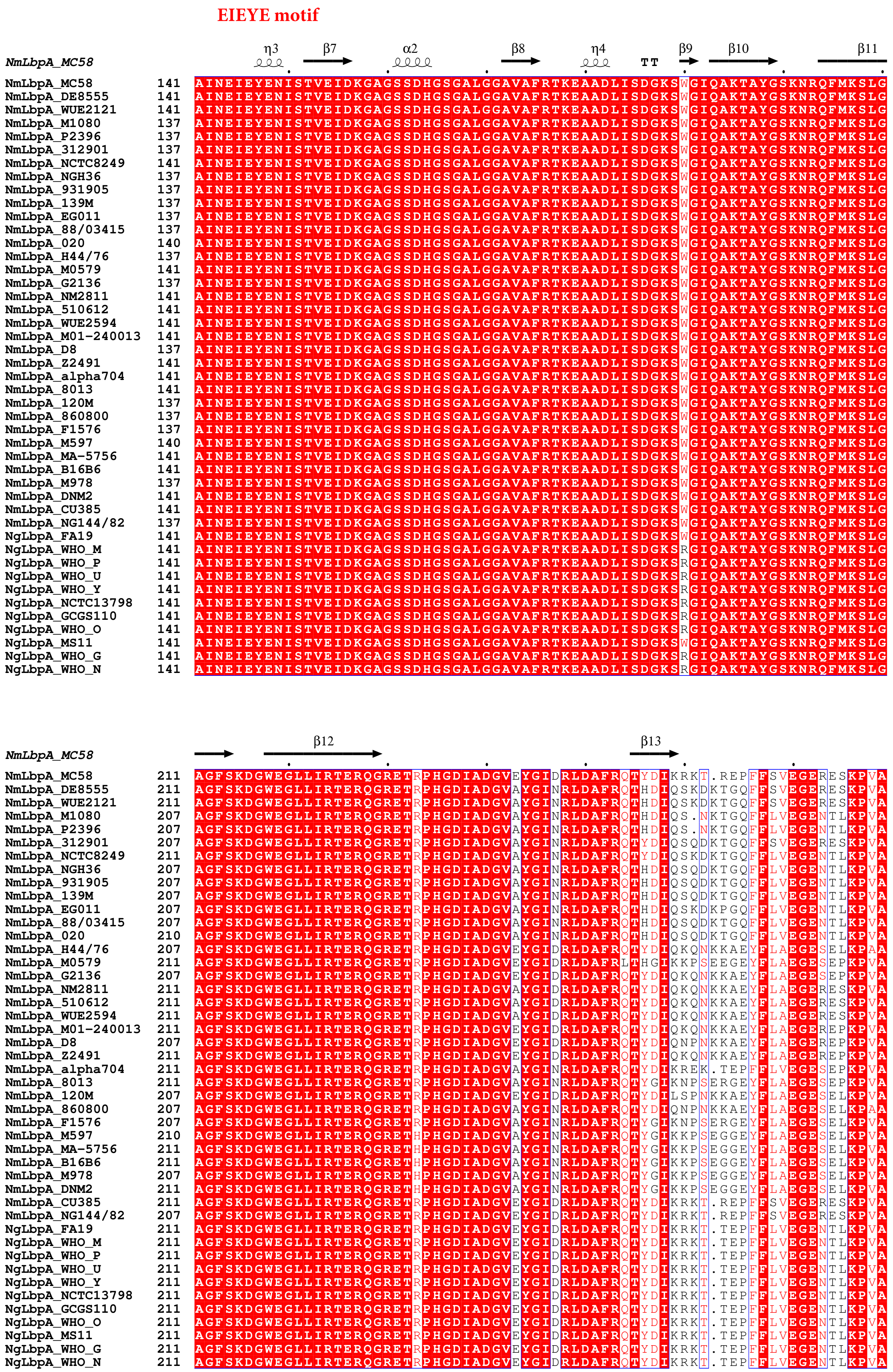
EIEYE motif
NmLbpA_MC58
η3
β7
α2
β8
η4
TT
β9
β10
β11
β12
β13

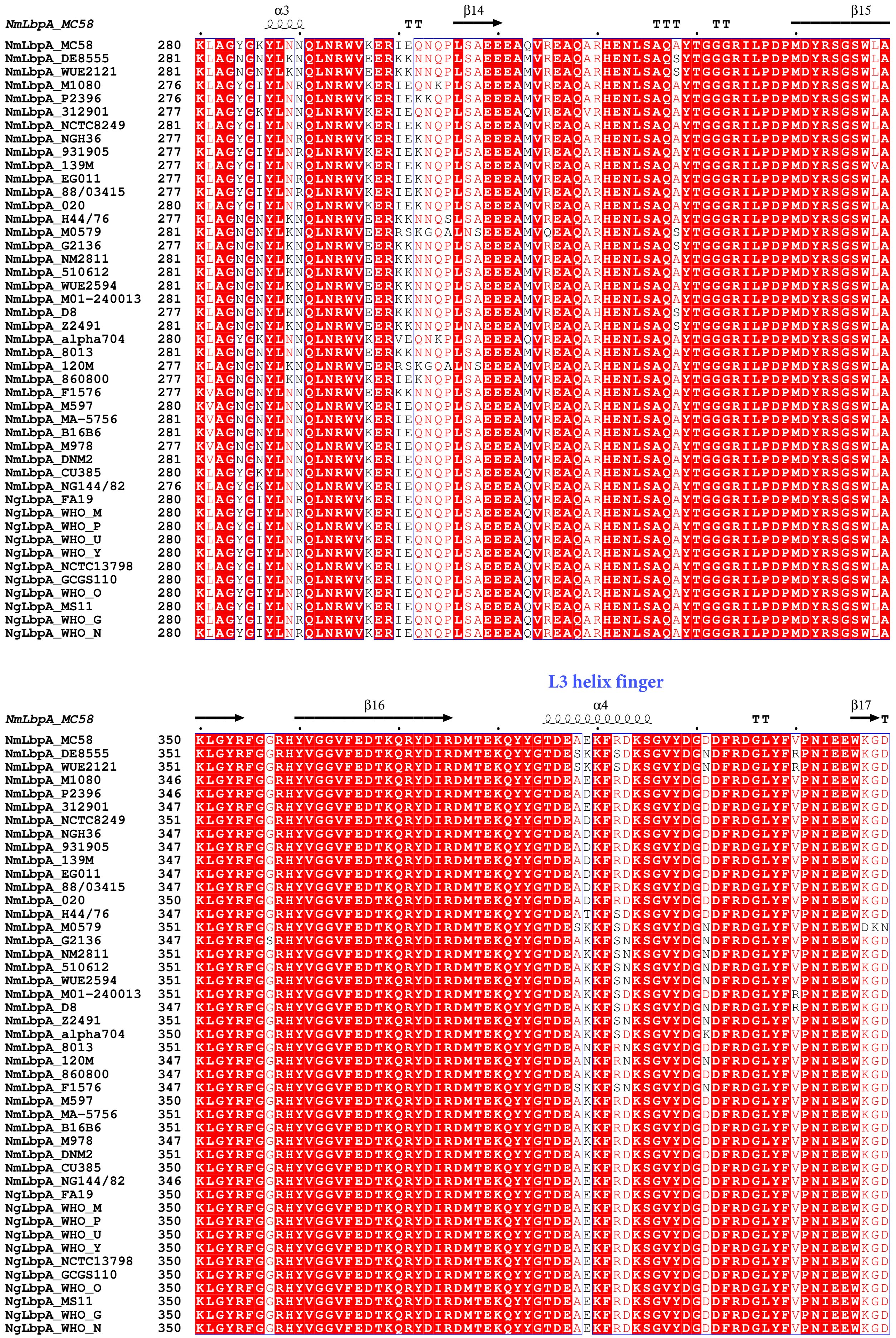

NmLbpA_MC58
α3
TT
β14
TTT
TT
β15
L3 helix finger
NmLbpA_MC58
β16
α4
TT
β17
T

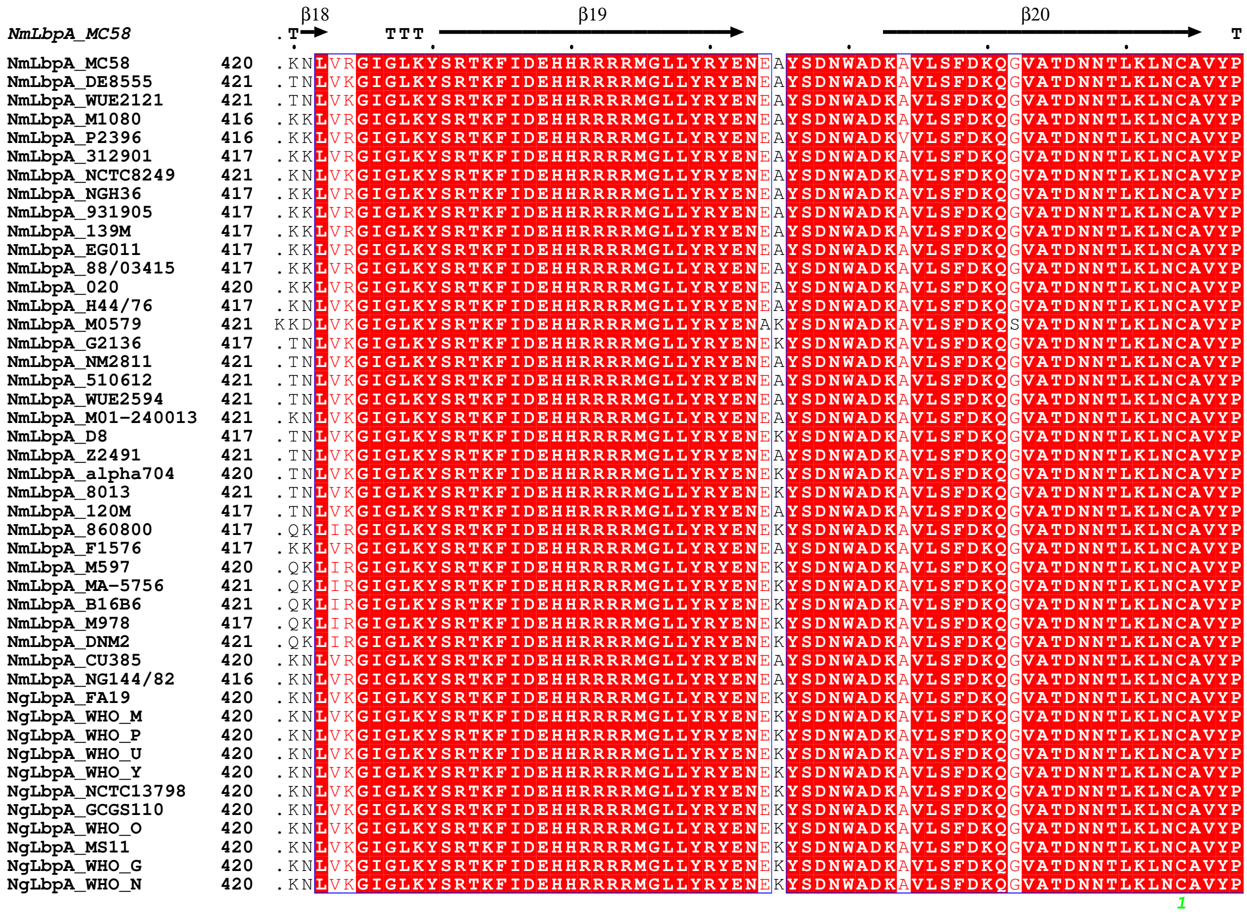
β18
β19
β20
NmLbpA_MC58
NmLbpA_MC58 420
NmLbpA_DE8555 421
NmLbpA_WUE2121 421
NmLbpA_M1080 416
NmLbpA_P2396 416
NmLbpA_312901 417
NmLbpA_NCTC8249 421
NmLbpA_NGH36 417
NmLbpA_931905 417
NmLbpA_139M 417
NmLbpA_EG011 417
NmLbpA_88/03415 417
NmLbpA_020 420
NmLbpA_H44/76 417
NmLbpA_M0579 421
NmLbpA_G2136 417
NmLbpA_NM2811 421
NmLbpA_510612 421
NmLbpA_WUE2594 421
NmLbpA_M01-240013 421
NmLbpA_D8 417
NmLbpA_Z2491 421
NmLbpA_alpha704 420
NmLbpA_8013 421
NmLbpA_120M 417
NmLbpA_860800 417
NmLbpA_F1576 417
NmLbpA_M597 420
NmLbpA_MA-5756 421
NmLbpA_B16B6 421
NmLbpA_M978 417
NmLbpA_DNM2 421
NmLbpA_CU385 420
NmLbpA_NG144/82 416
NgLbpA_FA19 420
NgLbpA_WHO_M 420
NgLbpA_WHO_P 420
NgLbpA_WHO_U 420
NgLbpA_WHO_Y 420
NgLbpA_NCTC13798 420
NgLbpA_GCGS110 420
NgLbpA_WHO_O 420
NgLbpA_MS11 420
NgLbpA_WHO_G 420
NgLbpA_WHO_N 420

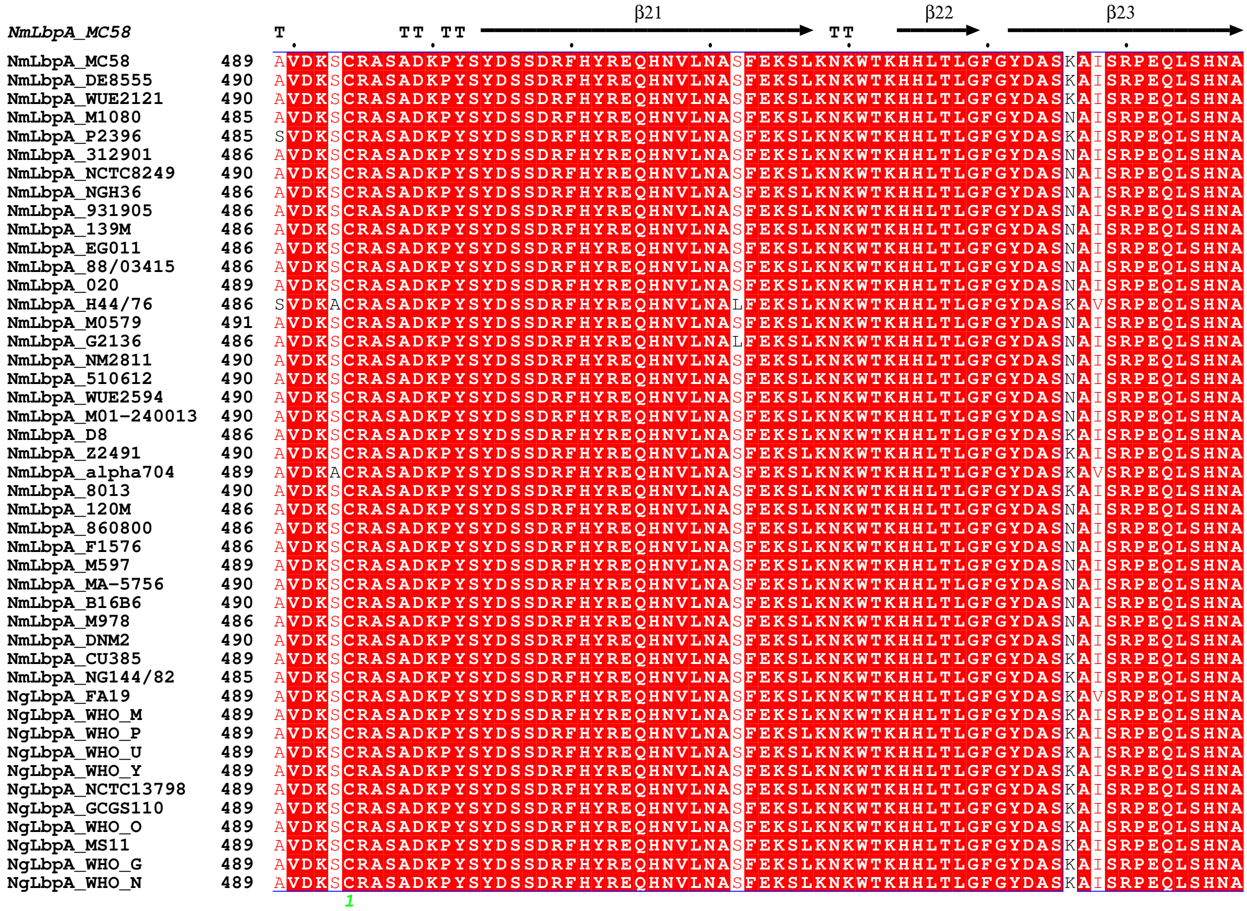
β21
β22
β23
NmLbpA_MC58
NmLbpA_MC58 489
NmLbpA_DE8555 490
NmLbpA_WUE2121 490
NmLbpA_M1080 485
NmLbpA_P2396 485
NmLbpA_312901 486
NmLbpA_NCTC8249 490
NmLbpA_NGH36 486
NmLbpA_931905 486
NmLbpA_139M 486
NmLbpA_EG011 486
NmLbpA_88/03415 486
NmLbpA_020 489
NmLbpA_H44/76 486
NmLbpA_M0579 491
NmLbpA_G2136 486
NmLbpA_NM2811 490
NmLbpA_510612 490
NmLbpA_WUE2594 490
NmLbpA_M01-240013 490
NmLbpA_D8 486
NmLbpA_Z2491 490
NmLbpA_alpha704 489
NmLbpA_8013 490
NmLbpA_120M 486
NmLbpA_860800 486
NmLbpA_F1576 486
NmLbpA_M597 489
NmLbpA_MA-5756 490
NmLbpA_B16B6 490
NmLbpA_M978 486
NmLbpA_DNM2 490
NmLbpA_CU385 489
NmLbpA_NG144/82 485
NgLbpA_FA19 489
NgLbpA_WHO_M 489
NgLbpA_WHO_P 489
NgLbpA_WHO_U 489
NgLbpA_WHO_Y 489
NgLbpA_NCTC13798 489
NgLbpA_GCGS110 489
NgLbpA_WHO_O 489
NgLbpA_MS11 489
NgLbpA_WHO_G 489
NgLbpA_WHO_N 489

```
                                                    β24                  β25
NmLbpA_MC58                                                  TT
NmLbpA_MC58          559  ARISESTG.FDENNQDKYLLGKPEVVEGSVCGYIETLRSRKCVPRKINGSNIHISLNDRFSIGKYFDFSL
NmLbpA_DE8555        560  ARISEHTGEYTDDGKDKYRLGKPEVVEGSVCGYIETLRSRKCVPRKINGSNIHISLNDRFSIGKYFDFSL
NmLbpA_WUE2121       560  ARISEHTGEYTDDGKDKYRLGKPEVVEGSVCGYIETLRSRKCVPRKINGSNIHISLNDRFSIGKYFDFSL
NmLbpA_M1080         555  ARISESTGF.DEKNQDKYLLGKPEVIEGSVCGYIETLRSRKCVPRKINGSNIHISLNDRFSIGKYFDFSL
NmLbpA_P2396         555  ARISESTGF.DDKNQDKYLLGKPEVVEGSVCGYIETLRSRKCVPRKINGSNIHISLNDRFSIGKYFDFSL
NmLbpA_312901        556  ARISEYSDY..TDKGDKYLLGKPEVVEGSVCGYIETLRSRKCVPRKINGSNIHISLNDRFSIGKYFDFSL
NmLbpA_NCTC8249      560  ARISEHTGEYTADDKDKYRLGKPEVVEGSVCGYIETLRSRKCVPRKINGSNIHISLNDRFSIGKYFDFSL
NmLbpA_NGH36         556  ARISEHTGEYTADDKDKYRLGKPEVVEGSVCGYIETLRSRKCVPRKINGSNIHISLNDRFSIGKYFDFSL
NmLbpA_931905        556  ARISESTG.FDEKNQDKYRLGKPEVVEGSVCGYIETLRSRKCVPRKINGSNIHISLNDRFSIGKYFDFSL
NmLbpA_139M          556  ARISESTG.FDEKNQDKYRLGKPEVVEGSVCGYIETLRSRKCVPRKINGSNIHISLNDRFSIGKYFDFSL
NmLbpA_EG011         556  ARISESTG.FDEKNQDKYRLGKPEVVEGSVCGYIETLRSRKCVPRKINGSNIHISLNDRFSIGKYFDFSL
NmLbpA_88/03415      556  ARISESTG.FDEKNQDKYRLGKPEVVEGSVCGYIETLRSRKCVPRKINGSNIHISLNDRFSIGKYFDFSL
NmLbpA_020           559  ARISESTG.FDEKNQDKYRLGKPEVVEGSVCGYIETLRSRKCVPRKINGSNIHISLNDRFSIGKYFDFSL
NmLbpA_H44/76        556  ARISEFS.DYADDGKYKYLLGKPEVVEGSVCGYIETLRSRKCVPRKINGSNIHISLNDRFSIGKYFDFSL
NmLbpA_M0579         561  ARISESTG.FDEKNQDKYRLGKPEVVEGSVCGYIETLRSRKCVPRKINGSNIHISLNDRFSIGKYFDFSL
NmLbpA_G2136         556  ARISESTG.FDEKNQDKYRLGKPEVVEGSVCGYIETLRSRKCVPRKINGSNIHISLNDRFSIGKYFDFSL
NmLbpA_NM2811        560  ARISEHTGEYTADDKDKYRLGKPEVVEGSVCGYIETLRSRKCVPRKINGSNIHISLNDRFSIGKYFDFSL
NmLbpA_510612        560  ARISEHTGEYTADDKDKYRLGKPEVVEGSVCGYIETLRSRKCVPRKINGSNIHISLNDRFSIGKYFDFSL
NmLbpA_WUE2594       560  ARISEHTGEYTADDKDKYRLGKPEVVEGSVCGYIETLRSRKCVPRKINGSNIHISLNDRFSIGKYFDFSL
NmLbpA_M01-240013    560  ARISESTG.FDDNNQDKYRLGKPEVVEGSVCGYIETLRSRKCVPRKINGSNIHISLNDRFSIGKYFDFSL
NmLbpA_D8            556  ARISESTG.FDDNNQDKYRLGKPEVVEGSVCGYIETLRSRKCVPRKINGSNIHISLNDRFSIGKYFDFSL
NmLbpA_Z2491         560  ARISESTG.FDDNNQDKYLLGKPEVVEGSVCGYIETLRSRKCVPRKINGSNIHISLNDRFSIGKYFDFSL
NmLbpA_alpha704      559  ARISEHTGEYTDDGKDKYRLGKPEVVEGSVCGYIETLRSRKCVPRKINGSNIHISLNDRFSIGKYFDFSL
NmLbpA_8013          560  ARISEHTGEYTDDGKDKYRLGKPEVVEGSVCGYIETLRSRKCVPRKINGSNIHISLNDRFSIGKYFDFSL
NmLbpA_120M          556  ARISEYSD.YT.DKGDKYLLGKPEVVEGSVCGYIETLRSRKCVPRKINGSNIHISLNDRFSIGKYFDFSL
NmLbpA_860800        556  ARISEYSD.YT.DKGDKYLLGKPEVVEGSVCGYIETLRSRKCVPRKINGSNIHISLNDRFSIGKYFDFSL
NmLbpA_F1576         556  ARISEYSD.YT.DKGDKYLLGKPEVVEGSVCGYIETLRSRKCVPRKINGSNIHISLNDRFSIGKYFDFSL
NmLbpA_M597          559  ARISEYSD.YT.DKGDKYLLGKPEVVEGSVCGYIETLRSRKCVPRKINGSNIHISLNDRFSIGKYFDFSL
NmLbpA_MA-5756       560  ARISEYSD.YT.DKEDKYLLGKPEVVEGSVCGYIETLRSRKCVPRKINGSNIHISLNDRFSIGKYFDFSL
NmLbpA_B16B6         560  ARISEYSD.YT.DKGDKYLLGKPEVVEGSVCGYIETLRSRKCVPRKINGSNIHISLNDRFSIGKYFDFSL
NmLbpA_M978          556  ARISEYSD.YT.DKGDKYLLGKPEVVEGSVCGYIETLRSRKCVPRKINGSNIHISLNDRFSIGKYFDFSL
NmLbpA_DNM2          560  ARISEYSD.YT.DKGDKYLLGKPEVVEGSVCGYIETLRSRKCVPRKINGSNIHISLNDRFSIGKYFDFSS
NmLbpA_CU385         559  ARISESTG.FDENNQDKYLLGKPEVVEGSVCGYIETLRSRKCVPRKINGSNIHISLNDRFSIGKYFDFSL
NmLbpA_NG144/82      555  ARISESTG.FDENNQDKYLLGKPEVVEGSVCGYIETLRSRKCVPRKINGSNIHISLNDRFSIGKYFDFSL
NgLbpA_FA19          559  ARISESTG.FDEKNQDKYRLGKPEVVEGSVCGYIETLRSRKCVPRKINGSNIHISLNDRFSIGKYFDFSL
NgLbpA_WHO_M         559  ARISESTG.FDEKNQDKYRLGKPEVVEGSVCGYIETLRSRKCVPRKINGSNIHISLNDRFSIGKYFDFSL
NgLbpA_WHO_P         559  ARISESTG.FDEKNQDKYRLGKPEVVEGSVCGYIETLRSRKCVPRKINGSNIHISLNDRFSIGKYFDFSL
NgLbpA_WHO_U         559  ARISESTG.FDEKNQDKYRLGKPEVVEGSVCGYIETLRSRKCVPRKINGSNIHISLNDRFSIGKYFDFSL
NgLbpA_WHO_Y         559  ARISESTG.FDEKNQDKYRLGKPEVVEGSVCGYIETLRSRKCVPRKINGSNIHISLNDRFSIGKYFDFSL
NgLbpA_NCTC13798     559  ARISESTG.FDEKNQDKYRLGKPEVVEGSVCGYIETLRSRKCVPRKINGSNIHISLNDRFSIGKYFDFSL
NgLbpA_GCGS110       559  ARISESTG.FDEKNQDKYRLGKPEVVEGSVCGYIETLRSRKCVPRKINGSNIHISLNDRFSIGKYFDFSL
NgLbpA_WHO_O         559  ARISESTG.FDEKNQDKYRLGKPEVVEGSVCGYIETLRSRKCVPRKINGSNIHISLNDRFSIGKYFDFSL
NgLbpA_MS11          559  ARISESTG.FDEKNQDKYRLGKPEVVEGSVCGYIETLRSRKCVPRKINGSNIHISLNDRFSIGKYFDFSL
NgLbpA_WHO_G         559  ARISESTG.FDEKNQDKYRLGKPEVVEGSVCGYIETLRSRKCVPRKINGSNIHISLNDRFSIGKYFDFSL
NgLbpA_WHO_N         559  ARISESTG.FDEKNQDKYRLGKPEVVEGSVCGYIETLRSRKCVPRKINGSNIHISLNDRFSIGKYFDFSL
                                                      2           2

                           β26              β27      β28      β29         α5
NmLbpA_MC58                             TT                                             TT
NmLbpA_MC58          628  GGRYDRKNFTTSEELVRSGRYVDRSWNSGILFKPNRHFSVSYRASSGFRTPSFQELFGIDIYHDYPKGWQ
NmLbpA_DE8555        630  GGRYDRQNFTTSEELVRSGRYVDRSWNSGIVFKPNRHFSVSYRASSGFRTPSFQELFGIDIYHDYPKGWQ
NmLbpA_WUE2121       630  GGRYDRQNFTTSEELVRSGRYVDRSWNSGIVFKPNRHFSVSYRASSGFRTPSFQELFGIDIYHDYPKGWQ
NmLbpA_M1080         624  GGRYDRKNFTTSEELVRSGRYVDRSWNSGIVFKPNRHFSVSYRASSGFRTPSFQELFGIDIYHDYPKGWQ
NmLbpA_P2396         624  GGRYDRKNFTTSEELVRSGRYVDRSWNSGIVFKPNRHFSLSYRASSGFRTPSFQELFGIDIYHDYPKGWQ
NmLbpA_312901        624  GGRYDRKNFTTSEELVRSGRYVDRSWNSGIVFKPNRHFSLSYRASSGFRTPSFQELFGIDIYHDYPKGWQ
NmLbpA_NCTC8249      630  GGRYDRKNFTTSEELVRSGRYVDRSWNSGIVFKPNRHFSLSYRASSGFRTPSFQELFGIDIYHDYPKGWQ
NmLbpA_NGH36         626  GGRYDRKNFTTSEELVRSGRYVDRSWNSGIVFKPNRHFSVSYRASSGFRTPSFQELFGIDIYHDYPKGWQ
NmLbpA_931905        625  GGRYDRKNFTTSEELVRSGRYVDRSWNSGIVFKPNRHFSLSYRASSGFRTPSFQELFGIDIYHDYPKGWQ
NmLbpA_139M          625  GGRYDRKNFTTSEELVRSGRYVDRSWNSGIVFKPNRHFSLSYRASSGFRTPSFQELFGIDIYHDYPKGWQ
NmLbpA_EG011         625  GGRYDRKNFTTSEELVRSGRYVDRSWNSGIVFKPNRHFSLSYRASSGFRTPSFQELFGIDIYHDYPKGWQ
NmLbpA_88/03415      625  GGRYDRKNFTTSEELVRSGRYVDRSWNSGIVFKPNRHFSLSYRASSGFRTPSFQELFGIDIYHDYPKGWQ
NmLbpA_020           628  GGRYDRKNFTTSEELVRSGRYVDRSWNSGIVFKPNRHFSLSYRASSGFRTPSFQELFGIDIYHDYPKGWQ
NmLbpA_H44/76        625  GGRYDRQNFTTSEELVRSGRYTDRSWNSGIVFKPSRHLSLSYRASSGFRTPSFQELFGIDIYHDYPKGWQ
NmLbpA_M0579         630  GGRYDRKNFTTSEELVRSGRYVDRSWNSGIVFKPNRHFSLSYRASSGFRTPSFQELFGIDIYHDYPKGWQ
NmLbpA_G2136         625  GGRYDRKNFTTSEELVRSGRYVDRSWNSGIVFKPNRHFSLSYRASSGFRTPSFQELFGIDIYHDYPKGWQ
NmLbpA_NM2811        630  GGRYDRKNFTTSEELVRSGRYVDRSWNSGIVFKPNRHFSLSYRASSGFRTPSFQELFGIDIYHDYPKGWQ
NmLbpA_510612        630  GGRYDRKNFTTSEELVRSGRYVDRSWNSGIVFKPNRHFSLSYRASSGFRTPSFQELFGIDIYHDYPKGWQ
NmLbpA_WUE2594       630  GGRYDRKNFTTSEELVRSGRYVDRSWNSGIVFKPNRHFSLSYRASSGFRTPSFQELFGIDIYHDYPKGWQ
NmLbpA_M01-240013    629  GGRYDRQNFTTREELVRSGRYVNRSWNSGIVFKPSRHFSLSYRASSGFRTPSFQELFGIDIYHDYPKGWQ
NmLbpA_D8            625  GGRYDRQNFTTSEELVRSGRYVDRSWNSGIVFKPNRHFSVSYRASSGFRTPSFQELFGIDIYHDYPKGWQ
NmLbpA_Z2491         629  GGRYDRQNFTTSEELVRSGRYVDRSWNSGIVFKPNRHFSVSYRASSGFRTPSFQELFGIDIYHDYPKGWQ
NmLbpA_alpha704      629  GGRYDRQNFTTSEELVRSGRYVNRSWNSGIVFKPNRHFSVSYRASSGFRTPSFQELFGIDIYHDYPKGWQ
NmLbpA_8013          630  GGRYDRQNFTTSEELVRSGRYVDRSWNSGIVFKPNRHFSVSYRASSGFRTPSFQELFGIDIYHDYPKGWQ
NmLbpA_120M          624  GGRYDRKNFTTSEELVRSGRYVDRSWNSGIVFKPNRHFSLSYRASSGFRTPSFQELFGIDIYHDYPKGWQ
NmLbpA_860800        624  GGRYDRQNFTTSEELVRSGRYVDRSWNSGIVFKPNRHFSLSYRASSGFRTPSFQELFGIDIYHDYPKGWQ
NmLbpA_F1576         624  GGRYDRKNFTTSEELVRSGRYVDRSWNSGIVFKPNRHFSVSYRASSGFRTPSFQELFGIDIYHDYPKGWQ
NmLbpA_M597          627  GGRYDRKNFTTSEELVRSGRYVDRSWNSGIVFKPNRHFSLSYRASSGFRTPSFQELFGIDIYHDYPKGWQ
NmLbpA_MA-5756       628  GGRYDRKNFTTSEELVRSGRYVDRSWNSGIVFKPNRHFSLSYRASSGFRTPSFQELFGIDIYHDYPKGWQ
NmLbpA_B16B6         628  GGRYDRKNFTTSEELVRSGRYVDRSWNSGIVFKPNRHFSLSYRASSGFRTPSFQELFGIDIYHDYPKGWQ
NmLbpA_M978          624  GGRYDRKNFTTSEELVRSGRYVDRSWNSGIVFKPNRHFSLSYRASSGFRTPSFQELFGIDIYHDYPKGWQ
NmLbpA_DNM2          628  GGRYDRKNFTTSEELVRSGRYVDRSWNSGIVFKPNRHFSLSYRASSGFRTPSFQELFGIDIYHDYPKGWQ
NmLbpA_CU385         628  GGRYDRKNFTTSEELVRSGRYVDRSWNSGILFKPNRHFSVSYRASSGFRTPSFQELFGIDIYHDYPKGWQ
NmLbpA_NG144/82      624  GGRYDRKNFTTSEELVRSGRYVDRSWNSGILFKPNRHFSVSYRASSGFRTPSFQELFGIDIYHDYPKGWQ
NgLbpA_FA19          628  GGRYDRKNFTTSEELVRSGRYADRSWNSGIVFKPNRHFSVSYRASSGFRTPSFQELFGIDIYHDYPKGWQ
NgLbpA_WHO_M         628  GGRYDRKNFTTSEELVRSGRYADRSWNSGIVFKPNRHFSVSYRASSGFRTPSFQELFGIDIYHDYPKGWQ
NgLbpA_WHO_P         628  GGRYDRKNFTTSEELVRSGRYADRSWNSGIVFKPNRHFSVSYRASSGFRTPSFQELFGIDIYHDYPKGWQ
NgLbpA_WHO_U         628  GGRYDRKNFTTSEELVRSGRYADRSWNSGIVFKPNRHFSVSYRASSGFRTPSFQELFGIDIYHDYPKGWQ
NgLbpA_WHO_Y         628  GGRYDRKNFTTSEELVRSGRYADRSWNSGIVFKPNRHFSVSYRASSGFRTPSFQELFGIDIYHDYPKGWQ
NgLbpA_NCTC13798     628  GGRYDRKNFTTSEELVRSGRYADRSWNSGIVFKPNRHFSVSYRASSGFRTPSFQELFGIDIYHDYPKGWQ
NgLbpA_GCGS110       628  GGRYDRKNFTTSEELVRSGRYADRSWNSGIVFKPNRHFSVSYRASSGFRTPSFQELFGIDIYHDYPKGWQ
NgLbpA_WHO_O         628  GGRYDRKNFTTSEELVRSGRYADRSWNSGIVFKPNRHFSVSYRASSGFRTPSFQELFGIDIYHDYPKGWQ
NgLbpA_MS11          628  GGRYDRQNFTTSEELVRSGRYADRSWNSGIVFKPNRHFSVSYRASSGFRTPSFQELFGIDIYHDYPKGWQ
NgLbpA_WHO_G         628  GGRYDRQNFTTSEELVRSGRYADRSWNSGIVFKPNRHFSVSYRASSGFRTPSFQELFGIDIYHDYPKGWQ
NgLbpA_WHO_N         628  GGRYDRQNFTTSEELVRSGRYADRSWNSGIVFKPNRHFSVSYRASSGFRTPSFQELFGIDIYHDYPKGWQ
```

```
                              β30                 β31                 β32      β33        β34
NmLbpA_MC58

NmLbpA_MC58          698  RPALKSEKAANREIGLQWKGDFGFLEISSFRNRYTDMIAVADHKTKLPNQAGQLTEIDIRDYYNAQNMSL
NmLbpA_DE8555        700  RPALKSEKAANREIGLQWKGDFGFLEISSFRNRYTDMIAVADHKTKLPNQAGQLTEIDIRDYYNAQNMSL
NmLbpA_WUE2121       700  RPALKSEKAANREIGLQWKGDFGFLEISSFRNRYTDMIAVADHKTKLPNQAGQLTEIDIRDYYNAQNMSL
NmLbpA_M1080         694  RPVLKSEKAANREIGLQWKGDFGFLEISSFRNRYTDMIAVADHKTKLPDSTGRLTEIDIRDYYNAQNMSL
NmLbpA_P2396         694  RPALKSEKAANREIGLQWKGDFGFLEISSFRNRYTDMIAVADHKTQLPDSTGRLTEIDIRDYYNAQNMSL
NmLbpA_312901        694  RPALKSEKAANREIGLQWKGDFGFLEISSFRNRYTDMIAVADHKTKLPNQAGQLTEIDIRDYYNAQNMSL
NmLbpA_NCTC8249      700  RPALKSEKAANREIGLQWKGDFGFLEISSFRNRYTDMIAVADHKTQLPNQAGQLTEIDIRDYYNAQNMSL
NmLbpA_NGH36         696  RPALKSEKAANREIGLQWKGDFGFLEISSFRNRYTDMIAVADHKTKLPNQAGQLTEIDIRDYYNAQNMSL
NmLbpA_931905        695  RPALKSEKAANREIGLQWKGDFGFLEISSFRNRYTDMIAVADHKTKLPNQAGQLTEIDIRDYYNAQNMSL
NmLbpA_139M          695  RPALKSEKAANREIGLQWKGDFGFLEISSFRNRYTDMIAVADHKTKLPNQAGQLTEIDIRDYYNAQNMSL
NmLbpA_EG011         695  RPALKSEKAANREIGLQWKGDFGFLEISSFRNRYTDMIAVADHKTKLPNQAGQLTEIDIRDYYNAQNMSL
NmLbpA_88/03415      695  RPALKSEKAANREIGLQWKGDFGFLEISSFRNRYTDMIAVADHKTKLPNQAGQLTEIDIRDYYNAQNMSL
NmLbpA_020           698  RPALKSEKAANREIGLQWKGDFGFLEISSFRNRYTDMIAVADHKTKLPNQAGQLTEIDIRDYYNAQNMSL
NmLbpA_H44/76        695  RPALKSEKAANREIGLQWKGDFGFLEISSFRNRYTDMIAVADHKTKLPNQAGRLTEIDIRDYYNAQNMSL
NmLbpA_M0579         700  RPALKSEKAANREIGLQWKGDFGFLEISSFRNRYTDMIAVADHKTKLPNQAGQLTEIDIRDYYNAQNMSL
NmLbpA_G2136         695  RPALKSEKAANREIGLQWKGDFGFLEISSFRNRYTDMIAVADHKTKLPNQAGQLTEIDIRDYYNAQNMSL
NmLbpA_NM2811        700  RPALKSEKAANREIGLQWKGDFGFLEISSFRNRYTDMIAVADQKTKLPDSAGRLTEIDIRDYYNAQNMSL
NmLbpA_510612        700  RPALKSEKAANREIGLQWKGDFGFLEISSFRNRYTDMIAVADQKTKLPDSAGRLTEIDIRDYYNAQNMSL
NmLbpA_WUE2594       700  RPALKSEKAANREIGLQWKGDFGFLEISSFRNRYTDMIAVADQKTKLPDSAGRLTEIDIRDYYNAQNMSL
NmLbpA_M01-240013    699  RPALKSEKAANREIGLQWKGDFGFLEISSFRNRYTDMIAVADQKTKLPDSTGRLTEIDIRDYYNAQNMSL
NmLbpA_D8            695  RPALKSEKAANREIGLQWKGDFGFLEISSFRNRYTDMIAVADHKTKLPNQAGQLTEIDIRDYYNAQNMSL
NmLbpA_Z2491         699  RPALKSEKAANREIGLQWKGDFGFLEISSFRNRYTDMIAVADHKTQLPDSTGRLTEIDIRDYYNAQNMSL
NmLbpA_alpha704      699  RPALKSEKAANREIGLQWKGDFGFLEISSFRNRYTDMIAVADQKTKLPDSTGRLTEIDIRDYYNAQNMSL
NmLbpA_8013          700  RPALKSEKAANREIGLQWKGDFGFLEISSFRNRYTDMIAVADQKTKLPDSTGRLTEIDIRDYYNAQNMSL
NmLbpA_120M          694  RPALKSEKAANREIGLQWKGDFGFLEISSFRNRYTDMIAVADHKTKLPDSTGRLTEIDIRDYYNAQNMSL
NmLbpA_860800        694  RPALKSEKAANREIGLQWKGDFGFLEISSFRNRYTDMIAVADHKTQLPDSAGRLTEIDIRDYYNAQNMSL
NmLbpA_F1576         694  RPALKSEKAANREIGLQWKGDFGFLEISSFRNRYTDMIAVADQKTKLPDSTGRLTEIDIRDYYNAQNMSL
NmLbpA_M597          697  RPALKSEKAANREIGLQWKGDFGFLEISSFRNRYTDMIAVADHKTKLPNQAGQLTEIDIRDYYNAQNMSL
NmLbpA_MA-5756       698  RPALKSEKAANREIGLQWKGDFGFLEISSFRNRYTDMIAVADHKTKLPNQAGQLTEIDIRDYYNAQNMSL
NmLbpA_B16B6         698  RPALKSEKAANREIGLQWKGDFGFLEISSFRNRYTDMIAVADHKTKLPNQAGQLTEIDIRDYYNAQNMSL
NmLbpA_M978          694  RPALKSEKAANREIGLQWKGDFGFLEISSFRNRYTDMIAVADHKTKLPNQAGQLTEIDIRDYYNAQNMSL
NmLbpA_DNM2          698  RPALKSEKAANREIGLQWKGDFGFLEISSFRNRYTDMIAVADHKTKLPNQAGQLTEIDIRDYYNAQNMSL
NmLbpA_CU385         698  RPALKSEKAANREIGLQWKGDFGFLEISSFRNRYTDMIAVADHKTKLPNQAGQLTEIDIRDYYNAQNMSL
NmLbpA_NG144/82      694  RPALKSEKAANREIGLQWKGDFGFLEISSFRNRYTDMIAVADHKTKLPNQAGQLTEIDIRDYYNAQNMSL
NgLbpA_FA19          698  RPALKSEKAANREIGLQWKGDFGFLEISSFRNRYTDMIAVADQKTKLPDSAGRLTEIDIRDYYNAQNMSL
NgLbpA_WHO_M         698  RPALKSEKAANREIGLQWKGDFGFLEISSFRNRYTDMIAVADQKTKLPDSAGRLTEIDIRDYYNAQNMSL
NgLbpA_WHO_P         698  RPALKSEKAANREIGLQWKGDFGFLEISSFRNRYTDMIAVADQKTKLPDSAGRLTEIDIRDYYNAQNMSL
NgLbpA_WHO_U         698  RPALKSEKAANREIGLQWKGDFGFLEISSFRNRYTDMIAVADQKTKLPDSAGRLTEIDIRDYYNAQNMSL
NgLbpA_WHO_Y         698  RPALKSEKAANREIGLQWKGDFGFLEISSFRNRYTDMIAVADQKTKLPDSAGRLTEIDIRDYYNAQNMSL
NgLbpA_NCTC13798     698  RPALKSEKAANREIGLQWKGDFGFLEISSFRNRYTDMIAVADQKTKLPDSAGRLTEIDIRDYYNAQNMSL
NgLbpA_GCGS110       698  RPALKSEKAANREIGLQWKGDFGFLEISSFRNRYTDMIAVADQKTKLPDSAGRLTEIDIRDYYNAQNMSL
NgLbpA_WHO_O         698  RPALKSEKAANREIGLQWKGDFGFLEISSFRNRYTDMIAVADQKTKLPDSAGRLTEIDIRDYYNAQNMSL
NgLbpA_MS11          698  RPALKSEKAANREIGLQWKGDFGFLEISSFRNRYTDMIAVADQKTKLPDSAGRLTEIDIRDYYNAQNMSL
NgLbpA_WHO_G         698  RPALKSEKAANREIGLQWKGDFGFLEISSFRNRYTDMIAVADQKTKLPDSAGRLTEIDIRDYYNAQNMSL
NgLbpA_WHO_N         698  RPALKSEKAANREIGLQWKGDFGFLEISSFRNRYTDMIAVADQKTKLPDSAGRLTEIDIRDYYNAQNMSL
```

```
                                                β35                          β36    β37
NmLbpA_MC58                                                         TT                   TT

NmLbpA_MC58          768  QGVNILGKIDWNGVYGKLPEGLYTTLAYNRIKPKSVSNRPGLSLRSYALDAVQPSRYVLGFGYDQPEGKW
NmLbpA_DE8555        770  QGVNILGKIDWNGVYGKLPEGLYTTLAYNRIKPKSVSNRPGLSLRSYALDAVQPSRYVLGFGYDQPEGKW
NmLbpA_WUE2121       770  QGVNILGKIDWNGVYGKLPEGLYTTLAYNRIKPKSVSNRPGLSLRSYALDAVQPSRYVLGFGYDQPEGKW
NmLbpA_M1080         764  QGINILGKIDWNGVYGKLPEGLYTTLAYNRIKPKSVSNRPGLSLRSYALDAVQPSRYVLGFGYDQPEGKW
NmLbpA_P2396         764  QGVNILGKIDWNGVYGKLPEGLYTTLAYNRIKPKSVSNRPGLSLRSYALDAVQPSRYVLGFGYDQPEGKW
NmLbpA_312901        764  QGVNILGKIDWNGVYGKLPEGLYTTLAYNRIKPKSVSNRPGLSLRSYALDAVQPSRYVLGFGYDQPEGKW
NmLbpA_NCTC8249      770  QGVNILGKIDWNGVYGKLPEGLYTTLAYNRIKPKSVSNRPGLSLRSYALDAVQPSRYVLGFGYDQPEGKW
NmLbpA_NGH36         766  QGVNILGKIDWNGVYGKLPEGLYTTLAYNRIKPKSVSNRPGLSLRSYALDAVQPSRYVLGFGYDQPEGKW
NmLbpA_931905        765  QGVNILGKIDWNGVYGKLPEGLYTTLAYNRIKPKSVSNRPDLSLRSYALDAVQPSRYVLGFGYDQPEGKW
NmLbpA_139M          765  QGVNILGKIDWNGVYGKLPEGLYTTLAYNRIKPKSVSNRPGLSLRSYALDAVQPSRYVLGFGYDQPEGKW
NmLbpA_EG011         765  QGVNILGKIDWNGVYGKLPEGLYTTLAYNRIKPKSVSNRPGLSLRSYALDAVQPSRYVLGFGYDQPEGKW
NmLbpA_88/03415      765  QGVNILGKIDWNGVYGKLPEGLYTTLAYNRIKPKSVSNRPDLSLRSYALDAVQPSRYVLGFGYDQPEGKW
NmLbpA_020           768  QGVNILGKIDWNGVYGKLPEGLYTTLAYNRIKPKSVSNRPGLSLRSYALDAVQPSRYVLGFGYDQPEGKW
NmLbpA_H44/76        765  QGVNILGKIDWNGVYGKLPEGLYTTLAYNRIKPKSVSNRPDLSLRSYALDAGQPSRYVLGFGYDQPEGKW
NmLbpA_M0579         770  QGVNILGKIDWNGVYGKLPEGLYTTLAYNRIKPKSVSNRPGLSLRSYALDAVQPSRYVLGFGYDQPEGKW
NmLbpA_G2136         765  QGVNILGKIDWNGVYGKLPEGLYTTLAYNRIKPKSVSNRPDLSLRSYALDAVQPSRYVLGFGYDQPEGKW
NmLbpA_NM2811        770  QGINILGKIDWNGVYHKLPEGLYTTLAYNRIKPKSVSNRPGLSLRSYALDAVQPSRYVLGFGYDQPEGKW
NmLbpA_510612        770  QGINILGKIDWNGVYHKLPEGLYTTLAYNRIKPKSVSNRPGLSLRSYALDAVQPSRYVLGFGYDQPEGKW
NmLbpA_WUE2594       770  QGINILGKIDWNGVYHKLPEGLYTTLAYNRIKPKSVSNRPGLSLRSYALDAVQPSRYVLGFGYDQPEGKW
NmLbpA_M01-240013    769  QGINILGKIDWNGVYGKLPEGLYTTLAYNRIKPKSVSNRPGLSLRSYALDAVQPSRYVLGFGYDQPEGKW
NmLbpA_D8            765  QGINILGKIDWNGVYHKLPEGLYTTLAYNRIKPKSVSNRPGLSLRSYALDAVQPSRYVLGFGYDQPEGKW
NmLbpA_Z2491         769  QGVNILGKIDWNGVYGKLPEGLYTTLAYNRIKPKSVSNRPDLSLRSYALDAVQPSRYVLGFGYDQPEGKW
NmLbpA_alpha704      769  QGINILGKIDWNGVYGKLPEGLYTTLAYNRIKPKSVSNRPGLSLRSYALDAVQPSRYVLGFGYDQPEGKW
NmLbpA_8013          770  QGINILGKIDWNGVYGKLPEGLYTTLAYNRIKPKSVSNRPGLSLRSYALDAVQPSRYVLGFGYDQPEGKW
NmLbpA_120M          764  QGINILGKIDWNGVYGKLPEGLYTTLAYNRIKPKSVSNRPGLSLRSYALDAVQPSRYVLGFGYDQPEGKW
NmLbpA_860800        764  QGVNILGKIDWNGVYGKLPEGLYTTLAYNRIKPKSVSNRPGLSLRSYALDAVQPSRYVLGFGYDQPEGKW
NmLbpA_F1576         764  QGINILGKIDWNGVYGKLPEGLYTTLAYNRIKPKSVSNRPGLSLRSYALDAVQPSRYVLGFGYDQPEGKW
NmLbpA_M597          767  QGVNILGKIDWNGVYGKLPEGLYTTLAYNRIKPKSVSNRPGLSLRSYALDAVQPSRYVLGFGYDQPEGKW
NmLbpA_MA-5756       768  QGVNILGKIDWNGVYGKLPEGLYTTLAYNRIKPKSVSNRPGLSLRSYALDAVQPSRYVLGFGYDQPEGKW
NmLbpA_B16B6         768  QGVNILGKIDWNGVYGKLPEGLYTTLAYNRIKPKSVSNRPGLSLRSYALDAVQPSRYVLGFGYDQPEGKW
NmLbpA_M978          764  QGVNILGKIDWNGVYGKLPEGLYTTLAYNRIKPKSVSNRPGLSLRSYALDAVQPSRYVLGFGYDQPEGKW
NmLbpA_DNM2          768  QGVNILGKIDWNGVYGKLPEGLYTTLAYNRIKPKSVSNRPGLSLRSYALDAVQPSRYVLGFGYDQPEGKW
NmLbpA_CU385         768  QGVNILGKIDWNGMYGKLPEGLYTTLAYNRIKPKSVSNRPGLSLRSYALDAVQPSRYVLGFGYDQPEGKW
NmLbpA_NG144/82      764  QGVNILGKIDWNGVYGKLPEGLYTTLAYNRIKPKSVSNRPGLSLRSYALDAVQPSRYVLGFGYDQPEGKW
NgLbpA_FA19          768  QGINILGKIDWNGVYGKLPEGLYTTLAYNRIKPKSVSNRPDLSLRSYALDAVQPSRYVLGFGYDQPEGKW
NgLbpA_WHO_M         768  QGINILGKIDWNGVYGKLPEGLYTTLAYNRIKPKSVSNRPDLSLRSYALDAVQPSRYVLGFGYDQPEGKW
NgLbpA_WHO_P         768  QGINILGKIDWNGVYGKLPEGLYTTLAYNRIKPKSVSNRPDLSLRSYALDAVQPSRYVLGFGYDQPEGKW
NgLbpA_WHO_U         768  QGINILGKIDWNGVYGKLPEGLYTTLAYNRIKPKSVSNRPDLSLRSYALDAVQPSRYVLGFGYDQPEGKW
NgLbpA_WHO_Y         768  QGINILGKIDWNGVYGKLPEGLYTTLAYNRIKPKSVSNRPDLSLRSYALDAVQPSRYVLGFGYDQPEGKW
NgLbpA_NCTC13798     768  QGINILGKIDWNGVYGKLPEGLYTTLAYNRIKPKSVSNRPDLSLRSYALDAVQPSRYVLGFGYDQPEGKW
NgLbpA_GCGS110       768  QGINILGKIDWNGVYGKLPEGLYTTLAYNRIKPKSVSNRPDLSLRSYALDAVQPSRYVLGFGYDQPEGKW
NgLbpA_WHO_O         768  QGINILGKIDWNGVYGKLPEGLYTTLAYNRIKPKSVSNRPDLSLRSYALDAVQPSRYVLGFGYDQPEGKW
NgLbpA_MS11          768  QGINILGKIDWNGVYGKLPEGLYTTLAYNRIKPKSVSNRPDLSLRSYALDAVQPSRYVLGFGYDQPEGKW
NgLbpA_WHO_G         768  QGINILGKIDWNGVYGKLPEGLYTTLAYNRIKPKSVSNRPDLSLRSYALDAVQPSRYVLGFGYDQPEGKW
NgLbpA_WHO_N         768  QGINILGKIDWNGVYGKLPEGLYTTLAYNRIKPKSVSNRPDLSLRSYALDAVQPSRYVLGFGYDQPEGKW
```

```
                              β38                                        β39          TT  β40               α6
NmLbpA_MC58
NmLbpA_MC58          838  GANIMLTYSKGKNPDELAYLAGDQKRYSTKRASSSWSTADVSAYLNLKKRLTLRAAIYNIGNYRYVTWES
NmLbpA_DE8555        840  GANIMLTYSKGKNPDELAYLAGDQKRYSTKRASSSWSTADVSAYLNLKKRLTLRAAIYNIGNYRYVTWES
NmLbpA_WUE2121       840  GANIMLTYSKGKNPDELAYLAGDQKRYSTKRASSSWSTADVSAYLNLKKRLTLRAAIYNIGNYRYVTWES
NmLbpA_M1080         834  GANIMLTYSKGKNPDELAYLAGDQKRYSTKRASSSWSTADVSAYLNLKKRLTLRAAIYNIGNYRYVTWES
NmLbpA_P2396         834  GANIMLTYSKGKNPDELAYLAGDQKRYSTKRASSSWSTADVSAYLNLKKRLTLRAAIYNIGNYRYVTWES
NmLbpA_312901        834  GANIMLTYSKGKNPDELAYLAGDQKRYSTKRASSSWSTADVSAYLNLKKRLTLRAAIYNIGNYRYVTWES
NmLbpA_NCTC8249      840  GANIMLTYSKGKNPDELAYLAGDQKRYSTKRASSSWSTADVSAYLNLKKRLTLRAAIYNIGNYRYVTWES
NmLbpA_NGH36         836  GANIMLTYSKGKNPDELAYLAGDQKRYSTKRASSSWSTADVSAYLNLKKRLTLRAAIYNIGNYRYVTWES
NmLbpA_931905        835  GANIMLTYSKGKNPDELAYLAGDQKRYSTKRASSSWSTADVSAYLNLKKRLTLRAAIYNIGNYRYVTWES
NmLbpA_139M          835  GANIMLTYSKGKNPDELAYLAGDQKRYSTKRASSSWSTADVSAYLNLKKRLTLRAAIYNIGNYRYVTWES
NmLbpA_EG011         835  GANIMLTYSKGKNPDELAYLAGDQKRYSTKRASSSWSTADVSAYLNLKKRLTLRAAIYNIGNYRYVTWES
NmLbpA_88/03415      835  GANIMLTYSKGKNPDELAYLAGDQKRYSTKRASSSWSTADVSAYLNLKKRLTLRAAIYNIGNYRYVTWES
NmLbpA_020           838  GANIMLTYSKGKNPDELAYLAGDQKRYSTKRASSSWSTADVSAYLNLKKRLTLRAAIYNIGNYRYVTWES
NmLbpA_H44/76        835  GANIMLTYSKGKNPDELAYLAGDQKRYSTKRASSSWSTADVSAYLNLKKRLTLRAAIYNIGNYRYVTWES
NmLbpA_M0579         840  GANIMLTYSKGKNPDELAYLAGDQKRYSTKRASSSWSTADVSAYLNLKKRLTLRAAIYNIGNYRYVTWES
NmLbpA_G2136         835  GANIMLTYSKGKNPDELAYLAGDQKRYSTKRASSSWSTADVSAYLNLKKRLTLRAAIYNIGNYRYVTWES
NmLbpA_NM2811        840  GANIMLTYSKGKNPDELAYLAGDQKRYSAKRASSSWSTADVSAYLNLKKRLTLRAGIYNIGNYRYVTWES
NmLbpA_510612        840  GANIMLTYSKGKNPDELAYLAGDQKRYSAKRASSSWSTADVSAYLNLKKRLTLRAGIYNIGNYRYVTWES
NmLbpA_WUE2594       840  GANIMLTYSKGKNPDELAYLAGDQKRYSAKRASSSWSTADVSAYLNLKKRLTLRAGIYNIGNYRYVTWES
NmLbpA_M01-240013    839  GANIMLTYSKGKNPDELAYLAGDQKRYSTKRASSSWSTADVSAYLNLKKRLTLRAAIYNIGNYRYVTWES
NmLbpA_D8            835  GANIMLTYSKGKNPDELAYLAGDQKRYSAGRVTSSWKTADVSAYLNLKKRLTLRAAIYNIGNYRYVTWES
NmLbpA_Z2491         839  GANIMLTYSKGKNPDELAYLAGDQKRYSTKRASSSWSTADVSAYLNLKKRLTLRAAIYNIGNYRYVTWES
NmLbpA_alpha704      839  GANIMLTYSKGKNPDELAYLAGDQKRYSAKRASSSWSTADVSAYLNLKKRLTLRAAIYNIGNYRYVTWES
NmLbpA_8013          840  GANIMLTYSKGKNPDELAYLAGDQKRYSTKRASSSWSTADVSAYLNLKKRLTLRAAIYNIGNYRYVTWES
NmLbpA_120M          834  GANIMLTYSKGKNPDELAYLAGDQKRYSTKRASSSWSTADVSAYLNLKKRLTLRAAIYNIGNYRYVTWES
NmLbpA_860800        834  GANIMLTYSKGKNPDELAYLAGDQKRYSTKRASSSWSTADVSAYLNLKKRLTLRAAIYNIGNYRYVTWES
NmLbpA_F1576         834  GANIMLTYSKGKNPDELAYLAGDQKRYSTKRASSSWSTADVSAYLNLKKRLTLRAAIYNIGNYRYVTWES
NmLbpA_M597          837  GANIMLTYSKGKNPDELAYLAGDQKRYSTKRASSSWSTADVSAYLNLKKRLTLRAAIYNIGNYRYVTWES
NmLbpA_MA-5756       838  GANIMLTYSKGKNPDELAYLAGDQKRYSTKRASSSWSTADVSAYLNLKKRLTLRAAIYNIGNYRYVTWES
NmLbpA_B16B6         838  GANIMLTYSKGKNPDELAYLAGDQKRYSTKRASSSWSTADVSAYLNLKKRLTLRAAIYNIGNYRYVTWES
NmLbpA_M978          834  GANIMLTYSKGKNPDELAYLAGDQKRYSTKRASSSWSTADVSAYLNLKKRLTLRAAIYNIGNYRYVTWES
NmLbpA_DNM2          838  GANIMLTYSKGKNPDELAYLAGDQKRYSTKRASSSWSTADVSAYLNLKKRLTLRAAIYNIGNYRYVTWES
NmLbpA_CU385         838  GANIMLTYSKGKNPDELAYLAGDQKRYSTKRASSSWSTADVSAYLNLKKRLTLRAAIYNIGNYRYVTWES
NmLbpA_NG144/82      834  GANIMLTYSKGKNPDELAYLAGDQKRYSTKRASSSWSTADVSAYLNLKKRLTLRAAIYNIGNYRYVTWES
NgLbpA_FA19          838  GANIMLTYSKGKNPDELAYLAGDQKRYSAGRVTSSWKTADVSAYLNLKKRLTLRAAIYNIGNYRYVTWES
NgLbpA_WHO_M         838  GANIMLTYSKGKNPDELAYLAGDQKRYSAGRVTSSWKTADVSAYLNLKKRLTLRAAIYNIGNYRYVTWES
NgLbpA_WHO_P         838  GANIMLTYSKGKNPDELAYLAGDQKRYSAGRVTSSWKTADVSAYLNLKKRLTLRAAIYNIGNYRYVTWES
NgLbpA_WHO_U         838  GANIMLTYSKGKNPDELAYLAGDQKRYSAGRVTSSWKTADVSAYLNLKKRLTLRAAIYNIGNYRYVTWES
NgLbpA_WHO_Y         838  GANIMLTYSKGKNPDELAYLAGDQKRYSAGRVTSSWKTADVSAYLNLKKRLTLRAAIYNIGNYRYVTWES
NgLbpA_NCTC13798     838  GANIMLTYSKGKNPDELAYLAGDQKRYSAGRVTSSWKTADVSAYLNLKKRLTLRAAIYNIGNYRYVTWES
NgLbpA_GCGS110       838  GANIMLTYSKGKNPDELAYLAGDQKRYSAGRVTSSWKTADVSAYLNLKKRLTLRAAIYNIGNYRYVTWES
NgLbpA_WHO_O         838  GANIMLTYSKGKNPDELAYLAGDQKRYSAGRVTSSWKTADVSAYLNLKKRLTLRAAIYNIGNYRYVTWES
NgLbpA_MS11          838  GANIMLTYSKGKNPDELAYLAGDQKRYSAGRVTSSWKTADVSAYLNLKKRLTLRAAIYNIGNYRYVTWES
NgLbpA_WHO_G         838  GANIMLTYSKGKNPDELAYLAGDQKRYSAGRVTSSWKTADVSAYLNLKKRLTLRAAIYNIGNYRYVTWES
NgLbpA_WHO_N         838  GANIMLTYSKGKNPDELAYLAGDQKRYSAGRVTSSWKTADVSAYLNLKKRLTLRAAIYNIGNYRYVTWES
```

```
                          η5     TT         TT     β41
NmLbpA_MC58
NmLbpA_MC58          908  LRQTAESTANRHGGDSNYGRYAAPGRNFSLALEMKF
NmLbpA_DE8555        910  LRQTAESTANRHGGDSNYGRYAAPGRNFSLALEMKF
NmLbpA_WUE2121       910  LRQTAESTANRHGGDSNYGRYAAPGRNFSLALEMKF
NmLbpA_M1080         904  LRQTAESTANRHGGDSNYGRYAAPGRNFSLALEMKF
NmLbpA_P2396         904  LRQTAESTANRHGGDSNYGRYAAPGRNFSLALEMKF
NmLbpA_312901        904  LRQTAESTANRHGGDSNYGRYAAPGRNFSLALEMKF
NmLbpA_NCTC8249      910  LRQTAESTANRHGGDSNYGRYAAPGRNFSLALEMKF
NmLbpA_NGH36         906  LRQTAESTANRHGGDSNYGRYAAPGRNFSLALEMKF
NmLbpA_931905        905  LRQTAESTANRHGGDSNYGRYAAPGRNFSLALEMKF
NmLbpA_139M          905  LRQTAESTANRHGGDSNYGRYAAPGRNFSLALEMKF
NmLbpA_EG011         905  LRQTAESTANRHGGDSNYGRYAAPGRNFSLALEMKF
NmLbpA_88/03415      905  LRQTAESTANRHGGDSNYGRYAAPGRNFSLALEMKF
NmLbpA_020           908  LRQTAESTANRHGGDSNYGRYAAPGRNFSLALEMKF
NmLbpA_H44/76        905  LRQTAESTANRHGGDSNYGRYAAPGRNFSLALEMKF
NmLbpA_M0579         910  LRQTAESTANRHGGDSNYGRYAAPGRNFSLALEMKF
NmLbpA_G2136         905  LRQTAESTANRHGGDSNYGRYAAPGRNFSLALEMKF
NmLbpA_NM2811        910  LRQTAEGTANRHGGDSNYGRYAAPGRNFSLALEMKF
NmLbpA_510612        910  LRQTAEGTANRHGGDSNYGRYAAPGRNFSLALEMKF
NmLbpA_WUE2594       910  LRQTAEGTANRHGGDSNYGRYAAPGRNFSLALEMKF
NmLbpA_M01-240013    909  LRQTAESTANRHGGDSNYGRYAAPGRNFSLALEMKF
NmLbpA_D8            905  LRQTAESTANRHGGDSNYGRYAAPGRNFSLALEMKF
NmLbpA_Z2491         909  LRQTAESTANRHGGDSNYGRYAAPGRNFSLALEMKF
NmLbpA_alpha704      909  LRQTAESTANRHGGDSNYGRYAAPGRNFSLALEMKF
NmLbpA_8013          910  LRQTAESTANRHGGDSNYGRYAAPGRNFSLALEMKF
NmLbpA_120M          904  LRQTAESTANRHGGDSNYGRYAAPGRNFSLALEMKF
NmLbpA_860800        904  LRQTAESTANRHGGDSNYGRYAAPGRNFSLALEMKF
NmLbpA_F1576         904  LRQTAESTANRHGGDSNYGRYAAPGRNFSLALEMKF
NmLbpA_M597          907  LRQTAESTANRHGGDSNYGRYAAPGRNFSLALEMKF
NmLbpA_MA-5756       908  LRQTAESTANRHGGDSNYGRYAAPGRNFSLALEMKF
NmLbpA_B16B6         908  LRQTAESTANRHGGDSNYGRYAAPGRNFSL......
NmLbpA_M978          904  LRQTAESTANRHGGDSNYGRYAAPGRNFSLALEMKF
NmLbpA_DNM2          908  LRQTAESTANRHGGDSNYGRYAAPGRNFSLALEMKF
NmLbpA_CU385         908  LRQTAESTANRHGGDSNYGRYAAPGRNFSLALEMKF
NmLbpA_NG144/82      904  LRQTAESTANRHGGDSNYGRYAAPGRNFSLALEMKF
NgLbpA_FA19          908  LRQTAESTANRHGGDSNYGRYAAPGRNFSLALEMKF
NgLbpA_WHO_M         908  LRQTAESTANRHGGDSNYGRYAAPGRNFSLALEMKF
NgLbpA_WHO_P         908  LRQTAESTANRHGGDSNYGRYAAPGRNFSLALEMKF
NgLbpA_WHO_U         908  LRQTAESTANRHGGDSNYGRYAAPGRNFSLALEMKF
NgLbpA_WHO_Y         908  LRQTAESTANRHGGDSNYGRYAAPGRNFSLALEMKF
NgLbpA_NCTC13798     908  LRQTAESTANRHGGDSNYGRYAAPGRNFSLALEMKF
NgLbpA_GCGS110       908  LRQTAESTANRHGGDSNYGRYAAPGRNFSLALEMKF
NgLbpA_WHO_O         908  LRQTAESTANRHGGDSNYGRYAAPGRNFSLALEMKF
NgLbpA_MS11          908  LRQTAESTANRHGGDSNYGRYAAPGRNFSLALEMKF
NgLbpA_WHO_G         908  LRQTAESTANRHGGDSNYGRYAAPGRNFSLALEMKF
NgLbpA_WHO_N         908  LRQTAESTANRHGGDSNYGRYAAPGRNFSLALEMKF
```

**Supplementary Figure 4. Sequence alignment of LbpBs.**

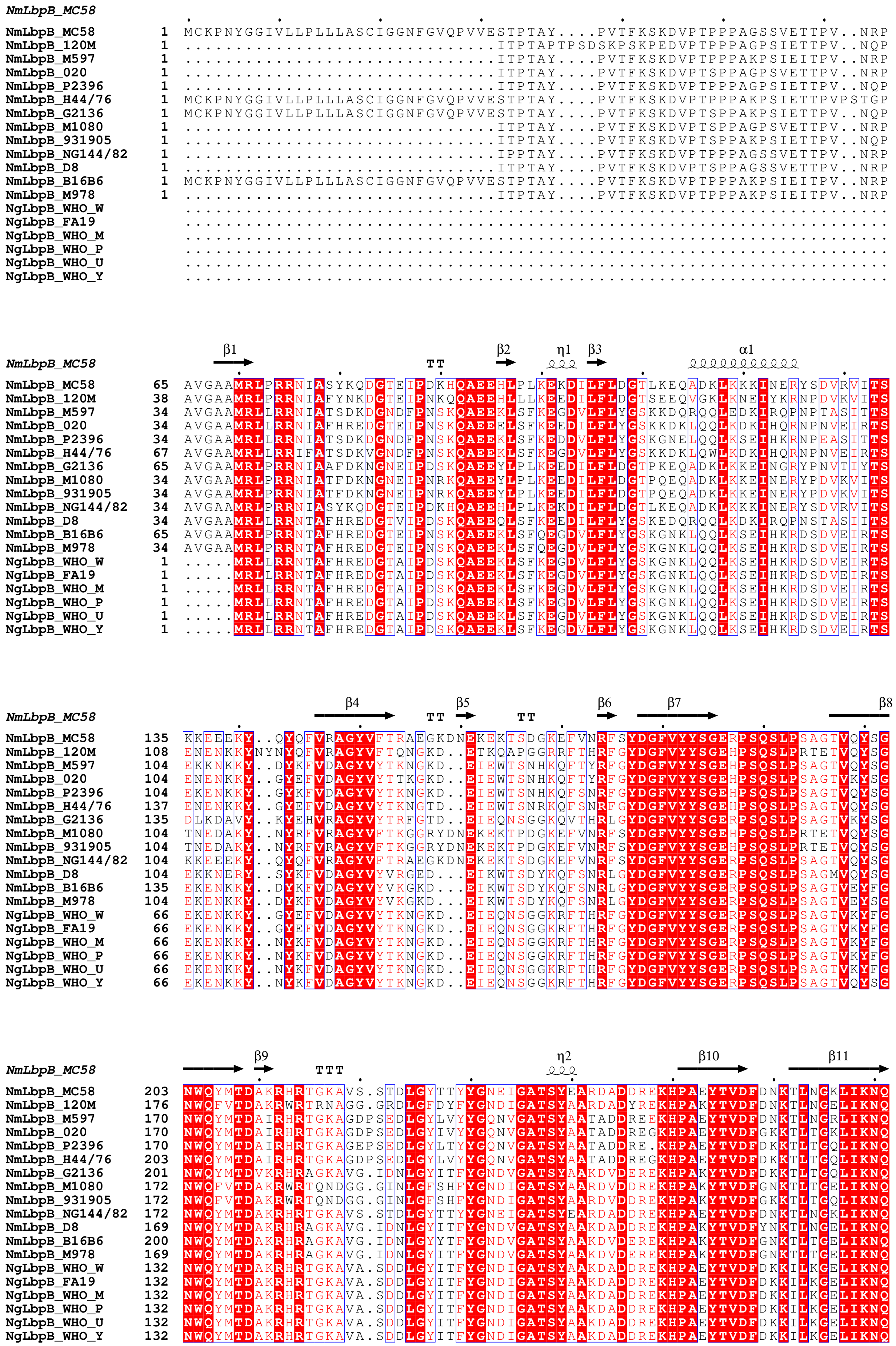

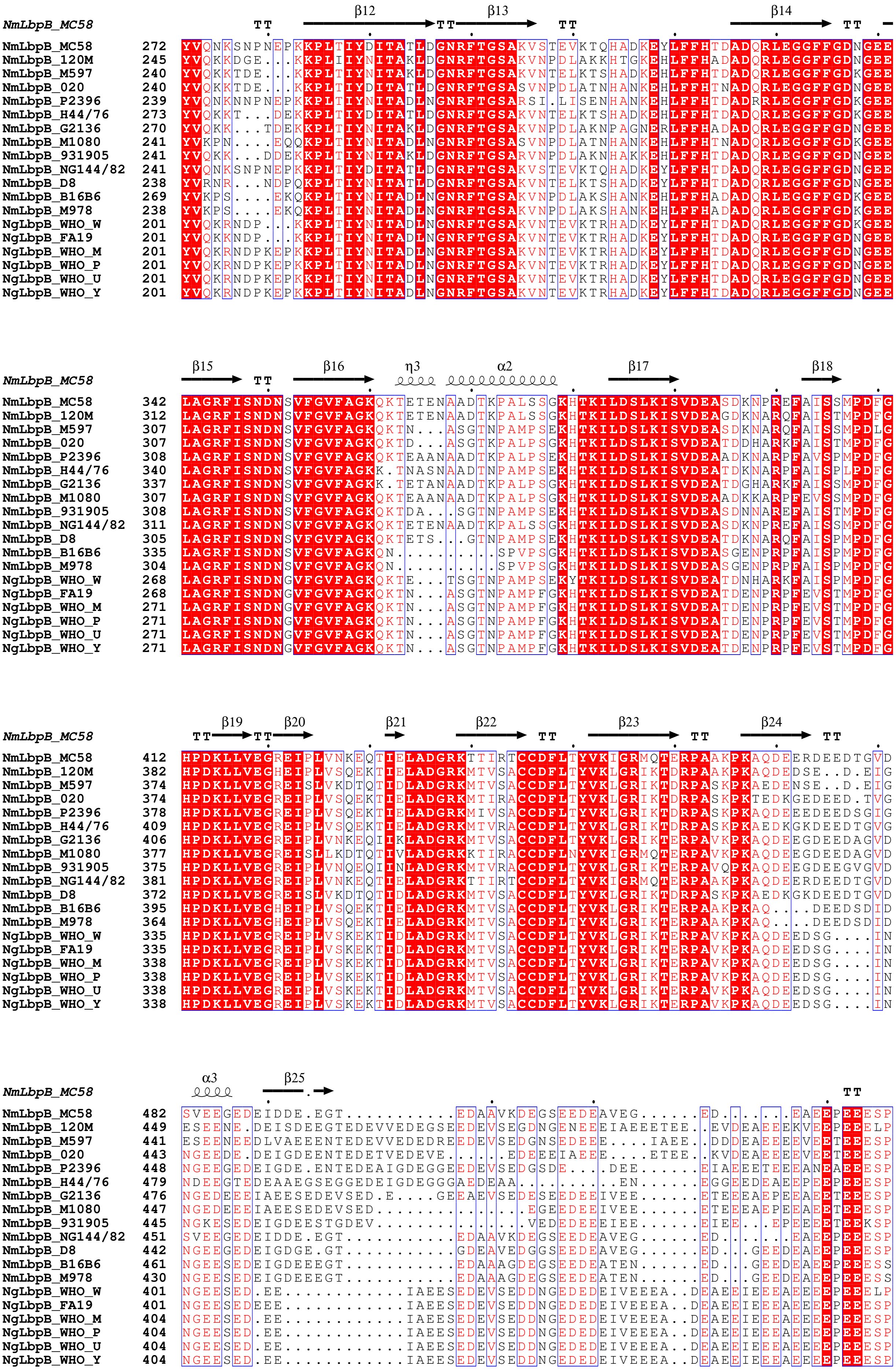
NmLbpB_MC58
β12 β13 β14 TT TT TT TT
NmLbpB_MC58 272 YVQNKSNPNEPKKPLTIYDITATLDGNRFTGSAKVSTEVKTQHADKEYLFFHTDADQRLEGGFFGDNGEE
NmLbpB_120M 245 YVQKKDGE...KKPLIIYNITAKLDGNRFTGSAKVNPDLAKKHTGKEHLFFHADADQRLEGGFFGDNGEE
NmLbpB_M597 240 YVQKKTDE...KKPLTIYNITAKLDGNRFTGSAKVNTELKTSHADKEHLFFHTDADQRLEGGFFGDKGEE
NmLbpB_020 240 YVQKKTDE...KKPLTIYDITATLDGNRFTGSASVNPDLATNHANKEHLFFHTNADQRLEGGFFGDNGEE
NmLbpB_P2396 239 YVQNKNNPNEPKKPLTIYNITADLNGNRFTGSARSI.LISENHANKEHLFFHTDADRRLEGGFFGDKGEE
NmLbpB_H44/76 273 YVQKKT...DEKKPLTIYDITATLDGNRFTGSAKVNTELKTSHADKEHLFFHTDADQRLEGGFFGDKGEE
NmLbpB_G2136 270 YVQKK...TDEKKPLTIYNITAKLDGNRFTGSAKVNPDLAKNPAGNERLFFHADADQRLEGGFFGDNGEE
NmLbpB_M1080 241 YVKPN....EQQKPLTIYNITADLNGNRFTGSASVNPDLATNHANKEHLFFHTNADQRLEGGFFGDKGEE
NmLbpB_931905 241 YVQKK...DDEKKPLTIYNITAKLDGNRFTGSARVNPDLAKNHAKKEHLFFHTDADQRLEGGFFGDNGEE
NmLbpB_NG144/82 241 YVQNKSNPNEPKKPLTIYDITATLDGNRFTGSAKVSTEVKTQHADKEYLFFHTDADQRLEGGFFGDNGEE
NmLbpB_D8 238 YVRNR...NDPQKPLTIYNITATLDGNRFTGSAKVNTELKTSHADKEYLFFHTDADQRLEGGFFGDNGEE
NmLbpB_B16B6 269 YVKPS....EKQKPLTIYNITADLNGNRFTGSAKVNPDLAKSHANKEHLFFHADADQRLEGGFFGDKGEE
NmLbpB_M978 238 YVKPS....EKQKPLTIYNITADLNGNRFTGSAKVNPDLAKSHANKEHLFFHADADQRLEGGFFGDKGEE
NgLbpB_WHO_W 201 YVQKRNDP...KKPLTIYNITADLNGNRFTGSAKVNTEVKTRHADKEYLFFHTDADQRLEGGFFGDNGEE
NgLbpB_FA19 201 YVQKKNDP...KKPLTIYNITADLNGNRFTGSAKVNTEVKTRHADKEYLFFHTDADQRLEGGFFGDNGEE
NgLbpB_WHO_M 201 YVQKRNDPKEPKKPLTIYNITADLNGNRFTGSAKVNTEVKTRHADKEYLFFHTDADQRLEGGFFGDNGEE
NgLbpB_WHO_P 201 YVQKRNDPKEPKKPLTIYNITADLNGNRFTGSAKVNTEVKTRHADKEYLFFHTDADQRLEGGFFGDNGEE
NgLbpB_WHO_U 201 YVQKRNDPKEPKKPLTIYNITADLNGNRFTGSAKVNTEVKTRHADKEYLFFHTDADQRLEGGFFGDNGEE
NgLbpB_WHO_Y 201 YVQKRNDPKEPKKPLTIYNITADLNGNRFTGSAKVNTEVKTRHADKEYLFFHTDADQRLEGGFFGDNGEE
NmLbpB_MC58
β15 β16 η3 α2 β17 β18 TT
NmLbpB_MC58 342 LAGRFISNDNSVFGVFAGKQKTETENAADTKPALSSGKHTKILDSLKISVDEASDKNPREFAISSMPDFG
NmLbpB_120M 312 LAGRFISNDNSVFGVFAGKQKTETENAADTKPALSSGKHTKILDSLKISVDEAGDKNARQFAISTMPDFG
NmLbpB_M597 307 LAGRFISNDNSVFGVFAGKQKTN...ASGTNPAMPSEKHTKILDSLKISVDEASDKNARQFAISSMPDLG
NmLbpB_020 307 LAGRFISNDNSVFGVFAGKQKTD...ASGTKPALPSGKHTKILDSLKISVDEATDDHARKFAISTMPDFG
NmLbpB_P2396 308 LAGRFISNDNSVFGVFAGKQKTEAANAADTKPALPSEKHTKILDSLKISVDEAADKNARPFAVSPMPDFG
NmLbpB_H44/76 340 LAGRFISNDNSVFGVFAGKK.TNASNAADTNPAMPSEKHTKILDSLKISVDEATDKNARPFAISPLPDFG
NmLbpB_G2136 337 LAGRFISNDNSVFGVFAGKK.TETANAADTKPALPSGKHTKILDSLKISVDEATDGHARKFAISSMPDFG
NmLbpB_M1080 307 LAGRFISNDNSVFGVFAGKQKTEAANAADTKPALPSGKHTKILDSLKISVDEAADKKARPFEVSSMPDFG
NmLbpB_931905 308 LAGRFISNDNSVFGVFAGKQKTDA...SGTNPAMPSEKHTKILDSLKISVDEASDNNAREFAISTMPDFG
NmLbpB_NG144/82 311 LAGRFISNDNSVFGVFAGKQKTETENAADTKPALSSGKHTKILDSLKISVDEASDKNPREFAISSMPDFG
NmLbpB_D8 305 LAGRFISNDNSVFGVFAGKQKTETS...GTNPAMPSEKHTKILDSLKISVDEATDKNARQFAISPMPDFG
NmLbpB_B16B6 335 LAGRFISNDNSVFGVFAGKQN..........SPVPSGKHTKILDSLKISVDEASGENPRPFAISPMPDFG
NmLbpB_M978 304 LAGRFISNDNSVFGVFAGKQN..........SPVPSGKHTKILDSLKISVDEASGENPRPFAISPMPDFG
NgLbpB_WHO_W 268 LAGRFISNDNGVFGVFAGKQKTE...TSGTNPAMPSEKYTKILDSLKISVDEATDNHARKFAISPMPDFG
NgLbpB_FA19 268 LAGRFISNDNGVFGVFAGKQKTN...ASGTNPAMPFGKHTKILDSLKISVDEATDENPRPFEVSTMPDFG
NgLbpB_WHO_M 271 LAGRFISNDNGVFGVFAGKQKTN...ASGTNPAMPFGKHTKILDSLKISVDEATDENPRPFEVSTMPDFG
NgLbpB_WHO_P 271 LAGRFISNDNGVFGVFAGKQKTN...ASGTNPAMPFGKHTKILDSLKISVDEATDENPRPFEVSTMPDFG
NgLbpB_WHO_U 271 LAGRFISNDNGVFGVFAGKQKTN...ASGTNPAMPFGKHTKILDSLKISVDEATDENPRPFEVSTMPDFG
NgLbpB_WHO_Y 271 LAGRFISNDNGVFGVFAGKQKTN...ASGTNPAMPFGKHTKILDSLKISVDEATDENPRPFEVSTMPDFG
NmLbpB_MC58
β19 β20 β21 β22 β23 β24 TT TT TT TT TT
NmLbpB_MC58 412 HPDKLLVEGREIPLVNKEQTIELADGRKTTIRTCCDFLTYVKIGRMQTERPAAKPKAQDEERDEEDTGVD
NmLbpB_120M 382 HPDKLLVEGHEIPLVSQEKTIELADGRKMTVSACCDFLTYVKLGRIKTDRPAAKPKAQDEDSE..D.EIG
NmLbpB_M597 374 HPDKLLVEGREISLVKDTQTIDLADGRKMIVSACCDFLTYVKLGRIKTDRPASKPKAEDENSE..D.EIG
NmLbpB_020 374 HPDKLLVEGREIPLVSQEKTIELADGRKMTIRACCDFLTYVKLGRIKTDRPASKPKTEDKGEDEED.TVG
NmLbpB_P2396 378 HPDKLLVEGREIPLVSQEKTIELADGRKMTVRACCDFLTYVKLGRIKTDRPASKPKAQDEEEDEEDSGIG
NmLbpB_H44/76 409 HPDKLLVEGREIPLVSQEKTIELADGRKMTVRACCDFLTYVKLGRIKTDRPASKPKAEDKGKDEEDTGVG
NmLbpB_G2136 406 HPDKLLVEGREIPLVNKEQIIKLADGRKMTVSACCDFLTYVKLGRIKTERPAVKPKAQDEEGDEEDAGVD
NmLbpB_M1080 377 HPDKLLVEGREISLLKDTQTIVLADGRKKTIRACCDFLNYVKIGRMQTERPAVKPKAQDEEGDEEDAGVD
NmLbpB_931905 375 HPDKLLVEGREIPLVSQEKTINLADGRKMTVRACCDFLTYVKLGRIKTERPAVQPKAQDEEGDEEGVGVD
NmLbpB_NG144/82 381 HPDKLLVEGREIPLVNKEQTIELADGRKTTIRTCCDFLTYVKIGRMQTERPAAKPKAQDEERDEEDTGVD
NmLbpB_D8 372 HPDKLLVEGREISLVKDTQTIDLADGRKMTVSACCDFLTYVKLGRIKTDRPASKPKAEDKGKDEEDTGVD
NmLbpB_B16B6 395 HPDKLLVEGHEIPLVSQEKTIELADGRKMTVSACCDFLTYVKLGRIKTERPAAKPKAQ...DEEDSDID
NmLbpB_M978 364 HPDKLLVEGHEIPLVSQEKTIELADGRKMTVSACCDFLTYVKLGRIKTERPAAKPKAQ...DEEDSDID
NgLbpB_WHO_W 335 HPDKLLVEGREIPLVSKEKTIDLADGRKMTVSACCDFLTYVKLGRIKTERPAVKPKAQDEEDSG....IN
NgLbpB_FA19 335 HPDKLLVEGREIPLVSKEKTIDLADGRKMTVSACCDFLTYVKLGRIKTERPAVKPKAQDEEDSG....IN
NgLbpB_WHO_M 338 HPDKLLVEGREIPLVSKEKTIDLADGRKMTVSACCDFLTYVKLGRIKTERPAVKPKAQDEEDSG....IN
NgLbpB_WHO_P 338 HPDKLLVEGREIPLVSKEKTIDLADGRKMTVSACCDFLTYVKLGRIKTERPAVKPKAQDEEDSG....IN
NgLbpB_WHO_U 338 HPDKLLVEGREIPLVSKEKTIDLADGRKMTVSACCDFLTYVKLGRIKTERPAVKPKAQDEEDSG....IN
NgLbpB_WHO_Y 338 HPDKLLVEGREIPLVSKEKTIDLADGRKMTVSACCDFLTYVKLGRIKTERPAVKPKAQDEEDSG....IN
NmLbpB_MC58
α3 β25 TT
NmLbpB_MC58 482 SVEEGEDEIDDE.EGT...........EDAAVKDEGSEEDEAVEG......ED......EAEEPEEESP
NmLbpB_120M 449 ESEENE.DEISDDEEGTEDEVVEDEGSEEDEVSEGDNGENEEEIAEEETEE..EVDEAEEEKVEEPEEELP
NmLbpB_M597 441 ESEENEEDLVAEEENTEDEVVEDEDREEDEVSEDGNSEDEEE....IAEE..DDDEAEEEEVEETEEESP
NmLbpB_020 443 NGEEDE.DEIGDEENTEDETVEDEVE......EDEEEIAEEE....ETEE..KVDEAEEEEVEETEEESP
NmLbpB_P2396 448 NGEEGEDEIGDE.ENTEDEAIGDEGGEEDEVSEDGSDE....DEE......EIAEEETEEEANEAEEESP
NmLbpB_H44/76 479 NDEEGTEDEAAEGSEGGEDEIGDEGGGAEDEAA..........EN......EGGEEDEAEEPEEPEEESP
NmLbpB_G2136 476 NGEDEEEIAEESEDEVSED.E....GEEAEVSEDESEEDEEIVEE......ETEEEAEPEEAEETEEESP
NmLbpB_M1080 447 NGEDEEEIAEESEDEVSED..............EGEEDEEIVEE......ETEEEAEPEEAEETEEESP
NmLbpB_931905 445 NGKESEDEIGDEESTGDEV...............VEDEDEEEIEE......EIEE...EPEEEETEEKSP
NmLbpB_NG144/82 451 SVEEGEDEIDDE.EGT...........EDAAVKDEGSEEDEAVEG......ED......EAEEPEEESP
NmLbpB_D8 442 NGEEGEDEIGDGE.GT...........GDEAVEDGGSEEDEAVEG......ED...GEEDEAEEPEEESP
NmLbpB_B16B6 461 NGEESEDEIGDEEEGT...........EDAAAGDEGSEEDEATEN......ED...GEEDEAEEPEEESS
NmLbpB_M978 430 NGEESEDEIGDEEEGT...........EDAAAGDEGSEEDEATEN......ED...GEEDEAEEPEEESS
NgLbpB_WHO_W 401 NGEESED.EE............IAEESEDEVSEDDNGEDEDEIVEEEA..DEAEEIEEEAEEEEPEEELP
NgLbpB_FA19 401 NGEESEDEEE............IAEESEDEVSEDDNGEDEDEIVEEEA..DEAEEIEEEAEEEEPEEESP
NgLbpB_WHO_M 404 NGEESED.EE............IAEESEDEVSEDDNGEDEDEIVEEEA..DEAEEIEEEAEEEEPEEESP
NgLbpB_WHO_P 404 NGEESED.EE............IAEESEDEVSEDDNGEDEDEIVEEEA..DEAEEIEEEAEEEEPEEESP
NgLbpB_WHO_U 404 NGEESED.EE............IAEESEDEVSEDDNGEDEDEIVEEEA..DEAEEIEEEAEEEEPEEESP
NgLbpB_WHO_Y 404 NGEESED.EE............IAEESEDEVSEDDNGEDEDEIVEEEA..DEAEEIEEEAEEEEPEEESP

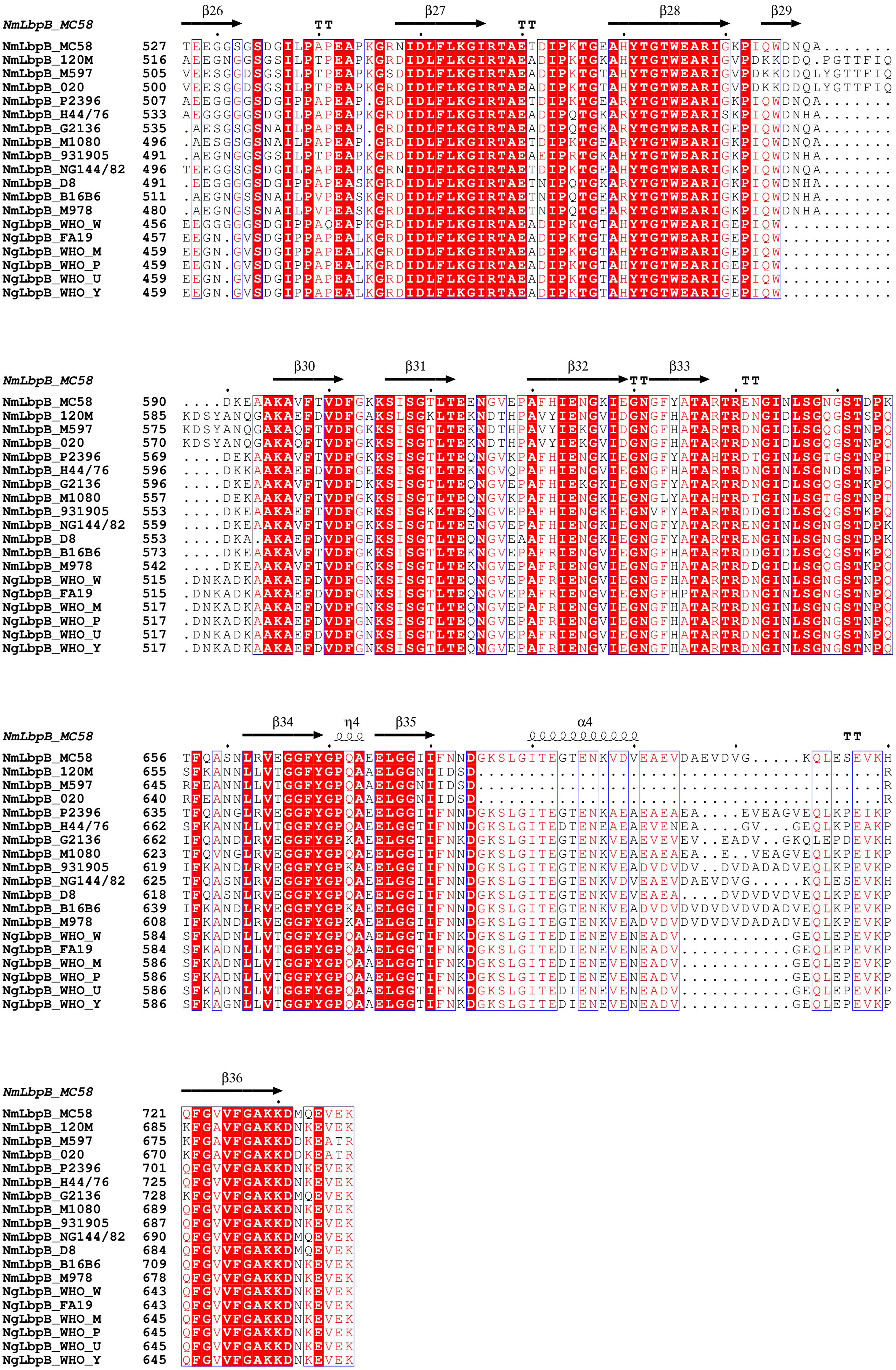
NmLbpB_MC58
β26
β27
β28
β29
β30
β31
β32
β33
β34
η4
β35
α4
β36
NmLbpB_MC58 527
NmLbpB_120M 516
NmLbpB_M597 505
NmLbpB_020 500
NmLbpB_P2396 507
NmLbpB_H44/76 533
NmLbpB_G2136 535
NmLbpB_M1080 496
NmLbpB_931905 491
NmLbpB_NG144/82 496
NmLbpB_D8 491
NmLbpB_B16B6 511
NmLbpB_M978 480
NgLbpB_WHO_W 456
NgLbpB_FA19 457
NgLbpB_WHO_M 459
NgLbpB_WHO_P 459
NgLbpB_WHO_U 459
NgLbpB_WHO_Y 459

# Supplementary Figure 5. Sequence alignment of FrpBs.

*NmFrpB_F3-3*: TT β1 α1 α2

```
NmFrpB_F3-3              1  .....................MAENNAKVVLDTVTVKGDRQGSKIRTNIVTLQQKDESTATDMRELLKEE
NmFrpB_alpha522          1  MNAPFFRLSLLSLTLAAGFAHAAENNANVALDTVTVKGDRQGSKIRTNIVTLQQKDESTATDMRELLKEE
NmFrpB_alpha275          1  MNAPFFRLSLLSLTLAAGFAHAAENNAKAVLDTITVKGDRQGSKIRTNIVTLQQKDESTATDMRELLKEE
NmFrpB_053442            1  MNAPLFRLSLLSLTLAAGFAHAAENNAKVVLDTVTVKGDRQGSKIRTNIVTLQQKDESTATDMRELLKEE
NmFrpB_H44/76            1  MNTPLFRLSLLSLTLAAGFAHAAENNAKVVLDTVTVKGDRQGSKIRTNIVTLQQKDESTATDMRELLKEE
NmFrpB_Z4696             1  .............................VLDTVTVKGDRQGSKIRTNIVTLQQKDESTATDMRELLKEE
NmFrpB_NM1264            1  MNAPFFRLSLLSLTLAAGFAHAAENNANVALDTVTVKGDRQGSKIRTNIVTLQQKDESTATDMRELLKEE
NmFrpB_NCTC8554          1  MNAPFFRLSLLSLTLAAGFAHAAENNANVALDTVTVKGDRQGSKIRTNIVTLQQKDESTATDMRELLKEE
NmFrpB_2996              1  ......................AENNAKVALDTVTVKGDRQGSKIRTNIVTLQQKDESTATDMRELLKEE
NmFrpB_MC58              1  MNTPLFRLSLLSLTLAAGFAHAAENNAKVVLDTVTVKGDRQGSKIRTNIVTLQQKDESTATDMRELLKEE
NmFrpB_892257            1  ......................AENNAKVVLDTVTVKGDRQGSKIRTNIVTLQQKDESTATDMRELLKEE
NmFrpB_M26503            1  MNAPFFRLSLLSLTLAAGFAHAAENNANVALDTVTVKGDRQGSKIRTNIVTLQQKDESTATDMRELLKEE
NmFrpB_CHUV              1  ....MFRLSLLSLTLAAGFAHAAENNAKVVLDTVTVKGDRQGSKIRTNIVTLQQKDESTATDMRELLKEE
NmFrpB_Z4756             1  .............................VLDTVTVKGDRQGSKIRTNIVTLQQKDESTATDMRELLKEE
NmFrpB_alpha14           1  MNAPFFRLSLLSLTLAAGFAHAAENNANVALDTVTVKGDRQGSKIRTNIVTLQQKDESTATDMRELLKEE
NgFrpB_WHO_Y             1  MNAPFFRLSLLSLTLAAGFAHAAENNANVALDTVTVKGDRQGSKIRTNIVTLQQKDESTATDMRELLKEE
NgFrpB_DGI2              1  MNAPFFRLSLLSLTLAAGFAHAAENNANVALDTVTVKGDRQGSKIRTNIVTLQQKDESTATDMRELLKEE
NgFrpB_WHO_F             1  MNAPFFRLSLLSLTLAAGFAHAAENNANVALDTVTVKGDRQGSKIRTNIVTLQQKDESTATDMRELLKEE
NmFrpB_alpha153          1  MNAPFFRLSLLSLTLAAGFAHAAENNANVALDTVTVKGDRQGSKIRTNIVTLQQKDESTATDMRELLKEE
NgFrpB_NG-k51.05         1  MNAPFFRLSLLSLTLAAGFAHAAENNANVALDTVTVKGDRQGSKIRTNIVTLQQKDESTATDMRELLKEE
NgFrpB_WHO_P             1  MNAPFFRLSLLSLTLAAGFAHAAENNANVALDTVTVKGDRQGSKIRTNIVTLQQKDESTATDMRELLKEE
NgFrpB_WHO_U             1  MNAPFFRLSLLSLTLAAGFAHAAENNANVALDTVTVKGDRQGSKIRTNIVTLQQKDESTATDMRELLKEE
NgFrpB_WHO_G             1  MNAPFFRLSLLSLTLAAGFAHAAENNANVALDTVTVKGDRQGSKIRTNIVTLQQKDESTATDMRELLKEE
NgFrpB_WHO_N             1  MNAPFFRLSLLSLTLAAGFAHAAENNANVALDTVTVKGDRQGSKIRTNIVTLQQKDESTATDMRELLKEE
NgFrpB_WHO_O             1  MNAPFFRLSLLSLTLAAGFAHAAENNANVALDTVTVKGDRQGSKIRTNIVTLQQKDESTATDMRELLKEE
NgFrpB_WHO_V             1  MNAPFFRLSLLSLTLAAGFAHAAENNANVALDTVTVKGDRQGSKIRTNIVTLQQKDESTATDMRELLKEE
NgFrpB_FA1090            1  MNAPFFRLSLLSLTLAAGFAHAAENNANVALDTVTVKGDRQGSKIRTNIVTLQQKDESTATDMRELLKEE
NgFrpB_WHO_L             1  MNAPFFRLSLLSLTLAAGFAHAAENNANVALDTVTVKGDRQGSKIRTNIVTLQQKDESTATDMRELLKEE
NgFrpB_WHO_M             1  MNAPFFRLSLLSLTLAAGFAHAAENNANVALDTVTVKGDRQGSKIRTNIVTLQQKDESTATDMRELLKEE
NgFrpB_FA19              1  MNAPFFRLSLLSLTLAAGFAHAAENNANVALDTVTVKGDRQGSKIRTNIVTLQQKDESTATDMRELLKEE
NgFrpB_MIA_2011_03-10    1  MNAPFFRLSLLSLTLAAGFAHAAENNANVALDTVTVKGDRQGSKIRTNIVTLQQKDESTATDMRELLKEE
NgFrpB_WHO_K             1  MNAPFFRLSLLSLTLAAGFAHAAENNANVALDTVTVKGDRQGSKIRTNIVTLQQKDESTATDMRELLKEE
NgFrpB_WHO_W             1  MNAPFFRLSLLSLTLAAGFAHAAENNANVALDTVTVKGDRQGSKIRTNIVTLQQKDESTATDMRELLKEE
NgFrpB_WHO_X             1  MNAPFFRLSLLSLTLAAGFAHAAENNANVALDTVTVKGDRQGSKIRTNIVTLQQKDESTATDMRELLKEE
NgFrpB_WHO_Z             1  MNAPFFRLSLLSLTLAAGFAHAAENNANVALDTVTVKGDRQGSKIRTNIVTLQQKDESTATDMRELLKEE
```

*NmFrpB_F3-3*: TT β2 η1 β3 TT β4 TT TT η2 β5 η3

```
NmFrpB_F3-3             50  PSIDFGGGNGTSQFLTLRGMGQNSVDIKVDNAYSDSQILYHQGRFIVDPALVKVVSVQKGAGSASAGIGA
NmFrpB_alpha522         71  PSIDFGGGNGTSQFLTLRGMGQNSVDIKVDNAYSDSQILYHQGRFIVDPALVKVVSVQKGAGSASAGIGA
NmFrpB_alpha275         71  PSIDFGGGNGTSQFLTLRGMGQNSVDIKVDNAYSDSQILYHQGRFIVDPALVKVVSVQKGAGSASAGIGA
NmFrpB_053442           71  PSIDFGGGNGTSQFLTLRGMGQNSVDIKVDNAYSDSQILYHQGRFIVDPALVKVVSVQKGAGSASAGIGA
NmFrpB_H44/76           71  PSIDFGGGNGTSQFLTLRGMGQNSVDIKVDNAYSDSQILYHQGRFIVDPALVKVVSVQKGAGSASAGIGA
NmFrpB_Z4696            42  PSIDFGGGNGTSQFLTLRGMGQNSVDIKVDNAYSDSQILYHQGRFIVDPALVKVVSVQKGAGSASAGIGA
NmFrpB_NM1264           71  PSIDFGGGNGTSQFLTLRGMGQNSVDIKVDNAYSDSQILYHQGRFIVDPALVKVVSVQKGAGSASAGIGA
NmFrpB_NCTC8554         71  PSIDFGGGNGTSQFLTLRGMGQNSVDIKVDNAYSDSQILYHQGRFIVDPALVKVVSVQKGAGSASAGIGA
NmFrpB_2996             49  PSIDFGGGNGTSQFLTLRGMGQNSVDIKVDNAYSDSQILYHQGRFIVDPALVKVVSVQKGAGSASAGIGA
NmFrpB_MC58             71  PSIDFGGGNGTSQFLTLRGMGQNSVDIKVDNAYSDSQILYHQGRFIVDPALVKVVSVQKGAGSASAGIGA
NmFrpB_892257           49  PSIDFGGGNGTSQFLTLRGMGQNSVDIKVDNAYSDSQILYHQGRFIVDPALVKVVSVQKGAGSASAGIGA
NmFrpB_M26503           71  PSIDFGGGNGTSQFLTLRGMGQNSVDIKVDNAYSDSQILYHQGRFIVDPALVKVVSVQKGAGSASAGIGA
NmFrpB_CHUV             67  PSIDFGGGNGTSQFLTLRGMGQNSVDIKVDNAYSDSQILYHQGRFIVDPALVKVVSVQKGAGSASAGIGA
NmFrpB_Z4756            42  PSIDFGGGNGTSQFLTLRGMGQNSVDIKVDNAYSDSQILYHQGRFIVDPALVKVVSVQKGAGSASAGIGA
NmFrpB_alpha14          71  PSIDFGGGNGTSQFLTLRGMGQNSVDIKVDNAYSDSQILYHQGRFIVDPALVKVVSVQKGAGSASAGIGA
NgFrpB_WHO_Y            71  PSIDFGGGNGTSQFLTLRGMGQNSVDIKVDNAYSDSQILYHQGRFIVDPALVKVVSVQKGAGSASAGIGA
NgFrpB_DGI2             71  PSIDFGGGNGTSQFLTLRGMGQNSVDIKVDNAYSDSQILYHQGRFIVDPALVKVVSVQKGAGSASAGIGA
NgFrpB_WHO_F            71  PSIDFGGGNGTSQFLTLRGMGQNSVDIKVDNAYSDSQILYHQGRFIVDPALVKVVSVQKGAGSASAGIGA
NmFrpB_alpha153         71  PSIDFGGGNGTSQFLTLRGMGQNSVDIKVDNAYSDSQILYHQGRFIVDPALVKVVSVQKGAGSASAGIGA
NgFrpB_NG-k51.05        71  PSIDFGGGNGTSQFLTLRGMGQNSVDIKVDNAYSDSQILYHQGRFIVDPALVKVVSVQKGAGSASAGIGA
NgFrpB_WHO_P            71  PSIDFGGGNGTSQFLTLRGMGQNSVDIKVDNAYSDSQILYHQGRFIVDPALVKVVSVQKGAGSASAGIGA
NgFrpB_WHO_U            71  PSIDFGGGNGTSQFLTLRGMGQNSVDIKVDNAYSDSQILYHQGRFIVDPALVKVVSVQKGAGSASAGIGA
NgFrpB_WHO_G            71  PSIDFGGGNGTSQFLTLRGMGQNSVDIKVDNAYSDSQILYHQGRFIVDPALVKVVSVQKGAGSASAGIGA
NgFrpB_WHO_N            71  PSIDFGGGNGTSQFLTLRGMGQNSVDIKVDNAYSDSQILYHQGRFIVDPALVKVVSVQKGAGSASAGIGA
NgFrpB_WHO_O            71  PSIDFGGGNGTSQFLTLRGMGQNSVDIKVDNAYSDSQILYHQGRFIVDPALVKVVSVQKGAGSASAGIGA
NgFrpB_WHO_V            71  PSIDFGGGNGTSQFLTLRGMGQNSVDIKVDNAYSDSQILYHQGRFIVDPALVKVVSVQKGAGSASAGIGA
NgFrpB_FA1090           71  PSIDFGGGNGTSQFLTLRGMGQNSVDIKVDNAYSDSQILYHQGRFIVDPALVKVVSVQKGAGSASAGIGA
NgFrpB_WHO_L            71  PSIDFGGGNGTSQFLTLRGMGQNSVDIKVDNAYSDSQILYHQGRFIVDPALVKVVSVQKGAGSASAGIGA
NgFrpB_WHO_M            71  PSIDFGGGNGTSQFLTLRGMGQNSVDIKVDNAYSDSQILYHQGRFIVDPALVKVVSVQKGAGSASAGIGA
NgFrpB_FA19             71  PSIDFGGGNGTSQFLTLRGMGQNSVDIKVDNAYSDSQILYHQGRFIVDPALVKVVSVQKGAGSASAGIGA
NgFrpB_MIA_2011_03-10   71  PSIDFGGGNGTSQFLTLRGMGQNSVDIKVDNAYSDSQILYHQGRFIVDPALVKVVSVQKGAGSASAGIGA
NgFrpB_WHO_K            71  PSIDFGGGNGTSQFLTLRGMGQNSVDIKVDNAYSDSQILYHQGRFIVDPALVKVVSVQKGAGSASAGIGA
NgFrpB_WHO_W            71  PSIDFGGGNGTSQFLTLRGMGQNSVDIKVDNAYSDSQILYHQGRFIVDPALVKVVSVQKGAGSASAGIGA
NgFrpB_WHO_X            71  PSIDFGGGNGTSQFLTLRGMGQNSVDIKVDNAYSDSQILYHQGRFIVDPALVKVVSVQKGAGSASAGIGA
NgFrpB_WHO_Z            71  PSIDFGGGNGTSQFLTLRGMGQNSVDIKVDNAYSDSQILYHQGRFIVDPALVKVVSVQKGAGSASAGIGA
```

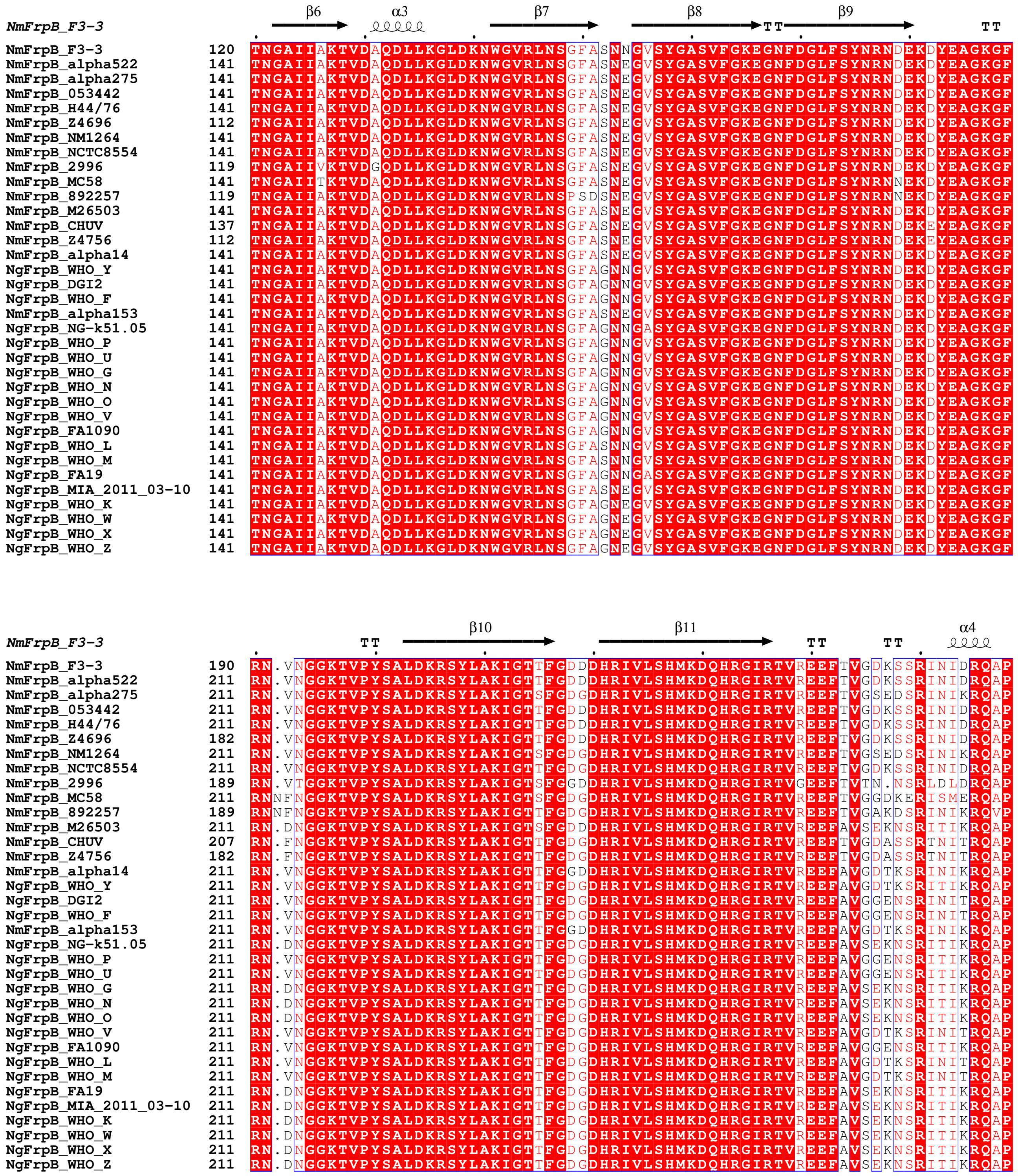
NmFrpB_F3-3
β6
α3
β7
β8
β9
TT
β10
β11
α4

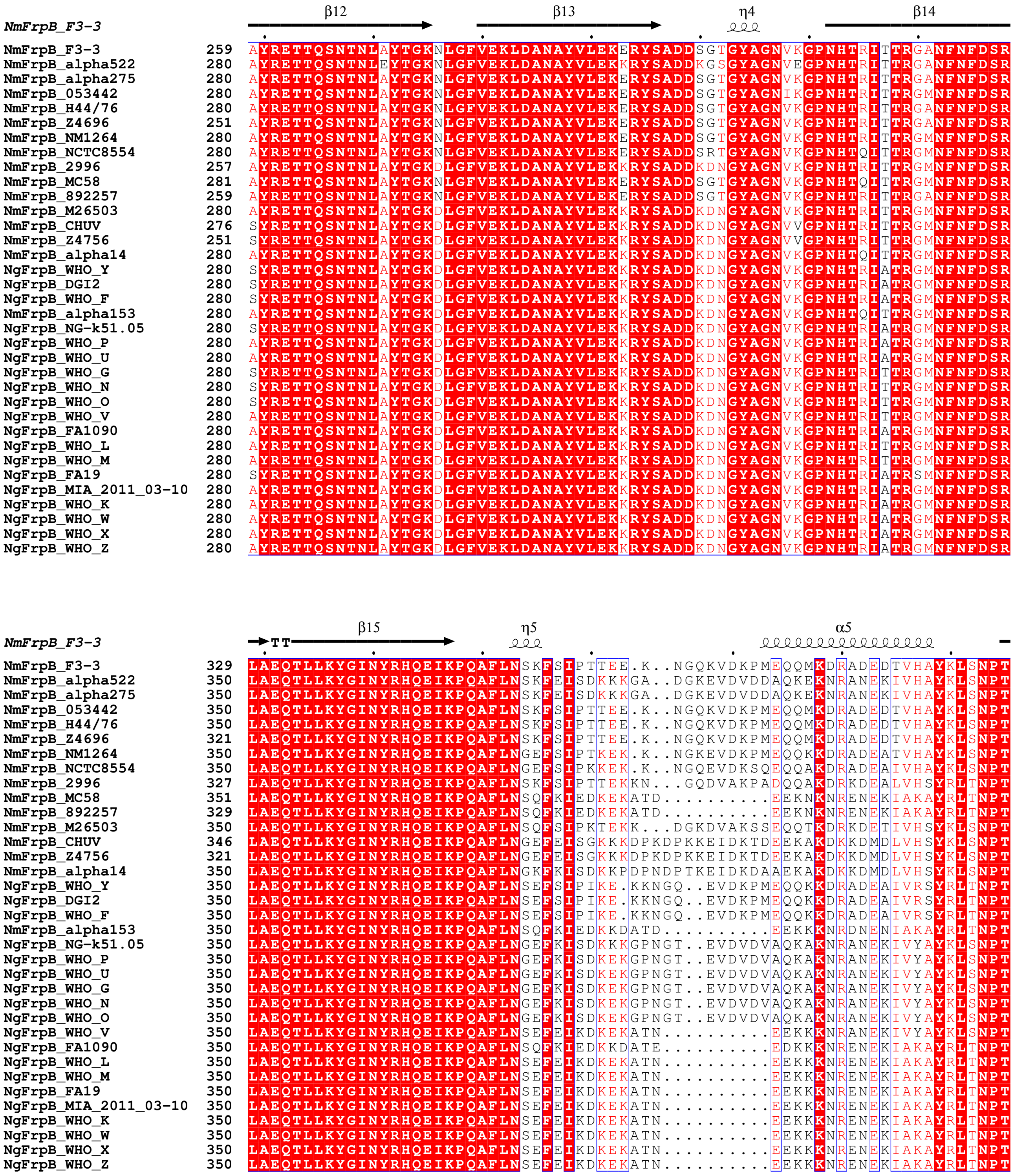
NmFrpB_F3-3
β12
β13
η4
β14
β15
η5
α5
TT

```
                                  β16          β17           β18          β19
NmFrpB_F3-3                    ━━━━━━━━━━━━━TT━━━━━━━━━━━━━ TT ━━━━━━━━━━━━━━━━TT━━━━━━━━━━━
NmFrpB_F3-3              396   KTDTGVYIEAIHDIGDFTLTGGLRYDRFKVKTHDGKTVSSSNLNPSFGVIWQPHEHWSFSASHNYASRSP
NmFrpB_alpha522          418   KTDTGAYIEAIHEIDGFTLTGGLRYDRFKVKTHDGKTVSSSNLNPSFGVIWQPHEHWSFSASHNYASRSP
NmFrpB_alpha275          418   KTDTGAYIEAIHEIDGFTLTGGLRYDRFKVKTHDGKTVSSSNLNPSFGVIWQPHEHWSFSASHNYASRSP
NmFrpB_053442            417   KTDTGVYVEAIHDIGDFTLTGGLRYDRFKVKTHDGKTVSSSNLNPSFGVIWQPHEHWSFSASHNYASRSP
NmFrpB_H44/76            417   KTDTGVYVEAIHDIGDFTLTGGLRYDRFKVKTHDGKTVSSSNLNPSFGVIWQPHEHWSFSASHNYASRSP
NmFrpB_Z4696             388   KTDTGVYVEAIHDIGDFTLTGGLRYDRFKVKTHDGKTVSSSNLNPSFGVIWQPHEHWSFSASHNYASRSP
NmFrpB_NM1264            417   KTDTGAYIEAIHEINGFTLTGGLRYDRFKVKTHDGKTVSNSSLNPSFGVIWQPHEHWSFSASHNYASRSP
NmFrpB_NCTC8554          417   KTDTGAYIEAIHEINGFTLTGGLRYDRFKVKTHDGKTVSSSSLNPSFGVIWQPHEHWSFSASHNYASRSP
NmFrpB_2996              394   KTDTGAYIEAIHEINGFTLTGGLRYDRFKVKTHDGKTVSSSNLNPSFGVIWQPHEHWSFSASHNYASRSP
NmFrpB_MC58              411   KTDTGAYIEAIHEIDGFTLTGGLRYDRFKVKTHDGKTVSSNNLNPSFGVIWQPHEHWSFSASHNYASRSP
NmFrpB_892257            389   KTDAGAYIEAIHEIDGFTLTGGLRYDRFKVKTHDGKTVSSSNLNPSFGVIWQPHEHWSFSASHNYASRSP
NmFrpB_M26503            417   KTDTGAYIEAIHEIDGFTLTGGLRYDRFKVKTHDGKTVSSSNLNPSFGVIWQPHEHWSFSASHNYASRSP
NmFrpB_CHUV              416   KTDTGAYIEAIHEIDGFTLTGGLRYDRFKVKTHDGKTVSSSNLNPSFGVIWQPHEHWSFSASHNYASRSP
NmFrpB_Z4756             391   KTDTGAYIEAIHELDGFTLTGGLRYDRFKVKTHDGKTVSSSNLNPSFGVIWQPHEHWSFSSSHNYASRSP
NmFrpB_alpha14           420   KTDTGAYIEAIHEIDGFTLTGGLRYDRFKVKTHDGKTVSSSNLNPSFGVIWQPHEHWSFSASHNYASRSP
NgFrpB_WHO_Y             417   KTDTGAYIEAIHEIDGFTLTGGLRYDRFKVKTHDGKTVSSSSLNPSFGVIWQPREHWSFSASHNYASRSP
NgFrpB_DGI2              417   KTDTGAYIEAIHEIDGFTLTGGLRYDRFKVKTHDGKTVSSSSLNPSFGVIWQPREHWSFSASHNYASRSP
NgFrpB_WHO_F             417   KTDTGAYIEAIHEINGFTLTGGLRYDRFKVKTHDGKTVSSSSLNPSFGVIWQPREHWSFSASHNYAGRSP
NmFrpB_alpha153          410   KTDTGAYIEAIHEIDGFTLTGGLRYDRFKVKTHDGKTVSSSNLNPSFGVIWQPHEHWSFSASHNYASRSP
NgFrpB_NG-k51.05         418   KTDTGAYIEAIHEIDGFTLTGGLRYDRFKVKTHDGKTVSSSSLNPSFGVIWQPREHWSFSASHNYAGRSP
NgFrpB_WHO_P             418   KTDTGAYIEAIHEIDGFTLTGGLRYDRFKVKTHDGKTVSSSSLNPSFGVIWQPHEHWSFSASHNYAGRSP
NgFrpB_WHO_U             418   KTDTGAYIEAIHEIDGFTLTGGLRYDRFKVKTHDGKTVSSSSLNPSFGVIWQPHEHWSFSASHNYAGRSP
NgFrpB_WHO_G             418   KTDTGAYIEAIHEIDGFTLTGGLRYDRFKVKTHDGKTVSSSSLNPSFGVIWQPREHWNFSASHNYASRSP
NgFrpB_WHO_N             418   KTDTGAYIEAIHEIDGFTLTGGLRYDRFKVKTHDGKTVSSSSLNPSFGVIWQPREHWNFSASHNYASRSP
NgFrpB_WHO_O             418   KTDTGAYIEAIHEIDGFTLTGGLRYDRFKVKTHDGKTVSSSSLNPSFGVIWQPREHWNFSASHNYASRSP
NgFrpB_WHO_V             410   KTDTGAYIEAIHEIDGFTLTGGLRYDRFKVKTHDGKTVSSSSLNPSFGVIWQPREHWNFSASHNYASRSP
NgFrpB_FA1090            410   KTDTGAYIEAIHEIDGFTLTGGLRYDRFKVKTHDGKTVSSSSLNPSFGVIWQPREHWSFSASHNYASRSP
NgFrpB_WHO_L             410   KTDTGAYIEAIHEIDGFTLTGGLRYDRFKVKTHDGKTVSSSSLNPSFGVIWQPREHWSFSASHNYAGRSP
NgFrpB_WHO_M             410   KTDTGAYIEAIHEIDGFTLTGGLRYDRFKVKTHDGKTVSSSSLNPSFGVIWQPREHWSFSASHNYAGRSP
NgFrpB_FA19              410   KTDTGAYIEAIHEIDGFTLTGGLRYDRFKVKTHDGKTVSSSSLNPSFGVIWQPREHWSFSASHNYASRSP
NgFrpB_MIA_2011_03-10    410   KTDTGAYIEAIHEIDGFTLTGGLRYDRFKVKTHDGKTVSSSSLNPSFGVIWQPREHWSFSASHNYASRSP
NgFrpB_WHO_K             410   KTDTGAYIEAIHEIDGFTLTGGLRYDRFKVKTHDGKTVSSSSLNPSFGVIWQPREHWSFSASHNYAGRSP
NgFrpB_WHO_W             410   KTDTGAYIEAIHEIDGFTLTGGLRYDRFKVKTHDGKTVSSSSLNPSFGVIWQPREHWSFSASHNYAGRSP
NgFrpB_WHO_X             410   KTDTGAYIEAIHEIDGFTLTGGLRYDRFKVKTHDGKTVSSSSLNPSFGVIWQPREHWSFSASHNYAGRSP
NgFrpB_WHO_Z             410   KTDTGAYIEAIHEIDGFTLTGGLRYDRFKVKTHDGKTVSSSSLNPSFGVIWQPREHWSFSASHNYAGRSP
```

```
                                  η6    β20          β21              β22                β23
NmFrpB_F3-3                       ℓℓℓ TT ━━━ TT  ━━━━━━━━━━━━━   ━━━━━━━━━━━━━━              ━━━
NmFrpB_F3-3              466   RLYDALQTHGKRGIISIADGTKAERARNTEIGFNYNDGTFAANGSYFWQTIKDALANPQNRHDSVAVREA
NmFrpB_alpha522          488   RLYDALQTHGKRGIISIADGTKAERARNTEIGFNYNDGTFAANGSYFWQTIKDALANPQNRHVSAAVREA
NmFrpB_alpha275          488   RLYDALQTHGKRGIISIADGTKAERARNTEIGFNYNNGTFAANGSYFWQTIKDALANPQNRHDSVAVREA
NmFrpB_053442            487   RLYDALQTHGKRGIISIADGTKAERARNTEIGFNYNDGTFAANGSYFWQTIKDALANPQNRHDSVAVREA
NmFrpB_H44/76            487   RLYDALQTHGKRGIISIADGTKAERARNTEIGFNYNDGTFAANGSYFWQTIKDALANPQNRHDSVAVREA
NmFrpB_Z4696             458   RLYDALQTHGKRGIISIADGTKAERARNTEIGFNYNDGTFAANGSYFWQTIKDALANPQNRHDSVAVREA
NmFrpB_NM1264            487   RLYDALQTHGKRGIISIADGTKAERARNTEIGFNYNDGTFAANGSYFWQTIKDALANPQNRHDSVAVREA
NmFrpB_NCTC8554          487   RLYDALQTHGKRGIISIADGTKAERARNTEIGFNYNDGTFAANGSYFWQTIKDALANPQNRHVSAAVREA
NmFrpB_2996              464   RLYDALQTHGKRGIISIGDGTKAERARNTEIGFNYNEGTFAANGRYFWQTIKDALANPQNRHVSAAVREA
NmFrpB_MC58              481   RLYDALQTHGKRGIISIADGTKAERARNTEIGFNYNDGTFAANGSYFWQTIKDALANPQNRHDSVAVREA
NmFrpB_892257            459   RLYDALQTHGKRGIISIADGTKAERARNTEIGFNYNDGTFAANGSYFWQTIKDALANPQNRHDSVAVREA
NmFrpB_M26503            487   RLYDALQTHGKRGIISIADGTKAERARNTEIGFNYNDGTFAANGSYFWQTIKDALANPQNRHDSVAVREA
NmFrpB_CHUV              486   RLYDALQTHGKRGIISIADGTKAERARNTEIGFNYNDGTFAANGSYFWQTIKDALANPQNRHVSAAVREA
NmFrpB_Z4756             461   RLYDALQTHGKRGIISIADGTKAERARNTEIGFNYNDGTFAANGSYFWQTIKDALANPQNRHVSAAVREA
NmFrpB_alpha14           490   RLYDALQTHGKRGIISIADGTKAERARNTEIGFNYNDGTFAANGSYFWQTIKDALANPQNRHDSVAVREA
NgFrpB_WHO_Y             487   RLYDALQTHGKRGIISIADGTKAERARNTEIGFNYNDGTFAANGSYFRQTIKDALANPQNRHDSVAVREA
NgFrpB_DGI2              487   RLYDALQTHGKRGIISIADGTKAERARNTEIGFNYNDGTFAANGSYFRQTIKDALANPQNRHDSVAVREA
NgFrpB_WHO_F             487   RLYDALQTHGKRGIISIADGTKAERARNTEIGFNYNDGTFAANGSYFRQTIKDALANPQNRHDSVAVREA
NmFrpB_alpha153          480   RLYDALQTHGKRGIISIADGTKAERARNTEIGFNYNDGTFAANGSYFWQTIKDALANPQNRHDSVAVREA
NgFrpB_NG-k51.05         488   RLYDALQTHGKRGIISIADGTKAERARNTEIGFNYNDGTFAANGSYFRQTIKDALANPQNRHDSVAVREA
NgFrpB_WHO_P             488   RLYDALQTHGKRGIISIADGTKAERARNTEIGFNYNDGTFAANGSYFRQTIKDALANPQNRHDSVAVREA
NgFrpB_WHO_U             488   RLYDALQTHGKRGIISIADGTKAERARNTEIGFNYNDGTFAANGSYFRQTIKDALANPQNRHDSVAVREA
NgFrpB_WHO_G             488   RLYDALQTHGKRGIISIADGTKAERARNTEIGFNYNDGTFAANGSYFRQTIKDALANPQNRHDSVAVREA
NgFrpB_WHO_N             488   RLYDALQTHGKRGIISIADGTKAERARNTEIGFNYNDGTFAANGSYFRQTIKDALANPQNRHDSVAVREA
NgFrpB_WHO_O             488   RLYDALQTHGKRGIISIADGTKAERARNTEIGFNYNDGTFAANGSYFRQTIKDALANPQNRHDSVAVREA
NgFrpB_WHO_V             480   RLYDALQTHGKRGIISIADGTKAERARNTEIGFNYNDGTFAANGSYFRQTIKDALANPQNRHDSVAVREA
NgFrpB_FA1090            480   RLYDALQTHGKRGIISIADGTKAERARNTEIGFNYNDGTFAANGSYFRQTIKDALANPQNRHDSVAVREA
NgFrpB_WHO_L             480   RLYDALQTHGKRGIISIADGTKAERARNTEIGFNYNDGTFAANGSYFRQTIKDALANPQNRHDSVAVREA
NgFrpB_WHO_M             480   RLYDALQTHGKRGIISIADGTKAERAHNTEIGFNYNDGTFAANGSYFRQTIKDALANPQNRHDSVAVREA
NgFrpB_FA19              480   RLYDALQTHGKRGIISIADGTKAERARNTEIGFNYNDGTFAANGSYFRQTIKDALANPQNRHDSVAVREA
NgFrpB_MIA_2011_03-10    480   RLYDALQTHGKRGIISIADGTKAERARNTEIGFNYNDGTFAANGSYFRQTIKDALANPQNRHDSVAVREA
NgFrpB_WHO_K             480   RLYDALQTHGKRGIISIADGTKAERARNTEIGFNYNDGTFAANGSYFRQTIKDALANPQNRHDSVAVREA
NgFrpB_WHO_W             480   RLYDALQTHGKRGIISIADGTKAERARNTEIGFNYNDGTFAANGSYFRQTIKDALANPQNRHDSVAVREA
NgFrpB_WHO_X             480   RLYDALQTHGKRGIISIADGTKAERARNTEIGFNYNDGTFAANGSYFRQTIKDALANPQNRHDSVAVREA
NgFrpB_WHO_Z             480   RLYDALQTHGKRGIISIADGTKAERARNTEIGFNYNDGTFAANGSYFRQTIKDALANPQNRHDSVAVREA
```

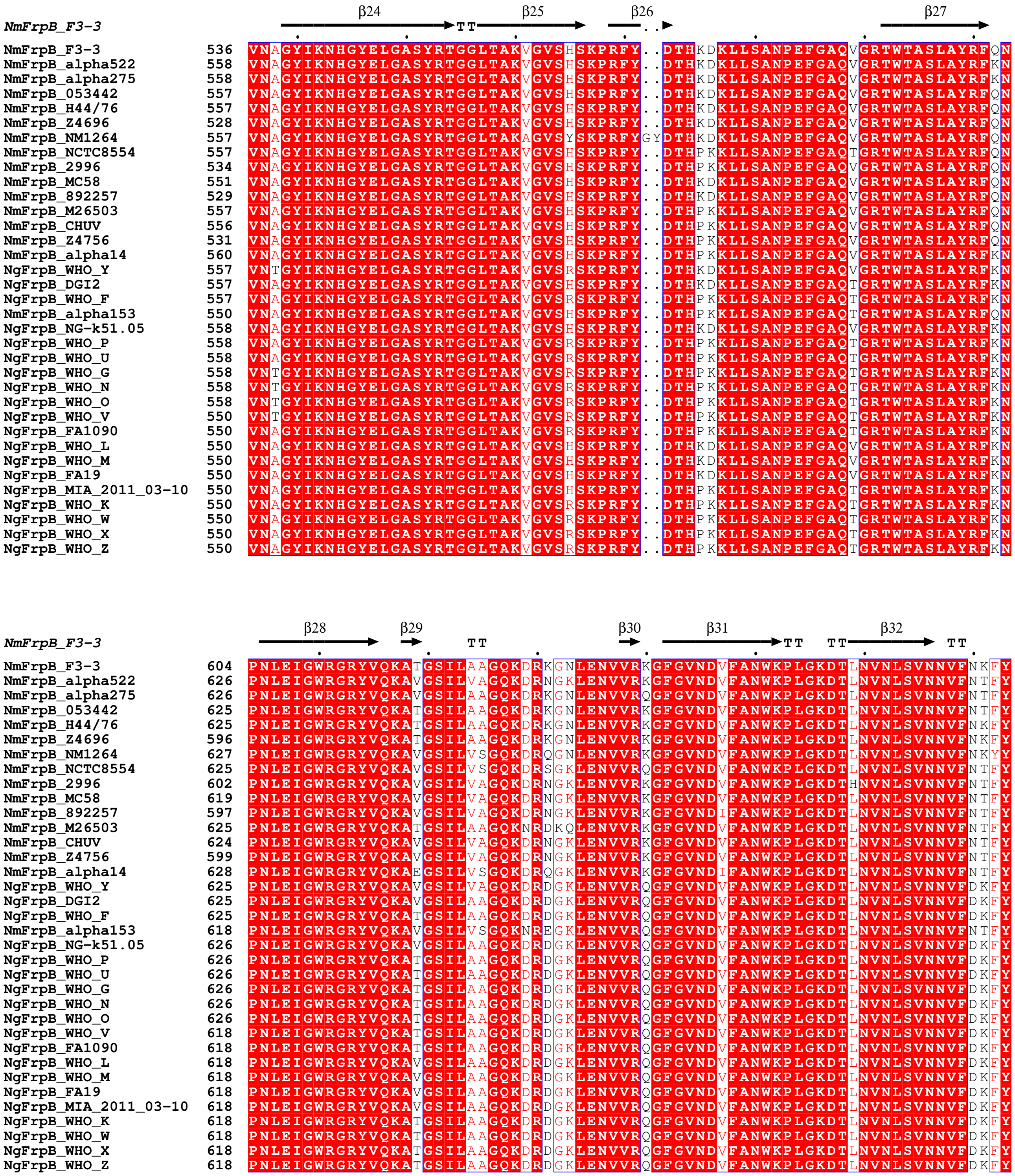
NmFrpB_F3-3
β24
β25
β26
β27
β28
β29
β30
β31
β32

```
                                              β33
NmFrpB_F3-3                       TTT   ───────────►
                                   .          .
NmFrpB_F3-3              674  YPHSQRWTNTLPGVGRDVRLGVNYKF
NmFrpB_alpha522          696  YPHSQRWTNTLPGVGRDVRLGVNYKF
NmFrpB_alpha275          696  YPHSQRWNNTLPGVGRDVRLGVNYKF
NmFrpB_053442            695  YPHSQRWTNTLPGTGRDVRLGVNYKF
NmFrpB_H44/76            695  YPHSQRWTNTLPGVGRDVRLGVNYKF
NmFrpB_Z4696             666  YPHS......................
NmFrpB_NM1264            697  YPHSQRWNNTLPGVGRDVRLGVNYKF
NmFrpB_NCTC8554          695  YPHSQRWTNTLPGVGRDVRLGVNYKF
NmFrpB_2996              672  YPHSQRWTNTLPDVGRDVRLGVNYKF
NmFrpB_MC58              689  YPHSQRWTNTLPGVGRDVRLGVNYKF
NmFrpB_892257            667  YPHSQRWTNTLPDVGRDVRLGVNYKF
NmFrpB_M26503            695  YPHSQRWTNTLPGVGRDVRLGVNYKF
NmFrpB_CHUV              694  YPHSQRWTNTLPGVGRDVRLGVNYKF
NmFrpB_Z4756             669  YPHS......................
NmFrpB_alpha14           698  YPHSQRWTNTLPGVGRDVRLGVNYKF
NgFrpB_WHO_Y             695  YPHSQRWTNTLPGVGRDVRLGVNYKF
NgFrpB_DGI2              695  YPHSQRWTNTLPGVGRDVRLGVNYKF
NgFrpB_WHO_F             695  YPHSQRWTNTLPGVGRDVRLGVNYKF
NmFrpB_alpha153          688  YPHSQRWTNTLPGVGRDVRLGVNYKF
NgFrpB_NG-k51.05         696  YPHSQRWTNTLPGVGRDVRLGVNYKF
NgFrpB_WHO_P             696  YPHSQRWTNTLPGVGRDVRLGVNYKF
NgFrpB_WHO_U             696  YPHSQRWTNTLPGVGRDVRLGVNYKF
NgFrpB_WHO_G             696  YPHSQRWTNTLPGVGRDVRLGVNYKF
NgFrpB_WHO_N             696  YPHSQRWTNTLPGVGRDVRLGVNYKF
NgFrpB_WHO_O             696  YPHSQRWTNTLPGVGRDVRLGVNYKF
NgFrpB_WHO_V             688  YPHSQRWTNTLPGVGRDVRLGVNYKF
NgFrpB_FA1090            688  YPHSQRWTNTLPGVGRDVRLGVNYKF
NgFrpB_WHO_L             688  YPHSQRWTNTLPGVGRDVRLGVNYKF
NgFrpB_WHO_M             688  YPHSQRWTNTLPGVGRDVRLGVNYKF
NgFrpB_FA19              688  YPHSQRWTNTLPGVGRDVRLGVNYKF
NgFrpB_MIA_2011_03-10    688  YPHSQRWTNTLPGVGRDVRLGVNYKF
NgFrpB_WHO_K             688  YPHSQRWTNTLPGVGRDVRLGVNYKF
NgFrpB_WHO_W             688  YPHSQRWTNTLPGVGRDVRLGVNYKF
NgFrpB_WHO_X             688  YPHSQRWTNTLPGVGRDVRLGVNYKF
NgFrpB_WHO_Z             688  YPHSQRWTNTLPGVGRDVRLGVNYKF
```

## Supplementary Figure 6. Sequence alignment of TdfHs.

```
                                                                                 β1
NgTdfH_FA1090
NgTdfH_FA1090               1  .......MRSSFRLKPICFYLMGVMLYHHSYAEDAGRAGSEAQIQVLEDVHVKAKRVPKDKKVFTDARAV
NgTdfH_WHO_M                1  .......MRSSFRLKPICFYLMGVMLYHHSYAEDAGRAGSEAQIQVLEDVHVKAKRVPKDKKVFTDARAV
NgTdfH_WHO_O                1  .......MRSSFRLKPICFYLMGVMLYHHSYAEDAGRAGSEAQIQVLEDVHVKAKRVPKDKKVFTDARAV
NgTdfH_WHO_U                1  .......MRSSFRLKPICFYLMGVMLYHHSYAEDAGRAGSEAQIQVLEDVHVKAKRVPKDKKVFTDARAV
NgTdfH_WHO_P                1  .......MRSSFRLKPICFYLMGVMLYHHSYAEDAGRAGSEAQIQVLEDVHVKAERVPKDKKVFTDARAV
NgTdfH_MS11                 1  .......MRSSFRLKPICFYLMGVMLYHHSYAEDAGRAGSEAQIQVLEDVHVKAKRVPKDKKVFTDARAV
NgTdfH_F62                  1  .......MRSSFRLKPICFYLMGVMLYHHSYAEDAGRAGSEAQIQVLEDVHVKAKRVPKDKKVFTDARAV
NgTdfH_WHO_F                1  .........................MLYHHSYAEDAGRAGSEAQIQVLEDVHVKAGRVPKDKKVFTDARAV
NgTdfH_MIA_2011_03-10       1  MFIVEENIRSSFRLKPICFYLMGVMLYHHSYAEDAGRAGSEAQIQVLEDVHVKAKRVPKDKKVFTDARAV
NgTdfH_DGI18                1  .......MRSSFRLKPICFYLMGVMLYHHSYAEDAGRAGSEAQIQVLEDVHVKAERVPKDKKVFTDARAV
NgTdfH_FA19                 1  .......MRSSFRLKPICFYLMGVMLYHHSYAEDAGRAGSEAQIQVLEDVHVKAKRVPKDKKVFTDARAV
NgTdfH_WHO_L                1  .......MRSSFRLKPICFYLMGVMLYHHSYAEDAGRAGSEAQIQVLEDVHVKAKRVPKDKKVFTDARAV
NgTdfH_DG12                 1  .............MKPICFYLMGVMLYHHSYAEDAGRAGSEAQIQVLEDVHVKAERVPKDKKVFTDARAV
NgTdfH_WHO_K                1  .........................MLYHHSYAEDAGRAGSEAQIQVLEDVHVKAKRVPKDKKVFTDARAV
NgTdfH_WHO_W                1  .........................MLYHHSYAEDAGRAGSEAQIQVLEDVHVKAKRVPKDKKVFTDARAV
NgTdfH_WHO_X                1  .........................MLYHHSYAEDAGRAGSEAQIQVLEDVHVKAKRVPKDKKVFTDARAV
NgTdfH_WHO_Z                1  .........................MLYHHSYAEDAGRAGSEAQIQVLEDVHVKAKRVPKDKKVFTDARAV
NgTdfH_WHO_G                1  .......MRSSFRLKPICFYLMGVMLYHHSYAEDAGRAGSEAQIQVLEDVHVKAKRVPKDKKVFTDARAV
NgTdfH_WHO_N                1  .......MRSSFRLKPICFYLMGVMLYHHSYAEDAGRAGSEAQIQVLEDVHVKAKRVPKDKKVFTDARAV
NgTdfH_NG-k51.05            1  MFIVEENIRSSFRLKPICFYLMGVMLYHHSYAEDAGRAGSEAQIQVLEDVHVKAKRVPKDKKVFTDARAV
NgTdfH_WHO_V                1  .........................MLYHHSYAEDAGRAGSEAQIQVLEDVHVKAKRVPKDKKVFTDARAV
NgTdfH_WHO_Y                1  .........................MLYHHSYAEDAGRAGSEAQIQVLEDVHVKAKRVPKDKKVFTDARAV
NgTdfH_BZG33                1  .......MRSSFRLKPICFYLMGVMLYHHSYAEDAGRAGSEAQIQVLEDVHVKAKRVPKDKKVFTDARAV
NgTdfH_NCCP11945            1  .........................MLYHHSYAEDAGRAGSEAQIQVLEDVHVKAKRVPKDKKVFTDARAV
NmTdfH_020-06               1  .......MKTSFRLKPIYFCLLNVTLYH.SYAEDLPRHSHNTQVQVLEDVHVKAKRVPKDKKVFTDARAV
NmTdfH_alpha710             1  .......MRSSFRLKPICFYLMGVMLYHHSYAEDAGRAGSEAQIQVLEDVHVKAKRVPKDKKVFTDARAV
NmTdfH_NZ-05/33             1  .......MRSSFRLKPICFYLMGVMLYHHSYAEDAGRAGSEAQIQVLEDVHVKAKRVPKDKKVFTDARAV
NmTdfH_93004                1  .......MRSSFRLKPICFYLMGVMLYHHSYAEDAGRAGSEAQIQVLEDVHVKAKRVPKDKKVFTDARAV
NmTdfH_ATCC13091            1  .......MKSSFRLKPICFYLMGVMLYHHSYAEDAGRAGSEAQIQVLEDVHVKAKRVPKDKKVFTDARAV
NmTdfH_alpha522             1  .......MRSSFRLKPICFYLMGVMLYHYSYAEDAGRAGSEAQIQVLEDVHVKAKRVPKDKKVFTDARAV
NmTdfH_NM2795               1  .......MKSSFRLKPICFYLMGVMLYHHSYAEDAGRAGSEAQIQVLEDVHVKAKRVPKDKKVFTDARAV
NmTdfH_81858                1  .......MRSSFRLKPICFYLMGVMLYHYSYAEDAGRAGSEAQIQVLEDVHVKAKRVPKDKKVFTDARAV
NmTdfH_ERS514534            1  .......MRSSFRLKPICFYLMGVMLYHHSYAEDAGRAGSEAQIQVLEDVHVKAKRVPKDKKVFTDARAV
NmTdfH_alpha153             1  .......MRSSFRLKPICFYLMGVMLYHHSYAEDAGRAGSEAQIQVLEDVHVKAKRVPKDKKVFTDARAV
NmTdfH_053442               1  .....................MGVMLYHHSYAEDAGRAGSEAQIQVLEDVHVKAKRVPKDKKVFTDARAV
NmTdfH_M0579                1  .......MRSSFRLKPICFYLTGVMLYHHSYAEDAGRAGSEAQIQVLEDVHVKAKRVPKDKKVFTDARAV
NmTdfH_MC58                 1  .......MRSSFRLKPICFYLMGVTLYHYSYAEDAGRAGSEAQIQVLEDVHVKAKRVPKDKKVFTDARAV
NmTdfH_LNP21362             1  .......MRSSFRLKPICFYLMGVTLYHYSYAEDAGRAGSEAQIQVLEDVHVKAKRVPKDKKVFTDARAV
NmTdfH_IR1074               1  .......MRSSFRLKPICFYLMGVTLYHYSYAEDAGRAGSEAQIQVLEDVHVKAKRVPKDKKVFTDARAV
NmTdfH_H44/76               1  .......MRSSFRLKPICFYLMGVTLYHYSYAEDAGRAGSEAQIQVLEDVHVKAKRVPKDKKVFTDARAV
NmTdfH_FDAARGOS_210         1  .......MRSSFRLKPICFYLMGVTLYHYSYAEDAGRAGSEAQIQVLEDVHVKAKRVPKDKKVFTDARAV
NmTdfH_FDAARGOS_215         1  .......MRSSFRLKPICFYLMGVMLYHHSYAEDAGRAGSEAQIQVLEDVHVKAKRVPKDKKVFTDARAV
NmTdfH_                     1  .......MRSSFRLKPICFYLMGVMLYHHSYAEDAGRAGSEAQIQVLEDVHVKAKRVPKDKKVFTDARAV
NmTdfH_510612               1  .........................MLYHHSYAEDAGRAGSEAQIQVLEDVHVKAKRVPKDKKVFTDARAV
NmTdfH_Z2491                1  .......MRSSFRLKPICFYLMGVMLYHHSYAEDAGRAGSEAQIQVLEDVHVKAKRVPKDKKVFTDARAV
NmTdfH_NCTC8249             1  .......MRSSFRLKPICFYLMGVMLYHHSYAEDAGRAGSEAQIQVLEDVHVKAKRVPKDKKVFTDARAV
NmTdfH_alpha14              1  .......MRSSFRLKPICFYLMGVMLYHHSYAEDAGRAGSEAQIQVLEDVHVKAKRVPKDKKVFTDARAV
NmTdfH_2842STDY5881378      1  .......MRSSFRLKPICFYLMGVMLYHHSYAEDAGRAGSEAQIQVLEDVHVKAKRVPKDKKVFTDARAV
NmTdfH_CHUV                 1  .......MRSSFRLKPICFYLMGVMLYHHSYAEDAGRAGSEAQIQVLEDVHVKAKRVPKDKKVFTDARAV
NmTdfH_FAM18                1  .......MRSSFRLKPICFYLMGVMLYHHSYAEDAGRAGSEAQIQVLEDVHVKAKRVPKDKKVFTDARAV
NmTdfH_M09293               1  .......MRSSFRLKPICFYLMGVMLYHHSYAEDAGRAGSEAQIQVLEDVHVKAKRVPKDKKVFTDARAV
NmTdfH_M26503               1  .......MRSSFRLKPICFYLMGVMLYHHSYAEDAGRAGSEAQIQVLEDVHVKAKRVPKDKKVFTDARAV
NmTdfH_2842STDY5881093      1  .......MRSSFRLKPICFYLMGVMLYHHSYAEDAGRTGSEAQIQVLEDVHVKAKRVPKDKKVFTDARAV
NmTdfH_PMB5301              1  .......MRSSFRLKPICFYLMGVMLYHHSYAEDAGRAGSEAQIQVLEDVHVKAKRVPKDKKVFTDARAV
```

| *NgTdfH_FA1090* | | α1 (helix), α2 (helix), TT β2, β3, TT, β4 TT β5, TT |
|---|---|---|
| NgTdfH_FA1090 | 64 | STRQDVFKSGENLDNIVRSIPGAFTQQDKSSGIVSLNIRGDSGFGRVNTMVDGITQTFYSTSTDAGRAGG |
| NgTdfH_WHO_M | 64 | STRQDIFKSGENLDNIVRSIPGAFTQQDKSSGIVSLNIRGDSGFGRVNTMVDGITQTFYSTSTDAGRAGG |
| NgTdfH_WHO_O | 64 | STRQDIFKSGENLDNIVRSIPGAFTQQDKSSGIVSLNIRGDSGFGRVNTMVDGITQTFYSTSTDAGRAGG |
| NgTdfH_WHO_U | 64 | STRQDIFKSGENLDNIVRSIPGAFTQQDKSSGIVSLNIRGDSGFGRVNTMVDGITQTFYSTSTDAGRAGG |
| NgTdfH_WHO_P | 64 | STRQDIFKSGENLDNIVRSIPGAFTQQDKSSGIVSLNIRGDSGFGRVNTMVDGITQTFYSTSTDAGRAGG |
| NgTdfH_MS11 | 64 | STRQDIFKSGENLDNIVRSIPGAFTQQDKSSGIVSLNIRGDSGFGRVNTMVDGITQTFYSTSTDAGRAGG |
| NgTdfH_F62 | 64 | STRQDVFKSGENLDNIVRSIPGAFTQQDKSSGIVSLNIRGDSGFGRVNTMVDGITQTFYSTSTDAGRAGG |
| NgTdfH_WHO_F | 47 | STRQDVFKSGENLDNIVRSIPGAFTQQDKSSGIVSLNIRGDSGFGRVNTMVDGITQTFYSTSTDAGRAGG |
| NgTdfH_MIA_2011_03-10 | 71 | STRQDVFKSGENLDNIVRSIPGAFTQQDKSSGIVSLNIRGDSGFGRVNTMVDGITQTFYSTSTDAGRAGG |
| NgTdfH_DGI18 | 64 | STRQDVFKSGENLDNIVRSIPGAFTQQDKSSGIVSLNIRGDSGFGRVNTMVDGITQTFYSTSTDAGRAGG |
| NgTdfH_FA19 | 64 | STRQDIFKSGENLDNIVRSIPGAFTQQDKSSGIVSLNIRGDSGFGRVNTMVDGITQTFYSTSTDAGRAGG |
| NgTdfH_WHO_L | 64 | STRQDIFKSGENLDNIVRSIPGAFTQQDKSSGIVSLNIRGDSGFGRVNTMVDGITQTFYSTSTDAGRAGG |
| NgTdfH_DG12 | 58 | STRQDVFKSGENLDNIVRSIPGAFTQQDKSSGIVSLNIRGDSGFGRVNTMVDGITQTFYSTSTDAGRAGG |
| NgTdfH_WHO_K | 47 | STRQDVFKSSENLDNIVRSIPGAFTQQDKSSGIVSLNIRGDSGFGRVNTMVDGITQTFYSTSTDAGRAGG |
| NgTdfH_WHO_W | 47 | STRQDVFKSSENLDNIVRSIPGAFTQQDKSSGIVSLNIRGDSGFGRVNTMVDGITQTFYSTSTDAGRAGG |
| NgTdfH_WHO_X | 47 | STRQDVFKSSENLDNIVRSIPGAFTQQDKSSGIVSLNIRGDSGFGRVNTMVDGITQTFYSTSTDAGRAGG |
| NgTdfH_WHO_Z | 47 | STRQDVFKSSENLDNIVRSIPGAFTQQDKSSGIVSLNIRGDSGFGRVNTMVDGITQTFYSTSTDAGRAGG |
| NgTdfH_WHO_G | 64 | STRQDVFKSSENLDNIVRSIPGAFTQQDKSSGIVSLNIRGDSGFGRVNTMVDGITQTFYSTSTDAGRAGG |
| NgTdfH_WHO_N | 64 | STRQDVFKSSENLDNIVRSIPGAFTQQDKSSGIVSLNIRGDSGFGRVNTMVDGITQTFYSTSTDAGRAGG |
| NgTdfH_NG-k51.05 | 71 | STRQDVFKSSENLDNIVRSIPGAFTQQDKSSGIVSLNIRGDSGFGRVNTMVDGITQTFYSTSTDAGRAGG |
| NgTdfH_WHO_V | 47 | STRQDVFKSSENLDNIVRSIPGAFTQQDKSSGIVSLNIRGDSGFGRVNTMVDGITQTFYSTSTDAGRAGG |
| NgTdfH_WHO_Y | 47 | STRQDVFKSSENLDNIVRSIPGAFTQQDKSSGIVSLNIRGDSGFGRVNTMVDGITQTFYSTSTDAGRAGG |
| NgTdfH_BZG33 | 64 | STRQDVFKSSENLDNIVRSIPGAFTQQDKSSGIVSLNIRGDSGFGRVNTMVDGITQTFYSTSTDAGRAGG |
| NgTdfH_NCCP11945 | 47 | STRQDVFKSSENLDNIVRSIPGAFTQQDKSSGIVSLNIRGDSGFGRVNTMVDGITQTFYSTSTDAGRAGG |
| NmTdfH_020-06 | 63 | STRQDVFKSGENLDTIVRSIPGAFTQQDKSSGIVSLNIRGDSGFGRVNTMVDGITQTFYSTSTDAGRAGG |
| NmTdfH_alpha710 | 64 | STRQDIFKSSENLDNIVRSIPGAFTQQDKSSGIVSLNIRGDSGFGRVNTMVDGITQTFYSTSTDAGRAGG |
| NmTdfH_NZ-05/33 | 64 | STRQDIFKSSENLDNIVRSIPGAFTQQDKSSGIVSLNIRGDSGFGRVNTMVDGITQTFYSTSTDAGRAGG |
| NmTdfH_93004 | 64 | STRQDIFKSSENLDNIVRSIPGAFTQQDKSSGIVSLNIRGDSGFGRVNTMVDGITQTFYSTSTDAGRAGG |
| NmTdfH_ATCC13091 | 64 | STRQDIFKSSENLDNIVRSIPGAFTQQDKSSGIVSLNIRGDSGFGRVNTMVDGITQTFYSTSTDAGRAGG |
| NmTdfH_alpha522 | 64 | STRQDIFKSSENLDNIVRSIPGAFTQQDKSSGIVSLNIRGDSGFGRVNTMVDGITQTFYSTSTDAGRAGG |
| NmTdfH_NM2795 | 64 | STRQDIFKSSENLDNIVRSIPGAFTQQDKSSGIVSLNIRGDSGFGRVNTMVDGITQTFYSTSTDAGRAGG |
| NmTdfH_81858 | 64 | STRQDIFKSSENLDNIVRSIPGAFTQQDKSSGIVSLNIRGDSGFGRVNTMVDGITQTFYSTSTDAGRAGG |
| NmTdfH_ERS514534 | 64 | STRQDIFKSSENLDNIVRSIPGAFTQQDKSSGIVSLNIRGDSGFGRVNTMVDGITQTFYSTSTDAGRAGG |
| NmTdfH_alpha153 | 64 | STRQDIFKSSENLDNIVRSIPGAFTQQDKSSGIVSLNIRGDSGFGRVNTMVDGITQTFYSTSTDAGRAGG |
| NmTdfH_053442 | 50 | STRQDIFKSSENLDNIVRSIPGAFTQQDKSSGIVSLNIRGDSGFGRVNTMVDGITQTFYSTSTDAGRAGG |
| NmTdfH_M0579 | 64 | STRQDIFKSSENLDNIVRSIPGAFTQQDKSSGIVSLNIRGDSGFGRVNTMVDGITQTFYSTSTDAGRAGG |
| NmTdfH_MC58 | 64 | STRQDIFKSSENLDNIVRSIPGAFTQQDKSSGIVSLNIRGDSGFGRVNTMVDGITQTFYSTSTDAGRAGG |
| NmTdfH_LNP21362 | 64 | STRQDIFKSSENLDNIVRSIPGAFTQQDKSSGIVSLNIRGDSGFGRVNTMVDGITQTFYSTSTDAGRAGG |
| NmTdfH_IR1074 | 64 | STRQDIFKSSENLDNIVRSIPGAFTQQDKSSGIVSLNIRGDSGFGRVNTMVDGITQTFYSTSTDAGRAGG |
| NmTdfH_H44/76 | 64 | STRQDIFKSSENLDNIVRSIPGAFTQQDKSSGIVSLNIRGDSGFGRVNTMVDGITQTFYSTSTDAGRAGG |
| NmTdfH_FDAARGOS_210 | 64 | STRQDIFKSSENLDNIVRSIPGAFTQQDKSSGIVSLNIRGDSGFGRVNTMVDGITQTFYSTSTDAGRAGG |
| NmTdfH_FDAARGOS_215 | 64 | STRQDIFKSSENLDNIVRSIPGAFTQQDKSSGIVSLNIRGDSGFGRVNTMVDGITQTFYSTSTDAGRAGG |
| NmTdfH_ | 64 | STRQDIFKSSENLDNIVRSIPGAFTQQDKSSGIVSLNIRGDSGFGRVNTMVDGITQTFYSTSTDAGRAGG |
| NmTdfH_510612 | 47 | STRQDIFKSSENLDNIVRSIPGAFTQQDKSSGIVSLNIRGDSGFGRVNTMVDGITQTFYSTSTDAGRAGG |
| NmTdfH_Z2491 | 64 | STRQDIFKSSENLDNIVRSIPGAFTQQDKSSGIVSLNIRGDSGFGRVNTMVDGITQTFYSTSTDAGRAGG |
| NmTdfH_NCTC8249 | 64 | STRQDIFKSSENLDNIVRSIPGAFTQQDKSSGIVSLNIRGDSGFGRVNTMVDGITQTFYSTSTDAGRAGG |
| NmTdfH_alpha14 | 64 | STRQDIFKSSENLDNIVRSIPGAFTQQDKSSGIVSLNIRGDSGFGRVNTMVDGITQTFYSTSTDAGRAGG |
| NmTdfH_2842STDY5881378 | 64 | STRQDIFKSSENLDNIVRSIPGAFTQQDKSSGIVSLNIRGDSGFGRVNTMVDGITQTFYSTSTDAGRAGG |
| NmTdfH_CHUV | 64 | STRQDIFKSSENLDNIVRSIPGAFTQQDKSSGIVSLNIRGDSGFGRVNTMVDGITQTFYSTSTDAGRAGG |
| NmTdfH_FAM18 | 64 | STRQDIFKSSENLDNIVRSIPGAFTQQDKSSGIVSLNIRGDSGFGRVNTMVDGITQTFYSTSTDAGRAGG |
| NmTdfH_M09293 | 64 | STRQDIFKSSENLDNIVRSIPGAFTQQDKSSGIVSLNIRGDSGFGRVNTMVDGITQTFYSTSTDAGRAGG |
| NmTdfH_M26503 | 64 | STRQDIFKSSENLDNIVRSIPGAFTQQDKSSGIVSLNIRGDSGFGRVNTMVDGITQTFYSTSTDAGRAGG |
| NmTdfH_2842STDY5881093 | 64 | STRQDIFKSSENLDNIVRSIPGAFTQQDKSSGIVSLNIRGDSGFGRVNTMVDGITQTFYSTSTDAGRAGG |
| NmTdfH_PMB5301 | 64 | STRQDIFKSSENLDNIVRSIPGAFTQQDKSSGIVSLNIRGDSGFGRVNTMVDGITQTFYSTSTDAGRAGG |

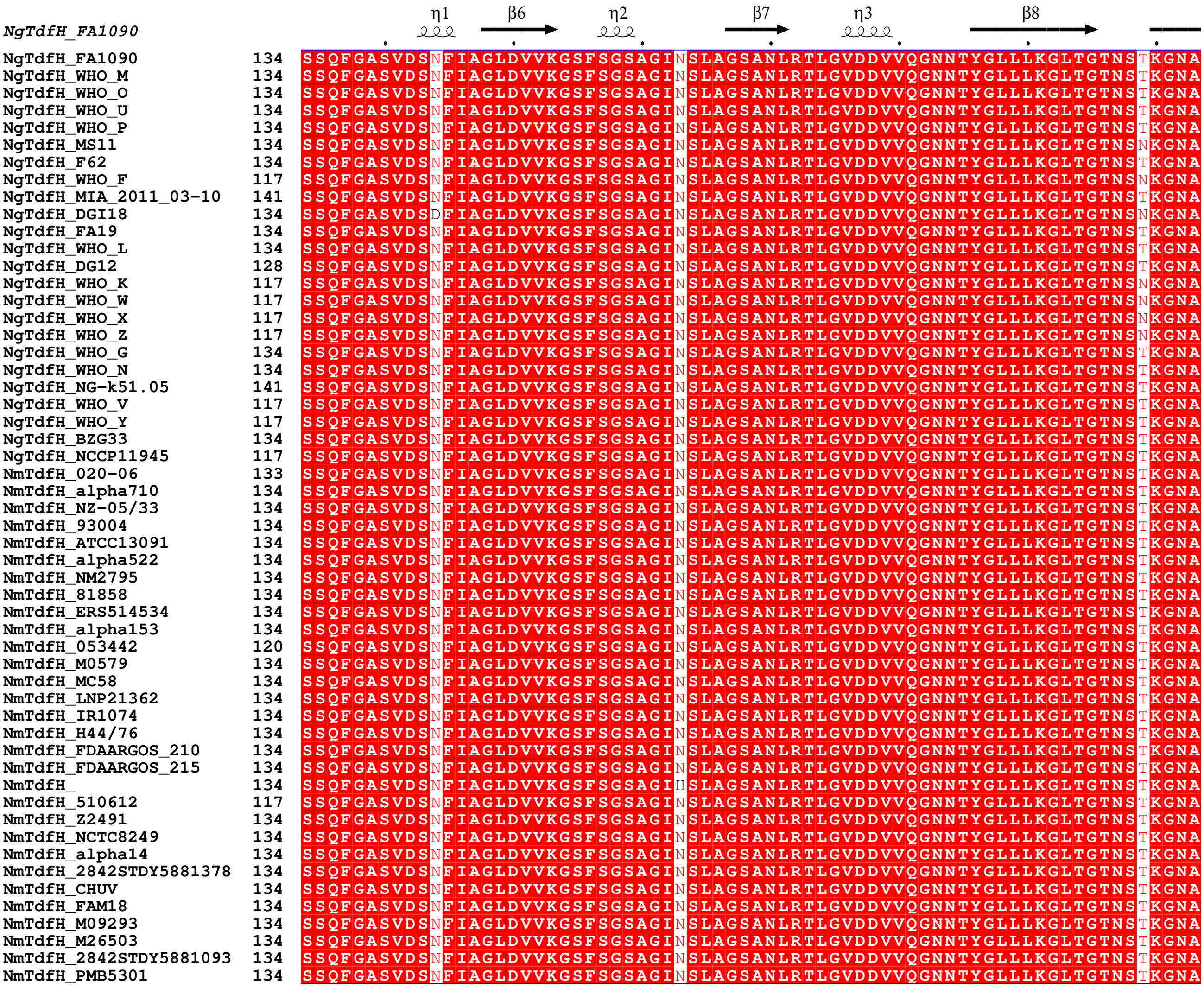
NgTdfH_FA1090
η1
β6
η2
β7
η3
β8
NgTdfH_FA1090 134 SSQFGASVDSNFIAGLDVVKGSFSGSAGINSLAGSANLRTLGVDDVVQGNNTYGLLLKGLTGTNSTKGNA
NgTdfH_WHO_M 134 SSQFGASVDSNFIAGLDVVKGSFSGSAGINSLAGSANLRTLGVDDVVQGNNTYGLLLKGLTGTNSTKGNA
NgTdfH_WHO_O 134 SSQFGASVDSNFIAGLDVVKGSFSGSAGINSLAGSANLRTLGVDDVVQGNNTYGLLLKGLTGTNSTKGNA
NgTdfH_WHO_U 134 SSQFGASVDSNFIAGLDVVKGSFSGSAGINSLAGSANLRTLGVDDVVQGNNTYGLLLKGLTGTNSTKGNA
NgTdfH_WHO_P 134 SSQFGASVDSNFIAGLDVVKGSFSGSAGINSLAGSANLRTLGVDDVVQGNNTYGLLLKGLTGTNSTKGNA
NgTdfH_MS11 134 SSQFGASVDSNFIAGLDVVKGSFSGSAGINSLAGSANLRTLGVDDVVQGNNTYGLLLKGLTGTNSNKGNA
NgTdfH_F62 134 SSQFGASVDSNFIAGLDVVKGSFSGSAGINSLAGSANLRTLGVDDVVQGNNTYGLLLKGLTGTNSTKGNA
NgTdfH_WHO_F 117 SSQFGASVDSNFIAGLDVVKGSFSGSAGINSLAGSANLRTLGVDDVVQGNNTYGLLLKGLTGTNSNKGNA
NgTdfH_MIA_2011_03-10 141 SSQFGASVDSNFIAGLDVVKGSFSGSAGINSLAGSANLRTLGVDDVVQGNNTYGLLLKGLTGTNSTKGNA
NgTdfH_DGI18 134 SSQFGASVDSDFIAGLDVVKGSFSGSAGINSLAGSANLRTLGVDDVVQGNNTYGLLLKGLTGTNSNKGNA
NgTdfH_FA19 134 SSQFGASVDSNFIAGLDVVKGSFSGSAGINSLAGSANLRTLGVDDVVQGNNTYGLLLKGLTGTNSTKGNA
NgTdfH_WHO_L 134 SSQFGASVDSNFIAGLDVVKGSFSGSAGINSLAGSANLRTLGVDDVVQGNNTYGLLLKGLTGTNSTKGNA
NgTdfH_DG12 128 SSQFGASVDSNFIAGLDVVKGSFSGSAGINSLAGSANLRTLGVDDVVQGNNTYGLLLKGLTGTNSTKGNA
NgTdfH_WHO_K 117 SSQFGASVDSNFIAGLDVVKGSFSGSAGINSLAGSANLRTLGVDDVVQGNNTYGLLLKGLTGTNSNKGNA
NgTdfH_WHO_W 117 SSQFGASVDSNFIAGLDVVKGSFSGSAGINSLAGSANLRTLGVDDVVQGNNTYGLLLKGLTGTNSNKGNA
NgTdfH_WHO_X 117 SSQFGASVDSNFIAGLDVVKGSFSGSAGINSLAGSANLRTLGVDDVVQGNNTYGLLLKGLTGTNSNKGNA
NgTdfH_WHO_Z 117 SSQFGASVDSNFIAGLDVVKGSFSGSAGINSLAGSANLRTLGVDDVVQGNNTYGLLLKGLTGTNSNKGNA
NgTdfH_WHO_G 134 SSQFGASVDSNFIAGLDVVKGSFSGSAGINSLAGSANLRTLGVDDVVQGNNTYGLLLKGLTGTNSTKGNA
NgTdfH_WHO_N 134 SSQFGASVDSNFIAGLDVVKGSFSGSAGINSLAGSANLRTLGVDDVVQGNNTYGLLLKGLTGTNSTKGNA
NgTdfH_NG-k51.05 141 SSQFGASVDSNFIAGLDVVKGSFSGSAGINSLAGSANLRTLGVDDVVQGNNTYGLLLKGLTGTNSTKGNA
NgTdfH_WHO_V 117 SSQFGASVDSNFIAGLDVVKGSFSGSAGINSLAGSANLRTLGVDDVVQGNNTYGLLLKGLTGTNSTKGNA
NgTdfH_WHO_Y 117 SSQFGASVDSNFIAGLDVVKGSFSGSAGINSLAGSANLRTLGVDDVVQGNNTYGLLLKGLTGTNSTKGNA
NgTdfH_BZG33 134 SSQFGASVDSNFIAGLDVVKGSFSGSAGINSLAGSANLRTLGVDDVVQGNNTYGLLLKGLTGTNSTKGNA
NgTdfH_NCCP11945 117 SSQFGASVDSNFIAGLDVVKGSFSGSAGINSLAGSANLRTLGVDDVVQGNNTYGLLLKGLTGTNSTKGNA
NmTdfH_020-06 133 SSQFGASVDSNFIAGLDVVKGSFSGSAGINSLAGSANLRTLGVDDVVQGNNTYGLLLKGLTGTNSTKGNA
NmTdfH_alpha710 134 SSQFGASVDSNFIAGLDVVKGSFSGSAGINSLAGSANLRTLGVDDVVQGNNTYGLLLKGLTGTNSTKGNA
NmTdfH_NZ-05/33 134 SSQFGASVDSNFIAGLDVVKGSFSGSAGINSLAGSANLRTLGVDDVVQGNNTYGLLLKGLTGTNSTKGNA
NmTdfH_93004 134 SSQFGASVDSNFIAGLDVVKGSFSGSAGINSLAGSANLRTLGVDDVVQGNNTYGLLLKGLTGTNSTKGNA
NmTdfH_ATCC13091 134 SSQFGASVDSNFIAGLDVVKGSFSGSAGINSLAGSANLRTLGVDDVVQGNNTYGLLLKGLTGTNSTKGNA
NmTdfH_alpha522 134 SSQFGASVDSNFIAGLDVVKGSFSGSAGINSLAGSANLRTLGVDDVVQGNNTYGLLLKGLTGTNSTKGNA
NmTdfH_NM2795 134 SSQFGASVDSNFIAGLDVVKGSFSGSAGINSLAGSANLRTLGVDDVVQGNNTYGLLLKGLTGTNSTKGNA
NmTdfH_81858 134 SSQFGASVDSNFIAGLDVVKGSFSGSAGINSLAGSANLRTLGVDDVVQGNNTYGLLLKGLTGTNSTKGNA
NmTdfH_ERS514534 134 SSQFGASVDSNFIAGLDVVKGSFSGSAGINSLAGSANLRTLGVDDVVQGNNTYGLLLKGLTGTNSTKGNA
NmTdfH_alpha153 134 SSQFGASVDSNFIAGLDVVKGSFSGSAGINSLAGSANLRTLGVDDVVQGNNTYGLLLKGLTGTNSTKGNA
NmTdfH_053442 120 SSQFGASVDSNFIAGLDVVKGSFSGSAGINSLAGSANLRTLGVDDVVQGNNTYGLLLKGLTGTNSTKGNA
NmTdfH_M0579 134 SSQFGASVDSNFIAGLDVVKGSFSGSAGINSLAGSANLRTLGVDDVVQGNNTYGLLLKGLTGTNSTKGNA
NmTdfH_MC58 134 SSQFGASVDSNFIAGLDVVKGSFSGSAGINSLAGSANLRTLGVDDVVQGNNTYGLLLKGLTGTNSTKGNA
NmTdfH_LNP21362 134 SSQFGASVDSNFIAGLDVVKGSFSGSAGINSLAGSANLRTLGVDDVVQGNNTYGLLLKGLTGTNSTKGNA
NmTdfH_IR1074 134 SSQFGASVDSNFIAGLDVVKGSFSGSAGINSLAGSANLRTLGVDDVVQGNNTYGLLLKGLTGTNSTKGNA
NmTdfH_H44/76 134 SSQFGASVDSNFIAGLDVVKGSFSGSAGINSLAGSANLRTLGVDDVVQGNNTYGLLLKGLTGTNSTKGNA
NmTdfH_FDAARGOS_210 134 SSQFGASVDSNFIAGLDVVKGSFSGSAGINSLAGSANLRTLGVDDVVQGNNTYGLLLKGLTGTNSTKGNA
NmTdfH_FDAARGOS_215 134 SSQFGASVDSNFIAGLDVVKGSFSGSAGINSLAGSANLRTLGVDDVVQGNNTYGLLLKGLTGTNSTKGNA
NmTdfH_ 134 SSQFGASVDSNFIAGLDVVKGSFSGSAGIHSLAGSANLRTLGVDDVVQGNNTYGLLLKGLTGTNSTKGNA
NmTdfH_510612 117 SSQFGASVDSNFIAGLDVVKGSFSGSAGINSLAGSANLRTLGVDDVVQGNNTYGLLLKGLTGTNSTKGNA
NmTdfH_Z2491 134 SSQFGASVDSNFIAGLDVVKGSFSGSAGINSLAGSANLRTLGVDDVVQGNNTYGLLLKGLTGTNSTKGNA
NmTdfH_NCTC8249 134 SSQFGASVDSNFIAGLDVVKGSFSGSAGINSLAGSANLRTLGVDDVVQGNNTYGLLLKGLTGTNSTKGNA
NmTdfH_alpha14 134 SSQFGASVDSNFIAGLDVVKGSFSGSAGINSLAGSANLRTLGVDDVVQGNNTYGLLLKGLTGTNSTKGNA
NmTdfH_2842STDY5881378 134 SSQFGASVDSNFIAGLDVVKGSFSGSAGINSLAGSANLRTLGVDDVVQGNNTYGLLLKGLTGTNSTKGNA
NmTdfH_CHUV 134 SSQFGASVDSNFIAGLDVVKGSFSGSAGINSLAGSANLRTLGVDDVVQGNNTYGLLLKGLTGTNSTKGNA
NmTdfH_FAM18 134 SSQFGASVDSNFIAGLDVVKGSFSGSAGINSLAGSANLRTLGVDDVVQGNNTYGLLLKGLTGTNSTKGNA
NmTdfH_M09293 134 SSQFGASVDSNFIAGLDVVKGSFSGSAGINSLAGSANLRTLGVDDVVQGNNTYGLLLKGLTGTNSTKGNA
NmTdfH_M26503 134 SSQFGASVDSNFIAGLDVVKGSFSGSAGINSLAGSANLRTLGVDDVVQGNNTYGLLLKGLTGTNSTKGNA
NmTdfH_2842STDY5881093 134 SSQFGASVDSNFIAGLDVVKGSFSGSAGINSLAGSANLRTLGVDDVVQGNNTYGLLLKGLTGTNSTKGNA
NmTdfH_PMB5301 134 SSQFGASVDSNFIAGLDVVKGSFSGSAGINSLAGSANLRTLGVDDVVQGNNTYGLLLKGLTGTNSTKGNA

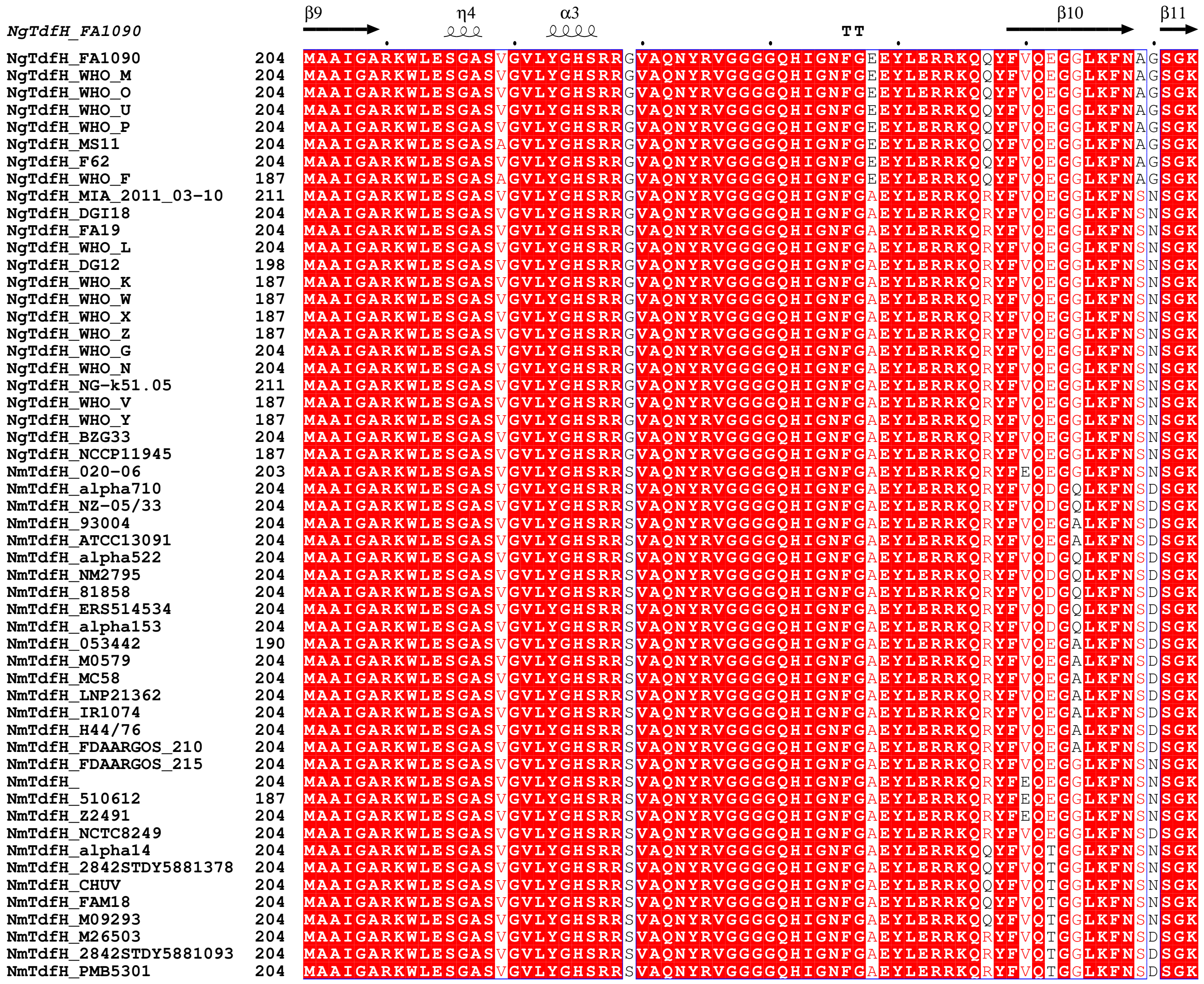
NgTdfH_FA1090
β9 η4 α3 TT β10 β11
NgTdfH_FA1090 204 MAAIGARKWLESGASVGVLYGHSRRGVAQNYRVGGGGQHIGNFGEEYLERRKQQYFVQEGGLKFNAGSGK
NgTdfH_WHO_M 204 MAAIGARKWLESGASVGVLYGHSRRGVAQNYRVGGGGQHIGNFGEEYLERRKQQYFVQEGGLKFNAGSGK
NgTdfH_WHO_O 204 MAAIGARKWLESGASVGVLYGHSRRGVAQNYRVGGGGQHIGNFGEEYLERRKQQYFVQEGGLKFNAGSGK
NgTdfH_WHO_U 204 MAAIGARKWLESGASVGVLYGHSRRGVAQNYRVGGGGQHIGNFGEEYLERRKQQYFVQEGGLKFNAGSGK
NgTdfH_WHO_P 204 MAAIGARKWLESGASVGVLYGHSRRGVAQNYRVGGGGQHIGNFGEEYLERRKQQYFVQEGGLKFNAGSGK
NgTdfH_MS11 204 MAAIGARKWLESGASAGVLYGHSRRGVAQNYRVGGGGQHIGNFGEEYLERRKQQYFVQEGGLKFNAGSGK
NgTdfH_F62 204 MAAIGARKWLESGASVGVLYGHSRRGVAQNYRVGGGGQHIGNFGEEYLERRKQQYFVQEGGLKFNAGSGK
NgTdfH_WHO_F 187 MAAIGARKWLESGASAGVLYGHSRRGVAQNYRVGGGGQHIGNFGEEYLERRKQQYFVQEGGLKFNAGSGK
NgTdfH_MIA_2011_03-10 211 MAAIGARKWLESGASVGVLYGHSRRGVAQNYRVGGGGQHIGNFGAEYLERRKQRYFVQEGGLKFNSNSGK
NgTdfH_DGI18 204 MAAIGARKWLESGASVGVLYGHSRRGVAQNYRVGGGGQHIGNFGAEYLERRKQRYFVQEGGLKFNSNSGK
NgTdfH_FA19 204 MAAIGARKWLESGASVGVLYGHSRRGVAQNYRVGGGGQHIGNFGAEYLERRKQRYFVQEGGLKFNSNSGK
NgTdfH_WHO_L 204 MAAIGARKWLESGASVGVLYGHSRRGVAQNYRVGGGGQHIGNFGAEYLERRKQRYFVQEGGLKFNSNSGK
NgTdfH_DG12 198 MAAIGARKWLESGASVGVLYGHSRRGVAQNYRVGGGGQHIGNFGAEYLERRKQRYFVQEGGLKFNSNSGK
NgTdfH_WHO_K 187 MAAIGARKWLESGASVGVLYGHSRRGVAQNYRVGGGGQHIGNFGAEYLERRKQRYFVQEGGLKFNSNSGK
NgTdfH_WHO_W 187 MAAIGARKWLESGASVGVLYGHSRRGVAQNYRVGGGGQHIGNFGAEYLERRKQRYFVQEGGLKFNSNSGK
NgTdfH_WHO_X 187 MAAIGARKWLESGASVGVLYGHSRRGVAQNYRVGGGGQHIGNFGAEYLERRKQRYFVQEGGLKFNSNSGK
NgTdfH_WHO_Z 187 MAAIGARKWLESGASVGVLYGHSRRGVAQNYRVGGGGQHIGNFGAEYLERRKQRYFVQEGGLKFNSNSGK
NgTdfH_WHO_G 204 MAAIGARKWLESGASVGVLYGHSRRGVAQNYRVGGGGQHIGNFGAEYLERRKQRYFVQEGGLKFNSNSGK
NgTdfH_WHO_N 204 MAAIGARKWLESGASVGVLYGHSRRGVAQNYRVGGGGQHIGNFGAEYLERRKQRYFVQEGGLKFNSNSGK
NgTdfH_NG-k51.05 211 MAAIGARKWLESGASVGVLYGHSRRGVAQNYRVGGGGQHIGNFGAEYLERRKQRYFVQEGGLKFNSNSGK
NgTdfH_WHO_V 187 MAAIGARKWLESGASVGVLYGHSRRGVAQNYRVGGGGQHIGNFGAEYLERRKQRYFVQEGGLKFNSNSGK
NgTdfH_WHO_Y 187 MAAIGARKWLESGASVGVLYGHSRRGVAQNYRVGGGGQHIGNFGAEYLERRKQRYFVQEGGLKFNSNSGK
NgTdfH_BZG33 204 MAAIGARKWLESGASVGVLYGHSRRGVAQNYRVGGGGQHIGNFGAEYLERRKQRYFVQEGGLKFNSNSGK
NgTdfH_NCCP11945 187 MAAIGARKWLESGASVGVLYGHSRRGVAQNYRVGGGGQHIGNFGAEYLERRKQRYFVQEGGLKFNSNSGK
NmTdfH_020-06 203 MAAIGARKWLESGASVGVLYGHSRRSVAQNYRVGGGGQHIGNFGAEYLERRKQRYFEQEGGLKFNSNSGK
NmTdfH_alpha710 204 MAAIGARKWLESGASVGVLYGHSRRSVAQNYRVGGGGQHIGNFGAEYLERRKQRYFVQDGQLKFNSDSGK
NmTdfH_NZ-05/33 204 MAAIGARKWLESGASVGVLYGHSRRSVAQNYRVGGGGQHIGNFGAEYLERRKQRYFVQDGQLKFNSDSGK
NmTdfH_93004 204 MAAIGARKWLESGASVGVLYGHSRRSVAQNYRVGGGGQHIGNFGAEYLERRKQRYFVQEGALKFNSDSGK
NmTdfH_ATCC13091 204 MAAIGARKWLESGASVGVLYGHSRRSVAQNYRVGGGGQHIGNFGAEYLERRKQRYFVQEGALKFNSDSGK
NmTdfH_alpha522 204 MAAIGARKWLESGASVGVLYGHSRRSVAQNYRVGGGGQHIGNFGAEYLERRKQRYFVQDGQLKFNSDSGK
NmTdfH_NM2795 204 MAAIGARKWLESGASVGVLYGHSRRSVAQNYRVGGGGQHIGNFGAEYLERRKQRYFVQDGQLKFNSDSGK
NmTdfH_81858 204 MAAIGARKWLESGASVGVLYGHSRRSVAQNYRVGGGGQHIGNFGAEYLERRKQRYFVQDGQLKFNSDSGK
NmTdfH_ERS514534 204 MAAIGARKWLESGASVGVLYGHSRRSVAQNYRVGGGGQHIGNFGAEYLERRKQRYFVQDGQLKFNSDSGK
NmTdfH_alpha153 204 MAAIGARKWLESGASVGVLYGHSRRSVAQNYRVGGGGQHIGNFGAEYLERRKQRYFVQDGQLKFNSDSGK
NmTdfH_053442 190 MAAIGARKWLESGASVGVLYGHSRRSVAQNYRVGGGGQHIGNFGAEYLERRKQRYFVQEGALKFNSDSGK
NmTdfH_M0579 204 MAAIGARKWLESGASVGVLYGHSRRSVAQNYRVGGGGQHIGNFGAEYLERRKQRYFVQEGALKFNSDSGK
NmTdfH_MC58 204 MAAIGARKWLESGASVGVLYGHSRRSVAQNYRVGGGGQHIGNFGAEYLERRKQRYFVQEGALKFNSDSGK
NmTdfH_LNP21362 204 MAAIGARKWLESGASVGVLYGHSRRSVAQNYRVGGGGQHIGNFGAEYLERRKQRYFVQEGALKFNSDSGK
NmTdfH_IR1074 204 MAAIGARKWLESGASVGVLYGHSRRSVAQNYRVGGGGQHIGNFGAEYLERRKQRYFVQEGALKFNSDSGK
NmTdfH_H44/76 204 MAAIGARKWLESGASVGVLYGHSRRSVAQNYRVGGGGQHIGNFGAEYLERRKQRYFVQEGALKFNSDSGK
NmTdfH_FDAARGOS_210 204 MAAIGARKWLESGASVGVLYGHSRRSVAQNYRVGGGGQHIGNFGAEYLERRKQRYFVQEGALKFNSDSGK
NmTdfH_FDAARGOS_215 204 MAAIGARKWLESGASVGVLYGHSRRSVAQNYRVGGGGQHIGNFGAEYLERRKQRYFVQEGGLKFNSNSGK
NmTdfH_ 204 MAAIGARKWLESGASVGVLYGHSRRSVAQNYRVGGGGQHIGNFGAEYLERRKQRYFEQEGGLKFNSNSGK
NmTdfH_510612 187 MAAIGARKWLESGASVGVLYGHSRRSVAQNYRVGGGGQHIGNFGAEYLERRKQRYFEQEGGLKFNSNSGK
NmTdfH_Z2491 204 MAAIGARKWLESGASVGVLYGHSRRSVAQNYRVGGGGQHIGNFGAEYLERRKQRYFEQEGGLKFNSNSGK
NmTdfH_NCTC8249 204 MAAIGARKWLESGASVGVLYGHSRRSVAQNYRVGGGGQHIGNFGAEYLERRKQRYFVQEGGLKFNSDSGK
NmTdfH_alpha14 204 MAAIGARKWLESGASVGVLYGHSRRSVAQNYRVGGGGQHIGNFGAEYLERRKQQYFVQTGGLKFNSNSGK
NmTdfH_2842STDY5881378 204 MAAIGARKWLESGASVGVLYGHSRRSVAQNYRVGGGGQHIGNFGAEYLERRKQQYFVQTGGLKFNSNSGK
NmTdfH_CHUV 204 MAAIGARKWLESGASVGVLYGHSRRSVAQNYRVGGGGQHIGNFGAEYLERRKQQYFVQTGGLKFNSNSGK
NmTdfH_FAM18 204 MAAIGARKWLESGASVGVLYGHSRRSVAQNYRVGGGGQHIGNFGAEYLERRKQQYFVQTGGLKFNSNSGK
NmTdfH_M09293 204 MAAIGARKWLESGASVGVLYGHSRRSVAQNYRVGGGGQHIGNFGAEYLERRKQQYFVQTGGLKFNSNSGK
NmTdfH_M26503 204 MAAIGARKWLESGASVGVLYGHSRRSVAQNYRVGGGGQHIGNFGAEYLERRKQRYFVQTGGLKFNSDSGK
NmTdfH_2842STDY5881093 204 MAAIGARKWLESGASVGVLYGHSRRSVAQNYRVGGGGQHIGNFGAEYLERRKQRYFVQTGGLKFNSDSGK
NmTdfH_PMB5301 204 MAAIGARKWLESGASVGVLYGHSRRSVAQNYRVGGGGQHIGNFGAEYLERRKQRYFVQTGGLKFNSDSGK

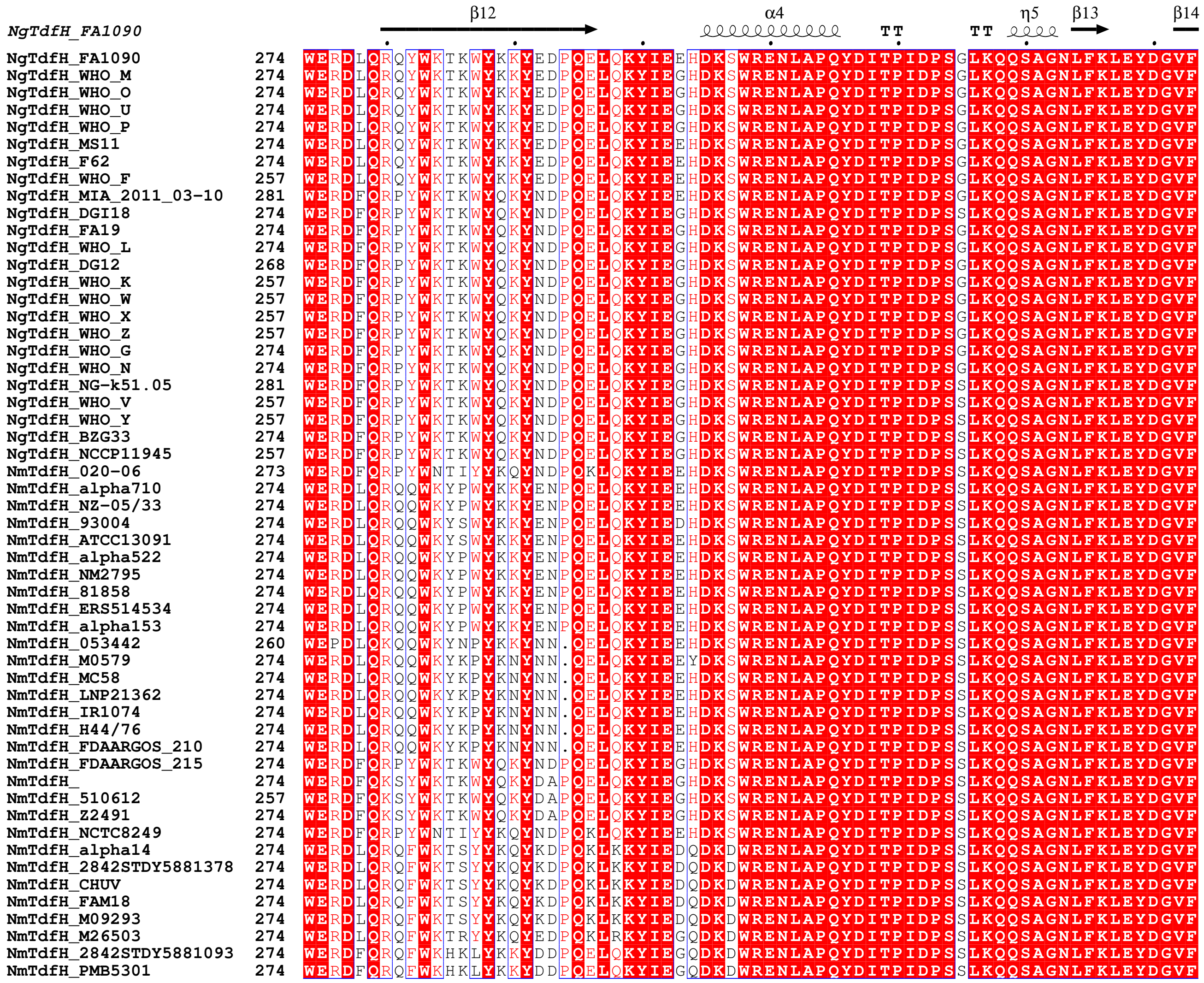

β12
α4
TT
TT
η5
β13
β14
NgTdfH_FA1090
NgTdfH_FA1090 274 WERDLQRQYWKTKWYKKYEDPQELQKYIEEHDKSWRENLAPQYDITPIDPSGLKQQSAGNLFKLEYDGVF
NgTdfH_WHO_M 274 WERDLQRQYWKTKWYKKYEDPQELQKYIEGHDKSWRENLAPQYDITPIDPSGLKQQSAGNLFKLEYDGVF
NgTdfH_WHO_O 274 WERDLQRQYWKTKWYKKYEDPQELQKYIEGHDKSWRENLAPQYDITPIDPSGLKQQSAGNLFKLEYDGVF
NgTdfH_WHO_U 274 WERDLQRQYWKTKWYKKYEDPQELQKYIEGHDKSWRENLAPQYDITPIDPSGLKQQSAGNLFKLEYDGVF
NgTdfH_WHO_P 274 WERDLQRQYWKTKWYKKYEDPQELQKYIEGHDKSWRENLAPQYDITPIDPSGLKQQSAGNLFKLEYDGVF
NgTdfH_MS11 274 WERDLQRQYWKTKWYKKYEDPQELQKYIEEHDKSWRENLAPQYDITPIDPSGLKQQSAGNLFKLEYDGVF
NgTdfH_F62 274 WERDLQRQYWKTKWYKKYEDPQELQKYIEEHDKSWRENLAPQYDITPIDPSGLKQQSAGNLFKLEYDGVF
NgTdfH_WHO_F 257 WERDLQRQYWKTKWYKKYEDPQELQKYIEEHDKSWRENLAPQYDITPIDPSGLKQQSAGNLFKLEYDGVF
NgTdfH_MIA_2011_03-10 281 WERDFQRPYWKTKWYQKYNDPQELQKYIEEHDKSWRENLAPQYDITPIDPSGLKQQSAGNLFKLEYDGVF
NgTdfH_DGI18 274 WERDFQRPYWKTKWYQKYNDPQELQKYIEGHDKSWRENLAPQYDITPIDPSSLKQQSAGNLFKLEYDGVF
NgTdfH_FA19 274 WERDFQRPYWKTKWYQKYNDPQELQKYIEGHDKSWRENLAPQYDITPIDPSGLKQQSAGNLFKLEYDGVF
NgTdfH_WHO_L 274 WERDFQRPYWKTKWYQKYNDPQELQKYIEGHDKSWRENLAPQYDITPIDPSGLKQQSAGNLFKLEYDGVF
NgTdfH_DG12 268 WERDFQRPYWKTKWYQKYNDPQELQKYIEGHDKSWRENLAPQYDITPIDPSGLKQQSAGNLFKLEYDGVF
NgTdfH_WHO_K 257 WERDFQRPYWKTKWYQKYNDPQELQKYIEGHDKSWRENLAPQYDITPIDPSGLKQQSAGNLFKLEYDGVF
NgTdfH_WHO_W 257 WERDFQRPYWKTKWYQKYNDPQELQKYIEGHDKSWRENLAPQYDITPIDPSGLKQQSAGNLFKLEYDGVF
NgTdfH_WHO_X 257 WERDFQRPYWKTKWYQKYNDPQELQKYIEGHDKSWRENLAPQYDITPIDPSGLKQQSAGNLFKLEYDGVF
NgTdfH_WHO_Z 257 WERDFQRPYWKTKWYQKYNDPQELQKYIEGHDKSWRENLAPQYDITPIDPSGLKQQSAGNLFKLEYDGVF
NgTdfH_WHO_G 274 WERDFQRPYWKTKWYQKYNDPQELQKYIEGHDKSWRENLAPQYDITPIDPSGLKQQSAGNLFKLEYDGVF
NgTdfH_WHO_N 274 WERDFQRPYWKTKWYQKYNDPQELQKYIEGHDKSWRENLAPQYDITPIDPSGLKQQSAGNLFKLEYDGVF
NgTdfH_NG-k51.05 281 WERDFQRPYWKTKWYQKYNDPQELQKYIEGHDKSWRENLAPQYDITPIDPSSLKQQSAGNLFKLEYDGVF
NgTdfH_WHO_V 257 WERDFQRPYWKTKWYQKYNDPQELQKYIEGHDKSWRENLAPQYDITPIDPSSLKQQSAGNLFKLEYDGVF
NgTdfH_WHO_Y 257 WERDFQRPYWKTKWYQKYNDPQELQKYIEGHDKSWRENLAPQYDITPIDPSSLKQQSAGNLFKLEYDGVF
NgTdfH_BZG33 274 WERDFQRPYWKTKWYQKYNDPQELQKYIEGHDKSWRENLAPQYDITPIDPSSLKQQSAGNLFKLEYDGVF
NgTdfH_NCCP11945 257 WERDFQRPYWKTKWYQKYNDPQELQKYIEGHDKSWRENLAPQYDITPIDPSSLKQQSAGNLFKLEYDGVF
NmTdfH_020-06 273 WERDFQRPYWNTIYYKQYNDPQKLQKYIEEHDKSWRENLAPQYDITPIDPSSLKQQSAGNLFKLEYDGVF
NmTdfH_alpha710 274 WERDLQRQQWKYPWYKKYENPQELQKYIEEHDKSWRENLAPQYDITPIDPSSLKQQSAGNLFKLEYDGVF
NmTdfH_NZ-05/33 274 WERDLQRQQWKYPWYKKYENPQELQKYIEEHDKSWRENLAPQYDITPIDPSSLKQQSAGNLFKLEYDGVF
NmTdfH_93004 274 WERDLQRQQWKYSWYKKYENPQELQKYIEDHDKSWRENLAPQYDITPIDPSSLKQQSAGNLFKLEYDGVF
NmTdfH_ATCC13091 274 WERDLQRQQWKYSWYKKYENPQELQKYIEEHDKSWRENLAPQYDITPIDPSSLKQQSAGNLFKLEYDGVF
NmTdfH_alpha522 274 WERDLQRQQWKYPWYKKYENPQELQKYIEEHDKSWRENLAPQYDITPIDPSSLKQQSAGNLFKLEYDGVF
NmTdfH_NM2795 274 WERDLQRQQWKYPWYKKYENPQELQKYIEEHDKSWRENLAPQYDITPIDPSSLKQQSAGNLFKLEYDGVF
NmTdfH_81858 274 WERDLQRQQWKYPWYKKYENPQELQKYIEEHDKSWRENLAPQYDITPIDPSSLKQQSAGNLFKLEYDGVF
NmTdfH_ERS514534 274 WERDLQRQQWKYPWYKKYENPQELQKYIEEHDKSWRENLAPQYDITPIDPSSLKQQSAGNLFKLEYDGVF
NmTdfH_alpha153 274 WERDLQRQQWKYPWYKKYENPQELQKYIEEHDKSWRENLAPQYDITPIDPSSLKQQSAGNLFKLEYDGVF
NmTdfH_053442 260 WEPDLQKQQWKYNPYKKYNN.QELQKYIEEHDKSWRENLAPQYDITPIDPSSLKQQSAGNLFKLEYDGVF
NmTdfH_M0579 274 WERDLQRQQWKYKPYKNYNN.QELQKYIEEYDKSWRENLAPQYDITPIDPSSLKQQSAGNLFKLEYDGVF
NmTdfH_MC58 274 WERDLQRQQWKYKPYKNYNN.QELQKYIEEHDKSWRENLAPQYDITPIDPSSLKQQSAGNLFKLEYDGVF
NmTdfH_LNP21362 274 WERDLQRQQWKYKPYKNYNN.QELQKYIEEHDKSWRENLAPQYDITPIDPSSLKQQSAGNLFKLEYDGVF
NmTdfH_IR1074 274 WERDLQRQQWKYKPYKNYNN.QELQKYIEEHDKSWRENLAPQYDITPIDPSSLKQQSAGNLFKLEYDGVF
NmTdfH_H44/76 274 WERDLQRQQWKYKPYKNYNN.QELQKYIEEHDKSWRENLAPQYDITPIDPSSLKQQSAGNLFKLEYDGVF
NmTdfH_FDAARGOS_210 274 WERDLQRQQWKYKPYKNYNN.QELQKYIEEHDKSWRENLAPQYDITPIDPSSLKQQSAGNLFKLEYDGVF
NmTdfH_FDAARGOS_215 274 WERDFQRPYWKTKWYQKYNDPQELQKYIEGHDKSWRENLAPQYDITPIDPSSLKQQSAGNLFKLEYDGVF
NmTdfH_ 274 WERDFQKSYWKTKWYQKYDAPQELQKYIEGHDKSWRENLAPQYDITPIDPSSLKQQSAGNLFKLEYDGVF
NmTdfH_510612 257 WERDFQKSYWKTKWYQKYDAPQELQKYIEGHDKSWRENLAPQYDITPIDPSSLKQQSAGNLFKLEYDGVF
NmTdfH_Z2491 274 WERDFQKSYWKTKWYQKYDAPQELQKYIEGHDKSWRENLAPQYDITPIDPSSLKQQSAGNLFKLEYDGVF
NmTdfH_NCTC8249 274 WERDFQRPYWNTIYYKQYNDPQKLQKYIEEHDKSWRENLAPQYDITPIDPSSLKQQSAGNLFKLEYDGVF
NmTdfH_alpha14 274 WERDLQRQFWKTSYYKQYKDPQKLKKYIEDQDKDWRENLAPQYDITPIDPSSLKQQSAGNLFKLEYDGVF
NmTdfH_2842STDY5881378 274 WERDLQRQFWKTSYYKQYKDPQKLKKYIEDQDKDWRENLAPQYDITPIDPSSLKQQSAGNLFKLEYDGVF
NmTdfH_CHUV 274 WERDLQRQFWKTSYYKQYKDPQKLKKYIEDQDKDWRENLAPQYDITPIDPSSLKQQSAGNLFKLEYDGVF
NmTdfH_FAM18 274 WERDLQRQFWKTSYYKQYKDPQKLKKYIEDQDKDWRENLAPQYDITPIDPSSLKQQSAGNLFKLEYDGVF
NmTdfH_M09293 274 WERDLQRQFWKTSYYKQYKDPQKLKKYIEDQDKDWRENLAPQYDITPIDPSSLKQQSAGNLFKLEYDGVF
NmTdfH_M26503 274 WERDLQRQFWKTRYYKQYEDPQKLRKYIEGQDKDWRENLAPQYDITPIDPSSLKQQSAGNLFKLEYDGVF
NmTdfH_2842STDY5881093 274 WERDFQRQFWKHKLYKKYDDPQELQKYIEGQDKDWRENLAPQYDITPIDPSSLKQQSAGNLFKLEYDGVF
NmTdfH_PMB5301 274 WERDFQRQFWKHKLYKKYDDPQELQKYIEGQDKDWRENLAPQYDITPIDPSSLKQQSAGNLFKLEYDGVF

*NgTdfH_FA1090* ▶TT β15 β16 β17 β18 T

| Sequence | Start | Alignment |
|---|---|---|
| NgTdfH_FA1090 | 344 | NKYTAQFRDLNTRIGSRKIINRNYQFNYGLSLNPYTNLNLTAAYNSGRQKYPKGAKFTGWGLLKDFETYN |
| NgTdfH_WHO_M | 344 | NKYTAQFRDLNTKIGSRKIINRNYQFNYGLSLNPYTNLNLTAAYNSGRQKYPKGSKFTGWGLLKDFETYN |
| NgTdfH_WHO_O | 344 | NKYTAQFRDLNTKIGSRKIINRNYQFNYGLSLNPYTNLNLTAAYNSGRQKYPKGSKFTGWGLLKDFETYN |
| NgTdfH_WHO_U | 344 | NKYTAQFRDLNTKIGSRKIINRNYQFNYGLSLNPYTNLNLTAAYNSGRQKYPKGSKFTGWGLLKDFETYN |
| NgTdfH_WHO_P | 344 | NKYTAQFRDLNTKIGSRKIINRNYQFNYGLSLNPYTNLNLTAAYNSGRQKYPKGSKFTGWGLLKDFETYN |
| NgTdfH_MS11 | 344 | NKYTAQFRDLNTKIGSRKIINRNYQFNYGLSLNPYTNLNLTAAYNSGRQKYPKGAKFTGWGLLKDFETYN |
| NgTdfH_F62 | 344 | NKYTAQFRDLNTRIGSRKIINRNYQFNYGLSLNPYTNLNLTAAYNSGRQKYPKGAKFTGWGLLKDFETYN |
| NgTdfH_WHO_F | 327 | NKYTAQFRDLNTRIGSRKIINRNYQFNYGLSLNPYTNLNLTAAYNSGRQKYPKGAKFTGWGLLKDFETYN |
| NgTdfH_MIA_2011_03-10 | 351 | NKYTAQFRDLNTRIGSRKIINRNYQFNYGLSLNPYTNLNLTAAYNSGRQKYPKGAKFTGWGLLKDFETYN |
| NgTdfH_DGI18 | 344 | NKYTAQFRDLNTKIGSRKIINRNYQFNYGLSLNPYTNLNLTAAYNSGRQKYPKGAKFTGWGLLKDFETYN |
| NgTdfH_FA19 | 344 | NKYTAQFRDLNTKIGSRKIINRNYQFNYGLSLNPYTNLNLTAAYNSGRQKYPKGSKFTGWGLLKDFETYN |
| NgTdfH_WHO_L | 344 | NKYTAQFRDLNTKIGSRKIINRNYQFNYGLSLNPYTNLNLTAAYNSGRQKYPKGSKFTGWGLLKDFETYN |
| NgTdfH_DG12 | 338 | NKYTAQFRDLNTKIGSRKIINRNYQFNYGLSLNPYTNLNLTAAYNSGRQKYPKGSKFTGWGLLKDFETYN |
| NgTdfH_WHO_K | 327 | NKYTAQFRDLNTRIGSRKIINRNYQFNYGLSLNPYTNLNLTAAYNSGRQKYPKGSKFTGWGLLKDFETYN |
| NgTdfH_WHO_W | 327 | NKYTAQFRDLNTRIGSRKIINRNYQFNYGLSLNPYTNLNLTAAYNSGRQKYPKGSKFTGWGLLKDFETYN |
| NgTdfH_WHO_X | 327 | NKYTAQFRDLNTRIGSRKIINRNYQFNYGLSLNPYTNLNLTAAYNSGRQKYPKGSKFTGWGLLKDFETYN |
| NgTdfH_WHO_Z | 327 | NKYTAQFRDLNTKIGSRKIINRNYQFNYGLSLNPYTNLNLTAAYNSGRQKYPKGSKFTGWGLLKDFETYN |
| NgTdfH_WHO_G | 344 | NKYTAQFRDLNTKIGSRKIINRNYQFNYGLSLNPYTNLNLTAAYNSGRQKYPKGSKFTGWGLLKDFETYN |
| NgTdfH_WHO_N | 344 | NKYTAQFRDLNTKIGSRKIINRNYQFNYGLSLNPYTNLNLTAAYNSGRQKYPKGSKFTGWGLLKDFETYN |
| NgTdfH_NG-k51.05 | 351 | NKYTAQFRDLNTKIGSRKIINRNYQFNYGLSLNPYTNLNLTAAYNSGRQKYPKGSKFTGWGLLKDFETYN |
| NgTdfH_WHO_V | 327 | NKYTAQFRDLNTKIGSRKIINRNYQFNYGLSLNPYTNLNLTAAYNSGRQKYPKGSKFTGWGLLKDFETYN |
| NgTdfH_WHO_Y | 327 | NKYTAQFRDLNTKIGSRKIINRNYQFNYGLSLNPYTNLNLTAAYNSGRQKYPKGSKFTGWGLLKDFETYN |
| NgTdfH_BZG33 | 344 | NKYTAQFRDLNTKIGSRKIINRNYQFNYGLSLNPYTNLNLTAAYNSGRQKYPKGSKFTGWGLLKDFETYN |
| NgTdfH_NCCP11945 | 327 | NKYTAQFRDLNTKIGSRKIINRNYQFNYGLSLNPYTNLNLTAAYNSGRQKYPKGSKFTGWGLLKDFETYN |
| NmTdfH_020-06 | 343 | NKYTAQFRDLNTKIGSRKIINRNYQFNYGLSLNPYANLNLTAAYNSGRQKYPKGAKFTGWGLLKDFETYN |
| NmTdfH_alpha710 | 344 | NKYTAQFRDLNTKIGSRKIINRNYQFNYGLSLNSYANLNLTAAYNSGRQKYPKGSKFTGWGLLKDFETYN |
| NmTdfH_NZ-05/33 | 344 | NKYTAQFRDLNTKIGSRKIINRNYQFNYGLSLNSYANLNLTAAYNSGRQKYPKGSKFTGWGLLKDFETYN |
| NmTdfH_93004 | 344 | NKYTAQFRDLNTKIGSRKIINRNYQFNYGLSLNSYTNLNLTAAYNSGRQKYPKGSKFTGWGLLKDFETYN |
| NmTdfH_ATCC13091 | 344 | NKYTAQFRDLNTKIGSRKIINRNYQFNYGLSLNSYTNLNLTAAYNSGRQKYPKGSKFTGWGLLKDFETYN |
| NmTdfH_alpha522 | 344 | NKYTAQFRDLNTKIGSRKIINRNYQFNYGLSLNSYTNLNLTAAYNSGRQKYPKGSKFTGWGLLKDFETYN |
| NmTdfH_NM2795 | 344 | NKYTAQFRDLNTKIGSRKIINRNYQFNYGLSLNSYTNLNLTAAYNSGRQKYPKGSKFTGWGLLKDFETYN |
| NmTdfH_81858 | 344 | NKYTAQFRDLNTKIGSRKIINRNYQFNYGLSLNSYANLNLTAAYNSGRQKYPKGSKFTGWGLLKDFETYN |
| NmTdfH_ERS514534 | 344 | NKYTAQFRDLNTKIGSRKIINRNYQFNYGLSLNSYANLNLTAAYNSGRQKYPKGSKFTGWGLLKDFETYN |
| NmTdfH_alpha153 | 344 | NKYTAQFRDLNTKIGSRKIINRNYQFNYGLSLNSYANLNLTAAYNSGRQKYPKGSKFTGWGLLKDFETYN |
| NmTdfH_053442 | 329 | NKYTAQFRDLNTKIGSRKIINRNYQFNYGLSLNSYANLNLTAAYNSGRQKYPKGSKFTGWGLLKDFETYN |
| NmTdfH_M0579 | 343 | NKYTAQFRDLNTKIGSRKIINRNYQFNYGLSLNPYTNLNLTAAYNSGRQKYPKGSKFTGWGLLKDFETYN |
| NmTdfH_MC58 | 343 | NKYTAQFRDLNTKIGSRKIINRNYQFNYGLSLNPYTNLNLTAAYNSGRQKYPKGSKFTGWGLLKDFETYN |
| NmTdfH_LNP21362 | 343 | NKYTAQFRDLNTKIGSRKIINRNYQFNYGLSLNPYTNLNLTAAYNSGRQKYPKGSKFTGWGLLKDFETYN |
| NmTdfH_IR1074 | 343 | NKYTAQFRDLNTKIGSRKIINRNYQFNYGLSLNPYTNLNLTAAYNSGRQKYPKGSKFTGWGLLKDFETYN |
| NmTdfH_H44/76 | 343 | NKYTAQFRDLNTKIGSRKIINRNYQFNYGLSLNPYTNLNLTAAYNSGRQKYPKGSKFTGWGLLKDFETYN |
| NmTdfH_FDAARGOS_210 | 343 | NKYTAQFRDLNTKIGSRKIINRNYQFNYGLSLNPYTNLNLTAAYNSGRQKYPKGSKFTGWGLLKDFETYN |
| NmTdfH_FDAARGOS_215 | 344 | NKYTAQFRDLNTKIGSRKIINRNYQFNYGLSLNSYANLNLTAAYNSGRQKYPKGSKFTGWGLLKDFETYN |
| NmTdfH_ | 344 | NKYTAQFRDLNTKIGSRKIINRNYQFNYGLSLNPYTNLNLTAAYNSGRQKYPKGSKFTGWGLLKDFETYN |
| NmTdfH_510612 | 327 | NKYTAQFRDLNTKIGSRKIINRNYQFNYGLSLNPYTNLNLTAAYNSGRQKYPKGSKFTGWGLLKDFETYN |
| NmTdfH_Z2491 | 344 | NKYTAQFRDLNTKIGSRKIINRNYQFNYGLSLNPYTNLNLTAAYNSGRQKYPKGSKFTGWGLLKDFETYN |
| NmTdfH_NCTC8249 | 344 | NKYTAQFRDLNTKIGSRKIINRNYQFNYGLSLNPYTNLNLTAAYNSGRQKYPKGSKFTGWGLLKDFETYN |
| NmTdfH_alpha14 | 344 | NKYTAQFRDLNTKIGSRKIINRNYQFNYGLSLNPYTNLNLTAAYNSGRQKYPKGSKFTGWGLLKDFETYN |
| NmTdfH_2842STDY5881378 | 344 | NKYTAQFRDLNTKIGSRKIINRNYQFNYGLSLNSYTNLNLTAAYNSGRQKYPKESKFTGWGLLKDFETYN |
| NmTdfH_CHUV | 344 | NKYTAQFRDLNTKIGSRKIINRNYQFNYGLSLNSYTNLNLTAAYNSGRQKYPKGSKFTGWGLLKDFETYN |
| NmTdfH_FAM18 | 344 | NKYTAQFRDLNTKIGSRKIINRNYQFNYGLSLNSYTNLNLTAAYNSGRQKYPKGSKFTGWGLLKDFETYN |
| NmTdfH_M09293 | 344 | NKYTAQFRDLNTKIGSRKIINRNYQFNYGLSLNSYTNLNLTAAYNSGRQKYPKGSKFTGWGLLKDFETYN |
| NmTdfH_M26503 | 344 | NKYTAQFRDLNTKIGSRKIINRNYQFNYGLSLNSYANLNLTAAYNSGRQKYPKGSKFTGWGLLKDFETYN |
| NmTdfH_2842STDY5881093 | 344 | NKYTAQFRDLNTKIGSRKIINRNYQFNYGLSLNPYTNLNLTAAYNSGRQKYPKGSKFTGWGLLKDFETYN |
| NmTdfH_PMB5301 | 344 | NKYTAQFRDLNTKIGSRKIINRNYQFNYGLSLNSYANLNLTAAYNSGRQKYPKGSKFTGWGLLKDFETYN |

```
                                   β19          β20           β21         β22                  β23
*NgTdfH_FA1090*                T TT                              TT
NgTdfH_FA1090               414 NAKILDLNNTATFRLPRETELQTTLGFNYFHNEYGKNRFPEELGLFFDGPDQDNGLYSYLGRFKGDKGLL
NgTdfH_WHO_M                414 NAKILDLNNTATFRLPRETELQTTLGFNYFHNEYGKNRFPEELGLFFDGPDQDNGLYSYLGRFKGDKGLL
NgTdfH_WHO_O                414 NAKILDLNNTATFRLPRETELQTTLGFNYFHNEYGKNRFPEELGLFFDGPDQDNGLYSYLGRFKGDKGLL
NgTdfH_WHO_U                414 NAKILDLNNTATFRLPRETELQTTLGFNYFHNEYGKNRFPEELGLFFDGPDQDNGLYSYLGRFKGDKGLL
NgTdfH_WHO_P                414 NAKILDLNNTATFRLPRETELQTTLGFNYFHNEYGKNRFPEELGLFFDGPDQDNGLYSYLGRFKGDKGLL
NgTdfH_MS11                 414 NAKILDLNNTATFRLPRETELQTTLGFNYFHNEYGKNRFPEELGLFFDGPDQDNGLYSYLGRFKGDKGLL
NgTdfH_F62                  414 NAKILDLNNTATFRLPRETELQTTLGFNYFHNEYGKNRFPEELGLFFDGPDQDNGLYSYLGRFKGDKGLL
NgTdfH_WHO_F                397 NAKILDLNNTATFRLPRETELQTTLGFNYFHNEYGKNRFPEELGLFFDGPDQDNGLYSYLGRFKGDKGLL
NgTdfH_MIA_2011_03-10       421 NAKILDLNNTATFRLPRETELQTTLGFNYFHNEYGKNRFPEELGLFFDGPDQDNGLYSYLGRFKGDKGLL
NgTdfH_DGI18                414 NAKILDLNNTATFRLPRETELQTTLGFNYFHNEYGKNRFPEELGLFFDGPDQDNGLYSYLGRFKGDKGLL
NgTdfH_FA19                 414 NAKILDLNNTATFRLPRETELQTTLGFNYFHNEYGKNRFPEELGLFFDGPDQDNGLYSYLGRFKGDKGLL
NgTdfH_WHO_L                414 NAKILDLNNTATFRLPRETELQTTLGFNYFHNEYGKNRFPEELGLFFDGPDQDNGLYSYLGRFKGDKGLL
NgTdfH_DG12                 408 NAKILDLNNTATFRLPRETELQTTLGFNYFHNEYGKNRFPEELGLFFDGPDQDNGLYSYLGRFKGDKGLL
NgTdfH_WHO_K                397 NAKILDLNNTATFRLPRETELQTTLGFNYFHNEYGKNRFPEELGLFFDGPDQDNGLYSYLGRFKGDKGLL
NgTdfH_WHO_W                397 NAKILDLNNTATFRLPRETELQTTLGFNYFHNEYGKNRFPEELGLFFDGPDQDNGLYSYLGRFKGDKGLL
NgTdfH_WHO_X                397 NAKILDLNNTATFRLPRETELQTTLGFNYFHNEYGKNRFPEELGLFFDGPDQDNGLYSYLGRFKGDKGLL
NgTdfH_WHO_Z                397 NAKILDLNNTATFRLPRETELQTTLGFNYFHNEYGKNRFPEELGLFFDGPDQDNGLYSYLGRFKGDKGLL
NgTdfH_WHO_G                414 NAKILDLNNTATFRLPRETELQTTLGFNYFHNEYGKNRFPEELGLFFDGPDQDNGLYSYLGRFKGDKGLL
NgTdfH_WHO_N                414 NAKILDLNNTATFRLPRETELQTTLGFNYFHNEYGKNRFPEELGLFFDGPDQDNGLYSYLGRFKGDKGLL
NgTdfH_NG-k51.05            421 NAKILDLNNTATFRLPRETELQTTLGFNYFHNEYGKNRFPEELGLFFDGPDQDNGLYSYLGRFKGDKGLL
NgTdfH_WHO_V                397 NAKILDLNNTATFRLPRETELQTTLGFNYFHNEYGKNRFPEELGLFFDGPDQDNGLYSYLGRFKGDKGLL
NgTdfH_WHO_Y                397 NAKILDLNNTATFRLPRETELQTTLGFNYFHNEYGKNRFPEELGLFFDGPDQDNGLYSYLGRFKGDKGLL
NgTdfH_BZG33                414 NAKILDLNNTATFRLPRETELQTTLGFNYFHNEYGKNRFPEELGLFFDGPDQDNGLYSYLGRFKGDKGLL
NgTdfH_NCCP11945            397 NAKILDLNNTATFRLPRETELQTTLGFNYFHNEYGKNRFPEELGLFFDGPDQDNGLYSYLGRFKGDKGLL
NmTdfH_020-06               413 NAKILDLNNTATFRLPRETELQTTLGFNYFHNEYGKNRFPEELGLFFDGPDQDNGLYSYLGRFKGDKGLL
NmTdfH_alpha710             414 NAKILDLNNTATFRLPRETELQTTLGFNYFHNEYGKNRFPEELGLFFDGPDQDNGLYSYLGRFKGDKGLL
NmTdfH_NZ-05/33             414 NAKILDLNNTATFRLPRETELQTTLGFNYFHNEYGKNRFPEELGLFFDGPDQDNGLYSYLGRFKGDKGLL
NmTdfH_93004                414 NAKILDLNNTATFRLPRETELQTTLGFNYFHNEYGKNRFPEELGLFFDGPDQDNGLYSYLGRFKGDKGLL
NmTdfH_ATCC13091            414 NAKILDLNNTATFRLPRETELQTTLGFNYFHNEYGKNRFPEELGLFFDGPDQDNGLYSYLGRFKGDKGLL
NmTdfH_alpha522             414 NAKILDLNNTATFRLPRETELQTTLGFNYFHNEYGKNRFPEELGLFFDGPDQDNGLYSYLGRFKGDKGLL
NmTdfH_NM2795               414 NAKILDLNNTATFRLPRETELQTTLGFNYFHNEYGKNRFPEELGLFFDGPDQDNGLYSYLGRFKGDKGLL
NmTdfH_81858                414 NAKILDLNNTATFRLPRETELQTTLGFNYFHNEYGKNRFPEELGLFFDGPDQDNGLYSYLGRFKGDKGLL
NmTdfH_ERS514534            414 NAKILDLNNTATFRLPRETELQTTLGFNYFHNEYGKNRFPEELGLFFDGPDQDNGLYSYLGRFKGDKGLL
NmTdfH_alpha153             414 NAKILDLNNTATFRLPRETELQTTLGFNYFHNEYGKNRFPEELGLFFDGPDQDNGLYSYLGRFKGDKGLL
NmTdfH_053442               399 NAKILDLNNTATFRLPRETELQTTLGFNYFHNEYGKNRFPEELGLFFDGPDQDNGLYSYLGRFKGDKGLL
NmTdfH_M0579                413 NAKILDLNNTATFRLPRETELQTTLGFNYFHNEYGKNRFPEELGLFFDGPDQDNGLYSYLGRFKGDKGLL
NmTdfH_MC58                 413 NAKILDLNNTATFRLPRETELQTTLGFNYFHNEYGKNRFPEELGLFFDGPDQDNGLYSYLGRFKGDKGLL
NmTdfH_LNP21362             413 NAKILDLNNTATFRLPRETELQTTLGFNYFHNEYGKNRFPEELGLFFDGPDQDNGLYSYLGRFKGDKGLL
NmTdfH_IR1074               413 NAKILDLNNTATFRLPRETELQTTLGFNYFHNEYGKNRFPEELGLFFDGPDQDNGLYSYLGRFKGDKGLL
NmTdfH_H44/76               413 NAKILDLNNTATFRLPRETELQTTLGFNYFHNEYGKNRFPEELGLFFDGPDQDNGLYSYLGRFKGDKGLL
NmTdfH_FDAARGOS_210         413 NAKILDLNNTATFRLPRETELQTTLGFNYFHNEYGKNRFPEELGLFFDGPDQDNGLYSYLGRFKGDKGLL
NmTdfH_FDAARGOS_215         414 NAKILDLNNTATFRLPRETELQTTLGFNYFHNEYGKNRFPEELGLFFDGPDQDNGLYSYLGRFKGDKGLL
NmTdfH_                     414 NAKILDLNNTSTFRLPRETELQTTLGFNYFHNEYGKNRFPEELGLFFDGPDQDNGLYSYLGRFKGDKGLL
NmTdfH_510612               397 NAKILDLNNTSTFRLPRETELQTTLGFNYFHNEYGKNRFPEELGLFFDGPDQDNGLYSYLGRFKGDKGLL
NmTdfH_Z2491                414 NAKILDLNNTSTFRLPRETELQTTLGFNYFHNEYGKNRFPEELGLFFDGPDQDNGLYSYLGRFKGDKGLL
NmTdfH_NCTC8249             414 NAKILDLNNTATFRLPRETELQTTLGFNYFHNEYGKNRFPEELGLFFDGPDQDNGLYSYLGRFKGDKGLL
NmTdfH_alpha14              414 NAKILDLNNTATFRLPRETELQTTLGFNYFHNEYGKNRFPEELGLFFDGPDQDNGLYSYLGRFKGDKGLL
NmTdfH_2842STDY5881378      414 NAKILDLNNTATFRLPRETELQTTLGFNYFHNEYGKNRFPEELGLFFDGPDQDNGLYSYLGRFKGDKGLL
NmTdfH_CHUV                 414 NAKILDLNNTATFRLPRETELQTTLGFNYFHNEYGKNRFPEELGLFFDGPDQDNGLYSYLGRFKGDKGLL
NmTdfH_FAM18                414 NAKILDLNNTATFRLPRETELQTTLGFNYFHNEYGKNRFPEELGLFFDGPDQDNGLYSYLGRFKGDKGLL
NmTdfH_M09293               414 NAKILDLNNTATFRLPRETELQTTLGFNYFHNEYGKNRFPEELGLFFDGPDQDNGLYSYLGRFKGDKGLL
NmTdfH_M26503               414 NAKILDLNNTATFRLPRETELQTTLGFNYFHNEYGKNRFPEELGLFFDGPDQDNGLYSYLGRFKGDKGLL
NmTdfH_2842STDY5881093      414 NAKILDLNNTATFRLPRETELQTTLGFNYFHNEYGKNRFPEELGLFFDGPDQDNGLYSYLGRFKGDKGLL
NmTdfH_PMB5301              414 NAKILDLNNTATFRLPRETELQTTLGFNYFHNEYGKNRFPEELGLFFDGPDQDNGLYSYLGRFKGDKGLL
```

NgTdfH_FA1090 — η6 · β24 · TT · β25 · TTT · β26

| Sequence | Start | Alignment |
|---|---|---|
| NgTdfH_FA1090 | 484 | PQKSTIVQPAGSQYFNTFYFDAALKKDIYRLNYSTNAINYRFGGEYTGYYGSENEFKRAFGENSPAYKEH |
| NgTdfH_WHO_M | 484 | PQKSTIVQPAGSQYFNTFYFDAALKKDIYRLNYSTNAINYRFGGEYTGYYGSENEFKRAFGENSPAYKEH |
| NgTdfH_WHO_O | 484 | PQKSTIVQPAGSQYFNTFYFDAALKKDIYRLNYSTNAINYRFGGEYTGYYGSENEFKRAFGENSPAYKEH |
| NgTdfH_WHO_U | 484 | PQKSTIVQPAGSQYFNTFYFDAALKKDIYRLNYSTNAINYRFGGEYTGYYGSENEFKRAFGENSPAYKEH |
| NgTdfH_WHO_P | 484 | PQKSTIVQPAGSQYFNTFYFDAALKKDIYRLNYSTNAINYRFGGEYTGYYGSENEFKRAFGENSPAYKEH |
| NgTdfH_MS11 | 484 | PQKSTIVQPAGSQYFNTFYFDAALKKDIYRLNYSTNAINYRFGGEYTGYYGSESEFKRAFGENSPAYKEH |
| NgTdfH_F62 | 484 | PQKSTIVQPAGSQYFNTFYFDAALKKDIYRLNYSTNAINYRFGGEYTGYYGSENEFKRAFGENSPAYKEH |
| NgTdfH_WHO_F | 467 | PQKSTIVQPAGSQYFNTFYFDAALKKDIYRLNYSTNAINYRFGGEYTGYYGSENEFKRAFGENSPAYKEH |
| NgTdfH_MIA_2011_03-10 | 491 | PQKSTIVQPAGSQYFNTFYFDAALKKDIYRLNYSTNAINYRFGGEYTGYYGSENEFKRAFGENSPAYKEH |
| NgTdfH_DGI18 | 484 | PQKSTIVQPAGSQYFNTFYFDAALKKDIYRLNYSTNAINYRFGGEYTGYYGSENEFKRAFGENSPAYKEH |
| NgTdfH_FA19 | 484 | PQKSTIVQPAGSQYFNTFYFDAALKKDIYRLNYSTNAINYRFGGEYTGYYGSENEFKRAFGENSPAYKEH |
| NgTdfH_WHO_L | 484 | PQKSTIVQPAGSQYFNTFYFDAALKKDIYRLNYSTNAINYRFGGEYTGYYGSENEFKRAFGENSPAYKEH |
| NgTdfH_DG12 | 478 | PQKSTIVQPAGSQYFNTFYFDAALKKDIYRLNYSTNAINYRFGGEYTGYYGSENEFKRAFGENSPAYKEH |
| NgTdfH_WHO_K | 467 | PQKSTIVQPAGSQYFNTFYFDAALKKDIYRLNYSTNAINYRFGGEYTGYYGSENEFKRAFGENSPAYKEH |
| NgTdfH_WHO_W | 467 | PQKSTIVQPAGSQYFNTFYFDAALKKDIYRLNYSTNAINYRFGGEYTGYYGSENEFKRAFGENSPAYKEH |
| NgTdfH_WHO_X | 467 | PQKSTIVQPAGSQYFNTFYFDAALKKDIYRLNYSTNAINYRFGGEYTGYYGSENEFKRAFGENSPAYKEH |
| NgTdfH_WHO_Z | 467 | PQKSTIVQPAGSQYFNTFYFDAALKKDIYRLNYSTNAINYRFGGEYTGYYGSENEFKRAFGENSPAYKEH |
| NgTdfH_WHO_G | 484 | PQKSTIVQPAGSQYFNTFYFDAALKKDIYRLNYSTNAINYRFGGEYTGYYGSENEFKRAFGENSPAYKEH |
| NgTdfH_WHO_N | 484 | PQKSTIVQPAGSQYFNTFYFDAALKKDIYRLNYSTNAINYRFGGEYTGYYGSENEFKRAFGENSPAYKEH |
| NgTdfH_NG-k51.05 | 491 | PQKSTIVQPAGSQYFNTFYFDAALKKDIYRLNYSTNAINYRFGGEYTGYYGSENEFKRAFGENSPAYKEH |
| NgTdfH_WHO_V | 467 | PQKSTIVQPAGSQYFNTFYFDAALKKDIYRLNYSTNAINYRFGGEYTGYYGSENEFKRAFGENSPAYKEH |
| NgTdfH_WHO_Y | 467 | PQKSTIVQPAGSQYFNTFYFDAALKKDIYRLNYSTNAINYRFGGEYTGYYGSENEFKRAFGENSPAYKEH |
| NgTdfH_BZG33 | 484 | PQKSTIVQPAGSQYFNTFYFDAALKKDIYRLNYSTNAINYRFGGEYTGYYGSENEFKRAFGENSPAYKEH |
| NgTdfH_NCCP11945 | 467 | PQKSTIVQPAGSQYFNTFYFDAALKKDIYRLNYSTNAINYRFGGEYTGYYGSENEFKRAFGENSPAYKEH |
| NmTdfH_020-06 | 483 | PQKSTIVQPAGNQYFNTFYFDAALKKDIYRLNYSTNTINYRFGGEYTGYYSSENEFKRAFGENSPAYKKH |
| NmTdfH_alpha710 | 484 | PQKSTIVQPAGSQYFNTFYFDAALKKDIYRLNYSTNTVGYRFGGEYTGYYGSDDEFKRAFGENSPTYREH |
| NmTdfH_NZ-05/33 | 484 | PQKSTIVQPAGSQYFNTFYFDAALKKDIYRLNYSTNTVGYRFGGEYTGYYGSDDEFKRAFGENSPTYREH |
| NmTdfH_93004 | 484 | PQKSTIVQPAGSQYFNTFYFDAALKKDIYRLNYSTNTVGYRFGGEYTGYYSSEGEFKRAFGENSPTYREH |
| NmTdfH_ATCC13091 | 484 | PQKSTIVQPAGSQYFNTFYFDAALKKDIYRLNYSTNTVGYRFGGEYTGYYSSEGEFKRAFGENSPTYREH |
| NmTdfH_alpha522 | 484 | PQKSTIVQPAGSQYFNTFYFDAALKKDIYRLNYSTNTVGYRFGGEYTGYYSSEGEFKRAFGENSPTYKKH |
| NmTdfH_NM2795 | 484 | PQKSTIVQPAGSQYFNTFYFDAALKKDIYRLNYSTNTVGYRFGGEYTGYYSSEGEFKRAFGENSPTYKKH |
| NmTdfH_81858 | 484 | PQKSTIVQPAGSQYFNTFYFDAALKKDIYRLNYSTNTVGYRFGGEYTGYYSSEGEFKRAFGENSPTYKKH |
| NmTdfH_ERS514534 | 484 | PQKSTIVQPAGSQYFNTFYFDAALKKDIYRLNYSTNTVGYRFGGEYTGYYSSEGEFKRAFGENSPTYKKH |
| NmTdfH_alpha153 | 484 | PQKSTIVQPAGSQYFNTFYFDAALKKDIYRLNYSTNTVGYRFGGEYTGYYSSEGEFKRAFGENSPTYKKH |
| NmTdfH_053442 | 469 | PQKSTIVQPAGSQYFNTFYFDAALKKDIYRLNYSTNTVGYRFGGEYTGYYSSEGEFKRAFGENSPTYKKH |
| NmTdfH_M0579 | 483 | PQKSTIVQPAGSQYFNTFYFDAALKKDIYRLNYSTNTVGYRFGGEYTGYYGSDDEFKRAFGENSPTYREH |
| NmTdfH_MC58 | 483 | PQKSTIVQPAGSQYFNTFYFDAALKKDIYRLNYSTNTVGYRFGGEYTGYYGSDDEFKRAFGENSPTYKKH |
| NmTdfH_LNP21362 | 483 | PQKSTIVQPAGSQYFNTFYFDAALKKDIYRLNYSTNTVGYRFGGEYTGYYGSDDEFKRAFGENSPTYKKH |
| NmTdfH_IR1074 | 483 | PQKSTIVQPAGSQYFNTFYFDAALKKDIYRLNYSTNTVGYRFGGEYTGYYGSDDEFKRAFGENSPTYKKH |
| NmTdfH_H44/76 | 483 | PQKSTIVQPAGSQYFNTFYFDAALKKDIYRLNYSTNTVGYRFGGEYTGYYGSDDEFKRAFGENSPTYKKH |
| NmTdfH_FDAARGOS_210 | 483 | PQKSTIVQPAGSQYFNTFYFDAALKKDIYRLNYSTNTVGYRFGGEYTGYYGSDDEFKRAFGENSPTYKKH |
| NmTdfH_FDAARGOS_215 | 484 | PQKSTIVQPAGSQYFNTFYFDAALKKDIYRLNYSTNTVGYRFGGEYTGYYGSDDEFKRAFGENSPTYKKH |
| NmTdfH_ | 484 | PQKSTIVQPAGSQYFNTFYFDAALKKDIYRLNYSTNTVGYRFGGEYTGYYGSDDEFKRAFGENSPTYKKH |
| NmTdfH_510612 | 467 | PQKSTIVQPAGSQYFNTFYFDAALKKDIYRLNYSTNTVGYRFGGEYTGYYGSDDEFKRAFGENSPTYKKH |
| NmTdfH_Z2491 | 484 | PQKSTIVQPAGSQYFNTFYFDAALKKDIYRLNYSTNTVGYRFGGEYTGYYGSDDEFKRAFGENSPTYKKH |
| NmTdfH_NCTC8249 | 484 | PQKSTIVQPAGSQYFNTFYFDAALKKDIYRLNYSTNTVGYRFGGEYTGYYGSDDEFKRAFGENSPTYKKH |
| NmTdfH_alpha14 | 484 | PQKSTIVQPAGSQYFNTFYFDAALKKDIYRLNYSTNTVGYRFGGEYTGYYGSEGEFKRAFGENSPIYREH |
| NmTdfH_2842STDY5881378 | 484 | PQKSTIVQPAGSQYFNTFYFDAALKKDIYRLNYSTNTVGYRFGGEYTGYYGSDDEFKRAFGENSPTYKKH |
| NmTdfH_CHUV | 484 | PQKSTIVQPAGSQYFNTFYFDAALKKDIYRLNYSTNTVGYRFGGEYTGYYSSEGEFKRAFGENSPTYKKH |
| NmTdfH_FAM18 | 484 | PQKSTIVQPAGSQYFNTFYFDAALKKDIYRLNYSTNTVGYRFGGEYTGYYSSEGEFKRAFGENSPTYKKH |
| NmTdfH_M09293 | 484 | PQKSTIVQPAGSQYFNTFYFDAALKKDIYRLNYSTNTVGYRFGGEYTGYYSSEGEFKRAFGENSPTYKKH |
| NmTdfH_M26503 | 484 | PQKSTIVQPAGSQYFNTFYFDAALKKDIYRLNYSTNTVGYRFGGEYTGYYGSDDEFKRAFGENSPTYKKH |
| NmTdfH_2842STDY5881093 | 484 | PQKSTIVQPAGSQYFNTFYFDAALKKDIYRLNYSTNTVGYRFGGEYTGYYGSDDEFKRAFGENSPTYKKH |
| NmTdfH_PMB5301 | 484 | PQKSTIVQPAGSQYFNTFYFDAALKKDIYRLNYSTNTVGYRFGGEYTGYYGSDDEFKRAFGENSPTYKKH |

*NgTdfH_FA1090* — β27 β28 β29 α5

| Sequence | Start | Alignment |
| --- | --- | --- |
| NgTdfH_FA1090 | 554 | CDPSCGLYEPVLKKYGKKRANNHSVSISADFGDYFMPFAGYSRTHRMPNIQEMYFSQIGDSGVHTALKPE |
| NgTdfH_WHO_M | 554 | CDPSCGLYEPVLKKYGKKRANNHSVSISADFGDYFMPFAGYSRTHRMPNIQEMYFSQIGDSGVHTALKPE |
| NgTdfH_WHO_O | 554 | CDPSCGLYEPVLKKYGKKRANNHSVSISADFGDYFMPFAGYSRTHRMPNIQEMYFSQIGDSGVHTALKPE |
| NgTdfH_WHO_U | 554 | CDPSCGLYEPVLKKYGKKRANNHSVSISADFGDYFMPFAGYSRTHRMPNIQEMYFSQIGDSGVHTALKPE |
| NgTdfH_WHO_P | 554 | CDPSCGLYEPVLKKYGKKRANNHSVSISADFGDYFMPFAGYSRTHRMPNIQEMYFSQIGDSGVHTALKPE |
| NgTdfH_MS11 | 554 | CDPSCGLYEPVLKKYGKKRANNHSVSISADFGDYFMPFAGYSRTHRMPNIQEMYFSQIGDSGVHTALKPE |
| NgTdfH_F62 | 554 | CDPSCGLYEPVLKKYGKKRANNHSVSISADFGDYFMPFAGYSRTHRMPNIQEMYFSQIGDSGVHTALKPE |
| NgTdfH_WHO_F | 537 | CDPSCGLYEPVLKKYGKKRANNHSVSISADFGDYFMPFAGYSRTHRMPNIQEMYFSQIGDSGVHTALKPE |
| NgTdfH_MIA_2011_03-10 | 561 | CDPSCGLYEPVLKKYGKKRANNHSVSISADFGDYFMPFAGYSRTHRMPNIQEMYFSQIGDSGVHTALKPE |
| NgTdfH_DGI18 | 554 | CDPSCGLYEPVLKKYGKKRANNHSVSISADFGDYFMPFAGYSRTHRMPNIQEMYFSQIGDSGVHTALKPE |
| NgTdfH_FA19 | 554 | CDPSCGLYEPVLKKYGKKRANNHSVSISADFGDYFMPFAGYSRTHRMPNIQEMYFSQIGDSGVHTALKPE |
| NgTdfH_WHO_L | 554 | CDPSCGLYEPVLKKYGKKRANNHSVSISADFGDYFMPFAGYSRTHRMPNIQEMYFSQIGDSGVHTALKPE |
| NgTdfH_DG12 | 548 | CDPSCGLYEPVLKKYGKKRANNHSVSISADFGDYFMPFAGYSRTHRMPNIQEMYFSQIGDSGVHTALKPE |
| NgTdfH_WHO_K | 537 | CDPSCGLYEPVLKKYGKKRANNHSVSISADFGDYFMPFAGYSRTHRMPNIQEMYFSQIGDSGVHTALKPE |
| NgTdfH_WHO_W | 537 | CDPSCGLYEPVLKKYGKKRANNHSVSISADFGDYFMPFAGYSRTHRMPNIQEMYFSQIGDSGVHTALKPE |
| NgTdfH_WHO_X | 537 | CDPSCGLYEPVLKKYGKKRANNHSVSISADFGDYFMPFAGYSRTHRMPNIQEMYFSQIGDSGVHTALKPE |
| NgTdfH_WHO_Z | 537 | CDPSCGLYEPVLKKYGKKRANNHSVSISADFGDYFMPFAGYSRTHRMPNIQEMYFSQIGDSGVHTALKPE |
| NgTdfH_WHO_G | 554 | CDPSCGLYEPVLKKYGKKRANNHSVSISADFGDYFMPFAGYSRTHRMPNIQEMYFSQIGDSGVHTALKPE |
| NgTdfH_WHO_N | 554 | CDPSCGLYEPVLKKYGKKRANNHSVSISADFGDYFMPFAGYSRTHRMPNIQEMYFSQIGDSGVHTALKPE |
| NgTdfH_NG-k51.05 | 561 | CDPSCGLYEPVLKKYGKKRANNHSVSISADFGDYFMPFAGYSRTHRMPNIQEMYFSQIGDSGVHTALKPE |
| NgTdfH_WHO_V | 537 | CDPSCGLYEPVLKKYGKKRANNHSVSISADFGDYFMPFAGYSRTHRMPNIQEMYFSQIGDSGVHTALKPE |
| NgTdfH_WHO_Y | 537 | CDPSCGLYEPVLKKYGKKRANNHSVSISADFGDYFMPFAGYSRTHRMPNIQEMYFSQIGDSGVHTALKPE |
| NgTdfH_BZG33 | 554 | CDPSCGLYEPVLKKYGKKRANNHSVSISADFGDYFMPFAGYSRTHRMPNIQEMYFSQIGDSGVHTALKPE |
| NgTdfH_NCCP11945 | 537 | CDPSCGLYEPVLKKYGKKRANNHSVSISADFGDYFMPFAGYSRTHRMPNIQEMYFSQIGDSGVHTALKPE |
| NmTdfH_020-06 | 553 | CDPSCGLYEPVLKKYGKKRANNHSVSISADFGDYFMPFVGYSRTHRMPNIQEMYFSQIGDSGVHTALKPE |
| NmTdfH_alpha710 | 554 | CNQSCGIYEPVLKKYGKKRANNHSVSISADFGDYFMPFASYSRTHRMPNIQEMYFSQIGDSGVHTALKPE |
| NmTdfH_NZ-05/33 | 554 | CNQSCGIYEPVLKKYGKKRANNHSVSISADFGDYFMPFASYSRTHRMPNIQEMYFSQIGDSGVHTALKPE |
| NmTdfH_93004 | 554 | CNPSCGLYEPVLKKYGKKRANNHSVSISADFGDYFMPFASYSRTHRMPNIQEMYFSQIGDSGVHTALKPE |
| NmTdfH_ATCC13091 | 554 | CNPSCGLYEPVLKKYGKKRANNHSVSISADFGDYFMPFASYSRTHRMPNIQEMYFSQIGDSGVHTALKPE |
| NmTdfH_alpha522 | 554 | CNPSCGLYEPVLKKYGKKRANNHSVSISADFGDYFMPFASYSRTHRMPNIQEMYFSQIGDSGVHTALKPE |
| NmTdfH_NM2795 | 554 | CNPSCGLYEPVLKKYGKKRANNHSVSISADFGDYFMPFASYSRTHRMPNIQEMYFSQIGDSGVHTALKPE |
| NmTdfH_81858 | 554 | CNPSCGLYEPVLKKYGKKRANNHSVSISADFGDYFMPFASYSRTHRMPNIQEMYFSQIGDSGVHTALKPE |
| NmTdfH_ERS514534 | 554 | CNPSCGLYEPVLKKYGKKRANNHSVSISADFGDYFMPFASYSRTHRMPNIQEMYFSQIGDSGVHTALKPE |
| NmTdfH_alpha153 | 554 | CNPSCGLYEPVLKKYGKKRANNHSVSISADFGDYFMPFASYSRTHRMPNIQEMYFSQIGDSGVHTALKPE |
| NmTdfH_053442 | 539 | CNRSCGIYEPVLKKYGKKRANNHSVSISADFGDYFMPFASYSRTHRMPNIQEMYFSQIGDSGVHTALKPE |
| NmTdfH_M0579 | 553 | CNQSCGIYEPVLKKYGKKRANNHSVSISADFGDYFMPFASYSRTHRMPNIQEMYFSQIGDSGVHTALKPE |
| NmTdfH_MC58 | 553 | CNRSCGIYEPVLKKYGKKRANNHSVSISADFGDYFMPFASYSRTHRMPNIQEMYFSQIGDSGVHTALKPE |
| NmTdfH_LNP21362 | 553 | CNRSCGIYEPVLKKYGKKRANNHSVSISADFGDYFMPFASYSRTHRMPNIQEMYFSQIGDSGVHTALKPE |
| NmTdfH_IR1074 | 553 | CNRSCGIYEPVLKKYGKKRANNHSVSISADFGDYFMPFASYSRTHRMPNIQEMYFSQIGDSGVHTALKPE |
| NmTdfH_H44/76 | 553 | CNRSCGIYEPVLKKYGKKRANNHSVSISADFGDYFMPFASYSRTHRMPNIQEMYFSQIGDSGVHTALKPE |
| NmTdfH_FDAARGOS_210 | 553 | CNRSCGIYEPVLKKYGKKRANNHSVSISADFGDYFMPFASYSRTHRMPNIQEMYFSQIGDSGVHTALKPE |
| NmTdfH_FDAARGOS_215 | 554 | CNQSCGIYEPVLKKYGKKRANNHSVSISADFGDYFMPFASYSRTHRMPNIQEMYFSQIGDSGVHTALKPE |
| NmTdfH_ | 554 | CNQSCGIYEPVLKKYGKKRANNHSVSISADFGDYFMPFASYSRTHRMPNIQEMYFSQIGDSGVHTALKPE |
| NmTdfH_510612 | 537 | CNQSCGIYEPVLKKYGKKRANNHSVSISADFGDYFMPFASYSRTHRMPNIQEMYFSQIGDSGVHTALKPE |
| NmTdfH_Z2491 | 554 | CNQSCGIYEPVLKKYGKKRANNHSVSISADFGDYFMPFASYSRTHRMPNIQEMYFSQIGDSGVHTALKPE |
| NmTdfH_NCTC8249 | 554 | CNPSCGLYEPVLKKYGKKRANNHSVSISADFGDYFMPFAGYSRTHRMPNIQEMYFSQIGDSGVHTALKPE |
| NmTdfH_alpha14 | 554 | CDPSCGLYEPVLKKYGKKRANNHSVSISADFGDYFMPFASYSRTHRMPNIQEMYFSQIGDSGVHTALKPE |
| NmTdfH_2842STDY5881378 | 554 | CNRSCGIYEPVLKKYGKKRANNHSVSISADFGDYFMPFASYSRTHRMPNIQEMYFSQIGDSGVHTALKPE |
| NmTdfH_CHUV | 554 | CNPSCGLYEPVLKKYGKKRANNHSVSISADFGDYFMPFASYSRTHRMPNIQEMYFSQIGDSGVHTALKPE |
| NmTdfH_FAM18 | 554 | CNPSCGLYEPVLKKYGKKRANNHSVSISADFGDYFMPFASYSRTHRMPNIQEMYFSQIGDSGVHTALKPE |
| NmTdfH_M09293 | 554 | CNPSCGLYEPVLKKYGKKRANNHSVSISADFGDYFMPFASYSRTHRMPNIQEMYFSQIGDSGVHTALKPE |
| NmTdfH_M26503 | 554 | CNQSCGIYEPVLKKYGKKRANNHSVSISADFGDYFMPFASYSRTHRMPNIQEMYFSQIGDSGVHTALKPE |
| NmTdfH_2842STDY5881093 | 554 | CNRSCGIYEPVLKKYGKKRANNHSVSISADFGDYFMPFASYSRTHRMPNIQEMYFSQIGDSGVHTALKPE |
| NmTdfH_PMB5301 | 554 | CNRSCGIYEPVLKKYGKKRANNHSVSISADFGDYFMPFASYSRTHRMPNIQEMYFSQIGDSGVHTALKPE |

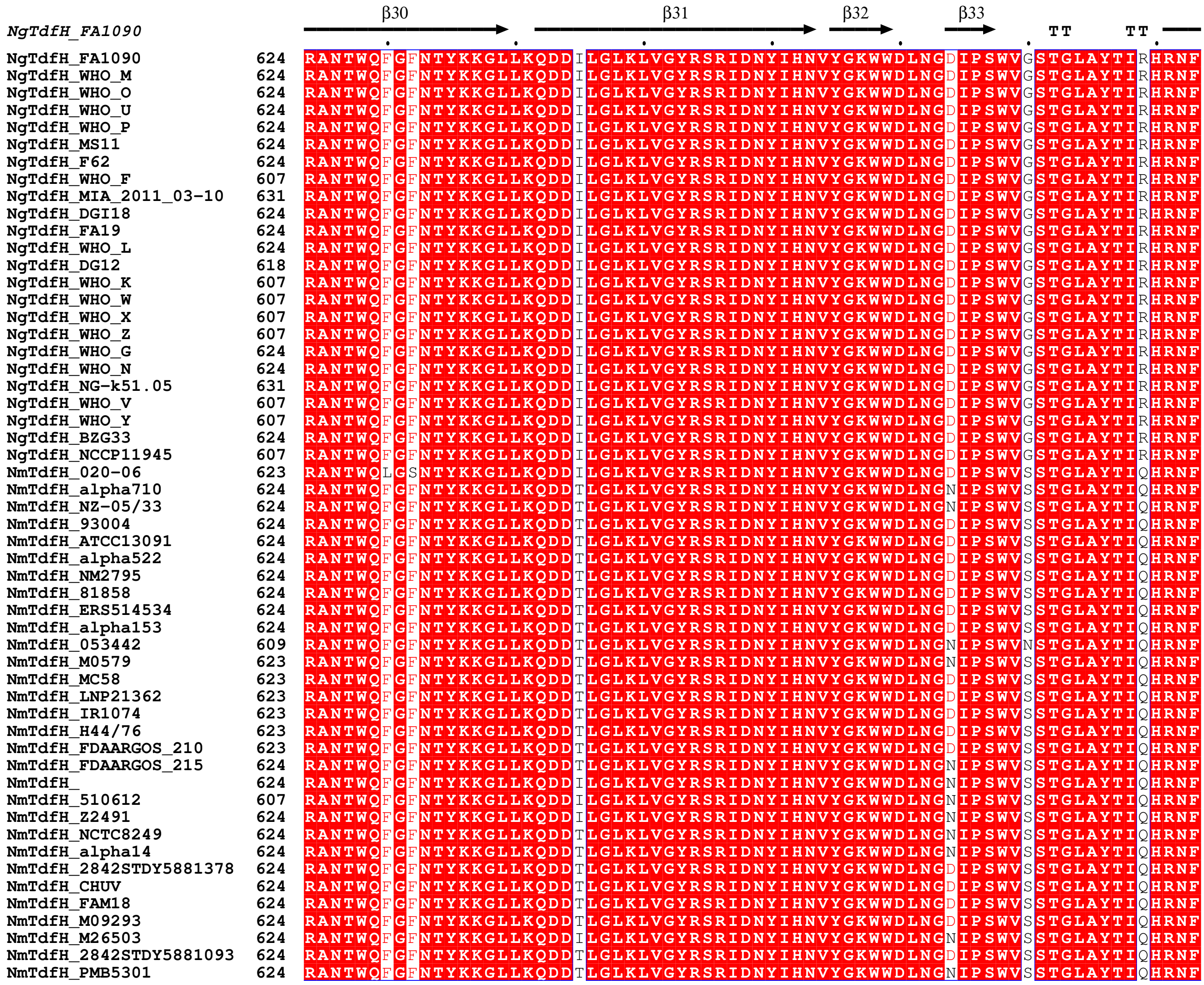
NgTdfH_FA1090
β30
β31
β32
β33
TT
TT
NgTdfH_FA1090 624 RANTWQFGFNTYKKGLLKQDDILGLKLVGYRSRIDNYIHNVYGKWWDLNGDIPSWVGSTGLAYTIRHRNF
NgTdfH_WHO_M 624 RANTWQFGFNTYKKGLLKQDDILGLKLVGYRSRIDNYIHNVYGKWWDLNGDIPSWVGSTGLAYTIRHRNF
NgTdfH_WHO_O 624 RANTWQFGFNTYKKGLLKQDDILGLKLVGYRSRIDNYIHNVYGKWWDLNGDIPSWVGSTGLAYTIRHRNF
NgTdfH_WHO_U 624 RANTWQFGFNTYKKGLLKQDDILGLKLVGYRSRIDNYIHNVYGKWWDLNGDIPSWVGSTGLAYTIRHRNF
NgTdfH_WHO_P 624 RANTWQFGFNTYKKGLLKQDDILGLKLVGYRSRIDNYIHNVYGKWWDLNGDIPSWVGSTGLAYTIRHRNF
NgTdfH_MS11 624 RANTWQFGFNTYKKGLLKQDDILGLKLVGYRSRIDNYIHNVYGKWWDLNGDIPSWVGSTGLAYTIRHRNF
NgTdfH_F62 624 RANTWQFGFNTYKKGLLKQDDILGLKLVGYRSRIDNYIHNVYGKWWDLNGDIPSWVGSTGLAYTIRHRNF
NgTdfH_WHO_F 607 RANTWQFGFNTYKKGLLKQDDILGLKLVGYRSRIDNYIHNVYGKWWDLNGDIPSWVGSTGLAYTIRHRNF
NgTdfH_MIA_2011_03-10 631 RANTWQFGFNTYKKGLLKQDDILGLKLVGYRSRIDNYIHNVYGKWWDLNGDIPSWVGSTGLAYTIRHRNF
NgTdfH_DGI18 624 RANTWQFGFNTYKKGLLKQDDILGLKLVGYRSRIDNYIHNVYGKWWDLNGDIPSWVGSTGLAYTIRHRNF
NgTdfH_FA19 624 RANTWQFGFNTYKKGLLKQDDILGLKLVGYRSRIDNYIHNVYGKWWDLNGDIPSWVGSTGLAYTIRHRNF
NgTdfH_WHO_L 624 RANTWQFGFNTYKKGLLKQDDILGLKLVGYRSRIDNYIHNVYGKWWDLNGDIPSWVGSTGLAYTIRHRNF
NgTdfH_DG12 618 RANTWQFGFNTYKKGLLKQDDILGLKLVGYRSRIDNYIHNVYGKWWDLNGDIPSWVGSTGLAYTIRHRNF
NgTdfH_WHO_K 607 RANTWQFGFNTYKKGLLKQDDILGLKLVGYRSRIDNYIHNVYGKWWDLNGDIPSWVGSTGLAYTIRHRNF
NgTdfH_WHO_W 607 RANTWQFGFNTYKKGLLKQDDILGLKLVGYRSRIDNYIHNVYGKWWDLNGDIPSWVGSTGLAYTIRHRNF
NgTdfH_WHO_X 607 RANTWQFGFNTYKKGLLKQDDILGLKLVGYRSRIDNYIHNVYGKWWDLNGDIPSWVGSTGLAYTIRHRNF
NgTdfH_WHO_Z 607 RANTWQFGFNTYKKGLLKQDDILGLKLVGYRSRIDNYIHNVYGKWWDLNGDIPSWVGSTGLAYTIRHRNF
NgTdfH_WHO_G 624 RANTWQFGFNTYKKGLLKQDDILGLKLVGYRSRIDNYIHNVYGKWWDLNGDIPSWVGSTGLAYTIRHRNF
NgTdfH_WHO_N 624 RANTWQFGFNTYKKGLLKQDDILGLKLVGYRSRIDNYIHNVYGKWWDLNGDIPSWVGSTGLAYTIRHRNF
NgTdfH_NG-k51.05 631 RANTWQFGFNTYKKGLLKQDDILGLKLVGYRSRIDNYIHNVYGKWWDLNGDIPSWVGSTGLAYTIRHRNF
NgTdfH_WHO_V 607 RANTWQFGFNTYKKGLLKQDDILGLKLVGYRSRIDNYIHNVYGKWWDLNGDIPSWVGSTGLAYTIRHRNF
NgTdfH_WHO_Y 607 RANTWQFGFNTYKKGLLKQDDILGLKLVGYRSRIDNYIHNVYGKWWDLNGDIPSWVGSTGLAYTIRHRNF
NgTdfH_BZG33 624 RANTWQFGFNTYKKGLLKQDDILGLKLVGYRSRIDNYIHNVYGKWWDLNGDIPSWVGSTGLAYTIRHRNF
NgTdfH_NCCP11945 607 RANTWQFGFNTYKKGLLKQDDILGLKLVGYRSRIDNYIHNVYGKWWDLNGDIPSWVGSTGLAYTIRHRNF
NmTdfH_020-06 623 RANTWQLGSNTYKKGLLKQDDILGLKLVGYRSRIDNYIHNVYGKWWDLNGDIPSWVSSTGLAYTIQHRNF
NmTdfH_alpha710 624 RANTWQFGFNTYKKGLLKQDDTLGLKLVGYRSRIDNYIHNVYGKWWDLNGNIPSWVSSTGLAYTIQHRNF
NmTdfH_NZ-05/33 624 RANTWQFGFNTYKKGLLKQDDTLGLKLVGYRSRIDNYIHNVYGKWWDLNGNIPSWVSSTGLAYTIQHRNF
NmTdfH_93004 624 RANTWQFGFNTYKKGLLKQDDTLGLKLVGYRSRIDNYIHNVYGKWWDLNGDIPSWVSSTGLAYTIQHRNF
NmTdfH_ATCC13091 624 RANTWQFGFNTYKKGLLKQDDTLGLKLVGYRSRIDNYIHNVYGKWWDLNGDIPSWVSSTGLAYTIQHRNF
NmTdfH_alpha522 624 RANTWQFGFNTYKKGLLKQDDTLGLKLVGYRSRIDNYIHNVYGKWWDLNGDIPSWVSSTGLAYTIQHRNF
NmTdfH_NM2795 624 RANTWQFGFNTYKKGLLKQDDTLGLKLVGYRSRIDNYIHNVYGKWWDLNGDIPSWVSSTGLAYTIQHRNF
NmTdfH_81858 624 RANTWQFGFNTYKKGLLKQDDTLGLKLVGYRSRIDNYIHNVYGKWWDLNGDIPSWVSSTGLAYTIQHRNF
NmTdfH_ERS514534 624 RANTWQFGFNTYKKGLLKQDDTLGLKLVGYRSRIDNYIHNVYGKWWDLNGDIPSWVSSTGLAYTIQHRNF
NmTdfH_alpha153 624 RANTWQFGFNTYKKGLLKQDDTLGLKLVGYRSRIDNYIHNVYGKWWDLNGDIPSWVSSTGLAYTIQHRNF
NmTdfH_053442 609 RANTWQFGFNTYKKGLLKQDDTLGLKLVGYRSRIDNYIHNVYGKWWDLNGNIPSWVNSTGLAYTIQHRNF
NmTdfH_M0579 623 RANTWQFGFNTYKKGLLKQDDTLGLKLVGYRSRIDNYIHNVYGKWWDLNGNIPSWVSSTGLAYTIQHRNF
NmTdfH_MC58 623 RANTWQFGFNTYKKGLLKQDDTLGLKLVGYRSRIDNYIHNVYGKWWDLNGDIPSWVSSTGLAYTIQHRNF
NmTdfH_LNP21362 623 RANTWQFGFNTYKKGLLKQDDTLGLKLVGYRSRIDNYIHNVYGKWWDLNGDIPSWVSSTGLAYTIQHRNF
NmTdfH_IR1074 623 RANTWQFGFNTYKKGLLKQDDTLGLKLVGYRSRIDNYIHNVYGKWWDLNGDIPSWVSSTGLAYTIQHRNF
NmTdfH_H44/76 623 RANTWQFGFNTYKKGLLKQDDTLGLKLVGYRSRIDNYIHNVYGKWWDLNGDIPSWVSSTGLAYTIQHRNF
NmTdfH_FDAARGOS_210 623 RANTWQFGFNTYKKGLLKQDDTLGLKLVGYRSRIDNYIHNVYGKWWDLNGDIPSWVSSTGLAYTIQHRNF
NmTdfH_FDAARGOS_215 624 RANTWQFGFNTYKKGLLKQDDTLGLKLVGYRSRIDNYIHNVYGKWWDLNGNIPSWVSSTGLAYTIQHRNF
NmTdfH_ 624 RANTWQFGFNTYKKGLLKQDDILGLKLVGYRSRIDNYIHNVYGKWWDLNGNIPSWVSSTGLAYTIQHRNF
NmTdfH_510612 607 RANTWQFGFNTYKKGLLKQDDILGLKLVGYRSRIDNYIHNVYGKWWDLNGNIPSWVSSTGLAYTIQHRNF
NmTdfH_Z2491 624 RANTWQFGFNTYKKGLLKQDDILGLKLVGYRSRIDNYIHNVYGKWWDLNGNIPSWVSSTGLAYTIQHRNF
NmTdfH_NCTC8249 624 RANTWQFGFNTYKKGLLKQDDTLGLKLVGYRSRIDNYIHNVYGKWWDLNGNIPSWVSSTGLAYTIQHRNF
NmTdfH_alpha14 624 RANTWQFGFNTYKKGLLKQDDTLGLKLVGYRSRIDNYIHNVYGKWWDLNGNIPSWVSSTGLAYTIQHRNF
NmTdfH_2842STDY5881378 624 RANTWQFGFNTYKKGLLKQDDTLGLKLVGYRSRIDNYIHNVYGKWWDLNGDIPSWVSSTGLAYTIQHRNF
NmTdfH_CHUV 624 RANTWQFGFNTYKKGLLKQDDTLGLKLVGYRSRIDNYIHNVYGKWWDLNGDIPSWVSSTGLAYTIQHRNF
NmTdfH_FAM18 624 RANTWQFGFNTYKKGLLKQDDTLGLKLVGYRSRIDNYIHNVYGKWWDLNGDIPSWVSSTGLAYTIQHRNF
NmTdfH_M09293 624 RANTWQFGFNTYKKGLLKQDDTLGLKLVGYRSRIDNYIHNVYGKWWDLNGDIPSWVSSTGLAYTIQHRNF
NmTdfH_M26503 624 RANTWQFGFNTYKKGLLKQDDILGLKLVGYRSRIDNYIHNVYGKWWDLNGNIPSWVSSTGLAYTIQHRNF
NmTdfH_2842STDY5881093 624 RANTWQFGFNTYKKGLLKQDDTLGLKLVGYRSRIDNYIHNVYGKWWDLNGDIPSWVSSTGLAYTIQHRNF
NmTdfH_PMB5301 624 RANTWQFGFNTYKKGLLKQDDTLGLKLVGYRSRIDNYIHNVYGKWWDLNGNIPSWVSSTGLAYTIQHRNF

*NgTdfH_FA1090* β34 β35 β36

| | | |
|---|---|---|
| NgTdfH_FA1090 | 694 | KDKVHKHGFELELNYDYGRFFTNLSYAYQKSTQPTNFSDASESPNNASKEDQLKQGYGLSRVSALPRDYG |
| NgTdfH_WHO_M | 694 | KDKVHKHGFELELNYDYGRFFTNLSYAYQKSTQPTNFSDASESPNNASKEDQLKQGYGLSRVSALPRDYG |
| NgTdfH_WHO_O | 694 | KDKVHKHGFELELNYDYGRFFTNLSYAYQKSTQPTNFSDASESPNNASKEDQLKQGYGLSRVSALPRDYG |
| NgTdfH_WHO_U | 694 | KDKVHKHGFELELNYDYGRFFTNLSYAYQKSTQPTNFSDASESPNNASKEDQLKQGYGLSRVSALPRDYG |
| NgTdfH_WHO_P | 694 | KDKVHKHGFELELNYDYGRFFTNLSYAYQKSTQPTNFSDASESPNNASKEDQLKQGYGLSRVSALPRDYG |
| NgTdfH_MS11 | 694 | KDKVHKHGFELELNYDYGRFFTNLSYAYQKSTQPTNFSDASESPNNASKEDQLKQGYGLSRVSALPRDYG |
| NgTdfH_F62 | 694 | KDKVHKHGFELELNYDYGRFFTNLSYAYQKSTQPTNFSDASESPNNASKEDQLKQGYGLSRVSALPRDYG |
| NgTdfH_WHO_F | 677 | KDKVHKHGFELELNYDYGRFFTNLSYAYQKSTQPTNFSDASESPNNASKEDQLKQGYGLSRVSALPRDYG |
| NgTdfH_MIA_2011_03-10 | 701 | KDKVHKHGFELELNYDYGRFFTNLSYAYQKSTQPTNFSDASESPNNASKEDQLKQGYGLSRVSALPRDYG |
| NgTdfH_DGI18 | 694 | KDKVHKHGFELELNYDYGRFFTNLSYAYQKSTQPTNFSDASESPNNASKEDQLKQGYGLSRVSALPRDYG |
| NgTdfH_FA19 | 694 | KDKVHKHGFELELNYDYGRFFTNLSYAYQKSTQPTNFSDASESPNNASKEDQLKQGYGLSRVSALPRDYG |
| NgTdfH_WHO_L | 694 | KDKVHKHGFELELNYDYGRFFTNLSYAYQKSTQPTNFSDASESPNNASKEDQLKQGYGLSRVSALPRDYG |
| NgTdfH_DG12 | 688 | KDKVHKHGFELELNYDYGRFFTNLSYAYQKSTQPTNFSDASESPNNASKEDQLKQGYGLSRVSALPRDYG |
| NgTdfH_WHO_K | 677 | KDKVHKHGFELELNYDYGRFFTNLSYAYQKSTQPTNFSDASESPNNASKEDQLKQGYGLSRVSALPRDYG |
| NgTdfH_WHO_W | 677 | KDKVHKHGFELELNYDYGRFFTNLSYAYQKSTQPTNFSDASESPNNASKEDQLKQGYGLSRVSALPRDYG |
| NgTdfH_WHO_X | 677 | KDKVHKHGFELELNYDYGRFFTNLSYAYQKSTQPTNFSDASESPNNASKEDQLKQGYGLSRVSALPRDYG |
| NgTdfH_WHO_Z | 677 | KDKVHKHGFELELNYDYGRFFTNLSYAYQKSTQPTNFSDASESPNNASKEDQLKQGYGLSRVSALPRDYG |
| NgTdfH_WHO_G | 694 | KDKVHKHGFELELNYDYGRFFTNLSYAYQKSTQPTNFSDASESPNNASKEDQLKQGYGLSRVSALPRDYG |
| NgTdfH_WHO_N | 694 | KDKVHKHGFELELNYDYGRFFTNLSYAYQKSTQPTNFSDASESPNNASKEDQLKQGYGLSRVSALPRDYG |
| NgTdfH_NG-k51.05 | 701 | KDKVHKHGFELELNYDYGRFFTNLSYAYQKSTQPTNFSDASESPNNASKEDQLKQGYGLSRVSALPRDYG |
| NgTdfH_WHO_V | 677 | KDKVHKHGFELELNYDYGRFFTNLSYAYQKSTQPTNFSDASESPNNASKEDQLKQGYGLSRVSALPRDYG |
| NgTdfH_WHO_Y | 677 | KDKVHKHGFELELNYDYGRFFTNLSYAYQKSTQPTNFSDASESPNNASKEDQLKQGYGLSRVSALPRDYG |
| NgTdfH_BZG33 | 694 | KDKVHKHGFELELNYDYGRFFTNLSYAYQKSTQPTNFSDASESPNNASKEDQLKQGYGLSRVSALPRDYG |
| NgTdfH_NCCP11945 | 677 | KDKVHKHGFELELNYDYGRFFTNLSYAYQKSTQPTNFSDASESPNNASKEDQLKQGYGLSRVSALPRDYG |
| NmTdfH_020-06 | 693 | KDKVHKHGFELELNYDYGRFFTNLSYAYQKSTQPTNFSDASESPNNASKEDQLKQGYGLSRVSALPRDYG |
| NmTdfH_alpha710 | 694 | KDKVHKHGFELELNYDYGRFFTNLSYAYQKSTQPTNFSDASESPNNASKEDQLKQGYGLSRVSALPRDYG |
| NmTdfH_NZ-05/33 | 694 | KDKVHKHGFELELNYDYGRFFTNLSYAYQKSTQPTNFSDASESPNNASKEDQLKQGYGLSRVSALPRDYG |
| NmTdfH_93004 | 694 | KDKVHKHGFELELNYDYGRFFTNLSYAYQKSTQPTNFSDASESPNNASKEDQLKQGYGLSRVSALPRDYG |
| NmTdfH_ATCC13091 | 694 | KDKVHKHGFELELNYDYGRFFTNLSYAYQKSTQPTNFSDASESPNNASKEDQLKQGYGLSRVSALPRDYG |
| NmTdfH_alpha522 | 694 | KDKVHKHGFELELNYDYGRFFTNLSYAYQKSTQPTNFSDASESPNNASKEDQLKQGYGLSRVSALPRDYG |
| NmTdfH_NM2795 | 694 | KDKVHKHGFELELNYDYGRFFTNLSYAYQKSTQPTNFSDASESPNNASKEDQLKQGYGLSRVSALPRDYG |
| NmTdfH_81858 | 694 | KDKVHKHGFELELNYDYGRFFTNLSYAYQKSTQPTNFSDASESPNNASKEDQLKQGYGLSRVSALPRDYG |
| NmTdfH_ERS514534 | 694 | KDKVHKHGFELELNYDYGRFFTNLSYAYQKSTQPTNFSDASESPNNASKEDQLKQGYGLSRVSALPRDYG |
| NmTdfH_alpha153 | 694 | KDKVHKHGFELELNYDYGRFFTNLSYAYQKSTQPTNFSDASESPNNASKEDQLKQGYGLSRVSALPRDYG |
| NmTdfH_053442 | 679 | KDKVHKHGFELELNYDYGRFFTNLSYAYQKSTQPTNFSDASESPNNASKEDQLKQGYGLSRVSALPRDYG |
| NmTdfH_M0579 | 693 | KDKVHKHGFELELNYDYGRFFTNLSYAYQKSTQPTNFSDASESPNNASKEDQLKQGYGLSRVSALPRDYG |
| NmTdfH_MC58 | 693 | KDKVHKHGFELELNYDYGRFFTNLSYAYQKSTQPTNFSDASESPNNASKEDQLKQGYGLSRVSALPRDYG |
| NmTdfH_LNP21362 | 693 | KDKVHKHGFELELNYDYGRFFTNLSYAYQKSTQPTNFSDASESPNNASKEDQLKQGYGLSRVSALPRDYG |
| NmTdfH_IR1074 | 693 | KDKVHKHGFELELNYDYGRFFTNLSYAYQKSTQPTNFSDASESPNNASKEDQLKQGYGLSRVSALPRDYG |
| NmTdfH_H44/76 | 693 | KDKVHKHGFELELNYDYGRFFTNLSYAYQKSTQPTNFSDASESPNNASKEDQLKQGYGLSRVSALPRDYG |
| NmTdfH_FDAARGOS_210 | 693 | KDKVHKHGFELELNYDYGRFFTNLSYAYQKSTQPTNFSDASESPNNASKEDQLKQGYGLSRVSALPRDYG |
| NmTdfH_FDAARGOS_215 | 694 | KDKVHKHGFELELNYDYGRFFTNLSYAYQKSTQPTNFSDASESPNNASKEDQLKQGYGLSRVSALPRDYG |
| NmTdfH_ | 694 | KDKVHKHGFELELNYDYGRFFTNLSYAYQKSTQPTNFSDASESPNNASKEDQLKQGYGLSRVSALPRDYG |
| NmTdfH_510612 | 677 | KDKVHKHGFELELNYDYGRFFTNLSYAYQKSTQPTNFSDASESPNNASKEDQLKQGYGLSRVSALPRDYG |
| NmTdfH_Z2491 | 694 | KDKVHKHGFELELNYDYGRFFTNLSYAYQKSTQPTNFSDASESPNNASKEDQLKQGYGLSRVSALPRDYG |
| NmTdfH_NCTC8249 | 694 | KDKVHKHGFELELNYDYGRFFTNLSYAYQKSTQPTNFSDASESPNNASKEDQLKQGYGLSRVSALPRDYG |
| NmTdfH_alpha14 | 694 | KDKVHKHGFELELNYDYGRFFTNLSYAYQKSTQPTNFSDASESPNNASKEDQLKQGYGLSRVSALPRDYG |
| NmTdfH_2842STDY5881378 | 694 | KDKVHKHGFELELNYDYGRFFTNLSYAYQKSTQPTNFSDASESPNNASKEDQLKQGYGLSRVSALPRDYG |
| NmTdfH_CHUV | 694 | KDKVHKHGFELELNYDYGRFFTNLSYAYQKSTQPTNFSDASESPNNASKEDQLKQGYGLSRVSALPRDYG |
| NmTdfH_FAM18 | 694 | KDKVHKHGFELELNYDYGRFFTNLSYAYQKSTQPTNFSDASESPNNASKEDQLKQGYGLSRVSALPRDYG |
| NmTdfH_M09293 | 694 | KDKVHKHGFELELNYDYGRFFTNLSYAYQKSTQPTNFSDASESPNNASKEDQLKQGYGLSRVSALPRDYG |
| NmTdfH_M26503 | 694 | KDKVHKHGFELELNYDYGRFFTNLSYAYQKSTQPTNFSDASESPNNASKEDQLKQGYGLSRVSALPRDYG |
| NmTdfH_2842STDY5881093 | 694 | KDKVHKHGFELELNYDYGRFFTNLSYAYQKSTQPTNFSDASESPNNASKEDQLKQGYGLSRVSALPRDYG |
| NmTdfH_PMB5301 | 694 | KDKVHKHGFELELNYDYGRFFTNLSYAYQKSTQPTNFSDASESPNNASKEDQLKQGYGLSRVSALPRDYG |

```
                                β37          TTT     β38                  β39         α6              β40                β41
NgTdfH_FA1090

NgTdfH_FA1090              764  RLEVGTRWLGNKLTLGGAMRYFGKSIRATAEERYIDGTNGGNTSNVRQLGKRSIKQTETLARQPLIFDFY
NgTdfH_WHO_M               764  RLEVGTRWLGNKLTLGGAMRYFGKSIRATAEERYIDGTNGGNTSNVRQLGKRSIKQTETLARQPLIFDFY
NgTdfH_WHO_O               764  RLEVGTRWLGNKLTLGGAMRYFGKSIRATAEERYIDGTNGGNTSNVRQLGKRSIKQTETLARQPLIFDFY
NgTdfH_WHO_U               764  RLEVGTRWLGNKLTLGGAMRYFGKSIRATAEERYIDGTNGGNTSNVRQLGKRSIKQTETLARQPLIFDFY
NgTdfH_WHO_P               764  RLEVGTRWLGNKLTLGGAMRYFGKSIRATAEERYIDGTNGGNTSNVRQLGKRSIKQTETLARQPLIFDFY
NgTdfH_MS11                764  RLEVGTRWLGNKLTLGGAMRYFGKSIRATAEERYIDGTNGGNTSNVRQLGKRSIKQTETLARQPLIFDFY
NgTdfH_F62                 764  RLEVGTRWLGNKLTLGGAMRYFGKSIRATAEERYIDGTNGGNTSNVRQLGKRSIKQTETLARQPLIFDFY
NgTdfH_WHO_F               747  RLEVGTRWLGNKLTLGGAMRYFGKSIRATAEERYIDGTNGGNTSNVRQLGKRSIKQTETLARQPLIFDFY
NgTdfH_MIA_2011_03-10      771  RLEVGTRWLGNKLTLGGAMRYFGKSIRATAEERYIDGTTGANTSDVRQLGKRSIKQTETLARQPLIFDFY
NgTdfH_DGI18               764  RLEVGTRWLGNKLTLGGAMRYFGKSIRATAEERYIDGTNGGNTRDVRQLGKRSIKQTETLARQPLIFDFY
NgTdfH_FA19                764  RLEVGTRWLGNKLTLGGAMRYFGKSIRATAEERYIDGTTGGNTSNVRQLGKRSIKQTETLARQPLIFDFY
NgTdfH_WHO_L               764  RLEVGTRWLGNKLTLGGAMRYFGKSIRATAEERYIDGTTGGNTSNVRQLGKRSIKQTETLARQPLIFDFY
NgTdfH_DG12                758  RLEVGTRWLGNKLTLGGAMRYFGKSIRATAEERYIDGTNGGNTSNVRQLGKRSIKQTETLARQPLIFDFY
NgTdfH_WHO_K               747  RLEVGTRWLGNKLTLGGAMRYFGKSIRATAEERYIDGTNGGNTSNVRQLGKRSIKQTETLARQPLIFDFY
NgTdfH_WHO_W               747  RLEVGTRWLGNKLTLGGAMRYFGKSIRATAEERYIDGTNGGNTSNVRQLGKRSIKQTETLARQPLIFDFY
NgTdfH_WHO_X               747  RLEVGTRWLGNKLTLGGAMRYFGKSIRATAEERYIDGTNGGNTSNVRQLGKRSIKQTETLARQPLIFDFY
NgTdfH_WHO_Z               747  RLEVGTRWLGNKLTLGGAMRYFGKSIRATAEERYIDGTNGGNTSNVRQLGKRSIKQTETLARQPLIFDFY
NgTdfH_WHO_G               764  RLEVGTRWLGNKLTLGGAMRYFGKSIRATAEERYIDGTNGGNTSNVRQLGKRSIKQTETLARQPLIFDFY
NgTdfH_WHO_N               764  RLEVGTRWLGNKLTLGGAMRYFGKSIRATAEERYIDGTNGGNTSNVRQLGKRSIKQTETLARQPLIFDFY
NgTdfH_NG-k51.05           771  RLEVGTRWLGNKLTLGGAMRYFGKSIRATAEERYIDGTNGGNTSNVRQLGKRSIKQTETLARQPLIFDFY
NgTdfH_WHO_V               747  RLEVGTRWLGNKLTLGGAMRYFGKSIRATAEERYIDGTNGGNTSNVRQLGKRSIKQTETLARQPLIFDFY
NgTdfH_WHO_Y               747  RLEVGTRWLGNKLTLGGAMRYFGKSIRATAEERYIDGTNGGNTSNVRQLGKRSIKQTETLARQPLIFDFY
NgTdfH_BZG33               764  RLEVGTRWLGNKLTLGGAMRYFGKSIRATAEERYIDGTNGGNTSNVRQLGKRSIKQTETLARQPLIFDFY
NgTdfH_NCCP11945           747  RLEVGTRWLGNKLTLGGAMRYFGKSIRATAEERYIDGTNGGNTSNVRQLGKRSIKQTETLARQPLIFDFY
NmTdfH_020-06              763  RLEVGTRWLGNKLTLGGAMRYFGKSIRATAEERYIDGTNGGNTSNVRQLGKRSIKQTETLARQPLIFDFY
NmTdfH_alpha710            764  RLEVGTRWLGNKLTLGGAMRYFGKSIRATAEERYIDGTNGGNTSNVRQLGKRSIKQTETLARQPLIFDFY
NmTdfH_NZ-05/33            764  RLEVGTRWLGNKLTLGGAMRYFGKSIRATAEERYIDGTNGGNTSNVRQLGKRSIKQTETLARQPLIFDFY
NmTdfH_93004               764  RLEVGTRWLGNKLTLGGAMRYFGKSIRATAEERYIDGTNGGNTSNVRQLGKRSIKQTETLARQPLIFDFY
NmTdfH_ATCC13091           764  RLEVGTRWLGNKLTLGGAMRYFGKSIRATAEERYIDGTNGGNTSNVRQLGKRSIKQTETLARQPLIFDFY
NmTdfH_alpha522            764  RLEVGTRWLGNKLTLGGAMRYFGKSIRATAEERYIDGTNGGNTRDVRQLGKRSIKQTETLARQPLIFDFY
NmTdfH_NM2795              764  RLEVGTRWLGNKLTLGGAMRYFGKSIRATAEERYIDGTNGGNTRDVRQLGKRSIKQTETLARQPLIFDFY
NmTdfH_81858               764  RLEVGTRWLGNKLTLGGAMRYFGKSIRATAEERYIDGTNGGNTSNVRQLGKRSIKQTETLARQPLIFDFY
NmTdfH_ERS514534           764  RLEVGTRWLGNKLTLGGAMRYFGKSIRATAEERYIDGTNGGNTSNVRQLGKRSIKQTETLARQPLIFDFY
NmTdfH_alpha153            764  RLEVGTRWLGNKLTLGGAMRYFGKSIRATAEERYIDGTNGGNTSNVRQLGKRSIKQTETLARQPLIFDFY
NmTdfH_053442              749  RLEVGTRWLGNKLTLGGAMRYFGKSIRATAEERYIDGTNGGNTSNVRQLGKRSIKQTETLARQPLIFDFY
NmTdfH_M0579               763  RLEVGTRWLGNKLTLGGAMRYFGKSIRATAEERYIDGTNGGNTSNVRQLGKRSIKQTETLARQPLIFDFY
NmTdfH_MC58                763  RLEVGTRWLGNKLTLGGAMRYFGKSIRATAEERYIDGTNGGNTSNFRQLGKRSIKQTETLARQPLIFDFY
NmTdfH_LNP21362            763  RLEVGTRWLGNKLTLGGAMRYFGKSIRATAEERYIDGTNGGNTSNFRQLGKRSIKQTETLARQPLIFDFY
NmTdfH_IR1074              763  RLEVGTRWLGNKLTLGGAMRYFGKSIRATAEERYIDGTNGGNTSNFRQLGKRSIKQTETLARQPLIFDFY
NmTdfH_H44/76              763  RLEVGTRWLGNKLTLGGAMRYFGKSIRATAEERYIDGTNGGNTSNFRQLGKRSIKQTETLARQPLIFDFY
NmTdfH_FDAARGOS_210        763  RLEVGTRWLGNKLTLGGAMRYFGKSIRATAEERYIDGTNGGNTSNFRQLGKRSIKQTETLARQPLIFDFY
NmTdfH_FDAARGOS_215        764  RLEVGTRWLGNKLTLGGAMRYFGKSIRATAEERYIDGTNGGNTSNVRQLGKRSIKQTETLARQPLIFDFY
NmTdfH_                    764  RLEVGTRWLGNKLTLGGAMRYFGKSIRATAEERYIDGTNGGNTSNFRQLGKRSIKQTETLARQPLIFDFY
NmTdfH_510612              747  RLEVGTRWLGNKLTLGGAMRYFGKSIRATAEERYIDGTNGGNTSNFRQLGKRSIKQTETLARQPLIFDFY
NmTdfH_Z2491               764  RLEVGTRWLGNKLTLGGAMRYFGKSIRATAEERYIDGTNGGNTSNFRQLGKRSIKQTETLARQPLIFDFY
NmTdfH_NCTC8249            764  RLEVGTRWLGNKLTLGGAMRYFGKSIRATAEERYIDGTNGGNTSNFRQLGKRSIKQTETLARQPLIFDFY
NmTdfH_alpha14             764  RLEVGTRWLGNKLTLGGAMRYFGKSIRATAEERYIDGTNGGNTSNVRQLGKRSIKQTETLARQPLIFDFY
NmTdfH_2842STDY5881378     764  RLEVGTRWLGNKLTLGGAMRYFGKSIRATAEERYIDGTNGGNTSNFRQLGKRSIKQTETLARQPLIFDFY
NmTdfH_CHUV                764  RLEVGTRWLGNKLTLGGAMRYFGKSIRATAEERYIDGTNGGNTSNFRQLGKRSIKQTETLARQPLIFDFY
NmTdfH_FAM18               764  RLEVGTRWLGNKLTLGGAMRYFGKSIRATAEERYIDGTNGGNTSNFRQLGKRSIKQTETLARQPLIFDFY
NmTdfH_M09293              764  RLEVGTRWLGNKLTLGGAMRYFGKSIRATAEERYIDGTNGGNTSNFRQLGKRSIKQTETLARQPLIFDFY
NmTdfH_M26503              764  RLEVGTRWLGNKLTLGGAMRYFGKSIRATAEERYIDGTNGGNTSNFRQLGKRSIKQTETLARQPLIFDFY
NmTdfH_2842STDY5881093     764  RLEVGTRWLGNKLTLGGAMRYFGKSIRATAEERYIDGTNGGNTSNFRQLGKRSIKQTETLARQPLIFDFY
NmTdfH_PMB5301             764  RLEVGTRWLGNKLTLGGAMRYFGKSIRATAEERYIDGTNGGNTSNVRQLGKRSIKQTETLARQPLIFDFY
```

```
                                      η7    β42                                                      TT   TT
NgTdfH_FA1090
NgTdfH_FA1090               834  AAYEPKKNLIFRAEVKNLFDRRYIDPLDAGNDAATQRYYSSFDPKDKDEDVTCNADKTLCNGKYGGTSKS
NgTdfH_WHO_M                834  AAYEPKKNLIFRAEVKNLFDRRYIDPLDAGNDAATQRYYSSFDPKDKDEDVTCNADKTLCNGKYGGTSKS
NgTdfH_WHO_O                834  AAYEPKKNLIFRAEVKNLFDRRYIDPLDAGNDAATQRYYSSFDPKDKDEDVTCNADKTLCNGKYGGTSKS
NgTdfH_WHO_U                834  AAYEPKKNLIFRAEVKNLFDRRYIDPLDAGNDAATQRYYSSFDPKDKDEDVTCNADKTLCNGKYGGTSKS
NgTdfH_WHO_P                834  AAYEPKKNLIFRAEVKNLFDRRYIDPLDAGNDAATQRYYSSFDPKDKDEDVTCNADKTLCNGKYGGTSKS
NgTdfH_MS11                 834  AAYEPKKNLIFRAEVKNLFDRRYIDPLDAGNDAATQRYYSSFDPKDKDEDVTCNADKTLCNGKYGGTSKS
NgTdfH_F62                  834  AAYEPKKNLIFRAEVKNLFDRRYIDPLDAGNDAATQRYYSSFDPKDKDEDVTCNADKTLCNGKYGGTSKS
NgTdfH_WHO_F                817  AAYEPKKNLIFRAEVKNLFDRRYIDPLDAGNDAATQRYYSSFDPKDKDEDVTCNADKTLCNGKYGGTSKS
NgTdfH_MIA_2011_03-10       841  AAYEPKKNLIFRAEVKNLFDRRYIDPLDAGNDAATQRYYSSFDPKDKDEEVTCNDDNTLCNGKYGGTSKS
NgTdfH_DGI18                834  AAYEPKKNLIFRAEVKNLFDRRYIDPLDAGNDAATQRYYSSFDPKDKDEDVTCNADKTLCNGKYGGTSKS
NgTdfH_FA19                 834  AAYEPKKNLIFRAEVKNLFDRRYIDPLDAGNDAATQRYYSSFDPKDKDEEVTCNDDNTLCNGKYGGTSKS
NgTdfH_WHO_L                834  AAYEPKKNLIFRAEVKNLFDRRYIDPLDAGNDAATQRYYSSFDPKDKDEEVTCNDDNTLCNGKYGGTSKS
NgTdfH_DG12                 828  AAYEPKKNLIFRAEVKNLFDRRYIDPLDAGNDAATQRYYSSFDPKDKDEDVTCNADKTLCNGKYGGTSKS
NgTdfH_WHO_K                817  AAYEPKKNLIFRAEVKNLFDRRYIDPLDAGNDAATQRYYSSFDPKDKDEDVTCNADKTLCNGKYGGTSKS
NgTdfH_WHO_W                817  AAYEPKKNLIFRAEVKNLFDRRYIDPLDAGNDAATQRYYSSFDPKDKDEDVTCNADKTLCNGKYGGTSKS
NgTdfH_WHO_X                817  AAYEPKKNLIFRAEVKNLFDRRYIDPLDAGNDAATQRYYSSFDPKDKDEDVTCNADKTLCNGKYGGTSKS
NgTdfH_WHO_Z                817  AAYEPKKNLIFRAEVKNLFDRRYIDPLDAGNDAATQRYYSSFDPKDKDEDVTCNADKTLCNGKYGGTSKS
NgTdfH_WHO_G                834  AAYEPKKNLIFRAEVKNLFDRRYIDPLDAGNDAATQRYYSSFDPKDKDEDVTCNADKTLCNGKYGGTSKS
NgTdfH_WHO_N                834  AAYEPKKNLIFRAEVKNLFDRRYIDPLDAGNDAATQRYYSSFDPKDKDEDVTCNADKTLCNGKYGGTSKS
NgTdfH_NG-k51.05            841  AAYEPKKNLIFRAEVKNLFDRRYIDPLDAGNDAATQRYYSSFDPKDKDEDVTCNADKTLCNGKYGGTSKS
NgTdfH_WHO_V                817  AAYEPKKNLIFRAEVKNLFDRRYIDPLDAGNDAATQRYYSSFDPKDKDEDVTCNADKTLCNGKYGGTSKS
NgTdfH_WHO_Y                817  AAYEPKKNLIFRAEVKNLFDRRYIDPLDAGNDAATQRYYSSFDPKDKDEDVTCNADKTLCNGKYGGTSKS
NgTdfH_BZG33                834  AAYEPKKNLIFRAEVKNLFDRRYIDPLDAGNDAATQRYYSSFDPKDKDEDVTCNADKTLCNGKYGGTSKS
NgTdfH_NCCP11945            817  AAYEPKKNLIFRAEVKNLFDRRYIDPLDAGNDAATQRYYSSFDPKDKDEDVTCNADKTLCNGKYGGTSKS
NmTdfH_020-06               833  AAYEPKKNLIFRAEVKNLFDRRYIDPLDAGNDAATQRYYSSFDPKDKDEDVTCNDD.GLCNGKYGGTSKS
NmTdfH_alpha710             834  AAYEPKKNLIFRAEVKNLFDRRYIDPLDAGNDAATQRYYSSFDPKDKDEDVTCNAD.GLCNGKYGGTSKS
NmTdfH_NZ-05/33             834  AAYEPKKNLIFRAEVKNLFDRRYIDPLDAGNDAATQRYYSSFDPKDKDEDVTCNAD.GLCNGKYGGTSKS
NmTdfH_93004                834  AAYEPKKNLIFRAEVKNLFDRRYIDPLDAGNDAATQRYYSSFDPKDKDEEVTCNADKTLCNGKYGGTSKS
NmTdfH_ATCC13091            834  AAYEPKKNLIFRAEVKNLFDRRYIDPLDAGNDAATQRYYSSFDPKDKDEEVTCNDDNTLCNGKYGGTSKS
NmTdfH_alpha522             834  AAYEPKKNLIFRAEVKNLFDRRYIDPLDAGNDAATQRYYSSFDPKDKDEDVTCNAD.GLCNGKYGGTSKS
NmTdfH_NM2795               834  AAYEPKKNLIFRAEVKNLFDRRYIDPLDAGNDAATQRYYSSFDPKDKDEEVTCNADKTLCNGKYGGTSKS
NmTdfH_81858                834  AAYEPKKNLIFRAEVKNLFDRRYIDPLDAGNDAATQRYYSSFDPKDKDEDVTCNADKTLCNGKYGGTSKS
NmTdfH_ERS514534            834  AAYEPKKNLIFRAEVKNLFDRRYIDPLDAGNDAATQRYYSSFDPKDKDEDVTCNADKTLCNGKYGGTSKS
NmTdfH_alpha153             834  AAYEPKKNLIFRAEVKNLFDRRYIDPLDAGNDAATQRYYSSFDPKDKDEDVTCNADKTLCNGKYGGTSKS
NmTdfH_053442               819  AAYEPKKNLIFRAEVKNLFDRRYIDPLDAGNDAATQRYYSSFDPKDKDEEVTCNADKTLCNGKYGGTSKS
NmTdfH_M0579                833  AAYEPKKNLIFRAEVKNLFDRRYIDPLDAGNDAATQRYYSSFDPKDKDEDVTCNAD.GLCNGKYGGTSKS
NmTdfH_MC58                 833  AAYEPKKNLIFRAEVKNLFDRRYIDPLDAGNDAATQRYYSSFDPKDKDEDVTCNADKTLCNGKYGGTSKS
NmTdfH_LNP21362             833  AAYEPKKNLIFRAEVKNLFDRRYIDPLDAGNDAATQRYYSSFDPKDKDEDVTCNADKTLCNGKYGGTSKS
NmTdfH_IR1074               833  AAYEPKKNLIFRAEVKNLFDRRYIDPLDAGNDAATQRYYSSFDPKDKDEDVTCNADKTLCNGKYGGTSKS
NmTdfH_H44/76               833  AAYEPKKNLIFRAEVKNLFDRRYIDPLDAGNDAATQRYYSSFDPKDKDEDVTCNADKTLCNGKYGGTSKS
NmTdfH_FDAARGOS_210         833  AAYEPKKNLIFRAEVKNLFDRRYIDPLDAGNDAATQRYYSSFDPKDKDEDVTCNAD.GLCNGKYGGTSKS
NmTdfH_FDAARGOS_215         834  AAYEPKKNLIFRAEVKNLFDRRYIDPLDAGNDAATQRYYSSFDPKDKDEEVTCNADKTLCNGKYGGTSKS
NmTdfH_                     834  AAYEPKKNLIFRAEVKNLFDRRYIDPLDAGNDAATQRYYSSFDPKDKDEEVTCNDDNTLCNGKYGGTSKS
NmTdfH_510612               817  AAYEPKKNLIFRAEVKNLFDRRYIDPLDAGNDAATQRYYSSFDPKDKDEEVTCNDDNTLCNGKYGGTSKS
NmTdfH_Z2491                834  AAYEPKKNLIFRAEVKNLFDRRYIDPLDAGNDAATQRYYSSFDPKDKDEEVTCNDDNTLCNGKYGGTSKS
NmTdfH_NCTC8249             834  AAYEPKKNLIFRAEVKNLFDRRYIDPLDAGNDAATQRYYSSFDPKDKDEDVTCNAD.GLCNGKYGGTSKS
NmTdfH_alpha14              834  AAYEPKKNLIFRAEVKNLFDRRYIDPLDAGNDAATQRYYSSFDPKDKDEEVTCNDDNTLCNGKYGGTSKS
NmTdfH_2842STDY5881378      834  AAYEPKKNLIFRAEVKNLFDRRYIDPLDAGNDAATQRYYSSFDPKDKDEDVTCNADKTLCNGKYGGTSKS
NmTdfH_CHUV                 834  AAYEPKKNLIFRAEVKNLFDRRYIDPLDAGNDAATQRYYSSFDPKDKDEDVTCNAD.GLCNGKYGGTSKS
NmTdfH_FAM18                834  AAYEPKKNLIFRAEVKNLFDRRYIDPLDAGNDAATQRYYSSFDPKDKDEDVTCNAD.GLCNGKYGGTSKS
NmTdfH_M09293               834  AAYEPKKNLIFRAEVKNLFDRRYIDPLDAGNDAATQRYYSSFDPKDKDEDVTCNAD.GLCNGKYGGTSKS
NmTdfH_M26503               834  AAYEPKKNLIFRAEVKNLFDRRYIDPLDAGNDAATQRYYSSFDPKDKDEDVTCNAD.GLCNGKYGGTSKS
NmTdfH_2842STDY5881093      834  AAYEPKKNLIFRAEVKNLFDRRYIDPLDAGNDAATQRYYSSFDPKDKDEDVTCNAD.GLCNGKYGGTSKS
NmTdfH_PMB5301              834  AAYEPKKNLIFRAEVKNLFDRRYIDPLDAGNDAATQRYYSSFDPKDKDEDVTCNAD.GLCNGKYGGTSKS
```

*NgTdfH_FA1090*

η8 β43

| | | |
|---|---|---|
| NgTdfH_FA1090 | 904 | VLTNFARGRTFLMTMSYKF |
| NgTdfH_WHO_M | 904 | VLTNFARGRTFLMTMSYKF |
| NgTdfH_WHO_O | 904 | VLTNFARGRTFLMTMSYKF |
| NgTdfH_WHO_U | 904 | VLTNFARGRTFLMTMSYKF |
| NgTdfH_WHO_P | 904 | VLTNFARGRTFLMTMSYKF |
| NgTdfH_MS11 | 904 | VLTNFARGRTFLMTMSYKF |
| NgTdfH_F62 | 904 | VLTNFARGRTFLMTMSYKF |
| NgTdfH_WHO_F | 887 | VLTNFARGRTFLMTMSYKF |
| NgTdfH_MIA_2011_03-10 | 911 | VLTNFARGRTFLMTMSYKF |
| NgTdfH_DGI18 | 904 | VLTNFARGRTFLMTMSYKF |
| NgTdfH_FA19 | 904 | VLTNFARGRTFLMTMSYKF |
| NgTdfH_WHO_L | 904 | VLTNFARGRTFLMTMSYKF |
| NgTdfH_DG12 | 898 | VLTNFARGRTFLMTMSYKF |
| NgTdfH_WHO_K | 887 | VLTNFARGRTFLMTMSYKF |
| NgTdfH_WHO_W | 887 | VLTNFARGRTFLMTMSYKF |
| NgTdfH_WHO_X | 887 | VLTNFARGRTFLMTMSYKF |
| NgTdfH_WHO_Z | 887 | VLTNFARGRTFLMTMSYKF |
| NgTdfH_WHO_G | 904 | VLTNFARGRTFLMTMSYKF |
| NgTdfH_WHO_N | 904 | VLTNFARGRTFLMTMSYKF |
| NgTdfH_NG-k51.05 | 911 | VLTNFARGRTFLMTMSYKF |
| NgTdfH_WHO_V | 887 | VLTNFARGRTFLMTMSYKF |
| NgTdfH_WHO_Y | 887 | VLTNFARGRTFLMTMSYKF |
| NgTdfH_BZG33 | 904 | VLTNFARGRTFLMTMSYKF |
| NgTdfH_NCCP11945 | 887 | VLTNFARGRTFLMTMSYKF |
| NmTdfH_020-06 | 902 | VLTNFARGRTFLMTMSYKF |
| NmTdfH_alpha710 | 903 | VLTNFARGRTFLITMSYKF |
| NmTdfH_NZ-05/33 | 903 | VLTNFARGRTFLITMSYKF |
| NmTdfH_93004 | 904 | VLTNFARGRTFLMTMSYKF |
| NmTdfH_ATCC13091 | 904 | VLTNFARGRTFLITMSYKF |
| NmTdfH_alpha522 | 903 | VLTNFARGRTFLITMSYKF |
| NmTdfH_NM2795 | 904 | VLTNFARGRTFLMTMSYKF |
| NmTdfH_81858 | 904 | VLTNFARGRTFLMTMSYKF |
| NmTdfH_ERS514534 | 904 | VLTNFARGRTFLMTMSYKF |
| NmTdfH_alpha153 | 904 | VLTNFARGRTFLMTMSYKF |
| NmTdfH_053442 | 889 | VLTNFARGRTFLMTMSYKF |
| NmTdfH_M0579 | 902 | VLTNFARGRTFLITMSYKF |
| NmTdfH_MC58 | 903 | VLTNFARGRTFLMTMSYKF |
| NmTdfH_LNP21362 | 903 | VLTNFARGRTFLMTMSYKF |
| NmTdfH_IR1074 | 903 | VLTNFARGRTFLMTMSYKF |
| NmTdfH_H44/76 | 903 | VLTNFARGRTFLMTMSYKF |
| NmTdfH_FDAARGOS_210 | 902 | VLTNFARGRTFLMTMSYKF |
| NmTdfH_FDAARGOS_215 | 904 | VLTNFARGRTFLITMSYKF |
| NmTdfH_ | 904 | VLTNFARGRTFLITMSYKF |
| NmTdfH_510612 | 887 | VLTNFARGRTFLITMSYKF |
| NmTdfH_Z2491 | 904 | VLTNFARGRTFLITMSYKF |
| NmTdfH_NCTC8249 | 903 | VLTNFARGRTFLMTMSYKF |
| NmTdfH_alpha14 | 904 | VLTNFARGRTFLITMSYKF |
| NmTdfH_2842STDY5881378 | 904 | VLTNFARGRTFLMTMSYKF |
| NmTdfH_CHUV | 903 | VLTNFARGRTFLMTMSYKF |
| NmTdfH_FAM18 | 903 | VLTNFARGRTFLMTMSYKF |
| NmTdfH_M09293 | 903 | VLTNFARGRTFLMTMSYKF |
| NmTdfH_M26503 | 903 | VLTNFARGRTFLMTMSYKF |
| NmTdfH_2842STDY5881093 | 903 | VLTNFARGRTFLITMSYKF |
| NmTdfH_PMB5301 | 903 | VLTNFARGRTFLITMSYKF |

## Supplementary Figure 7. Sequence alignment of ZnuDs.

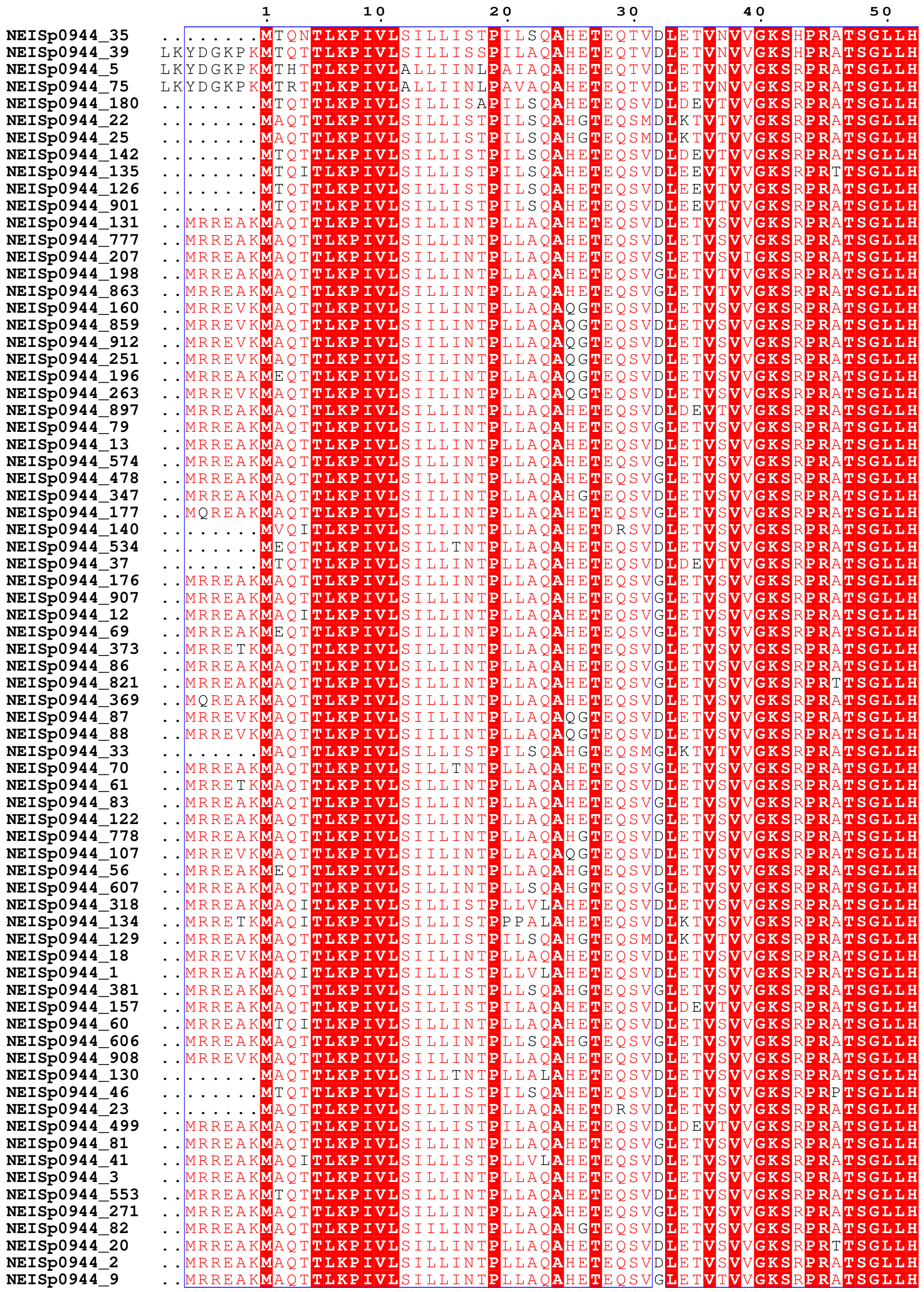

```
                   60        70        80        90        100       110
NEISp0944_35   TSTASDKIIAGDTLRQKSVNLGDALNGVPGVHASQYGGGASAPVIRGQTGRRIKVLNHHG
NEISp0944_39   SSTASDKIIAGDTLRQKAVNLGDALNGVPGVHASQYGGGASAPVIRGQTGRRIKVLNHHG
NEISp0944_5    TSTASDKIIAGDTLRQKAVNLGDALNGVPGVHASQYGGGASAPVIRGQTGRRIKVLNHHG
NEISp0944_75   SSTASDKIISGDTLRQKAVNLGDALNGVPGVHASQYGGGASAPVIRGQTGRRIKVLNHHG
NEISp0944_180  TSTASDKIISGDILRQKAVNLGDALDGVPGIHASQYGGGASAPVIRGQTGRRIKVLNHHG
NEISp0944_22   TSTASDKIISGDTLRQKAVNLGDALDGVPGIHASQYGGGASAPVIRGQTGRRIKVLNHHG
NEISp0944_25   TSTASDKIISGDTLRQKAVNLGDALDGVPGIHASQYGGGASAPVIRGQTGRRIKVLNHHG
NEISp0944_142  TSTASDKIISGDTLRQKAVNLGDALDGVPGIHASQYGGGASAPVIRGQTGRRIKVLNHHG
NEISp0944_135  TSTASDKIISGDTLRQKAVNLGDALDGVPGIHASQYGGGASAPVIRGQTGRRIKVLNHHG
NEISp0944_126  TSTASDKIISGDTLRQKAVNLGDALDGVPGIHASQYGGGASAPVIRGQTGRRIKVLNHHG
NEISp0944_901  ASTASDKIISGDTLRQKAVNLGDALDGVPGIHASQYGGGASAPVIRGQTGRRIKVLNHHG
NEISp0944_131  TSTASDKIISGDTLRQKAVNLGDALDGVPGIHASQYGGGASAPVIRGQTGRRIKVLNHHG
NEISp0944_777  TSTASDKIISGDTLRQKAVNLGDALDGVPGIHASQYGGGASAPVIRGQTGRRIKVLNHHG
NEISp0944_207  TSTASDKIISGDTLRQKAVNLGDALDGVPGIHASQYGGGASAPVIRGQTGRRIKVLNHHG
NEISp0944_198  TSTASDKIISGDTLRQKAVNLGDALDGVPGIHASQYGGGASAPVIRGQTGRRIKVLNHHG
NEISp0944_863  TSTASDKIISGDTLRQKAVNLGDALDGVPGIHASQYGGGASAPVIRGQTGRRIKVLNHHG
NEISp0944_160  TSTASDKIISGDTLRQKAVNLGDALDGVPGIHASQYGGGASAPVIRGQTGRRIKVLNHHG
NEISp0944_859  TSTASDKIISGDTLRQKAVNLGDALDGVPGIHASQYGGGASAPVIRGQTGRRIKVLNHHG
NEISp0944_912  TSTASDKIISGDTLRQKAVNLGDALDGVPGIHASQYGGGASAPVIRGQTGRRIKVLNHHG
NEISp0944_251  TSTASDKIISGDTLRQKAVNLGDALDGVPGIHASQYGGGASAPVIRGQTGRRIKVLNHHG
NEISp0944_196  TSTASDKIISGDTLRQKAVNLGDALDGVPGIHASQYGGGASAPVIRGQTGRRIKVLNHHG
NEISp0944_263  TSTASDKIISGDTLRQKAVNLGDALDGVPGIHASQYGGGASAPVIRGQTGRRIKVLNHHG
NEISp0944_897  TSTASDKILSGDTLRQKAVNLGDALDGVPGIHASQYGGGASAPVIRGQTGRRIKVLNHHG
NEISp0944_79   TSSSDKIISGDTLRQKAVNLGDALDGVPGIHASQYGGGASAPVIRGQTGRRIKVLNHHG
NEISp0944_13   TSTASDKIISGDTLRQKAVNLGDALDGVPGIHASQYGGGASAPVIRGQTGRRIKVLNHHG
NEISp0944_574  TSTASDKIISGDTLRQKAVNLGDALDGVPGIHASQYGGGASAPVIRGQTGRRIKVLNHHG
NEISp0944_478  TSTASDKIISGDTLRQKAVNLGDALDGVPGIHASQYGGGASAPVIRGQTGRRIKVLNHHG
NEISp0944_347  TSTASDKIISGDTLRQKAVNLGDALDGVPGIHASQYGGGASAPVIRGQTGRRIKVLNHHG
NEISp0944_177  TSTASDKIISGDTLRQKAVNLGDALDGVPGIHASQYGGGASAPVIRGQTGRRIKVLNHHG
NEISp0944_140  TSTASDKIISGDTLRQKAVNLGDALDGVPGIHASQYGGGASAPVIRGQTGRRIKVLNHHG
NEISp0944_534  TSTASDKIISGDTLRQKAVNLGDALDGVPGIHASQYGGGASAPVIRGQTGRRIKVLNHHG
NEISp0944_37   TSTASDKILSGDTLRQKAVNLGDALDGVPGIHASQYGGGASAPVIRGQTGRRIKVLNHHG
NEISp0944_176  TSTASDKIISGDTLRQKAVNLGDALDGVPGIHASQYGGGASAPVIRGQTGRRIKVLNHHG
NEISp0944_907  TSTASDKIISGDTLRQKAVNLGDALDGVPGIHASQYGGGASAPVIRGQTGRRIKVLNHHG
NEISp0944_12   TSTASDKIISGDTLRQKAVNLGDALDGVPGIHASQYGGGASAPVIRGQTGRRIKVLNHHG
NEISp0944_69   TSTASDKIISGDTLRQKAVNLGDALDGVPGIHASQYGGGASAPVIRGQTGRRIKVLNHHG
NEISp0944_373  TSTASDKIISGDTLRQKAVNLGDALDGVPGIHASQYGGGASAPVIRGQTGRRIKVLNHHG
NEISp0944_86   TSTASDKIISGDTLRQKAVNLGDALDGVPGIHASQYGGGASAPVIRGQTGRRIKVLNHHG
NEISp0944_821  TSTASDKIISGDTLRQKAVNLGDALDGVPGIHASQYGGGASAPVIRGQTGRRIKVLNHHG
NEISp0944_369  TSTASDKIISGDTLRQKAVNLGDALDGVPGIHASQYGGGASAPVIRGQTGRRIKVLNHHG
NEISp0944_87   TSTASDKIISGDTLRQKAVNLGDALDGVPGIHASQYGGGASAPVIRGQTGRRIKVLNHHG
NEISp0944_88   TSTASDKIISGDTLRQKAVNLGDALDGVPGIHASQYGGGASAPVIRGQTGRRIKVLNHHG
NEISp0944_33   TSTASDKIISGDTLRQKAVNLGDALDGVPGIHASQYGGGASAPVIRGQTGRRIKVLNHHG
NEISp0944_70   TSTASDKIISGDTLRQKAVNLGDALDGVPGIHASQYGGGASAPVIRGQTGRRIKVLNHHG
NEISp0944_61   TSTASDKIISGDTLRQKAVNLGDALDGVPGIHASQYGGGASAPVIRGQTGRRIKVLNHHG
NEISp0944_83   TSTASDKIISGDTLRQKAVNLGDALDGVPGIHASQYGGGASAPVIRGQTGRRIKVLNHHG
NEISp0944_122  TSTASDKIISGDTLRQKAVNLGDALDGVPGIHASQYGGGASAPVIRGQTGRRIKVLNHHG
NEISp0944_778  TSTASDKIISGDTLRQKAVNLGDALDGVPGIHASQYGGGASAPVIRGQTGRRIKVLNHHG
NEISp0944_107  TSTASDKIISGDTLRQKAVNLGDALDGVPGIHASQYGGGASAPVIRGQTGRRIKVLNHHG
NEISp0944_56   TSTASDKIISGDTLRQKAVNLGDALDGVPGIHASQYGGGASAPVIRGQTGRRIKVLNHHG
NEISp0944_607  TSTASDKIISGDTLRQKAVNLGDALDGVPDIHASQYGGGASAPVIRGQTGRRIKVLNHHG
NEISp0944_318  ASTSSDKIISGDTLRQKAVNLGDALDGVPGIHASQYGGGASAPVIRGQTGRRIKVLNHHG
NEISp0944_134  TSTASDKILSGDTLRQKAVNLGDALDGVPGIHASQYGGGSSAPVIRGQTGRRIKVLNHHG
NEISp0944_129  TSTASDKIISGDTLRQKAVNLGDALDGVPGVHASQYGGGASAPVIRGQTGRRIKVLNHHG
NEISp0944_18   TSTASDKIISGDTLRQKAVNLGDALDGVPGIHASQYGGGASAPVIRGQTGRRIKVLNHHG
NEISp0944_1    ASTSSDKIISGDTLRQKAVNLGDALDGVPGIHASQYGGGASAPVIRGQTGRRIKVLNHHG
NEISp0944_381  TSTASDKIISGDTLRQKAVNLGDALDGVPGIHASQYGGGASAPVIRGQTGRRIKVLNHHG
NEISp0944_157  TSTASDKILSGDTLRQKAVNLGDALDGVPGIHASQYGGGASAPVIRGQTGRRIKVLNHHG
NEISp0944_60   TSTASDKIISGDTLRQKAVNLGDALDGVPGIHASQYGGGASAPVIRGQTGRRIKVLNHHG
NEISp0944_606  TSTASDKIISGDTLRQKAVNLGDALDGVPGIHASQYGGGASAPVIRGQTGRRIKVLNHHG
NEISp0944_908  TSTASDKIISGDTLHQKAVNLGDALDGVPGIHASQYGGGASAPVIRGQTGRRIKVLNHHG
NEISp0944_130  TSTASDKIISGDTLRQKAVNLGDALDGVPGIHASQYGGGASAPVIRGQTGRRIKVLNHHG
NEISp0944_46   TSTASDKIISGDTLRQKAVNLGDALDGVPGIHASQYGGGASAPVIRGQTGRRIKVLNHHG
NEISp0944_23   TSTASDKIISGDTLRQKAVNLGDALDGVPGIHASQYGGGASAPVIRGQTGRRIKVLNHHG
NEISp0944_499  TSTASDKILSGDTLRQKAVNLGDALDGVPGIHASQYGGGASAPVIRGQTGRRIKVLNHHG
NEISp0944_81   TSTASDKIISGDTLHQKAVNLGDALDGVPGIHASQYGGGASAPVIRGQTGRRIKVLNHHG
NEISp0944_41   ASTSSDKIISGDTLRQKAVNLGDALDGVPGIHASQYGGGASAPVIRGQTGRRIKVLNHHG
NEISp0944_3    TSTASDKIISGDTLRQKAVNLGDALDGVPGIHASQYGGGASAPVIRGQTGRRIKVLNHHG
NEISp0944_553  TSTASDKIISGDTLRQKAVNLGDALDGVPGIHASQYGGGASAPVIRGQTGRRIKVLNHHG
NEISp0944_271  TSTASDKIISGDTLRQKAVNLGDALDGVPGIHASQYGGGASAPVIRGQTGRRIKVLNHHG
NEISp0944_82   TSTASDKIISGDTLRQKAVNLGDALDGVPGVHASQYGGGASAPVIRGQTGRRIKVLNHHG
NEISp0944_20   TSTASDKIISGDTLRQKAVNLGDALDGVPGIHASQYGGGASAPVIRGQTGRRIKVLNHHG
NEISp0944_2    TSTASDKILSGDTLRQKAVNLGDALDGVPGIHASQYGGGASAPVIRGQTGRRIKVLNHHG
NEISp0944_9    TSTASDKIISGDTLRQKAVNLGDALDGVPGIHASQYGGGASAPVIRGQTGRRIKVLNHHG
```

```
                        120       130       140       150       160       170
                           .         .         .         .         .         .
NEISp0944_35       ETGDMADFSPDHALMVDTALSQQVEILRGPVTLLYSSGNVAGLVDVADGKIPEKMPENGV
NEISp0944_39       ETGDMADFSPDHALMVDTALSQQVEILRGPVTLLYSSGNVAGLVDVADGKIPEKMPENGV
NEISp0944_5        ETGDMADFSPDHALMVDTALSQQVEILRGPVTLLYSSGNVAGLVDVADGKIPEKMPENGV
NEISp0944_75       ETGDMADFSPDHALMVDTALSQQVEILRGPVTLLYSSGNVAGLVDVADGKIPEKMPEKGV
NEISp0944_180      ETGDMADFSPDHALMVDSALSQQVEILRGPVTLLYSSGNVAGLVDVADGKIPEKMPENGV
NEISp0944_22       ETGDMADFSPDHAIMVDSALSQQVEILRGPVTLLYSSGNVAGLVDVADGKIPEKMPENGV
NEISp0944_25       ETGDMADFSPDHAIMVDSALSQQVEILRGPVTLLYSSGNVAGLVDVADGKIPEKMPENGV
NEISp0944_142      ETGDMADFSPDHALMVDSALSQQVEILRGPVTLLYSSGNVAGLVDVADGKIPEKMPANGV
NEISp0944_135      ETGDMADFSPDHALMVDSALSQQIEILRGPVTLLYSSGNVAGLVDVADGKIPEKMPENGV
NEISp0944_126      ETGDMADFSPDHAIMVDSALSQQVEILRGPVTLLYSSGNVAGLVDVADGKIPEKMPENGV
NEISp0944_901      ETGDMADFSPDHALMVDSALSQQVEILRGPVTLLYSSGNVAGLVDVADGKIPEKMPENGV
NEISp0944_131      ETGDMADFSPDHAIMVDSALSQQVEILRGPVTLLYSSGNVAGLVDVADGKIPEKMPENGV
NEISp0944_777      ETGDMADFSPDHALMVDSALSQQVEILRGPVTLLYSSGNVASLVDVADGKIPEKMPENGV
NEISp0944_207      ETGDMADFSPDHVLMVDSALSQQVEILRGPVTLLYSSGNVAGLVDVADGKIPEKMPENGV
NEISp0944_198      ETGDMADFSPDHAIMVDTALSQQVEILRGPVTLLYSSGNVAGLVDVADGKIPEKMPENGV
NEISp0944_863      ETGDMADFSPDHAIMVDTALSQQVEILRGPVTLLYSSGNVAGLVDVADGKIPEKMPENGV
NEISp0944_160      ETGDMADFSPDHAIMVDTALSQQVEILRGPVTLLYSSGNVAGLVDVADGKIPEKMPENGV
NEISp0944_859      ETGDMADFSPDHAIMVDTALSQQVEILRGPVTLLYSSGNVAGLVDVADGKIPEKMPENGV
NEISp0944_912      ETGDMADFSPDHAIMVDTALSQQVEILRGPVTLLYSSGNVAGLVDVADGKIPEKMPENGV
NEISp0944_251      ETGDMADFSPDHAIMVDTALSQQVEILRGPVTLLYSSGNVAGLVDVADGKIPEKMPENGV
NEISp0944_196      ETGDMADFSPDHAIMVDTALSQQVEILRGPVTLLYSSGNVAGLVDVADGKIPEKMPENGV
NEISp0944_263      ETGDMADFSPDHAIMVDTALSQQVEILRGPITLLYSSGNVAGLVDVADGKIPEKMPENGV
NEISp0944_897      ETGDMADFSPDHAIMVDTALSQQVEILRGPVTLLYSSGNVAGLVDVADNKIPEKMPENGV
NEISp0944_79       ETGDMADFSPDHAIMVDTALSQQVEILRGPVTLLYSSGNVAGLVDVADNKIPEKMPENGV
NEISp0944_13       ETGDMADFSPDHAIMVDTALSQQVEILRGPVTLLYSSGNVAGLVDVADNKIPEKMPENGV
NEISp0944_574      ETGDMADFSPDHAIMVDTALSQQVEILRGPVTLLYSSGNVAGLVDVADNKIPEKMPENGV
NEISp0944_478      ETGDMADFSPDHAIMVDTALSQQVEILRGPVTLLYSSGNVAGLVDVADGKIPEKMPENGV
NEISp0944_347      ETGDMADFSPDHAIMVDTALSQQVEILRGPVTLLYSSGNVAGLVDVADGKIPEKMPENGV
NEISp0944_177      ETGDMADFSPDHAIMVDTALSQQVEILRGPVTLLYSSGNVAGLIDVADGKIPEKMPENGV
NEISp0944_140      ETGDMADFSPDHAIMVDTALSQQVEILRGPVTLLYSSGNVAGLVDVADGKIPEKMPENGV
NEISp0944_534      ETGDMADFSPDHAIMVDTALSQQVEILRGPVTLLYSSGNVAGLVDVADGKIPEKMPENGV
NEISp0944_37       ETGDMADFSPDHAIMVDTALSQQVEILRGPVTLLYSSGNVAGLIDVADGKIPEKMPENGV
NEISp0944_176      ETGDMADFSPDHAIMVDTALSQQVEILRGPVTLLYSSGNVAGLIDVADGKIPEKMPENSV
NEISp0944_907      ETGDMADFSPDHAIMVDTALSQQVEILRGPVTLLYSSGNVAGLIDVADGKIPEKMPENGV
NEISp0944_12       ETGDMADFSPDHAIMVDTALSQQVEILRGPVTLLYSSGNVAGLVDVADGKIPEKMPENGV
NEISp0944_69       ETGDMADFSPDHAIMVDTALSQQVEILRGPVTLLYSSGNVAGLVDVADGKIPEKMPENGV
NEISp0944_373      ETGDMADFSPDHAIMVDTALSQQVEILRGPVTLLYSSGNVAGLVDVADGKIPEKMPENGV
NEISp0944_86       ETGDMADFSPDHAIMVDTALSQQVEILRGPVTLLYSSGNVAGLIDVADGKIPEKMPENGV
NEISp0944_821      ETGDMADFSPDHAIMVDTALSQQVEILRGPVTLLYSSGNVAGLIDVADGKIPEKMPENGV
NEISp0944_369      ETGDMADFSPDHAIMVDTALSQQVEILRGPVTLLYSSGNVAGLIDVADGKIPEKMPENGV
NEISp0944_87       ETGDMADFSPDHAIMVDTALSQQVEILRGPVTLLYSSGNVAGLVDVADGKIPEKMPENGV
NEISp0944_88       ETGDMADFSPDHAIMVDTALSQQVEILRGPITLLYSSGNVAGLVDVADGKIPEKMPENGV
NEISp0944_33       ETGDMADFSPDHAIMVDTALSQQVEILRGPVTLLYSSGNVAGLVDVADGKIPEKMPENGV
NEISp0944_70       ETGDMADFSPDHAIMVDTALSQQVEILRGPVTLLYSSGNVAGLVDVADGKIPEKMPENGV
NEISp0944_61       ETGDMADFSPDHAIMVDTALSQQVEILRGPVTLLYSSGNVAGLVDVADNKIPEKMPENGV
NEISp0944_83       ETGDMADFSPDHAIMVDTALSQQVEILRGPVTLLYSSGNVAGLVDVADNKIPEKMPENGV
NEISp0944_122      ETGDMADFSPDHAIMVDTALSQQVEILRGPVTLLYSSGNVAGLVDVADNKIPEKMPENGV
NEISp0944_778      ETGDMADFSPDHAIMVDTALSQQVEILRGPVTLLYSSGNVAGLVDVADGKIPEKMPENGV
NEISp0944_107      ETGDMADFSPDHAIMVDTALSQQVEILRGPVTLLYSSGNVAGLVDVADGKIPEKMPENGV
NEISp0944_56       ETGDMADFSPDHAIMVDTALSQQVEILRGPVTLLYSSGNVAGLVDVADGKIPEKMPENGV
NEISp0944_607      ETGDMADFSPDHAIMVDSALSQQVEILRGPVTLLYSSGNVAGLVDVADNKIPEKMPENGV
NEISp0944_318      ETGDMADFSPDHAIMVDTALSQQVEILRGPVTLLYSSGNVAGLVDVTDGKIPEKMPENGV
NEISp0944_134      ETGDMADFSPDHAIMVDTALSQQVEILRGPVTLLYSSGNVAGLVDVADGKIPEKMPENGV
NEISp0944_129      ETGDMADFSPDHAIMVDTALSQQVEILRGPVTLLYSSGNVAGLVDVADGKIPEKMPENGV
NEISp0944_18       ETGDMADFSPDHALMVDSALSQQVEILRGPVTLLYSSGNVAGLVDVADGKIPEKMPENGV
NEISp0944_1        ETGDMADFSPDHAIMVDTALSQQVEILRGPVTLLYSSGNVAGLVDVTDGKIPEKMPENGV
NEISp0944_381      ETGDMADFSPDHAIMVDSALSQQVEILRGPVTLLYSSGNVAGLVDVADGKIPEKMPENGV
NEISp0944_157      ETGDMADFSPDHAIMVDTALSQQVEILRGPVTLLYSSGNVAGLVDVADGKIPEKMPENGV
NEISp0944_60       ETGDMADFSPDHAIMVDTALSQQVEILRGPVTLLYSSGNVAGLVDVADGKIPEKMPENGV
NEISp0944_606      ETGDMADFSPDHAIMVDTALSQQVEILRGPVTLLYSSGNVAGLVDVADGKIPEKMPENGV
NEISp0944_908      ETGDMADFSPDHAIMVDTALSQQVEILRGPVTLLYSSGNVAGLVDVADGKIPEKMPENGV
NEISp0944_130      ETGDMADFSPDHALMVDSALSQQVEILRGPVTLLYSSGNVAGLVDVADGKIPEKMPENGV
NEISp0944_46       ETGDMADFSPDHAIMVDTALSQQVEILRGPVTLLYSSGNVAGLVDVADGKIPEKMPENGV
NEISp0944_23       ETGDMADFSPDHAIMVDTALSQQVEILRGPVTLLYSSGNVAGLVDVADGKIPEKMPENGV
NEISp0944_499      ETGDMADFSPDHAIMVDTALSQQVEILRGPVTLLYSSGNVAGLVDVADGKIPEKMPENGV
NEISp0944_81       ETGDMADFSPDHAIMVDTALSQQVEILRGPVTLLYSSGNVAGLVDVADGKIPEKMPENGV
NEISp0944_41       ETGDMADFSPDHAIMVDTALSQQVEILRGPVTLLYSSGNVAGLVDVADSKIPEKMPENGV
NEISp0944_3        ETGDMADFSPDHAIMVDSALSQQVEILRGPVTLLYSSGNVAGLVDVADGKIPEKMPENGV
NEISp0944_553      ETGDMADFSPDHAIMVDTALSQQVEILRGPVTLLYSSGNVAGLVDVADGKIPEKMPENGV
NEISp0944_271      ETGDMADFSPDHAIMVDTALSQQVEILRGPVTLLYSSGNVAGLIDVADGKIPEKMPENGV
NEISp0944_82       ETGDMADFSPDHAIMVDTALSQQVEILRGPITLLYSSGNVAGLVDVADGKIPEKMPENGV
NEISp0944_20       ETGDMADFSPDHAIMVDTALSQQVEILRGPVTLLYSSGNVAGLVDVADGKIPEKMPENGV
NEISp0944_2        ETGDMADFSPDHAIMVDTALSQQVEILRGPVTLLYSSGNVAGLVDVADGKIPEKMPENGV
NEISp0944_9        ETGDMADFSPDHAIMVDTALSQQVEILRGPVTLLYSSGNVAGLVDVADGKIPEKMPENGV
```

```
                           180       190       200       210       220       230
                              .         .         .         .         .         .
NEISp0944_35        SGELGLRLSSGNLEKLTSGGINVGLGKNFVLHTEGLYRKSGDYAVPRYRKEEGRLKRLPD
NEISp0944_39        SGELGLRLSSGNLEKLTSGGINVGLGKNFVLHTEGLYRKSGDYAVPRYRKEEGRLKRLPD
NEISp0944_5         SGELGLRLSSGNLEKLTSGGINVGLGKNFVLHTEGLYRKSGDYAVPRYRKEEGRLKRLPD
NEISp0944_75        SGELGLRLSSGNLEKLTSGGINFGLGKNFVLHTEGLYRKSGDYAVPRYRKEEGRLKRLPD
NEISp0944_180       SGEAGLRLSSGNLEKLTSAGINIGLGKNFVLHTEGLYRKSGDYAVPRYQKEEGRLKRLPD
NEISp0944_22        SGEAGLRLSSGNLEKLTSAGINIGLGNNFVLHTEGLYRKSGDYAVPRYQKEKGRLKRLPD
NEISp0944_25        SGETGLRLSSGNLEKLTSAGINIGLGNNFVLHTEGLYRKSGDYAVPRYQKEKGRLKRLPD
NEISp0944_142       SGEAGLRLSSGNLEKLTSASINIVLGKNFVLHTEGLYRKSGDYAIPRYHKEEGRLKRLPD
NEISp0944_135       SGEAGLRLSSGNLEKLTSAGINIGLGNNFVLHTEGLYRKSGDYAVPRYQKEEGRLKRLPD
NEISp0944_126       SGEAGLRLSSGNLEKLTSAGINIGLGNNFVLHTEGLYRKSGDYAVPHYQKEEGRLKRLPD
NEISp0944_901       SGEAGLRLSSGNLEKLTSAGINIGLGKNFVLHTEGLYRKSGDYAVPRYQKEEGRLKRLPD
NEISp0944_131       SGELGLRLSSGNLEKLTSGDINIGLGKNFVLHTEGLYRKSGDYAVPRYQKEEGRLKRLPD
NEISp0944_777       SGELGLRLSSGNLEKLTSGDINIGLGKNFVLHTEGLYRKSGDYAVPRYQKEEGRLKRLPD
NEISp0944_207       SGEAGLRLSSGNLEKLTSGGINIGLGNNFVLHTEGLYRKSGDYAVPRYRN....LKRLPD
NEISp0944_198       SGELGLRLSSGNLEKLTSGGINIGLGKNFVLHTESLYRKSGDYAVPRYRN....LKRLPD
NEISp0944_863       SGELGLRLSSGNLEKLTSGGINIGLGKNFVLHTESLYRKSGDYAVPRYRN....LKRLPD
NEISp0944_160       SGELGLRLSSGNLEKLTSGGINIGLGNNFVLHTEGLYRKSGDYAVPRYRN....LKRLPD
NEISp0944_859       SGELGLRLSSGNLEKLTSGGINIGLGNNFVLHTEGLYRKSGDYAVPRYRN....LKRLPD
NEISp0944_912       SGEAGLRLSSGNLEKLTSAGINIGLGKNFVLHTEGLYRKSGDYAVPRYRN....LKRLPD
NEISp0944_251       SGEAGLRLSSGNLEKLTSAGINIGLGKNFVLHTEGLYRKSGDYAVPRYRN....LKRLPD
NEISp0944_196       SGEAGLRLSSGNLEKLTSAGINIGLGKNFVLHTEGLYRKSGDYAVPRYRN....LKRLPD
NEISp0944_263       SGELGLRLSSGNLEKLTSGGINIGLGKNFVLHTEGLYRKSGDYAVPRYRN....LKRLPD
NEISp0944_897       SGEAGLRLSSGNLEKLTSAGINIGLGKNFVLHTEGLYRKSGDYAVPRYRN....LKRLPD
NEISp0944_79        SGEAGLRLSSGNLEKLTSAGINIGLGKNFVLHTEGLYRKSGDYAVPRYRN....LKRLPD
NEISp0944_13        SGEAGLRLSSGNLEKLTSAGINIGLGKNFVLHTEGLYRKSGDYAVPRYRN....LKRLPD
NEISp0944_574       SGEAGLRLSSGNLEKLTSGGINIGLGKNFVLHTEGLYRKSGDYAVPRYRN....LKRLPD
NEISp0944_478       SGELGLRLSSGNLEKLTSGGINIGLGKNFVLHTEGLYRKSGDYAVPRYRN....LKRLPD
NEISp0944_347       SGELGLRLSSGNLEKLTSGGINIGLGKNFVLHTEGLYRKSGDYAVPRYRN....LKRLPD
NEISp0944_177       SGELGLRLSSGNLEKLTSGGINIGLGNNFVLHTEGLYRKSGDYAVPRYRN....LKRLPN
NEISp0944_140       SGELGLRLSSGNLEKLTSGGINIGLGKNFVLHTEGLYRKSGDYAVPRYRN....LKRLPD
NEISp0944_534       SGELGLRLSSGNLEKLTSGGINIGLGKNFVLHTEGLYRKSGDYAVPRYRN....LKRLPD
NEISp0944_37        SGELGLRLSSGNLEKLTSGGINIGLGNNFVLHTEGLYRKSGDYAVPRYRN....LKRLPD
NEISp0944_176       SGELGLRLSSGNLEKLTSGGINIGLGKNFVLHTESLYRKSGDYAVPRYRN....LKRLPD
NEISp0944_907       SGELGLRLSSGNLEKLTSGGINIGLGNNFVLHTEGLYRKSGDYAVPRYRN....LKRLPD
NEISp0944_12        SGEAGLRLSSGNLEKLTSAGINIGLGKNFVLHTEGLYRKSGDYAVPRYRN....LKRLPD
NEISp0944_69        SGEAGLRLSSGNLEKLTSGGINIGLGNNFVLHTEGLYRKSGDYAVPRYRN....LKRLPD
NEISp0944_373       SGELGLRLSSGNLEKLTSGGINIGLGKNFVLHTEGLYRKSGDYAVPRYRN....LKRLPD
NEISp0944_86        SGELGLRLSSGNLEKLTSGGINIGLGKNFVLHTESLYRKSGDYAVPRYRN....LKRLPD
NEISp0944_821       SGELGLRLSSGNLEKLTSGGINIGLGKNFVLHTESLYRKSGDYAVPRYRN....LKRLPD
NEISp0944_369       SGELGLRLSSGNLEKLTSGGINIGLGNNFVLHTEGLYRKSGDYAVPRYRN....LKRLPN
NEISp0944_87        SGELGLRLSSSNLEKLTSGGINVGLGKNFVLHTEGLYRKSDDYAVPRYRN....LKRLPD
NEISp0944_88        SGELGLRLSSGNLEKLTSGDINIGLGKNFVLHTEGLYRKSGDYAVPRYRN....LKRLPD
NEISp0944_33        SGEAGLRLSSGNLEKLTSAGINIDLGKNFVLHTEGLYRKSGDYAVPRYRN....LKRLPD
NEISp0944_70        SGELGLRLSSGNLEKLTSGGINIGLGKNFVLHTEGLYRKSGDYAVPRYQKEEGRLKRLPD
NEISp0944_61        SGEAGLRLSSGNLEKLTSAGINIGLGKNFVLHTEGLYRKSGDYAVPRYRN....LKRLPD
NEISp0944_83        SGEAGLRLSSGNLEKLTSGGINIGLGKNFVLHTEGLYRKSGDYAVPRYRN....LKRLPD
NEISp0944_122       SGEAGLRLSSGNLEKLTSGGINIGLGKNFVLHTEGLYRKSGDYAVPRYRN....LKRLPD
NEISp0944_778       SGEAGLRLSSGNLEKLTSGGINVGLGKNFVLHTEGLYRKSGDYAVPRYRKEEGRLKRLPD
NEISp0944_107       SGELGLRLSSGNLEKLTSGDINIGLGKNFVLHTEGLYRKSGDYAVPRYRN....LKRLPD
NEISp0944_56        SGELGLRLSSGNLEKLTSGGINIGLGNNFVLHTEGLYRKSGDYAVPRYRN....LKRLPD
NEISp0944_607       SGEAGLRLSSGNLEKLTSAGINIGLGKNFVLHTEGLYRKSGDYAVPRYRN....LKRLPD
NEISp0944_318       SGEAGLRLSSGNLEKLTSAGINIGLGNNFVLHTEGLYRKSGDYAVPRYRN....LKRLPD
NEISp0944_134       SGELGLRLSSGNLEKLTSGGINIGLGKNFVLHTEGLYRKSGDYAVPRYRN....LKRLPD
NEISp0944_129       SGELGLRLSSGNLEKLTSGGINIGLGKNFVLHTESLYRKSGDYAVPRYRN....LKRLPD
NEISp0944_18        SGELGLRLSSGNLEKLTSAGINIGLGNNFVLHTEGLYRKSGDYAVPRYQKEKGRLKRLPD
NEISp0944_1         SGEAGLRLSSGNLEKLTSAGINIGLGNNFVLHTEGLYRKSGDYAVPRYRN....LKRLPD
NEISp0944_381       SGELGLRLSSGNLEKLTSGGINIGLGKNFVLHTEGLYRKSGDYAIPRYRN....LKRLPD
NEISp0944_157       SGELGLRLSSGNLEKLTSGGINIGLGKNFVLHTEGLYRKSGDYAVPRYRN....LKRLPD
NEISp0944_60        SGELGLRLSSGNLEKLTSGGINVDLGKNFVLHTEGLYRKSGDYAVPRYRN....LKRLPD
NEISp0944_606       SGELGLRLSSGNLEKLTSGGINIGLGKNFVLHTEGLYRKSGDYAVPRYRN....LKRLPD
NEISp0944_908       SGELGLRLSSGNLEKLTSGGINIGLGKNFVLHTEGLYRKSGDYAVPRYRN....LKRLPD
NEISp0944_130       SGEAGLRLSSGNLEKLTSAGINIGLGNNFVLHTEGLYRKSGDYAVPRYRN....LKRLPD
NEISp0944_46        SGELGLRLSSGNLEKLTSGGINIGLGKNFVLHTEGLYRKSGDYAVPRYRN....LKRLPD
NEISp0944_23        SGELGLRLSSGNLEKLTSGGINIGLGKNFVLHTEGLYRKSGDYAVPRYRN....LKRLPD
NEISp0944_499       SGELGLRLSSGNLEKLTSGGINIGLGKNFVLHTEGLYRKSGDYAVPRYRN....LKRLPD
NEISp0944_81        SGELGLRLSSGNLEKLTSGGINIGLGKNFVLHTEGLYRKSGDYAVPRYRN....LKRLPD
NEISp0944_41        SGEAGLRLSSGNLEKLTSAGINIGLGKNFVLHTEGLYRKSGDYAVPRYRN....LKRLPD
NEISp0944_3         SGELGLRLSSGNLEKLTSGGINIGLGKNFVLHTEGLYRKSGDYAVPRYRN....LKRLPD
NEISp0944_553       SGEAGLRLSSGNLEKLTSAGINIGLGKNFVLHTEGLYRKSGDYAVPRYRN....LKRLPD
NEISp0944_271       SGELGLRLSSGNLEKLTSGGINIGLGKNFVLHTESLYRKSGDYAVPRYRN....LKRLPD
NEISp0944_82        SGEAGLRLSSGNLEKLTSGGINIGLGKNFVLHTEGLYRKSGDYAVPRYRN....LKRLPD
NEISp0944_20        SGELGLRLSSGNLEKLTSGGINIGLGKNFVLHTEGLYRKSGDYAVPRYRN....LKRLPD
NEISp0944_2         SGELGLRLSSGNLEKLTSGGINIGLGKNFVLHTEGLYRKSGDYAVPRYRN....LKRLPD
NEISp0944_9         SGELGLRLSSGNLEKLTSGGINIGLGKNFVLHTEGLYRKSGDYAVPRYRN....LKRLPD
```

```
                      240       250       260       270       280       290
                       .         .         .         .         .         .
NEISp0944_35     SHADSQTGSIGLSWVGEKGFIGAAYSDRRDRYGLPAHSHLYDDCHADIIWQKSLINKRYL
NEISp0944_39     SHADSQTGSIGLSWVGEKGFIGAAYSDRRDRYGLPAHSHLYDDCHADIIWQKSLINKRYL
NEISp0944_5      SHADSQTGSIGLSWVGEKGFIGAAYSDRRDQYGLPAHSHLYDDCHADIIWQKSLINKRYL
NEISp0944_75     SHADSQTGSIGLSWVGEKGFIGAAYSDRHDRYGLPAHSHLYDDCHADIIWQKSLINKRYL
NEISp0944_180    SHADSKTGSIGLSWVGEKGFLGVAYSDRRDQYGLPAHSHEYDGCDADIIWQSSLINKGYL
NEISp0944_22     SHADSKTGSIGLSWVGEKGFIGVAYSDRRDQYGLPAHSHEYDDCHADIIWQKSLINKRYL
NEISp0944_25     SHADSKTGTIGLSWVGEKGFIGVAYSDRRDQYGLPAHSHEYDDCHADIIWQKSLINKRYL
NEISp0944_142    SHADSKTGSIGLSWVGEKGFLGVAYSDRRDRYGLPAHSHLYDDCHADIIWQKSLINKRYL
NEISp0944_135    SHADSKTGSIGLSWVGDKGFLGIAYSDRRDRYGLPAHSHLYDDCHADIIWQKSLINKRYL
NEISp0944_126    SHADSKTGSIGLSWVGDKGFLGVAYSDRRDRYGLPAHSHLYDDCHADIIWQKSLINKRYL
NEISp0944_901    SHADSKTGSIGLSWVGEKGFLGVAYSDRRDRYGLPAHSHEYDDCHADIIWQKSLINKRYL
NEISp0944_131    SHADSKTGTIGLSWVGEKGFIGVAYSDRRDQYGLPAHSHEYDDCHADIIWQKSLINKGYL
NEISp0944_777    SHADSKTGTIGLSWVGEKGFIGVAYSDRRDQYGLPAHSHEYDDCHADIIWQKSLINKGYL
NEISp0944_207    SHADSKTGSIGLSWVGEKGFIGVAYSDRRDQYGLPAHSHEYDDCHADIIWQKSLINKGYL
NEISp0944_198    SRADSQTGSIGLSWVGEKGFIGVAYSDRRDQYGLPAHSHEYDDCHADIIWQKSLINKRYL
NEISp0944_863    SRADSQTGSIGLSWVGEKGFIGVAYSDRRDQYGLPAHSHEYDDCHADIIWQKSLINKRYL
NEISp0944_160    SRADSQTGSIGLSWVGEKGFIGVAYSDRRDQYGLPAHSHEYDDCHADIIWQKSLINKGYL
NEISp0944_859    SRADSQTGSIGLSWVGEKGFIGVAYSDRRDQYGLPAHSHEYDDCHADIIWQKSLINKRYL
NEISp0944_912    SHADSQTGSIGLSWVGEKGFIGVAYSDRRDQYGLPAHSHEYDDCHADIIWQKSLINKRYL
NEISp0944_251    SHADSQTGSIGLSWVGEKGFIGVAYSDRRDQYGLPAHSHEYDDCHADIIWQKSLINKRYL
NEISp0944_196    SHADSQTGSIGLSWVGEKGFIGAAYSDRRDQYGLPAHSHEYDDCHADIIWQKSLINKRYL
NEISp0944_263    SHADSQTGSIGLSWVGEKGFIGAAYSDRRDQYGLPAHSHEYDDCHADIIWQKSLINKRYL
NEISp0944_897    SRADSQTGSIGLSWVGEKGFIGVAYSDRRDQYGLPAHSHEYDDCHADIIWQKSLINKRYL
NEISp0944_79     SRADSQTGSIGLSWVGEKGFIGVSYSDRRDQYGLPAHSHEYDGCHADIIWQKSLINKGYL
NEISp0944_13     SRADSQTGSIGLSWVGEKGFIGAAYSDRRDQYGLPAHSHEYDDCHADIIWQKSLINKRYL
NEISp0944_574    SRADSQTGSIGLSWVGEKGFIGAAYSDRRDQYGLPAHSHEYDDCHADIIWQKSLINKGYL
NEISp0944_478    SHADSQTGSIGLSWVGEKGFIGAAYSDRRDQYGLPAHSHEYDGCHADIIWQKSLINKGYL
NEISp0944_347    SRADSQTGSIGLSWVGEKGFIGAAYSDRRDQYGLPAHSHEYDGCHADIIWQKSLINKGYL
NEISp0944_177    SRADSQTGSIGLSWVGEKGFIGAAYSDRRDQYGLPAHSHEYDGCHADIIWQKSLINKGYL
NEISp0944_140    SHADSQTGSIGLSWVGEKGFIGAAYSDRRDQYGLPAHSHEYDGCHADIIWQKSLINKGYL
NEISp0944_534    SHADSQTGSIGLSWVGEKGFIGAAYSDRRDQYSLPAHSHEYDGCHADIIWQKSLINKGYL
NEISp0944_37     SHADSKTGSIGLSWVGEKGFIGVAYSDRRDQYGLPAHSHEYDDCHADIIWQKSLINKRYL
NEISp0944_176    SRANSQTGSIGLSWVGEKGFIGAAYSDRRDQYGLPAHSHEYDDCHADIIWQKSLINKRYL
NEISp0944_907    SHADSKTGSIGLSWVGEKGFIGVAYSDRRDQYGLPAHSHEYDDCHADIIWQKSLINKRYL
NEISp0944_12     SHADSQTGSIGLSWVGEKGFIGAAYSDRRDRYGLPAHSHEYDDCHADIIWQKSLINKRYL
NEISp0944_69     SHADSKTGSIGLSWVGEKGFIGVAYSDRRDQYGLPAHSHEYDDCHADIIWQKSLINKGYL
NEISp0944_373    SRADSQTGSIVLSWVGEKGFIGVAYSDRRDQYGLPAHSHEYDDCHADIIWQKSLINKGYL
NEISp0944_86     SRADSQTGSIGLSWVGEKGFIGVAYSDRRDQYGLPAHSHEYDDCHADIIWQKSLINKRYL
NEISp0944_821    SRADSQTGSIGLSWVGEKGFIGVAYSDRRDQYGLPAHSHEYDDCHADIIWQKSLINKRYL
NEISp0944_369    SRADSQTGSIGLSWVGEKGFIGAAYSDRRDQYGLPAHSHEYDDCHADIIWQKSLISKRYL
NEISp0944_87     SRADSQTGSIGLSWVGEKGFIGVAYSDRRDQYGLPAHSHEYDDCHADIIWQKSLINKRYL
NEISp0944_88     SRADSQTGSIGLSWVGEKGFIGVAYSDRRDQYGLPAHSHEYDDCHADIIWQKSLINKRYL
NEISp0944_33     SHADSQTGSIGLSWVGEKGFIGVAYSDRRDQYGLPAHSHEYDDCHADIIWQKSLINKGYL
NEISp0944_70     SHADSKTGTIGLSWVGEKGFIGAAYSDRRDQYGLPAHSHEYDDCHADIIWQKSLINKRYL
NEISp0944_61     SRADSQTGSIGLSWVGEKGFIGVAYSDRRDQYGLPAHSHEYDDCHADIIWQKSLINKRYL
NEISp0944_83     SRADSQTGSIGLSWVGEKGFIGVAYSDRRDQYGLPAHSHEYDDCHADIIWQKSLISKRYL
NEISp0944_122    SRADSQTGSIGLSWVGEKGFIGVAYSDRRDQYGLPAHSHEYDDCHADIIWQKSLINKRYL
NEISp0944_778    SRADSQTGSIGLSWVGEKGFIGVAYSDRRDQYGLPAHSHEYDDCHADIIWQKSLINKRYL
NEISp0944_107    SRADSQTGSIGLSWVGEKGFIGVAYSDRRDQYGLPAHSHEYDDCHADIIWQKSLINKRYL
NEISp0944_56     SRADSQTGSIGLSWVGEKGFIGVAYSDRRDQYGLPAHSHEYDDCHADIIWQKSLINKRYL
NEISp0944_607    SRADSQTGSIGLSWVGEKGFIGVAYSDRRDQYGLPAHSHEYDDCHADIIWQKSLINKRYL
NEISp0944_318    SHADSQTGSIGLSWVGEKGFIGVVYSDRRDQYGLPAHSHEYDDCHADIIWQKSLINKRYL
NEISp0944_134    SHADSKTGSIGLSWVGEKGFIGVAYSDRRDQYGLPAHSHEYDDCHADIIWQKSLINKRYL
NEISp0944_129    SRADSQTGSIGLSWVGEKGFIGVAYSDRRDQYGLPAHSHEYDDCHADIIWQKSLINKRYL
NEISp0944_18     SHADSKTGTIGLSWVGEKGFIGVAYSDRRDQYGLPAHSHEYDDCHADIIWQKSLINKRYL
NEISp0944_1      SHADSQTGSIGLSWVGEKGFIGVVYSDRRDQYGLPAHSHEYDDCHADIIWQKSLINKRYL
NEISp0944_381    SHADSQTGSIGLSWVGEKGFIGVAYSDRRDQYGLPAHSHEYDDCHADIIWQKSLINKRYL
NEISp0944_157    SHADSQTGSIGLSWVGEKGFIGVAYSDRRDQYGLPAHSHEYDDCHADIIWQKSLINKRYL
NEISp0944_60     SHADSQTGSIGLSWVGEKGFIGVAYSDRRDQYGLPAHSHEYDDCHADIIWQKSLINKRYL
NEISp0944_606    SHADSQTGSIGLSWVGEKGFIGVAYSDRRDQYGLPAHSHEYDDCHADIIWQKSLINKRYL
NEISp0944_908    SHADSQTGSIGLSWVGEKGFIGAAYSDRRDQYGLPAHSHEYDDCHADIIWQKSLINKRYL
NEISp0944_130    SHADSQTGSIGLSWVGEKGFIGSAYSDRRDQYGLPAHSHEYDDCHADIIWQKSLINKRYL
NEISp0944_46     SHADSQTGSIGLSWVGEKGFIGAAYSDRRDQYGLPAHSHEYDDCHADIIWQKSLINKRYL
NEISp0944_23     SHADSQTGSIGLSWVGEKGFIGAAYSDRRDQYGLPAHSHEYDDCHADIIWQKSLINKRYL
NEISp0944_499    SHADSQTGSIELSWVGEKGFIGVAYSDRRDQYGLPAHSHEYDDCHADIIWQKSLINKRYL
NEISp0944_81     SHADSQTGSIGLSWVGEKGFIGAAYSDRRDQYGLPAHSHEYDDCHADIIWQKSLINKRYL
NEISp0944_41     SRADSQTGSIGLSWVGEKGFIGVAYSDRRDQYGLPAHSHEYDDCHADIIWQKSLINKRYL
NEISp0944_3      SHADSQTGSIGLSWVGEKGFIGAAYSDRRDQYGLPAHSHEYDDCHADIIWQKSLINKRYL
NEISp0944_553    SHADSQTGSIGLSWVGEKGFIGAAYSDRRDQYGLPAHSHEYDDCHADIIWQKSLINKRYL
NEISp0944_271    SRADSQTGSIGLSWVGEKGFIGVAYSDRRDQYGLPAHSHEYDDCHADIIWQKSLINKRYL
NEISp0944_82     SHADSKTGSIGLSWVGEKGFIGVAYSDRRDQYGLPAHSHEYDDCHADIIWQKSLINKGYL
NEISp0944_20     SHADSQTGSIGLSWVGEKGFIGAAYSDRRDQYGLPAHSHEYDDCHADIIWQKSLINKRYL
NEISp0944_2      SHADSQTGSIGLSWVGEKGFIGVAYSDRRDQYGLPAHSHEYDDCHADIIWQKSLINKRYL
NEISp0944_9      SHADSKTGSIGLSWVGEKGFIGAAYSDRRDQYGLPAHSHEYDDCHADIIWQKSLINKRYL
```

```
                      300       310       320       330       340       350
NEISp0944_35     QLYPHLLTEEDIDYDNPGLSCGFHDDDNAHAHAHSGRPWIDLRNKRYEIRAEWKQPLPGF
NEISp0944_39     QLYPHLLTEEDIDYDNPGLSCGFHDDDNAHAHTHSGRPWIDLRNKRYEIRAEWKQPLPGF
NEISp0944_5      QLYPHLLTEEDVDYDNPGLSCGFHDDDNAHAHTHSGRPWIDLRNKRYEIRAEWKQPLPGF
NEISp0944_75     QLYPHLLTEEDVDYDNPGLSCGFHDDDNAHAHTHNGRPWIDLRNKRYEIRAEWKQPLPGF
NEISp0944_180    RLYPHLLTEEDVDYDNPGLSCGSHDGDGAHAHTHSGRPWIDLRNKRYELRAEWKQPLPGF
NEISp0944_22     QLYPHLLTEEDVDYDNPGLSCGFHDDDNAHTHAHSGRPWIDLRNKRYELRAEWKQPLPGF
NEISp0944_25     QLYPHLLTEEDVDYDNPGLSCGFHDDDNAHAHTHSGRPWIDLRNKRYELRAEWKQPLPGF
NEISp0944_142    QLYPHLLTEEDVDYDNPGLSCGFHDDDNAHTHVHNGKPWIDLRNKRYELRAEWKQPFPGF
NEISp0944_135    QLYPHLLTEEDVDYDNPGLSCGFHDDDDVHAHAHNGKPWIDLRNKRYELRAEWKQPFPGF
NEISp0944_126    QLYPHLLTEEDVDYDNPGLSCGFHDDDNAHTHAHNGKPWIDLRNKRYELRAEWKQPFPGF
NEISp0944_901    QLYPHLLTEEDIDYDNPGLSCGFHDDDNAHAHAHNGKPWIDLRNKRYELRAEWKQPFPGF
NEISp0944_131    RLYPHLLTEEDIDYDNPGLSCGSHDGDGAHAHTHNGRPWIDLRNKRYELRAEWKQPLPGF
NEISp0944_777    RLYPHLLTEEDIDYDNPGLSCGSHDGDGAHAHTHNGRPWIDLRNKRYELRAEWKQPLPGF
NEISp0944_207    RLYPHLLTEEDIDYDNPGLSCGSHDGDGAHAHTHSGRPWIDLRNKRYELRAEWKQPLPGF
NEISp0944_198    QLYPHLLTEEDIDYDNPGLSCGFHDDDNAHAHTHSGRPWIDLHNKRYELRAEWKQPLPGF
NEISp0944_863    QLYPHLLTEEDIDYDNPGLSCGFHDDDNAHAHTHSGRPWIDLHNKRYELRAEWKQPLPGF
NEISp0944_160    RLYPHLLTEEDVDYDNPGLSCGFHDDDNAHAHTHSGRPWIDLHNKRYELRAEWKQPLPGF
NEISp0944_859    QLYPHLLTEEDVDYDNPGLSCGFHDDDNAHAHTHSGRPWIDLRNKRYELRAEWKQPLPGF
NEISp0944_912    QLYPHLLTEEDIDYDNPGLSCGFHDDDNAHAHTHNGRPWIDLRNKRYELRAEWKQPLPGF
NEISp0944_251    QLYPHLLTEEDIDYDNPGLSCGFHDDDNAHAHTHNGKPWIDLRNKRYELRAEWKQPLPGF
NEISp0944_196    QLYPHLLTEEDVDYDNPGLSCGFHDDDNAHAHTHSGRPWIDLRNKRYELRAEWKQPFPGF
NEISp0944_263    QLYPHLLTEEDVDYDNPGLSCGFHDDDNAHAHTHNGKPWIDLRNKRYELRAEWKQPFPGF
NEISp0944_897    QLYPHLLTEEDIDYDNPGLSCGFHDDDNAHAHAHNGRPWIDLRNKRYELRAEWKQPFAGF
NEISp0944_79     RLYPHLLTEEDVDYDNPGLSCGFHDDDNAHAHTHNGRPWIDLRNKRYELRAEWKQPLPGF
NEISp0944_13     QIYPHLLTEEDIDYDNPGLSCGFHDDDNAHAHTHSGRPWIDLRNKRYELRAEWKQPLPGF
NEISp0944_574    RLYPHLLTEEDVDYDNPGLSCGFHDDDNAHAHTHNGKPWIDLRNKRYELRAEWKQPLPGF
NEISp0944_478    RLYPHLLTEEDVDYDNPGLSCGFHDDDNAHAHTHSGRPWIDLRNKRYELRAEWKQPLPGF
NEISp0944_347    RLYPHLLTEEDVDYDNPGLSCGSHDGDGTHAHAHNGKPWIDLRNKRYELRAEWKHPLPGF
NEISp0944_177    RLYPHLLTEEDVDYDNPGLSCGFHDDDNAHAHTHNGKPWIDLRNKRYELRAEWKQPFPGF
NEISp0944_140    RLYPHLLTEEDVDYDNPGLSCGFHDDDNAHAHTHNGKPWIDLRNKRYELRAEWKQPLPGF
NEISp0944_534    RLYPHLLTEEDVDYDNPGLSCGFHDDDNAHAHTHNGKPWIDLRNKRYELRAEWKQPLPGF
NEISp0944_37     QLYPHLLTEEDIDYDNPGLSCGFHDDDNAHAHTHNGRPWIDLRNKRYELRAEWKQPFPGF
NEISp0944_176    QLYPHLLTEEDIDYDNPGLSCGFHDDDNAHAHTHNGRPWIDLRNKRYELRAEWKQPLPGF
NEISp0944_907    QLYPHLLTEEDIDYDNPGLSCGFHDDDNAHAHTHNGRPWIDLRNKRYELRAEWKQPLPGF
NEISp0944_12     QLYPHLLTEEDIDYDNPGLSCGFHDGDGAHAHTHNGKPWIDLRNKRYELRAEWKQPFPGF
NEISp0944_69     RLYPHLLTEEDIDYDNPGLSCGSHDGDGAHAHTHNGRPWIDLRNKRYELRAEWKQPLPGF
NEISp0944_373    RLYPHLLTEEDIDYDNPGLSCGSHDGDGAHAHTHNGRPWIDLRNKRYELRAEWKQPLPGF
NEISp0944_86     QLYPHLLTEEDIDYDNPGLSCGSHDGDGAHAHTHSGRPWIDLRNKRYELRAEWKQPFPGF
NEISp0944_821    QLYPHLLTEEDIDYDNPGLSCGFHDDDDAHAHTHNGRPWIDLRNKRYELRAEWKQPFPGF
NEISp0944_369    QFYPHLLTEEDVDYDNPGLSCGFHDDDNAHAHTHNGRPWIDLRNKRYELRAEWKQPLPGF
NEISp0944_87     QLYPHLLTEEDVDYDNPGLSCGFHDDDNAHAHTHSSRPWIDLRNKRYELRAEWKQPLPGF
NEISp0944_88     QLYPHLLTEEDVDYDNPGLSCGFHDDDNAHAHTHSSRPWIDLRNKRYELRAEWKQPLPGF
NEISp0944_33     RLYPHLLTEEDVDYDNPGLSCGFHDDDNAHAHTHSGRPWIDLRNKRYELRAEWKQPFPGF
NEISp0944_70     QLYPHLLTEEDVDYDNPGLSCGFHDDDNAHAHTHNGKPWIDLRNKRYELRAEWKQPLPGF
NEISp0944_61     QLYPHLLTEEDIDYDNPGLSCGFHDDDNAHAHTHSGRPWIDLRNKRYELRAEWKQPFPGF
NEISp0944_83     QLYPHLLTEEDIDYDNPGLSCGFHDDDNAHAHTHNGRPWIDLRNKRYELRAEWKQPLPGF
NEISp0944_122    QLYPHLLTEEDIDYDNPGLSCGFHDDDNAHAHTHSGRPWIDLRNKRYELRAEWKQPFPGF
NEISp0944_778    QLYPHLLTEEDIDYDNPGLSCGFHDDDNAHAHTHNGKPWIDLRNKRYELRAEWKQPLPGF
NEISp0944_107    QLYPHLLTEEDIDYDNPGLSCGFHDDDDAHAHTHNGRPWIDLRNKRYELRAEWKQPLPGF
NEISp0944_56     QLYPHLLTEEDVDYDNPGLSCGFHDDDNAHAHAHSGRPWIDLRNKRYELRAEWKQPLPGF
NEISp0944_607    QLYPHLLTEEDIDYDNPGLSCDFHDDDNAHAHAHNGKPWIDLHNKRYELRAEWKQPFPGF
NEISp0944_318    QLYPHLLTEEDVDYDNPGLSCGFHDDDDAHAHAHNGKPWIDLRNKRYELRAEWKQPFPGF
NEISp0944_134    QLYPHLLTEEDIDYDNPGLSCGFHDDDNAHAHTHNGRPWIDLRNKRYELRAEWKQPLPGF
NEISp0944_129    QLYPHLLTEEDIDYDNPGLSCGFHDDDNAHAHTHNGRPWIDLRNKRYELRAEWKQPLPGF
NEISp0944_18     QLYPHLLTEEDIDYDNPGLSCGFHDDDDAHAHAHNGKPWIDLRNKRYELRAEWKQPFPGF
NEISp0944_1      QLYPHLLTEEDIDYDNPGLSCGSHDGDGAHAHTHSGRPWIDLRNKRYELRAEWKQPLPGF
NEISp0944_381    QLYPHLLTEEDVDYDNPGLSCGFHDDDNAHAHAHNGKPWIDLRNKRYELRAEWKQPFPGF
NEISp0944_157    QLYPHLLTEEDIDYDNPGLSCGFHDDDDAHAHTHSGRPWIDLRNKRYELRAEWKQPFPGF
NEISp0944_60     QLYPHLLTEEDVDYDNPGLSCGFHDDDNAHAHTHNGKPWIDLRNKRYELRAEWKQPLPGF
NEISp0944_606    QLYPHLLTEEDIDYDNPGLSCGFHDDDNAHAHTHNGRPWIDLRNKRYELRAEWKQPFPGF
NEISp0944_908    QLYPHLLTEEDVDYDNPGLSCGFHDDDNAHAHTHNGRPWIDLRNKRYELRAEWKQPFPGF
NEISp0944_130    QLYPHLLTEEDIDYDNPGLSCGFHDDDNAHAHTHSGRPWIDLHNKRYELRAEWKQPLPGF
NEISp0944_46     QIYPHLLTEEDIDYDNPGLSCGFHDDDDAHAHAHSGRPWIDLHNKRYELRAEWKQPLPGF
NEISp0944_23     QLYPHLLTEEDIDYDNPGLSCGFHDDDNAHAHAHNGKPWIDLHNKRYELRAEWKHPLPGF
NEISp0944_499    QLYPHLLTEEDVDYDNPGLSCGFHDDDDAHAHAHNGKPWIDLRNKRYELRAEWKQPFPGF
NEISp0944_81     QLYPHLLTEEDVDYDNPGLSCGFHDDDNAHAHTHNGRPWIDLRNKRYELRAEWKQPFPGF
NEISp0944_41     QLYPHLLTEEDIDYDNPGLSCGFHDDDNAHAHTHSGRPWIDLRNKRYELRAEWKQPFPGF
NEISp0944_3      QLYPHLLTEEDIDYDNPGLSCGFHDDDDAHAHAHNGKPWIDLRNKRYELRAEWKQPFPGF
NEISp0944_553    QLYPHLLTEEDIDYDNPGLSCGFHDDDDAHAHAHNGRPWIDLRNKRYELRAEWKQPLPGF
NEISp0944_271    QLYPHLLTEEDIDYDNPGLSCGFHDDDDAHAHTHNGRPWIDLRNKRYELRAEWKQPFPGF
NEISp0944_82     RLYPHLLTEEDIDYDNPGLSCGFHDDDNAHAHTHSGRPWIDLHNKRYELRAEWKQPLPGF
NEISp0944_20     QLYPHLLTEEDIDYDNPGLSCGFHDDDDAHAHAHNGRPWIDLRNKRYELRAEWKHPLPGF
NEISp0944_2      QLYPHLLTEEDIDYDNPGLSCGFHDDDDAHAHTHSGRPWIDLRNKRYELRAEWKQPFPGF
NEISp0944_9      QLYPHLLTEEDVDYDNPGLSCGFHDDDNAHAHTHNGKPWIDLRNKRYELRAEWKQPLPGF
```

```
                          360       370       380       390       400       410
                            .         .         .         .         .         .
NEISp0944_35    EALRVHLNRNDYRHDEKAGDAVENFFKNKTQNARIELRHLPIGRLKGSWGVQYLTQKSSA
NEISp0944_39    EALRVHLNRNDYRHDEKAGDAVENFFKNKTQNARIELRHLPIGRLKGSWGVQYLTQKSSA
NEISp0944_5     EALRVHLNRNDYRHDEKAGDTVENFFKNKTQNARIELRHLPIGRLKGSWGVQYLTQKSSA
NEISp0944_75    EALRVHLNRNDYRHDEKAGDAVENFFKNKTQNARIELRHLPIGRLKGSWGVQYLTQKSSA
NEISp0944_180   EVLRVHLNRNDYHHDEKAGDAVENFFNNKTHNARIELRHQPIGRLKGSWGVQYLGQKSSA
NEISp0944_22    EALRVHLNRNDYHHDEKAGDAVENFFNNKTHNARIELRHQPIGRLKGSWGVQYLGQKSSA
NEISp0944_25    EALRVHLNRNDYQHDEKAGDAVENFFNNKTHNARIELRHQPIGRLKGSWGMQYLGQKSSA
NEISp0944_142   EALRVHLNRNDYHHDEKAGDAVENFFNNKTHNARIELRHQPIGRLKGSWGVQYLGQKSSA
NEISp0944_135   KALRVHLNRNDYHHDEKAGDAVENFFNNKTHNARIELRHQPIGRLKGSWGVQYLGQKSSA
NEISp0944_126   EALRVHLNHNDYHHDEKAGDAVENFFNNKTHNARIELRHQPIGRLKGSWGVQYLGQKSSA
NEISp0944_901   EALRVHLNRNDYHHDEKAGDAVENFFNNKTHNARIELRHQPIGRLKGSWGVQYLGQKSSA
NEISp0944_131   EALRVHLNRNDYHHDEKAGDAVENFFNNKTHNARIELRHQPIGRLKGSWGVQYLQQKSSA
NEISp0944_777   EALRVHLNRNDYHHDEKAGDAVENFFNNKTHNARIELRHQPIGRLKGSWGVQYLGQKSSA
NEISp0944_207   EALRVHLNRNNYHHDEKAGDAVENFFKNKTHNARIELRHQPIGRLKGSWGVQYLQQKSSA
NEISp0944_198   EALRVHLNRNDYRHDEKAGDAVENFFNNQTQNARIELRHQSIGRLKGSWGVQYLQQKSSA
NEISp0944_863   EALRVHLNRNDYRHDEKAGDAVENFFNNQTQNARIELRHQSIGRLKGSWGVQYLQQKSSA
NEISp0944_160   EALRVHLNRNDYRHDEKAGDAVENFFNNQTQNARIELRHQSIGRLKGSWGVQYLQQKSSA
NEISp0944_859   EALRVHLNRNNYHHDEKAGDAVENFFKNKTHNARIELRHQPIGRLKGSWGVQYLQQKSSA
NEISp0944_912   EALRVHLNRNDYHHDEKAGDAVENFFNNKTHNARIELRHQPIGRLKGSWGVQYLQQKSSA
NEISp0944_251   EALRVHLNRNDYRHDEKAGDAVENFFNNQTQNARIELRHQPIGRLKGSWGVQYLQQKSSA
NEISp0944_196   EALRVHLNRNDYHHDEKAGDAVENFFNNQTQNARIELRHQSIGRLKGSWGVQYLQQKSSA
NEISp0944_263   EALRAHLNRNDYHHDEKAGDAVENFFNNQTQNARIELRHQPIGRLKGSWGVQYLQQKSSA
NEISp0944_897   EALRVHLNRNDYRHDEKAGDAVENFFNNQTQNARIELRHQAVGRLKGSWGVQYLQQKSSA
NEISp0944_79    EALRVHLNRNDYRHDEKAGDAVENFFNNQTQNARIELRHQPIGRLKGSWGVQYLQQKSSA
NEISp0944_13    EALRVHLNRNDYRHDEKAGDAVENFFKNKTQNARIELRHQPIGRLKGSWGVQYLQQKSSA
NEISp0944_574   EALRVHLNRNDYRHDEKAGDAVENFFNNQTQNARIELRHQPIGRLKGSWGVQYLQQKSSA
NEISp0944_478   EALRVHLNRNDYRHDEKAGDAVENFFNNKTHNARIELRHQPIGRLKGSWGVQYLQQKSSA
NEISp0944_347   EALRVHLNRNDYRHDEKAGDAVENFFNNQTQNARIELRHQPIGRLKGSWGVQYLQQKSSA
NEISp0944_177   EALRVHLNRNDYRHDEKAGDAVENFFNNKTQNARIELRHQPIGRLKGSWGVQYLQQKSSA
NEISp0944_140   EALRVHLNRNDYRHDEKAGDAVENFFNNQTQNARIELRHHPIGRLKGSWGVQYLQQKSSA
NEISp0944_534   EALRVHLNRNDYRHDEKAGDAVENFFNNQTQNARIELRHQPIGRLKGSWGVQYLQQKSSA
NEISp0944_37    EALRVHLNRNDYRHDEKAGDAVENFFKNKTQNARIELRHQPIGRLKGSWGVQYLQQKSSA
NEISp0944_176   EALRVHLNRNDYRHDEKAGDAVENFFNNKTHNARIELRHQPIGRLKGSWGIQYLQQKSSA
NEISp0944_907   EALRVHLNRNDYRHDEKAGDAVENFFNNKTHNARIELRHQPIGRLKGSWGIQYLQQKSSA
NEISp0944_12    EALRVHLNRNDYHHDEKAGDAVENFFNNKTHNARIELRHQPIGRLKGSWGVQYLGQKSSA
NEISp0944_69    EALRVHLNRNDYHHDEKAGDAVENFFNNKTHNARIELRHQPIGRLKGSWGVQYLGQKSSA
NEISp0944_373   EALRVHLNRNDYHHDEKAGDAVENFFNNKTQNARIELRHQPIGRLKGSWGVQYLGQKSSA
NEISp0944_86    EALRVHLNRNDYHHDEKAGDAVENFFNNQTQNARIELRHQPIGRLKGSWGVQYLGQKSSA
NEISp0944_821   EALRVHLNRNDYHHDEKAGDAVENFFNNQTQNARIELRHQPIGRLKGSWGVQYLGQKSSA
NEISp0944_369   EALRVHLNRNDYHHDEKAGDAVENFFNNKTHNARIELRHQPIGRLKGSWGVQYLGQKSSA
NEISp0944_87    EALRVHLNRNDYHHDEKAGDAVENFFNNKTHNARIELRHQPIGRLKGSWGVQYLGQKSSA
NEISp0944_88    EALRVHLNRNDYHHDEKAGDAVENFFNNKTQNARIELRHQPIGRLKGSWGVQYLGQKSSA
NEISp0944_33    EALRVHLNRNDYHHDEKAGDTVENFFKNKTQNARIELRHQPIGRLKGSWGVQYLQQKSSA
NEISp0944_70    EALRVHLNRNDYHHDEKAGDAVENFFNNKTHNARIELRHQPIGRLKGSWGVQYLGQKSSA
NEISp0944_61    EALRVYLNRNDYRHDEKAGDAVENFFKNKTQNARIELRHQPIGRLKGSWGVQYLQQKSSA
NEISp0944_83    EALRVHLNRNDYRHDEKAGDAVENFFKNKTQNARIELRHQPIGRLKGSWGVQYLQQKSSA
NEISp0944_122   EALRVHLNRNDYRHDEKAGDAVENFFNNQTQNARIELRHQPIGRLKGSWGVQYLGQKSSA
NEISp0944_778   EALRVHLNRNDYRHDEKAGDAVENFFNNQTQNARIELRHQPIGRLKGSWGVQYLGQKSSA
NEISp0944_107   EALRVHLNRNDYHHDEKAGDAVENFFNNQTQNARIELRHQPIGRLKGSWGVQYLQQKSSA
NEISp0944_56    EALRVHLNRNDYHHDEKAGDTVENFFNNKTHNARIELRHQPIGRLKGSWGVQYLQQKSSA
NEISp0944_607   EALRVHLNRNDYRHDEKAGDAVENFFNNQTQNARIELRHQPIGRLKGSWGVQYLGQKSSA
NEISp0944_318   EALRVHLNRNDYHHDEKAGDAVENFFNNQTQNARIELRHQPIGRLKGSWGVQYLQQKSSA
NEISp0944_134   EALRVHLNRNDYHHDEKAGDAVENFFNNKTQNARIELRHQPIGRLKGSWGVQYLGQKSSA
NEISp0944_129   EALRVHLNRNDYRHDEKAGDAVENFFNNKTHNARIELRHQPIGRLKGSWGVQYLQQKSSA
NEISp0944_18    EALRVHLNRNDYHHDEKAGDAVENFFNNQTQNARIELRHQPIGRLKGSWGVQYLQQKSSA
NEISp0944_1     EALRVHLNRNDYHHDEKAGDAVENFFNNKTHNARIELRHQPIGRLKGSWGVQYLGQKSSA
NEISp0944_381   EALRVHLNRNDYHHDEKAGDAIENFFNNKTHNARIELRHQPIGRLKGSLGVQYLGQKSSA
NEISp0944_157   EALRVHLNRNDYHHDEKAGDAVENFFNNKTHNARIELRHQPIGRLKGSWGVQYLGQKSSA
NEISp0944_60    EALRVHLNRNDYHHDEKAGDAVENFFNNKTLNARIELRHQPIGRLKGSWGVQYLGQKSSA
NEISp0944_606   EALRVHLNRNDYHHDEKAGDAVENFFNNKTHNARIELRHQPIGRLKGSWGVQYLQQKSSA
NEISp0944_908   EALRVHLNRNDYRHDEKAGDAVENFFNNQTQNARIELRHQPIGRLRGSWGVQYLQQKSSA
NEISp0944_130   EALRVHLNRNDYRHDEKAGDAVENFFNNQTQNARIELRHQPIGRLKGSWGVQYLQQKSSA
NEISp0944_46    EALRVHLNRNDYRHDEKAGDAVENFFNNQTQNARIELRHQPIGRLKGSWGVQYLQQKSSA
NEISp0944_23    EALRVHLNRNDYRHDEKAGDAVENFFNNQTQNARIELRHQPIGRLKGSWGVQYLGQKSSA
NEISp0944_499   EALRVHLNRNDYHHDEKAGDAVENFFNNQTQNARIELRHQPIGRLKGSWGVQYLQQKSSA
NEISp0944_81    EALRVHLNRNDYRHDEKAGDAVENFFNNQTQNARIELRHQAVGRLKGSWGVQYLQQKSSA
NEISp0944_41    EALRVHLNRNDYRHDEKAGDAVENFFNNQTQNARIELRHQPIGRLKGSWGVQYLQQKSSA
NEISp0944_3     EALRVHLNRNDYHHDEKAGDAVENFFNNQTQNARIELRHQPIGRLKGSWGVQYLGQKSSA
NEISp0944_553   EALRVHLNRNDYHHDEKAGDAVENFFNNKTHNARIELRHQPIGRLKGSWGVQYLQQKSSA
NEISp0944_271   EALRVHLNRNDYHHDEKAGDTVENFFNNKTHNARIELRHQPIGRLKGSWGVQYLQQKSSA
NEISp0944_82    EALRVHLNRNDYHHDEKAGDAVENFFNNQTQNARIELRHQPIGRLKGSWGVQYLQQKSSA
NEISp0944_20    EALRVHLNRNDYRHDEKAGDAVENFFNNKTHNARIELRHQPIGRLKGSWGVQYLQQKSSA
NEISp0944_2     EALRVHLNRNDYRHDEKAGDAVENFFNNQTQNARIELRHQPIGRLKGSWGVQYLQQKSSA
NEISp0944_9     EALRVHLNRNDYHHDEKAGDAVENFFNNKTQNARIELRHQPIGRLKGSWGVQYLQQKSSA
```

```
                          420       430       440       450       460       470
NEISp0944_35        LSAIPETVQQPMLIDNDVHHYSFFGVEQANWDNFSLEGGVRVEKQKAAIQYDKALIDREN
NEISp0944_39        LSAIPETVQQPMLIDNDVHHYSFFGVEQANWDNFSLEGGVRVEKQKAAIQYDKALIDREN
NEISp0944_5         LSAIPETVQQPMLIDNDVHHYSFFGVEQANWDNFSLEGGVRVEKQKAAIQYNKALIDREN
NEISp0944_75        LSAIPETVQQPMLIDNDVHHYSFFGVEQANWDNFSLEGGVRVEKQKAAIQYDKELIDREN
NEISp0944_180       LSAIPETVQQPMLIDNNVRHYSFFGVEQANWDNFTLEGGVRVEKQKASIQYDKALIDREN
NEISp0944_22        LSAIPETVQQPMLIDNNVRHYSFFGVEQANWDNFTLEGGVRVEKQKASIQYDKALIDREN
NEISp0944_25        LSAIPETVQQPMLIDNNVRHYSFFGVEQANWDNFTLEGGVRVEKQKASIQYDKALIDREN
NEISp0944_142       LSAIPETVQQPMLIDNNVRHYSFFGVEQANWDNFTLEGGVRVEKQKASIQYDNALIDREN
NEISp0944_135       LSAIPETVQQPMLIDNNVRHYSFFGVEQANWDNFTLEGGVRVEKQKASIQYDKALIVREN
NEISp0944_126       LSAIPETVQQPMLIDNNVRHYSFFGVEQANWDNFTLEGGVRVEKQKASIQYDKALIDREN
NEISp0944_901       LSAIPETVQQPMLIDNNVRHYSFFGVEQANWDNFSLEGGVRVEKQKASIQYDKALIDREN
NEISp0944_131       LSATSKAIKQPMLLDNKVQHYGFFGVEQANWDNFTLEGGVRVEKQKASIQYDKALIDREN
NEISp0944_777       LSAIPETVQQPMLIDNNVRHYSFFGVEQANWDNFTLEGGVRVEKQKASIQYDKALIDREN
NEISp0944_207       LSATSEAVKQPMLLDNKVQHYSFFGVEQANWDNFTLEGGVRVEKQKASIQYDKALIDREN
NEISp0944_198       LSATSEAVKQPMLLDNNVQHYSFFGVEQAEWDNFTLEGGVRVEKQKASIRYDKALIDREN
NEISp0944_863       LSATSEAVKQPMLLDNNVQHYSFFGVEQANWDNFTLEGGVRVEKQKASIQYDKALIDREN
NEISp0944_160       LSATSEAVKQPMLLDNNVQHYSFFGVEQAEWDNFTLEGGVRVEKQKASIRYDKALIDREN
NEISp0944_859       LSATSEAVKQPMLLDNKVQHYSFFGVEQANWDNFTLEGGVRVEKQKASIQYDKALIDREN
NEISp0944_912       LSATSKAVKQPMLLDNKVQHYSFFVVEQANWDNFTLEGGVRVEKQKASIQYDKTLIDREN
NEISp0944_251       LSATSEAVKQPMLLDNKVQHYSFFGVEQANWDNFTLEGGVRVEKQKASIRYDKALIDREN
NEISp0944_196       LSATSEAVKQPMLLDNNVQHYSFFGVEQAEWDNFTLEGGVRVEKQKASIQYDKALIDREN
NEISp0944_263       LSATSEAVKQPMLLDNKVQHYSFFGVEQAEWDNFTLEGGVRVEKQKASIRYDKALIDREN
NEISp0944_897       LSATSEAVKQPMLIDNNVQHYSFFGVEQAEWDNFTLEGGVRVEKQKASIRYDKALINREN
NEISp0944_79        LSAISEAVKQPMLLDNKVQHYSFFGVEQANWDNFTLEGGVRVEKQKASIQYDKALIDREN
NEISp0944_13        LSATSEAVKQPMLLDNKVQHYSFFGVEQANWDNFTLEGGVRVEKQKASIRYDKALIDREN
NEISp0944_574       LSATSEAVKQPMLLDNKVQHYSFFGVEQANWDNFTLEGGVRVEKQKASIQYDKALIDREN
NEISp0944_478       LSATSEAVKQPMLLDNKVQHYSFFGVEQANWDNFTLEGGVRMEKQKASIQYDKALIDREN
NEISp0944_347       LSATSEAVKQPMLLDNKVQHYSFFGVEQANWDNFTLEGGVRVEKQKASIRYDKALIDREN
NEISp0944_177       LSATSEAVKQPMLLDNKVQHYSFFGVEQANWDNFTLEGGVRVEKQKASIRYDKALIDREN
NEISp0944_140       LSATSEAVKQPMLLDNKVQHYSFFGVEQANWDNFTLESGVRVEKQKASIRYDKALIDREN
NEISp0944_534       LSATSEAVKQPMLLDNKVQHYSFFGVEQANWDNFTLESGVRVEKQKASIRYDKALIDREN
NEISp0944_37        LSATSEAVKQPMLLDNKVQHYSFFGVEQANWDNFTLEGGVRVEKQKASIRYDKALIDREN
NEISp0944_176       LSATSEAVKQPMLLDNKVQHYSFFGVEQANWDNFTLEGGVRVEKQKASIRYDKALIDREN
NEISp0944_907       LSATSEAVKQPMLLDNKVQHYSFFGVEQANWDNFTLEGGVRVEKQKASIRYDKALIDREN
NEISp0944_12        LSAIPETVQQPMLIDNNVRHYSFFGVEQANWDNFTLEGGVRVEKQKASIRYDKALIDREN
NEISp0944_69        LSAIPETVQQPMLIDNNVRHYSFFGVEQANWDNFTLEGGVRVEKQKASIQYDKALIDREN
NEISp0944_373       LSAIPETVQQPMLIDNNVRHYSFFGVEQANWDNFTLEGGVRVEKQKASIQYDKALIDREN
NEISp0944_86        LSAIPETVQQPMLIDNNVRHYSFFGVEQAEWDNFTLEGGVRVEKQKASIQYDKALIDREN
NEISp0944_821       LSAIPETVQQPMLIDNNVRHYGFFGVEQANWDNFTLEGGVRVEKQKASIQYDKALINREN
NEISp0944_369       LSAIPETVQQPMLIDNNVRHYSFFGVEQANWDNFTLEGGVRVEKQKASIQYDKALIDREN
NEISp0944_87        LSAIPETVQQPMLIDNNVRHYSFFGVEQANWDNFTLEGGVRVEKQKASIQYDKALIDREN
NEISp0944_88        LSAIPETVQQPMLIDNNVRHYSFFGVEQANWDNFTLEGGVRVEKQKASIQYDKALIDREN
NEISp0944_33        LSATSEAVKQPMLLDNKVQHYGFFGVEQANWDNFTLEGGVRVEKQKASIQYDKALLDREN
NEISp0944_70        LSAIPETVQQPMLIDNNVRHYSFFGVEQANWDNFTLEGGVRVEKQKASIQYDKALIDREN
NEISp0944_61        LSATSEAVKQPMLIDNNVRHYSFFGVEQANWGNFTLEGGVRVEKQKASIRYDKALIDREN
NEISp0944_83        LSATSEAVKQPMLLDNKVQHYSFFGVEQANWDNFTLEGGVRVERQKASIRYDKALIDREN
NEISp0944_122       LSAIPETVKQPMLIDNNVRHYSFFGVEQANWDNFTLEGGVRVEKQKASIQYDKALIDREN
NEISp0944_778       LSAIPETVQQPMLIDNNVRHYSFFGVEQANWDNFTLEGGVRVEKQKASIQYDKALIDREN
NEISp0944_107       LSATSEAVKQPMLLDNKVQHYSFFGVEQANWDNFTLEGGVRVEKQKASIRYDKALIDREN
NEISp0944_56        LSATSEAVKQPMLLDNNVRHYSFFGVEQANWDNFTLEGGVRVEKQKASIQYDKALIDREN
NEISp0944_607       LSATSEAVKQPMLLDNKVQHYSFFGVEQANWDNFTLEGGVRVEKQKASIRYDKALIDREN
NEISp0944_318       LSATSEAVKQPMLIDNNVRHYSFFGVEQAEWDNFTLEGGVRVEKQKASIRYDKALIDREN
NEISp0944_134       LSAIPETVQQPMLIDNNVRHYSFFGVEQANWDNFTLEGGVRVEKQKASIQYDKALIDREN
NEISp0944_129       LSATSEAVKQPMLLDNKVQHYSFFGVEQANWDNFTLEGGVRVEKQKASIRYDKALIDREN
NEISp0944_18        LSATSEAVKQPMLLDNKVQHYSFFGVEQANWDNFTLEGGVRVEKQKASIRYDKALIDREN
NEISp0944_1         LSAIPETVQQPMLIDNNVRHYSFFGVEQANWDNFTLEGGVRVEKQKASIRYDKALIDREN
NEISp0944_381       LSAIPETVQQPMLIDNNVRHYSFFGVEQANWDNFTLEGGVRVEKQKASIRYDKALIDREN
NEISp0944_157       LSAIPETVQQPMLIDNNVRHYSFFGVEQANWDNFTLEGGVRVEKQKASIRYDKALIDREN
NEISp0944_60        LSAIPETVQQPMLIDNNVRHYSFFGVEQANWDNFTLEGGVRVEKQKASIQYDKALINREN
NEISp0944_606       LSATSEAVKQPMLIDNNVRHYSFFGVEQANWDNFTLEGGVRVEKQKASIQYDKALIDREN
NEISp0944_908       LSATSEAVKQPMLIDNNVRHYSFFGVEQANWDNFTLEGGVRVEKQKASIQYDKALIDREN
NEISp0944_130       LSATSEAVKQPMLLDNNVRHYSFFGVEQAEWDNFTLEGGVRVEKQKASIRYDKALIDREN
NEISp0944_46        LSATSEAVKQPMLIDNNVRHYSFFGVEQANWDNFTLEGGVRVEKQKASIRYDKALIDREN
NEISp0944_23        LSAIPETVQQPMLIDNNVRHYSFFGVEQANWDNFTLEGGVRVEKQKASIQYDKALIDREN
NEISp0944_499       LSATSEAVKQPMLIDNNVRHYSFFGVEQANWDNFTLEGGVRVEKQKASIQYDKALIDREN
NEISp0944_81        LSATSEAVKQPMLLDNKVQHYSFFGVEQANWDNFTLEGGVRVEKQKASIRYDKALIDREN
NEISp0944_41        LSATSEAVKQPMLLDNKVQHYSFFGVEQANWDNFTLEGGVRVEKQKASIRYDKALINREN
NEISp0944_3         LSAIPETVKQPMLIDNNVRHYSFFGVEQANWDNFTLEGGVRVEKQKASIQYDKALIDREN
NEISp0944_553       LSATSEAVKQPMLLDNKVQHYSFFGVEQANWDNFTLEGGVRVKKQKASIRYDKALIDREN
NEISp0944_271       LSATSEAVKQPMLLDNKVQHYSFFGVEQANWDNFTLEGGVRVEKQKASIRYDKALIDREN
NEISp0944_82        LSAISEAVKQPMLLDNKVQHYSFFGVEQANWDNFTLEGGVRVEKQKASIQYDKALIDREN
NEISp0944_20        LSATSEAVKQPMLLDNNVQHYSFFGVEQAEWDNFTLEGGVRVEKQKASIRYDKALIDREN
NEISp0944_2         LSAISEAVKQPMLLDNKVQHYSFFGVEQANWDNFTLEGGVRVEKQKASIRYDKALIDREN
NEISp0944_9         LSAISEAVKQPMLLDNKVQHYSFFGVEQAEWDNFTLEGGVRVEKQKASIQYDKALIDREN
```

```
                     480       490       500       510       520       530
NEISp0944_35     YYNQPLPDLGAHRQTARSFALSGNWYFTPQHKLSLTASHQERLPSTQELYAHGKHVATNT
NEISp0944_39     YYNQPLPDLGAHRQTARSFALSGNWYFTPHHKLSLTASHQERLPSTQELYAHGKHVATNT
NEISp0944_5      YYNQPLPDLGAHRQTARSFALSGNWYFSPQYKLSLTASHQERLPSTQELYAHGKHVATNT
NEISp0944_75     YYNQPLPDLGAHRQTARSFALSGNWYFSPYHKLSLTASHQERLPSTQELYAHGKHVATNT
NEISp0944_180    YYNYPLPDLGAHRQTARSFALSGNWYFTPQHKLSLTASHQERLPSTQELYAHGKHVATNT
NEISp0944_22     YYNQPLPDLGAHRQTARSFALSGNWYFTPQHKLSLTASHQERLPSTQELYAHGKHVATNT
NEISp0944_25     YYNQPLPDLGAHRQTARSFALSGNWYFTPHHKLSLTASHQERLPSTQELYAHGKHVATNT
NEISp0944_142    YYNQPLPDLGAHRQTARSFALSGNWYFTPQHKLSLTASHQERLPSTQELYAHGKHVATNT
NEISp0944_135    YYNQPLPDLGAHRQTARSFALSGNWYFTPQHKLSLTASHQERLPSTQELYAHGKHVATNT
NEISp0944_126    YYNQPLPDLGAHRQTARSFALSGNWYFTPQHKLSLTTSHQERLPSTQELYAHGKHVATNT
NEISp0944_901    YYNQPLPDLGAHRQTARSFALSGNWYFTPYHKLSLTASHQERLPSTQELYAHGKHVATNT
NEISp0944_131    YYNHPLPDLGAHRQTARSFALSGNWYFTPHHKLSLTASHQERLPSTQELYAHGKHVATNT
NEISp0944_777    YYNHPLPDLGAHRQTARSFALSGNWYFTPHHKLSLTASHQERLPSTQELYAHGKHVATNT
NEISp0944_207    YYNQPLPDLGAHRQTARSFALSGNWYFTPHHKLSLTASHQERLPSTQELYAHGKHVATNT
NEISp0944_198    YYKQPLPDLGAHRQTARSFALSGNWYFTPQHKLSLTASHQERLPSTQELYAHGKHVATNT
NEISp0944_863    YYNQPLPDLGAHRQTARSFALSGNWYFTPHHKLSLTASHQERLPSTQELYAHGKHVATNT
NEISp0944_160    YYKQPLPDLGAHRQTARSFALSGNWYFTPQHKLSLTASHQERLPSTQELYAHGKHVATNT
NEISp0944_859    YYNQPLPDLGAHRQTARSFALSGNWYFTPHHKLSLTASHQERLPSTQELYAHGKHVATNT
NEISp0944_912    YYNHPLPDLGAHRQTARSFALSGNWYFTPHHKLSLTASHQERLPSTQELYAHGKHVATNT
NEISp0944_251    YYNQPLPDLGAHRQTARSFALSGNWYFTPQHKLSLTASHQERLPSTQELYAHGKHVATNT
NEISp0944_196    YYNYPLPDLGAHRQTARSFALSGNWYFTPQHKLSLTASHQERLPSTQELYAHGKHVATNT
NEISp0944_263    YYKHPLPDLGAHRQTARSFALSGNWYFTPQHKLSLTTSHQERLPSTQELYAHGKHVATNT
NEISp0944_897    YYKQPLPDLGAHRQTARSFALSGNWYFTPRHKLSLTASHQERLPSTQELYAHGKHVATNT
NEISp0944_79     YYKHPLPDLGAHRQTARSFALSGNWYFTPQHKLSLTASHQERLPSTQELYAHGKHVATNT
NEISp0944_13     YYNYPLPDLGAHRQTARSFALSGNWYFTPHHKLSLTASHQERLPSTQELYAHGKHVATNT
NEISp0944_574    YYNHPLPDLGAHRQTARSFALSGNWYFTPQHKLSLTASHQERLPSTQELYAHGKHVATNT
NEISp0944_478    YYNHPLPDLGAHRQTARSFALSGNWYFTPQHKLSLTASHQERLPSTQELYAHGKHVATNT
NEISp0944_347    YYNHPLPDLGAHRQTARSFALSGNWYFTPQHKLSLTASHQERLPSTQELYAHGKHVATNT
NEISp0944_177    YYNQPLPDLGAHRQTARSFALSGNWYFTPQHKLSLTASHQERLPSTQELYAHGKHVATNT
NEISp0944_140    HYNYPLPDLGAHRQTARSFALSGNWYFTPHHKLSLTASHQERLPSTQELYAHGKHVATNT
NEISp0944_534    HYNYPLPDLGAHRQTARSFALSGNWYFTPYHKLSLTASHQERLPSTQELYAHGKHVATNT
NEISp0944_37     YYNHPLPDLGAHRQTARSFALSGNWYFTPQHKLSLTASHQERLPSTQELYAHGKHVATNT
NEISp0944_176    YYNQPLPDLGAHRQTARSFALSGNWYFTPQHKLSLTASHQERLPSTQELYAHGKHVATNT
NEISp0944_907    YYNQPLPDLGAHRQTARSFALSGNWYFTPQHKLSLTASHQERLPSTQELYAHGKHVATNT
NEISp0944_12     YYNQPLPDLGAHRQTARSFALSGNWYFTPHHKLSLTASHQERLPSTQELYAHGKHVATNT
NEISp0944_69     YYNHPLPDLGAHRQTARSFALSGNWYFTPHHKLSLTASHQERLPSTQELYAHGKHVATNT
NEISp0944_373    YYNHPLPDLGAHRQTARSFALSGNWYFTPQHKLSLTASHQERLPSTQELYAHGKHVATNT
NEISp0944_86     YYNHPLPDLGAHRQTARSFALSGNWYFTPQHKLSLTASHQERLPSTQELYAHGKHVATNT
NEISp0944_821    YYNQPLPDLGAHRQTARSFALSGNWYFTPHHKLSLTASHQERLPSTQELYAHGKHVATNT
NEISp0944_369    YYNQPLPDLDAHRQTARSFALSGNWYFTPQHKLSLTASHQERLPSTQELYAHGKHVATNT
NEISp0944_87     YYNHPLPDLGAHRQTARSFALSGNWYFTPQHKLSLTASHQERLPSTQELYAHGKHVATNT
NEISp0944_88     YYNQPLPDLGAHRQTARSFALSGNWYFTPQHKLSLTASHQERLPSTQELYAHGKHVATNT
NEISp0944_33     YYNYPLPDLGAHRQTARSFALSGNWYFTPQHKLSLTASHQERLPSTQELYAHGKHVATNT
NEISp0944_70     YYNQPLPDLGAHHQTARSFALSGNWYFSPQHKLSLTASHQERLPSTQELYAHGKHVATNT
NEISp0944_61     YYNHPLPDLGAHRQTARSFALSGNWYFTPQHKLSLTASHQERLPSTQELYAHGKHVATNT
NEISp0944_83     YYKQPLPDLGAHRQTARSFALSGNWYFTPQHKLSLTASHQERLPSTQELYAHGKHVATNT
NEISp0944_122    YYNQPLPDLGAHRQAARSFALSGNWYFTPQHKLSLTASHQERLPSTQELYAHGKHVATNT
NEISp0944_778    YYNHPLPDLGAHRQTARSFALSGNWYFTPHHKLSLTASHQERLPSTQELYAHGKHVATNT
NEISp0944_107    YYNHPLPDLGAHRQTARSFALSGNWYFSPYHKLSLTASHQERLPSTQELYAHGKHVATNT
NEISp0944_56     YYNQPLPDLGAHRQTARSFALSSNWYFTPQHKLSLTASHQERLPSTQELYAHGKHVATNT
NEISp0944_607    YYNHPLPDLGAHRQTARSFALSGNWYFTPQHKLSLTASHQERLPSTQELYAHGKHVATNT
NEISp0944_318    YYKQPLPDLGAHRQTARSFALSGNWYFTPQHKLSLTASHQERLPSTQELYAHGKHVATNT
NEISp0944_134    YYNQPLPDLGAHRQTARSFALSGNWYFTPQHKLSLTASHQERLPSTQELYAHGKHVATNT
NEISp0944_129    YYNQPLPDLGAHRQTARSFALSGNWYFTPQHKLSLTASHQERLLSTQELYAHGKHVATNT
NEISp0944_18     YYNHPLPDLGAHRQTARSFALSGNWYFTPQHKLSLTASHQERLPSTQELYAHGKHVATNT
NEISp0944_1      YYNQPLPDLGAHRQTARSFALSGNWYFTPHHKLSLTASHQERLPSTQELYAHGKHVATNT
NEISp0944_381    YYNQPLPDLGAHRQTARSFALSGNWYFTPQHKLSLTASHQERLPSTQELYAHGKHVATNT
NEISp0944_157    YYNQPLPDLGAHRQTARSFALSGNWYFTPHHKLSLTASHQERLPSTQELYAHGKHVATNT
NEISp0944_60     YYNQPLPDLGAHRQTARSFALSGNWYFTPQHKLSLTASHQERLPSTQELYAHGKHVATNT
NEISp0944_606    YYNQPLPDLGAHRQTARSFALSGNWYFTPQHKLSLTASHQERLPSTQELYAHGKHVATNT
NEISp0944_908    YYNQPLPDLGAHRQTARSFALSGNWYFTPQHKLSLTASHQERLPSTQELYAHGKHVATNT
NEISp0944_130    YYNHPLPDLGAHRQTARSFALSGNWYFTPQHKLSLTASHQERLPSTQELYAHGKHVATNT
NEISp0944_46     YYNHPLPDLGAHRQTARSFALSGNWYFTPQHKLSLTASHQERLPSTQELYAHGKHVATNT
NEISp0944_23     YYNQPLPDLGAHRQTARSFALSGNWYFTPQHKLSLTASHQERLPSTQELYAHGKHVATNT
NEISp0944_499    YYNHPLPDLGAHRQTARSFALSGNWYFTPQHKLSLTASHQERLPSTQELYAHGKHVATNT
NEISp0944_81     YYKQPLPDLGAHRQTARSFALSGNWYFTPQHKLSLTASHQERLPSTQELYAHGKHVATNT
NEISp0944_41     YYNQPLPDLGAHRQTARSFALSGNWYFTPQHKLSLTASHQERLPSTQELYAHGKHVATNT
NEISp0944_3      YYNQPLPDLGAHRQTARSFALSGNWYFTPQHKLSLTASHQERLPSTQELYAHGKHVATNT
NEISp0944_553    YYKHPLPDLGAHRQTARSFALSGNWYFTPQHKLSLTASHQERLPSTQELYAHGKHVATNT
NEISp0944_271    YYKHPLPDLGAHRQTARSFALSGNWYFTPHHKLSLTASHQERLPSTQELYAHGKHVATNT
NEISp0944_82     YYNHPLPDLGAHRQTARSFALSGNWYFTPQHKLSLTASHQERLPSTQELYAHGKHVATNT
NEISp0944_20     YYNQPLPDLGAHRQTARSFALSGNWYFTPQHKLSLTASHQERLPSTQELYAHGKHVATNT
NEISp0944_2      YYNHPLPDLGAHRQTARSFALSGNWYFTPQHKLSLTASHQERLPSTQELYAHGKHVATNT
NEISp0944_9      YYKQPLPDLGAHRQTARSFALSGNWYFTPQHKLSLTASHQERLPSTQELYAHGKHVATNT
```

```
                       540       550       560       570       580       590
NEISp0944_35   FEVGNKHLNKERSNNIELALGYEGDRWQYNLALYRNRFGNYIYAQTLNDGRGPKSIEDDS
NEISp0944_39   FEVGNKHLNKERSNNIELALGYEGDRWQYNLALYRNRFGNYIYAQTLNDGRGPKSIEDDS
NEISp0944_5    FEVGNKHLNKERSNNIELALGYEGDRWQYNLALYRNRFGNYIYAQTLNDGRGPKSIEDDS
NEISp0944_75   FEVGNKHLNKERSNNIELALGYEGDRWQYNLALYRNRFGNYIYAQTLNDGRGPKSIEDDS
NEISp0944_180  FEVGNKHLNKERSNNIELALGYEGDRWQYNLALYRNRFGNYIYAQTLNDGRGFKSIEDDS
NEISp0944_22   FEVGNKHLNKERSNNIELALGYEGDRWQYNLALYRNRFGNYIYAQTLNDGRGPKSIENDS
NEISp0944_25   FEVGNKHLNKERSNNIELALGYEGDRWQYNLALYRNRFGNYIYAQTLNDGRGPKSIEDDS
NEISp0944_142  FEVGNKHLNKERSNNIELALGYEGDRWQYNLALYRNRFGNYIYAQTLNDGRGFKSIEDDS
NEISp0944_135  FEVGNKHLNKERSNNIELALGYEGDRWQYNLALYRNRFDNYIYAQTLNDGRGPKSIENDS
NEISp0944_126  FEVGNKHLNKERSNNIELALGYEGDRWQYNLALYRNRFGNYIYAQTLNDGRGPKSIEDDS
NEISp0944_901  FEVGNKHLNKERSNNIELALGYEGDRWQYNLALYRNRFGNYIYAQTLNDGRGPKSIEDDS
NEISp0944_131  FEVGNKHLNKERSNNIEIALGYEGDRWQYNLALYHNRFGNYIYAQTLNDGRGPKSIEDDS
NEISp0944_777  FEVGNKYLNKERSNNIEIALGYEGDRWQYNLALYRNRFGNYIYAQTLNDGRGPKSIEDDS
NEISp0944_207  FEVGNKHLNKERSNNIELALGYEGDRWQYNLALYRNRFGNYIYAQTLNDGRGPKSIEDDS
NEISp0944_198  FEVGNKYLNKERSNNIEIALGYEGDRWQYNLALYRNRFGNYIYAQTLNDGRGPKSIENDS
NEISp0944_863  FEVGNKHLNKERSNNIELALGYEGDRWQYNLAVYRNRFGNYIYAQTLNDGRGPKSIEDDS
NEISp0944_160  FEVGNKHLNKERSNNIELALGYEGDRWQYNLALYRNRFGNYIYAQTLNDGRGPKSIEDDS
NEISp0944_859  FEVGNKHLNKERSNNIELALGYEGDRWQYNLALYRNRFGNYIYAQTLNDGRGPKSIEDDS
NEISp0944_912  FEVGNKHLNKERSNNIELALGYEGDRWQYNLALYRNRFGNYIYAQTLNDGRGRKSIENDS
NEISp0944_251  FEVGNKHLNKERSNNIELALGYEGDRWQYNLALYRNRFGNYIYAQTLNDGRGPKSIEDDS
NEISp0944_196  FEVGNKHLNKERSNNIELALGYEGDRWQYNLALYRNRFGNYIYAQTLNDGRGPKSIEDDS
NEISp0944_263  FEVGNKHLNKERSNNIELALGYEGDRWQYNLALYRNRFGNYIYAQTLNDGRGPKSIEDDS
NEISp0944_897  FEVGNKHLNKERSNNIELALGYEGDRWQYNLALYRNLFGNYIYAQTLNDGRGPKSIEDDS
NEISp0944_79   FEVGNKHLNKERSNNIELALGYEGDRWQYNLALYRNRFGNYIYAQTLNDGRGPKSIEDDS
NEISp0944_13   FEVGNKHLNKERSNNIELALGYEGDRWQYNLALYRNRFGNYIYAQTLNDGRGPKSIEDDS
NEISp0944_574  FEVGNKHLNKERSNNIELALGYEGDRWQYNLALYRNRFGNYIYAQTLNDGRGPKSIEDDS
NEISp0944_478  FEIGNKHLKKERSNNIELALGYEGDRWQYNLALYRNRFGNYIYAQTLNDGRGPKSIEDDS
NEISp0944_347  FEVGNKHLNKERSNNIELALGYEGDRWQYNLALYRNRFGNYIYAQTLNDGRGPKSIEDDS
NEISp0944_177  FEVGNKHLNKERSNNIELALGYEGDRWQYNLALYRNRFGNYIYAQTLNDGRGPKSIEDDS
NEISp0944_140  FEVGNKHLNKERSNNIELALGYEGDRWQYNLALYRNRFGNYIYAQTLNDGRGPKSIEDDS
NEISp0944_534  FEVGNKHLNKERSNNIELALGYEGDRWQYNLALYRNRFGNYIYAQTLNDGRGPKSIEDDS
NEISp0944_37   FEVGNKHLNKERSNNIELALGYEGDRWQYNLALYRNRFGNYIYAQTLNDGRGPKSIEDDS
NEISp0944_176  FEVGNKHLNKERSNNIELALGYEGDRWQYNLALYRNRFGNYIYAQTLNDGRGPKSIEDDS
NEISp0944_907  FEVGNKHLNKERSNNIELALGYEGDRWQYNLALYRNRFGNYIYAQTLNDGRGPKSIEDDS
NEISp0944_12   FEVGNKHLNKERSNNIELALGYEGDRWQYNLAAYRNRFGNYIYAQTLNDGRGPKSIEDDS
NEISp0944_69   FEVGNKHLNKERSNNIELALGYEGDHWQYNLALYRNRFGNYIYAQTLNDGRGPKSIEDDS
NEISp0944_373  FEVGNKYLNKERSNNIEIALGYEGDRWQYNLALYRNRFGNYIYAQTLNDGRGPKSIENDS
NEISp0944_86   FEVGNKHLNKERSNNIELALGYEGDRWQYNLALYRNRFGNYIYAQTLNDGRGPKSIEDDS
NEISp0944_821  FEVGNKHLNKERSNNIELALGYEGDRWQYNLALYRNRFGNYIYAQTLNDGRGPKSIENDS
NEISp0944_369  FEVGNKHLNKERSNNIELALGYEGDRWQYNLALYRNRFGNYIYAQTLNDGRGPKSIEDDS
NEISp0944_87   FEVGNKHLNKERSNNIELALGYEGDRWQYNLALYRNRFGNYIYAQTLNDGRGPKSIEDDS
NEISp0944_88   FEVGNKHLNKERSNNIELALGYEGDRWQYNLALYRNRFGNYIYAQTLNDGRGPKSIEDDS
NEISp0944_33   FEVGNKHLNKERSNNIELALGYEGDRWQYNLALYRNRFGNYIYAQTLNDGRGPKSIEDDS
NEISp0944_70   FEVGNKHLNKERSNNIELALGYEGDRWQYNLALYRNRFGNYIYAQTLNDGRGPKSIENDS
NEISp0944_61   FEVGNKHLNKERSNNIELALGYEGDRWQYNLALYRNRFGNYIYAQTLNDGRGPKSIEDDS
NEISp0944_83   FEVGNKHLNKERSNNIEIALGYEGDRWQYNLALYRNRFGNYIYAQTLNDGRGPKSIEDDS
NEISp0944_122  FEVGNKHLNKERSNNIEIALGYEGDRWQYNLALYRNRFGNYIYAQTLNDGRGPKSIEDDS
NEISp0944_778  FEVGNKHLNKERSNNIELALGYEGDHWQYNLALYRNRFGNYIYAQTLNDGRGPKSIEDDS
NEISp0944_107  FEVGNKHLKKERSNNIEIALGYEGDHWQYNLALYRNRFGNYIYAQTLNDGRGPKSIEDDS
NEISp0944_56   FEVGNKHLNKERSNNIELALGYEGDRWQYNLALYRNRFGNYIYAQTLNDGRGPKSIENDS
NEISp0944_607  FEVGNKHLNKERSNNIELALGYEGDRWQYNLALYRNRFGNYIYAQTLNDGRGPKSIEDDS
NEISp0944_318  FEVGNKHLNKERSNNIELALGYEGDRWQYNLALYRNRFGNYIYAQTLNDGRGPKSIEDDS
NEISp0944_134  FEVGNKHLNKERSNNIELALGYEGDRWQYNLALYRNRFGNYIYAQTLNDGRGPKSIEDDS
NEISp0944_129  FEVGNKHLNKERSNNIELALGYEGDRWQYNLALYRNRFGNYIYAQTLNDGRGPKSIEDDS
NEISp0944_18   FEVGNKHLNKERSNNIELALGYEGDRWQYNLALYRNRFGNYIYAQTLNDGRGPKSIEDDS
NEISp0944_1    FEVGNKHLNKERSNNIELALGYEGDRWQYNLALYRNHFGNYIYAQTLNDGRGPKSIEDDS
NEISp0944_381  FEVGNKHLNKERSNNIELALGYEGDRWQYNLAVYRNRFGNYIYAQTLNDGRGPKSIENDS
NEISp0944_157  FEVGNKHLNKERSNNIELALGYEGDRWQYNLALYRNHFGNYIYAQTLNDGRGPKSIEDDS
NEISp0944_60   FEVGNKHLNKERSNNIELALGYEGDRWQYNLALYRNRFGNYIYAQTLNDGRGPKSIEDDS
NEISp0944_606  FEVGNKHLNKERSNNIELALGYEGDRWQYNLALYRNRFGNYIYAQTLNDGRGPKSIEDDS
NEISp0944_908  FEVGNKHLNKERSNNIELALGYEGDRWQYNLALYRNRFGNYIYAQTLNDGRGPKSIEDDS
NEISp0944_130  FEVGNKHLNKERSNNIELALGYEGDRWQYNLALYRNRFGNYIYAQTLNDGRGPKSIEDDS
NEISp0944_46   FEVGNKHLNKERSNNIELALGYEGDRWQYNLALYRNRFGNYIYAQTLNDGRGPKSIEDDS
NEISp0944_23   FEVGNKYLNKERSNNIELALGYEGDHWQYNLALYRNRFGNYIYAQTLNDGRGPKSIEDDS
NEISp0944_499  FEVGNKHLNKERSNNIELALGYEGDRWQYNLALYRNRFGNYIYAQTLNDGRGPKSIEDDS
NEISp0944_81   FEVGNKYLNKERSNNIEIALGYEGDRWQYNLALYRNRFGNYIYAQTLNDGRGPKSIENDS
NEISp0944_41   FEVGNKHLNKERSNNIELALGYEGDRWQYNLALYRNRFGNYIYAQTLNDGRGPKSIEDDS
NEISp0944_3    FEVGNKHLNKERSNNIELALGYEGDRWQYNLALYRNRFGNYIYAQTLNDGRGPKSIEDDS
NEISp0944_553  FEIGNKHLKKERSNNIELALGYEGDRWQYNLALYRNRFGNYIYAQTLNDGRGPKSIEDDS
NEISp0944_271  FEVGNKHLNKERSNNIELALSYEGDRWQYNLALYRNRFGNYIYAQTLNDGRGPKSIEDDS
NEISp0944_82   FEVGNKHLNKERSNNIELALGYEGDRWQYNLALYRNRFGNYIYAQTLNDGRGPKSIEDDS
NEISp0944_20   FEVGNKHLNKERSNNIELALGYEGDRWQYNLALYRNRFGNYIYAQTLNDGRGPKSIEDDS
NEISp0944_2    FEVGNKHLNKERSNNIELALGYEGDRWQYNLALYRNRFGNYIYAQTLNDGRGPKSIEDDS
NEISp0944_9    FEVGNKHLNKERSNNIELALGYEGDRWQYNLALYRNRFGNYIYAQTLNDGRGPKSIEDDS
```

```
                        600       610       620       630       640       650
NEISp0944_35      EMKLVRYNQSGADFYGAEGEIYFKPTPRYRIGVSGDYVRGRLKNLPSLPGREDAYGNRPF
NEISp0944_39      EMKLVRYNQSGADFYGAEGEIYFKPTPRYRIGLSGDYVRGRLKNLPSLPGREDAYGNRPY
NEISp0944_5       EMKLVRYNQSGADFYGAEGEIFFKPTPRYRIGLSGDYVRGRLKNLPSLPGREDAYGNRPL
NEISp0944_75      EMKLVRYNQSGADFYGAEGEIYFKPTPRYRIGLSGDYVRGRLKNLPSLPGREDAYGNRPL
NEISp0944_180     EMKLVRYNQSGADFYGAEGEIYFKPTPRYRIGVSGDYVRGRLKNLPSLPGRGDAYGNRPF
NEISp0944_22      EMKLVRYNQSGADFYGAEGEIYFKPTPRYRIGVSGDYVRGRLKNLPSLPGREDAYGNRPF
NEISp0944_25      EMKLVRYNQSGADFYGAEGEIYFKPTPRYRIGVSGDYVRGRLKNLPSLPGRGDGNDKRPF
NEISp0944_142     EMKLVRYNQSGADFYGAEGEIYFKPTPRYRIGVSGDYVRGRLKNLPSLPGRGDAYGNRPF
NEISp0944_135     EMKLVRYNQSGADFYGAEGEIYFKPTPRYRIGVSGDYVRGRLKNLPSLPGREDAYGNRPF
NEISp0944_126     EMKLVRYNQSGADFYGAEGEIYFKPTPRYRIGVSGDYVRGRLKNLPSLPGREDAYGNRPF
NEISp0944_901     EMKLVRYNQSGADFYGAEGELYFKPTPRYRIGVSGDYVRGRLKNLPSLPGREDAYGNRPF
NEISp0944_131     EMKLVRYNQSGADFYGAEGEIYFKPTPRYRIGVSGDYVRGRLKNLPSLPGREDAYGNRPF
NEISp0944_777     EMKLVRYNQSGSDFYGAEGEIYFKPTPRYRIGVSGDYVRGRLKNLPSLPGREDAYGKRPF
NEISp0944_207     EMKLVRYNQSGADFYGAEGEIYFKPTPRYRIGVSGDYVRGRLKNLPSLPGREDAYGKRPF
NEISp0944_198     EMKLVRYNQSGADFYGAEGEIYFKPTPRYRIGVSGDYVRGRLKNLPSLPGREDAYGNRPF
NEISp0944_863     EMKLVRYNQSGADFYGAEGEIYFKPTPRYRIGVSGDYVRGRLKNLPSLPGREDAYGKRPF
NEISp0944_160     EMKLVRYNQSGADFYGAEGEIYFKPTPRYRIGVSGDYVRGRLKNLPSLPGREDAYGNRPF
NEISp0944_859     EMKLVRYNQSGADFYGAEGEIYFKPTPRYRIGVSGDYVRGRLKNLPSLPGREDAYGKRPF
NEISp0944_912     EMKLVRYNQSGADFYGAEGEIYFKPTPRYRIGVSGDYVRGRLKNLPSLPGREDAYGNRPF
NEISp0944_251     EMKLVRYNQSGADFYGAEGEIYFKPTPRYRIGVSGDYVRGRLKNLPSLPGREDAYGKRPF
NEISp0944_196     EMKLVRYNQSGADFYGAEGEIYFKPTPRYRIGVSGDYVRGRLKNLPSLPGREDAYGNRPF
NEISp0944_263     EMKLVRYNQSGADFYGAEGEIYFKPTPRYRIGVSGDYVQGRLKNLPSLPGREDAYGKRPF
NEISp0944_897     EMKLVRYNQSGADFYGAEGEIYFKPTPRYRIGVSGDYVRGRLKNLPSLPGRGDGNDKRPF
NEISp0944_79      EMKLVRYNQSGADFYGAEGEIYFKPTPRYRIGVSGDYVRGRLKNLPSLPGREDAYGNRPF
NEISp0944_13      EMKLVRYNQSGADFYGAEGEIYFKPTPRYRIGVSGDYVRGRLKNLPSLPGRGDGNDKRPL
NEISp0944_574     EMKLVRYNQSGADFYGAEGEIYFKPTPRYRIGVSGDYVRGRLKNLPSLPGRGDGNDKRPF
NEISp0944_478     EMKLVRYNQSGADFYGAEGEIYFKPTPRYRIGVSGDYVRGRLKNLPSLPGREDAYGNRPF
NEISp0944_347     EMKLVRYNQSGADFYGAEGEIYFKPTPRYRIGVSGDYVRGRLKNLPSLPGREDAYGKRPF
NEISp0944_177     EMKLVRYNQSGADFYGAEGEIYFKPTPRYRIGVSGDYVRGRLKNLPSLPGREDAYGNRPF
NEISp0944_140     EMKLVRYNQSGADFYGAEGEIYFKPTPRYRIGVSGDYVRGRLKNLPSLPGRGDGNDKRPL
NEISp0944_534     EMKLVRYNQSGADFYGAEGEIYFKPTPRYRIGVSGDYVRGRLKNLPSLPGREDAYGNRPF
NEISp0944_37      EMKLVRYNQSGADFYGAEGEIYFKPTPRYRIGVSGDYVRGRLKNLPSLPGRGDGNDKRPF
NEISp0944_176     EMKLVRYNQSGADFYGAEGEIYFKPTPHYRIGVSGDYVRGRLKNLPSLPGRGDGNDKRPF
NEISp0944_907     EMKLVRYNQSGADFYGAEGEIYFKPTPRYRIGISGDYVRGRLKNLPSLPGREDAYGKRPF
NEISp0944_12      EMKLVRYNQSGADFYGAEGEIYFKPTPRYRIGVSGDYVRGRLKNLPSLPGREDPYGKRPF
NEISp0944_69      EMKLVRYNQSGADFYGAEGEIYFKPTPRYRIGVSGDYVRGRLKNLPSLPGREDAYGNRPF
NEISp0944_373     EMKLVRYNQSGADFYGAEGEIYFKPTPRYRIGVSGDYVRGRLKNLPSLPGREDAYGNRPF
NEISp0944_86      EMKLVRYNQSGADFYGTEGEIYFKPTPRYRIGVSGDYVRGRLKNLPSLPGREDAYGNRPF
NEISp0944_821     EMKLVRYNQSGADFYGAEGEIYFKPTPRYRIGVSGDYVRGRLKNLPSLPGREDAYGNRPF
NEISp0944_369     EMKLVRYNQSGADFYGAEGEIYFKPTPRYRIGVSGDYVRGRLKNLPSLPGREDAYGKRPF
NEISp0944_87      EMKLVRYNQSGADFYGAEGEIYFKPTPRYRIGVSGDYVRGRLKNLPSLPGRGDGNDKRPL
NEISp0944_88      EMKLVRYNQSGADFYGAEGEIYFKLTPRYRIGVSGDYVRGRLKNLPSLPGREDAYGNRPF
NEISp0944_33      EMKLVRYNQSSADFYGAEGEIYFKPTPRYRIGVSGDYVRGRLKNLPSLPGREDAYGNRPF
NEISp0944_70      EMKLVRYNQSGADFYGAEGEIYFKPTPRYRIGVSGDYVRGRLKNLPSLPGREDAYGNRPF
NEISp0944_61      EMKLVRYNQSGADFYGAEGEIYFKPTPRYRIGVSGDYVRGRLKNLPSLPGREDAYGKRPF
NEISp0944_83      EMKLVRYNQSGADFYGAEGEIYFKPTPRYRIGVSGDYVRGRLKNLPSLPGREDAYGKRPF
NEISp0944_122     EMKLVRYNQSGADFYGAEGEIYFKPTPRYRIGVSGDYVRGRLKNLPSLPGREDAYGKRPF
NEISp0944_778     EMKLVRYNQSGADFYGAEGEIYFKPTPRYRIGVSGDYVRGRLKNLPSLPGREDAYGNRPF
NEISp0944_107     EMKLVRYNQSSADFYGAEGEIYFKPTPRYRIGVSGDYVRGRLKNLPSLPGREDAYGNRPF
NEISp0944_56      EMKLVRYNQSGADFYGAEGEIYFKPTPRYRIGVSGDYVRGRLKNLPSLPGREDAYGNRPF
NEISp0944_607     EMKLVRYNQSGADFYGAEGEIYFKPTPRYRIGISGDYVRGRLKNLPSLPGREDAYGNRPF
NEISp0944_318     EMKLVRYNQSGADFYGAEGEIYFKPTPRYRIGVSGDYVRGRLKNLPSLPGREDAYGNRPF
NEISp0944_134     EMKLVRYNQSGADFYGAEGEIYFKPTPRYRIGVSGDYVRGRLKNLPSLPGREDAYGKRPF
NEISp0944_129     EMKLVRYNQSGADFYGAEGEIYFKPTPRYRIGVSGDYVRGRLKNLPSLPGREDAYGNRPF
NEISp0944_18      EMKLVRYNQSGADFYGAEGEIYFKPTPRYRIGVSGDYVRGRLKNLPSLPGREDAYGNRPF
NEISp0944_1       EMKLVRYNQSGADFYGAEGEIYFKPTPRYRIGVSGDYVRGRLKNLPSLPGREDAYGNRPF
NEISp0944_381     EMKLVRYNQSGADFYGAEGEIYFKPTPRYRIGVSGDYVRGRLKNLPSLPGREDAYGKRPF
NEISp0944_157     EMKLVRYNQSGADFYGAEGEIYFKPTPRYRIGVSGDYVRGRLKNLPSLPGREDAYGNRPF
NEISp0944_60      EMKLVRYNQSGADFYGAEGEIYFKPTPRYRIGVSGDYVRGRLKNLPSLPGREDAYGNRPF
NEISp0944_606     EMKLVRYNQSGADFYGAEGEIYFKPTPRYRIGVSGDYVRGRLKNLPSLPGREDAYGNRPF
NEISp0944_908     EMKLVRYNQSGADFYGAEGEIYFKPTPRYRIGVSGDYVRGRLKNLPSLPGREDAYGNRPF
NEISp0944_130     EMKLVRYNQSGADFYGAEGEIYFKPTPRYRIGVSGDYVRGRLKNLPSLPGREDAYGNRPF
NEISp0944_46      EMKLVRYNQSGADFYGAEGEIYFKPTPRYRIGVSGDYVRGRLKNLPSLPGREDAYGNRPF
NEISp0944_23      EMKLVRYNQSGADFYGAEGEIYFKPTPRYRIGVSGDYVRGRLKNLPSLPGREDAYGKRPF
NEISp0944_499     EMKLVRYNQSGADFYGAEGEIYFKPTPRYRIGVSGDYVRGRLKNLPSLPGREDAYGNRPF
NEISp0944_81      EMKLVRYNQSGADFYGAEGEIYFKPTPRYRIGVSGDYVRGRLKNLPSLPGREDAYGNRPF
NEISp0944_41      EMKLVRYNQSGADFYGAEGEIYFKPTPRYRIGVSGDYVRGRLKNLPSLPGREDAYGKRPF
NEISp0944_3       EMKLVRYNQSGADFYGAEGEIYFKPTPRYRIGVSGDYVQGRLKNLPSLPGREDAYGNRPF
NEISp0944_553     EMKLVRYNQSGADFYGAEGEIYFKPTPRYRIGVSGDYVRGRLKNLPSLPGREDAYGNRPF
NEISp0944_271     EMKLVRYNQSGADFYGAEGEIYFKPTPRYRIGVSGDYVRGRLKNLPSLPGREDAYGNRPF
NEISp0944_82      EMKLVRYNQSGADFYGAEGEIYFKPTPRYRIGVSGDYVRGRLKNLPSLPGREDAYGKRPF
NEISp0944_20      EMKLVRYNQSGADFYGAEGEIYFKPTPRYRIGVSGDYVRGRLKNLPSLPGREDAYGNRPL
NEISp0944_2       EMKLVRYNQSGADFYGAEGEIYFKPTPRYRIGVSGDYVRGRLKNLPSLPGREDAYGNRPF
NEISp0944_9       EMKLVRYNQSGADFYGAEGEIYFKPTPRYRIGVSGDYVRGRLKNLPSLPGREDAYGNRPF
```

```
                  660       670       680       690       700       710
NEISp0944_35   IAQDDQNAPRVPAARLGFHLKASLTDRIDAHLDYYRVFAQNKLARYETRTPGHHMLNLGA
NEISp0944_39   IAQADQNAPRVPAARLGFHLNAALTDRIDAHLDYYRVFAQNKLARYETRTPGHHMLNLGA
NEISp0944_5    IAQADQNAPRVPAARLGFHLNAALTDRIDAHLDYYRVFAQNKLARYETRTPGHHMLNLGA
NEISp0944_75   IAQADQNAPRVPAARLGFHLNAALTNRIDAHLDYYRVFAQNKLARYETRTPGHHMLNLGA
NEISp0944_180  IAQADQNAPRVPAARLGFHLKASLTDRIDANLDYYRVFAQNKLARYETRTPGHHMLNLGA
NEISp0944_22   IAQDNQNAPRVPAARLGVHLKASLTDRIDANLDYYRVFAQNKLARYETRTPGHHMLNLGA
NEISp0944_25   IAQADQNAPRVPAARLGFHLKASLTDRIDANLDYYRVFAQNKLARYETRTPGHHMLNLGA
NEISp0944_142  IAQADQNAPRVPAARLGVHLKAALTDRIDANLDYYRVFAQNKLARYETRTPGHHMLNLGA
NEISp0944_135  IAQNDQNAPRVPAARLGVHLKASLTDRIDANLDYYRVFAQNKLARYETRTPGHHMLNLGA
NEISp0944_126  IAQDDQNAPRVPAARLGFHLKASLTDRIDANLDYYRVFAQNKLARYETRTPGHHMLNLGA
NEISp0944_901  IAQEDQNAPRVPAARLGVHLKASLTNRIDANLDYYRVFAQNKLARYETRTPGHHMLNLGA
NEISp0944_131  IAQDNQNAPRVPAARLGFHLKASLTDRIDANLDYYRVFAQNKLARYETHTPGHHMLNLGA
NEISp0944_777  IAQADQNAPRVPAARLGVHLKASLTDRIDANLDYYRVFAQNKLARYETRTPGHHMLNLGA
NEISp0944_207  IAQADQNAPRVPAARLGFHLKASLTDRIDANLDYYRVFAQNKLARYETRTPGHHMLNLGA
NEISp0944_198  IAQDNQNAPRVPAVRLGFHLKASLTDRIDANLDYYRVFAQNKLARHETRTPRHHMLNLGA
NEISp0944_863  IAQDDQNAPRVPAARLGVHLKASLTDRIDANLDYYRVFAQNKLARYETRTPGHHMLNLGA
NEISp0944_160  IAQDNQNAPRVPAARLGFHLKASLTDRIDANLDYYRVFAQNKLARYETHTPGHHMLNLGA
NEISp0944_859  IAQADQNAPRVPAARLGVHLKASLTDRIDANLDYYRVFAQNKLARYETRTPGHHMLNLGA
NEISp0944_912  IAQDDQNAPRVPAARLGFHLKASLTDRIDANLDYYRVFAQNKLARYETRTPGHHMLNLGA
NEISp0944_251  IAQADQNAPRVPASRLGIHLKASLTDRIDANLDYYRVFAQNKLARYETRTPGHHMLNLGT
NEISp0944_196  IAQDDQNAPRVPAARLGFHLKASLTDRIDANLDYYRVFAQNKLARYETRTPGHHMLNLGA
NEISp0944_263  IAQDDQNAPRVPAARLGFHLKASLTDRIDANLDYYRVFAQNKLARYETRTPGHHMLNLGA
NEISp0944_897  IAQADQNAPRVPAARLGFHLNVALTDRIDANLDYYRVFAQNKLARYETRTPGHHMLNLGA
NEISp0944_79   IAQDNQNAPRVPAARLGVHLKASLTDRIDANLDYYRVFAQNKLARYETRTPGHHMLNLGA
NEISp0944_13   IAQADQNAPRVPAARLGVHLKASLTDRIDANLDYYRVFAQNKLARYETRTPGHHMLNLGA
NEISp0944_574  IAQADQNAPRVPAARLGFHLKASLTERIDANLDYYRVFAQNKLARYETRTPGHHMLNLGA
NEISp0944_478  IAQDDQNAPRVPAARLGFHLKASLTDRIDANLDYYRVFAQNKLARYETRTPGHHMLNLGA
NEISp0944_347  IAQADQNAPRVPAARLGFHLKASLTDRIDANLDYYRVFAQNKLARYETRTPGHHMLNLGA
NEISp0944_177  IAQADQNAPRVPAARLGFHLKASLTDRIDANLDYYRVFAQNKLARYETRTPGHHMLNLGA
NEISp0944_140  IAQADQNAPRVPAARLGFHLKASLTDRIDANLDYYRVFAQNKLARYETRTPGHHMLNLGA
NEISp0944_534  IAQDDQNAPRVPAARLGVHLKASLTDRIDANLDYYRVFAQNKLARYETRTPGHHMLNLGA
NEISp0944_37   IAQADQNAPRVPAARLGFHLKASLTERIDANLDYYRVFAQNKLARYETRTPGHHMLNLGA
NEISp0944_176  IAQADQNAPRVPAARLGVHLKAALTDRIDANLDYYRVFAQNKLARYETRTPGHHMLNLGA
NEISp0944_907  IAQADQNAPRVPAARLGIHLKASLTDRIDANLDYYRVFAQNKLARYETRTPGHHMLNLGA
NEISp0944_12   IAQADQNAPRIPAARLGFHLKTSLTDRIDANLDYYRVFAQNKLARYETRTPGHHMLNLGA
NEISp0944_69   IAQDDQNAPRVPAARLGFHLKASLTDRIDANLDYYRVFAQNKLARYETRTPGHHMLNLGA
NEISp0944_373  IAQDDQNAPRVPAARLGFHLKASLTDRIDANLDYYRVFTQNKLARYETHTPGHHMLNLGA
NEISp0944_86   IAQNDQNAPRVPAARLGFHLKASLTDRIDANLDYYRVFAQNKLARYETRTPGHHMLNLGA
NEISp0944_821  ITQDNQNAPRVPAARLGVHLKASLTDRIDANLDYYRVFAQNKLARYETRTPGHHMLNLGA
NEISp0944_369  IAQADQNAPRVPAARLGVHLKASLTDRIDANLDYYRVFAQNKLARYETRTPGHHMLNLGA
NEISp0944_87   IAQADQNAPRVPAARLGVHLKAALTDRIDANLDYYRVFAQNKLARYETRTPGHHMLNLGA
NEISp0944_88   IAQADQNAPRVPAARLGFHLKASLTDRIDANLDYYRVFAQNKLARYETRTPGHHMLNLGA
NEISp0944_33   IAQDNQNAPRVPAARLGFHLKASLTDRIDANLDYYRVFAQNKLARYETRTPGHHMLNLGA
NEISp0944_70   IAQDDQNAPRVPAARLGFHLKASLTDRIDANLDYSRVFAQNKLARYETRTPGHHMLNLGA
NEISp0944_61   IAQADQNAPRVPAARLGVHLKASLTDLIDANLDYYRVFAQNKLARYETRTPGHHMLNLGA
NEISp0944_83   IAQADQNAPRVPAARLGVHLKASLTDRIDAHLDYYRVFAQNKLARYETRTPGHHMLNLGA
NEISp0944_122  IAQADQNAPRVPAARLGVHLKASLTDRIDAHLDYYRVFAQNKLARYETRTPGHHMLNLGA
NEISp0944_778  IAQDDQNAPRVPAARLGFHLKASLTDRIDANLDYYRVFAQNKLARYETRTPGHHMLNLGA
NEISp0944_107  IAQDDQNAPRIPAARLGFHLKASLTDRIDANLDYYRVFAQNKLARYETRTPGHHMLNLGA
NEISp0944_56   IEQADQNAPRVPAARLGFHLNAALTDRIDAHLDYYRVFAQNKLARYETRTPGHHMLNLGA
NEISp0944_607  IAQDDQNAPRVPAARLGVHLKASLTDRIDANLDYYRVFAQNKLARYETRTPGHHMLNLGA
NEISp0944_318  IAQDDQNAPRVPAARLGVHLKASLTDRIDANLDYYRVFAQNKLARYETRTPGHHMLNLGA
NEISp0944_134  IAQDDQNAPRVPAARLGFHLKASLTDRIDANLDYYRVFAQNKLARYETRTPGHHMLNLGA
NEISp0944_129  IAQDNQNAPRVPAARLGFHLKASLTDRIDANLDYYRVFAQNKLARYETRTPGHHMLNLGA
NEISp0944_18   IAQDDQNAPRVPAARLGVHLKASLTDRIDANLDYYRVFAQNKLARYETRTPGHHMLNLGA
NEISp0944_1    IAQDDQNAPRVPAARLGFHLKASLTDRIDANLDYYRVFAQNKLARYETRTPGHHMLNLGA
NEISp0944_381  IAQADQNAPRVPAARLGIHLKASLTDRIDANLDYYRVFAQNKLARYETRTPGHHMLNLGA
NEISp0944_157  IAQDDQNAPRVPAARLGFHLKASLTDRIDANLDYYRVFAQNKLARYETRTPGHHMLNLGA
NEISp0944_60   IAQDNQNAPRVPAARLGFHLKAALTDRIDANLDYYRVFAQNKLARYETRTPGHHMLNLGA
NEISp0944_606  IAQNDQNAPRVPAARLGFHLKASLTDRIDANLDYYRVFAQNKLARYETRTPGHHMLNLGA
NEISp0944_908  IAQDNQNAPRVPAARLGVHLKASLTDRIDANLDYYRVFAQNKLARYETRTPGHHMLNLGA
NEISp0944_130  IAQDDQNAPRVPAARLGVHLKASLTDRIDANLDYYRVFAQNKLARYETRTPGHHMLNLGA
NEISp0944_46   IAQDDQNAPRVPAARLGFHLKASLTDRIDANLDYYRVFAQNKLARYETRTPGHHMLNLGA
NEISp0944_23   IAQADQNAPRVPAAHLGVHLKASLTDLIDANLDYYRVFAQNKLARYETRTPGHHMLNLGA
NEISp0944_499  IAQDDQNAPRVPAARLGFHLKASLTDRIDANLDYYRVFAQNKLARYETRTPGHHMLNLGA
NEISp0944_81   IAQDDQNAPRVPAARLGFHLKASLTDRIDANLDYYRVFAQNKLARYETHTPGHHMLNLGA
NEISp0944_41   IAQADQNAPRVPAARLGVHLKASLTDRIDANLDYYRVFAQNKLARYETRTPGHHMLNLGA
NEISp0944_3    IAQDDQNAPRVPAARLGIHLKASLTDRIDANLDYYRVFAQNKLARYETRTPGHHMLNLGA
NEISp0944_553  IAQDNQNAPRVPAARLGFHLKASLTDRIDANLDYYRVFAQNKLARYETRTPGHHMLNLGA
NEISp0944_271  IAQDNQNAPRVPAARLGFHLKASLTDRIDANLDYYRVFAQNKLARYETRTPGHHMLNLGA
NEISp0944_82   IAQDDQNAPRVPAARLGFHLKASLTDRIDANLDYYRVFAQNKLARYETRTPGHHMLNLGA
NEISp0944_20   IAQADQNAPRVPAARLGVHLKAALTDRIDANLDYYRVFAQNKLARYETRTPGHHMLNLGA
NEISp0944_2    IAQDDQNAPRVPAARLGVHLKASLTDRIDANLDYYRVFAQNKLARYETRTPGHHMLNLGA
NEISp0944_9    IAQDDQNAPRVPAARLGVHLKASLTDRIDANLDYYRVFAQNKLARYETRTPGHHMLNLGA
```

```
                    720       730       740       750       760
NEISp0944_35   NYRRRTGYGEWNWYVKADNLFNQSVYAHSSFLSDTPQMGRSFTGGVNFKF
NEISp0944_39   NYRRNTRYGEWNWYVKADNLLNQSVYAHSSFLSDTPQIGRSFTGGVNFKF
NEISp0944_5    NYRRRTRYGEWNWYVKADNLFNQSVYAHSSFLSDTPQMGRSFTGGVNFKF
NEISp0944_75   NYRRRTGYGEWNWYVKADNLFNQSVYAHSSFLSDTPQMGRSFTGGVNFKF
NEISp0944_180  NYRRNTRYGEWNWYVKADNLLNQSVYAHSSFLSDTPQMGRSFTGGVNVKF
NEISp0944_22   NYRRNTRYGEWNWYVKADNLLNQSVYAHSSFLSDTPQMGRSFTGGVNVKF
NEISp0944_25   NYRRNTRYGEWNWYVKADNLLNQSVYAHSSFLSDTPQMGRSFTGGVNVKF
NEISp0944_142  NYRRNTRYGEWNWYVKADNLLNQSVYAHSSFLSDTPQMGRSFTGGVNVKF
NEISp0944_135  NYRRNTRYGEWNWYVKADNLLNQSVYAHSSFLSDTPQMGRSFTGGVNVKF
NEISp0944_126  NYRRNTRYGEWNWYVKADNLLNQSVYAHSSFLSDTPQMGRSFTGGVNVKF
NEISp0944_901  NYRRNTRYGEWNWYVKADNLLNQSVYAHSSFLSDTPQMGRSFTGGVNVKF
NEISp0944_131  NYRRNTRYGEWNWYVKADNLLNQSVYAHSSFLSDTPQMGRSFTGGVNVKF
NEISp0944_777  NYRRNTRYGEWNWYVKADNLLNQSVYAHSSFLSDTPQMGRSFTGGVNVKF
NEISp0944_207  NYRRNTRYGEWNWYVKADNLLNQYVYAHSSFLSDTPQMGRSFTGGVNVKF
NEISp0944_198  NYRRNTRYGEWNWYVKADNLLNQSVYAHSSFLSDTPQMGRSFTGGVNVKF
NEISp0944_863  NYRRNTRYGEWNWYVKANNLLNQSIYAHSSFLSDTPQMGRSFTGGVNVKF
NEISp0944_160  NYRRNTRYGEWNWYVKANNLLNQSIYAHSSFLSDTPQMGRSFTGGVNVKF
NEISp0944_859  NYRRNTRYGEWNWYVKADNLLNQSVYAHSSFLSDTPQMGRSFTGGVNVKF
NEISp0944_912  NYRRNTRYGEWNWYVKADNLLNQSVYAHSSFLSDTPQMGRSFTGGVNVKF
NEISp0944_251  NYRRNTRYGEWNWYVKADNLLNQSIYAHSSFLSDTPQMGRSFTGGVNVKF
NEISp0944_196  NYRRNTRYGEWNWYVKADNLLNQSVYAHSSFLSDTPQMGRSFTGGVNVKF
NEISp0944_263  NYRRNTRYGEWNWYVKADNLLNQSVYAHSSFLSDTPQMGRSFTGGVNVKF
NEISp0944_897  NYRRNTHYGEWNWYVKADNLLNQSVYAHSSFLSDTPQMGRSFTGGVNVKF
NEISp0944_79   NYRRNTRYGEWNWYVKADNLLNQSVYAHSSFLSDTPQMGRSFTGGVNVKF
NEISp0944_13   NYRRNTRFGEWNWYVKADNLLNQSVYAHSSFLSDTPQMGRSFTGGVNVKF
NEISp0944_574  NYRRNTRYGEWNWYVKADNLLNQSVYAHSSFLSDTPQMGRSFTGGVNVKF
NEISp0944_478  NYRRNTRYGEWNWYIKTDNLLNQSVYAHSSFLSDTPQMGRSFTGGVNVKF
NEISp0944_347  NYRRNTRYGEWNWYVKADNLLNQSVYAHSSFLSDTPQMGRSFTGGVNVKF
NEISp0944_177  NYRRNTRYGEWNWYVKADNLLNQSVYAHSSFLSDTPQMGRSFTGGVNVKF
NEISp0944_140  NYRRNTRYGEWNWYVKADNLLNQSVYAHSSFLSDTPQMGRSFTGGVNVKF
NEISp0944_534  NYRRNTRYGEWNWYVKADNLLNQSVYAHSSFLSDTPQMGRSFTGGVNVKF
NEISp0944_37   NYRRNTRYGEWNWYVKADNLLNQSVYAHSSFLSDTPQMGRSFTGGVNVKF
NEISp0944_176  NYRRNTRYGEWNWYVKADNLLNQSVYAHSSFLSDTPQMGRSFTGGVNVKF
NEISp0944_907  NYRRNTCYGEWNWYVKADNLLNQSVYAHSSFLSDTPQMGRSFTGGVNVKF
NEISp0944_12   NYRRNTRYGEWNWYVKADNLLNQSVYAHSSFLSDTPQMGRSFTGGVNVKF
NEISp0944_69   NYRRNTRYGEWNWYVKADNLLNQSVYAHSSFLSDTPQMGRSFTGGVNVKF
NEISp0944_373  NYRRNTRYGEWNWYVKADNLLNQSVYAHSSFLSDTPQMGRSFTGGVNVKF
NEISp0944_86   NYRRNTRYGEWNWYVKADNLLNQSVYAHSSFLSDTPQMGRSFTGGVNVKF
NEISp0944_821  NYRRNTRYGEWNWYVKADNLLNQSVYAHSSFLSDTPQMGRSFTGGVNVKF
NEISp0944_369  NYRRNTRYGEWNWYVKADNLLNQSVYAHSSFLSDTPQMGRSFTGGVNVKF
NEISp0944_87   NYRRNTRYGEWNWYVKADNLLNQSVYAHSSFLSDTPQMGRSFTGGVNVKF
NEISp0944_88   NYRRNTRYGEWNWYVKADNLLNQSVYAHSSFLSDTPQMGRSFTGGVNVKF
NEISp0944_33   NYRRNTRYGEWNWYVKADNLLNQSVYAHSSFLSDTPQMGRSFTGGVNVKF
NEISp0944_70   NYRRNTRYGEWNWYVKADNLLNQSVYAHSSFLSDTPQMGRSFTGGVNVKF
NEISp0944_61   NYRRNTRYGEWNWYVKADNLLNQSVYAHSSFLSDTPQMGRSFTGGVNVKF
NEISp0944_83   NYRRNTRYGEWNWYVKADNLLNQSVYAHSSFLSDTPQMGRSFTGGVNVKF
NEISp0944_122  NYRRNTRYGEWNWYVKADNLLNQSVYAHSSFLSDTPQMGRSFTGGVNVKF
NEISp0944_778  NYRRNTRYGEWNWYVKADNLLNQSVYAHSSFLSDTPQMGRSFTGGVNVKF
NEISp0944_107  NYRHNTRYGEWNWYVKADNLLNQSVYAHSSFLSDTPQMGRSFTGGVNVKF
NEISp0944_56   NYRRRTGYGEWNWYVKADNLLNQSVYAHSSFLSDTPQMGRSFTGGVNVKF
NEISp0944_607  NYRRNTRYGEWNWYVKADNLLNQSVYAHSSFLSDTPQMGRSFTGGVNVKF
NEISp0944_318  NYRRNTRYGEWNWYVKADNLLNQSVYAHSSFLSDTPQMGRSFTGGVNVKF
NEISp0944_134  NYRRNTRYGEWNWYVKADNLLNQSVYAHSSFLSDTPQMGRSFTGGVNVKF
NEISp0944_129  NYRRNTRYGEWNWYIKADNLLNQSVYAHSSFLSDTPQMGRSFTGGVNVKF
NEISp0944_18   NYRRNTRYGEWNWYVKADNLLNQSVYAHSSFLSDTPQMGRSFTGGVNVKF
NEISp0944_1    NYRRNTRYGEWNWYIKTDNLLNQSVYAHSSFLSDTPQMGRSFTGGVNVKF
NEISp0944_381  NYRRNTRYGEWNWYVKADNLLNQSVYAHSSFLSDTPQMGRSFTGGVNVKF
NEISp0944_157  NYRRNTRYGEWNWYIKTDNLLNQSVYAHSSFLSDTPQMGRSFTGGVNVKF
NEISp0944_60   NYRRNTRYGEWNWYVKADNLLNQSVYAHSSFLSDTPQMGRSFTGGVNVKF
NEISp0944_606  NYRRNTRYGEWNWYVKADNLLNQSVYAHSSFLSDTPQMGRSFTGGVNVKF
NEISp0944_908  NYRRNTRYGEWNWYVKADNLFNQSVYAHSSFLSDTPQMGRSFTGGVNVKF
NEISp0944_130  NYRRNTRYGEWNWYVKADNLLNQSVYAHSSFLPDTPQMGRSFTGGVNVKF
NEISp0944_46   NYRRNTRYGEWNWYVKADNLLNQSVYAHSSFLSDTSQMGRSFTGGVNVKF
NEISp0944_23   NYRRNTRYGEWNWYVKADNLLNQSVYAHSSFLSDTPQMGRSFTGGVNVKF
NEISp0944_499  NYRRNTRYGEWNWYVKADNLLNQSVYAHSSFLSDTPQMGRSFTGGVNVKF
NEISp0944_81   NYRRNTRYGEWNWYVKADNLLNQSVYAHSSFLSDTPQMGRSFTGGVNVKF
NEISp0944_41   NYRRNTRYGEWNWYVKADNLLNQSVYAHSSFLSDTPQMGRSFTGGVNVKF
NEISp0944_3    NYRRNTRYGEWNWYVKADNLLNQSVYAHSSFLSDTPQMGRSFTGGVNVKF
NEISp0944_553  NYRRNTRYGEWNWYVKADNLLNQSVYAHSSFLSDTPQMGRSFTGGVNVKF
NEISp0944_271  NYRRNTRYGEWNWYVKADNLLNQSVYAHSSFLSDTPQMGRSFTGGVNVKF
NEISp0944_82   NYRRNTRYGEWNWYVKADNLLNQSVYAHSSFLSDTPQMGRSFTGGVNVKF
NEISp0944_20   NYRRNTRYGEWNWYVKADNLLNQSVYAHSSFLSDTPQMGRSFTGGVNVKF
NEISp0944_2    NYRRNTRYGEWNWYVKADNLLNQSVYAHSSFLSDTPQMGRSFTGGVNVKF
NEISp0944_9    NYRRNTRYGEWNWYVKADNLLNQSVYAHSSFLSDTPQMGRSFTGGVNVKF
```
